# The Molecular Landscape of Medulloblastoma in Teenagers and Young Adults

**DOI:** 10.3390/cancers14010251

**Published:** 2022-01-05

**Authors:** Omar J. Mohammed, Maria Estevez Cebrero, Omar Ahmad, Andrew Peet, Richard G. Grundy, Anbarasu Lourdusamy

**Affiliations:** 1 Children’s Brain Tumour Research Centre, School of Medicine, University of Nottingham, Nottingham NG7 2RD, UK; omar.mohammed@nottingham.ac.uk (O.J.M.); mszme1@exmail.nottingham.ac.uk (M.E.C.); omarakramj@yahoo.com (O.A.); richard.grundy@nottingham.ac.uk (R.G.G.); 2 Institute of Cancer and Genomic Sciences, University of Birmingham, Birmingham B15 2SY, UK; A.Peet@bham.ac.uk; 3 Birmingham Women’s and Children’s Hospital NHS Foundation Trust, Birmingham B15 2TG, UK

**Keywords:** teenagers and young adults (TYA), medulloblastoma, DNA methylation, gene expression

## Abstract

**Simple Summary:**

Medulloblastoma is a malignant primary brain tumour that commonly occurs in children but also occurs in teenagers and young adults (TYA, aged 13–24 years) frequently enough to warrant attention. While remarkable progress has been made with paediatric tumours, TYAs with medulloblastoma represents an understudied population, lacking dedicated studies. The study aims to comprehensively characterise medulloblastoma in TYAs using data obtained from genomic technologies. It highlights that TYA medulloblastoma constitutes heterogenous molecular subgroups with distinct epigenetic and transcriptomic characteristics. Additionally, the prognostic gene signature-based score stratifies TYA patients into low- and high-risk groups and associates significantly with the outcomes. These results demonstrate unique molecular characteristics of TYA medulloblastoma that may contribute to the clinical differences at presentation between TYAs and other age groups. A better understanding of the TYA-specific biology might impact the treatment of those patients in the future.

**Abstract:**

Medulloblastoma (MB) is a childhood malignant brain tumour but also occurs in teenagers and young adults (TYA). Considering that MB is heterogeneous, this study aimed to define the molecular landscape of MBs in TYAs. We collated more than 2000 MB samples that included 287 TYA patients (13–24 years). We performed computational analyses consisting of genome-wide methylation and transcriptomic profiles and developed a prognostics model for the TYAs with MB. We identified that TYAs predominantly comprised of Group 4 (40%) and Sonic Hedgehog (SHH)-activated (33%) tumours, with Wingless-type (WNT, 17%) and Group 3 (10%) being less common. TYAs with SHH tumours displayed significantly more gene expression alterations, whereas no gene was detected in the Group 4 tumours. Across MB subgroups, we identified unique and shared sets of TYA-specific differentially methylated probes and DNA-binding motifs. Finally, a 22-gene signature stratified TYA patients into high- and low-risk groups, and the prognostic significance of these risk groups persisted in multivariable regression models (*P* = 0.001). This study is an important step toward delineating the molecular landscape of TYAs with MB. The emergence of novel genes and pathways may provide a basis for improved clinical management of TYA with MB.

## 1. Introduction

The teenagers and young adult (TYA) cancer population encompasses patients aged 13–24 years old at cancer diagnosis [1]. In the UK, TYAs account for approximately 2500 new cancer cases every year, and cancer is the leading cause of death by disease in this population [2]. Overall cancer incidence increased in the TYA age group by more than a quarter (28%) in the UK since the early 1990s [2]. TYAs experience a distinct set of challenges when presented with a cancer diagnosis, including inequitable access to oncology services [3]. These patients are often lost in cancer care as they age out of the paediatric practice but do not seem to fit in with the older adult practice [4]. Over the last several years, the unique needs of TYAs with cancer have been recognised, and it has emerged as a growing field of research [3,5]. Despite this, survival gains in this patient population have improved only modestly compared with older adults and children with cancer.

TYAs present with a spectrum of cancers distinct from children and older adults. More than any other malignancies, central nervous system (CNS) tumours demand a focused approach in TYAs because of their complexity and prognosis [6]. Even benign primary CNS tumours can be life-threatening because of their brain-damaging effects, local infiltration, and for some, a tendency to undergo malignant transformation over time. CNS tumours are among the top five common cancers in the TYA population [2]. These tumours remain a major contributor to morbidity and mortality as the third most common causes of cancer-related death among TYAs [7]. While advancement in cancer genomics has led to an increased knowledge of CNS tumour biology in children and adults, TYAs continue to be an understudied population.

Medulloblastoma (MB) is the most common malignant primary brain tumour in children, although, 20% of all MBs occur in patients older than 15 years of age. It has long been known that MB is a very heterogeneous disease with varied clinical outcomes. Based on gene expression or DNA methylation profiling, numerous studies showed that MB could be classified into four discrete subgroups: Wingless activated (WNT), Sonic Hedgehog (SHH), Group 3, and Group 4 [8,9,10]. Recent integrated analysis has suggested the existence of further MB subtypes within each subgroup, two WNT subtypes: WNTα and WNTβ; four SHH subtypes: SHHα, SHHβ, SHHγ, and SHHδ; three Group 3 subtypes: 3α, 3β, and 3γ; and three Group 4 subtypes: group 4α, group 4β, and group 4γ [11]. Among subgroups, only Group 3 MBs predominantly occur in children, whereas all other three subgroups occur both in children and young adults. TYA patients with MB may have a slightly different course of disease biology when compared with children and adults. The data on clinical outcomes and molecular features are scarce in TYAs, and to date, no comprehensive characterisation of MB in the TYA population has been described.

To address this knowledge gap, we outline the landscape of TYA medulloblastoma in this study. We conducted a systematic data collection and investigated the distribution and clinical characteristics of MB in the TYA population. Through rigorous bioinformatic approaches, we aim to identify TYA-specific transcriptional and DNA methylation alterations in MB subgroups. Subsequently, we evaluated whether a panel of genes can stratify TYAs and assessed its prognostic values for TYAs with MB.

## 2. Materials and Methods

### 2.1. Collection of Datasets

Following an extensive screening of literature, we collected 2487 MB samples from seven potentially eligible studies: the PubMed ID (https://pubmed.ncbi.nlm.nih.gov/ accessed on 7 February 2020)—20823417 [9], 22722829 [12], 22832581 [13], 26919435 [14], 28545823 [15], 28609654 [11], 29753700 [16]. Publications were eligible if they met the following criteria: (1) full-text articles written in English and (2) cohort studies with publicly available data. We removed all 285 samples from PMID: 22832581 as these samples were included in the study, PMID: 28609654. We considered only primary tumour samples from MB patients. Accounting for duplicate samples of the same patient in any study and missing age information, the final collection comprised a total of 2061 unique tumour samples.

### 2.2. Microarray Gene Expression Analysis

#### 2.2.1. Pre-Processing

We obtained the previously published gene expression profiling of 763 primary MB samples from the Gene Expression Omnibus (GEO) database (GEO Series accession number: GSE85217; PMID: 28609654) [11,17]. We excluded the samples with missing ages (n = 34) and calculated gene-level (core meta-probeset) expression values for the 729 Affymetrix Human Gene 1.1 ST array CEL files using Affymetrix Power Tools (APT) [18]. Arrays were normalised using the Robust Multi-array Average (RMA) approach, which included RMA background correction, quantile normalisation, log transformation, and probeset summarisation. Detection above background (DABG) was performed at both the probe and the probeset level using GC-matched background probes, and low variance probe sets were excluded. Normalised expression values on a log_2_ scale were used for subsequent analyses. The expression levels were mapped from probe sets to unique genes according to the corresponding annotation. We selected the probe set with the highest mean expression value when multiple probe sets were mapped to the same gene.

#### 2.2.2. Differential Gene Expression Analysis

Differential expression analysis was performed to identify genes associated with TYAs by comparing the expression levels in TYAs with those in other age groups. Differential expression was detected by a moderated t-statistic based on the empirical Bayesian method [19]. The *p*-values obtained from moderated *t*-test were corrected for multiple hypotheses using Benjamini and Hochberg algorithm (false discovery rate, FDR at 5% threshold) [20].

#### 2.2.3. Rank-Rank Hypergeometric Overlap Analysis

To compare the TYA associated genes of four molecular subgroups, we used a rank-rank hypergeometric overlap (RRHO) mapping, an unbiased method to uncover the concordances and discordances between two similarly ranked lists [21]. Briefly, for a pair of subgroups, the full list of genes is ranked according to their fold change from the most down-regulated to the most up-regulated gene. Then, an intersection of shared genes is performed, and the analysis of the ranking order of genes is performed with a hypergeometric test.

The visual output of this analysis is an RRHO level map, where the hypergeometric *p* value for enrichment of k overlapping genes is calculated for all possible threshold pairs for each experiment, generating a matrix where the indices are the current rank in each experiment. *p* values for each test are then log-transformed and reported on a heatmap to display the degrees of similarities according to four quadrants representing the concordance or the discordance in gene ranking in the two differential expression analysis (e.g., up-regulated in one subgroup and down-regulated in the other).

#### 2.2.4. Survival Analysis and Prognostic Signature Construction

We selected differentially expressed genes between TYAs and other age groups for the survival analysis. Cox proportional hazards regression analysis was performed to examine the correlation between gene expression and overall survival (OS). The “*survival*” R package v3.2.7 was utilised to calculate log-rank *p* values, hazard ratios (HR), and 95% confidence intervals (CI). In addition, the survival differences between MB patient groups based on the high- and low expression of the selected gene were visualised by generating Kaplan-Meier survival plots.

Survival-related genes were analysed by the least absolute shrinkage and selection operator (*LASSO*)–penalised multivariable Cox regression to identify a prognostic gene signature for the TYAs with MB [22]. To identify a multivariable model incorporating gene expression data and overall survival of MB patients, the R package *glmnet* (v4.1.1) was used with the LASSO penalty (α = 1), 10,000 iterations and 10-fold cross-validation to find the optimal value of λ. The variables (genes) with non-zero coefficients were retained and the regularised coefficients at the chosen value of λ were saved as the survival model.

By combining the expression values of prognostic genes weighted by their beta coefficients, a risk score for each patient was constructed as follows: Risk score = $\overset{n}{\underset{i=1}{\sum }}\beta_{i}\times exp_{i}$, where *n* is the number of prognostic genes *exp_i_* the expression value of gene *i*, and *β_i_* the regression coefficient of gene *i* in the univariate Cox regression analysis. Using the median risk score as a cut-off value, MB patients were classified into high- and low-risk groups. The Kaplan-Meier method was used to assess the differences in survival time of low- and high-risk TYA patients, and the log-rank test was used to determine the statistical significance of observed differences between groups. Multivariable Cox regression analysis was used to assess whether the risk score was independent of other clinical features. In addition, the predictive accuracies of the variables were assessed with the Harrell’s concordance index (C-index) in both univariate and multivariate analyses [23]. To internally validate the final Cox proportional hazard model, 1000 computations with random selections of samples from TYA patients with multiple available samples were run.

### 2.3. DNA Methylation Data Analysis

Clinical and DNA methylation data from tumour samples were collected from the GEO database (GEO Series accession number: GSE85212) for 763 MB patients [11]. The DNA methylation data utilised for this study were normalised and samples with no age information were excluded from the analyses. We retained a total of 321,174 probes after stringent filtering procedures, as previously described. β-values range from 0 to 1, with 0 indicating no DNA methylation and 1 indicating complete DNA methylation.

Differentially methylated probes (DMPs) were identified using the R package “*ChAMP*” (v2.20.1), which performs the moderated *t*-test based on the empirical Bayesian method [24]. DMPs with FDR adjusted *p*-values < 0.05 were considered statistically significant. Probes were annotated using the Infinium HumanMethylation450 Bead Chip annotation file, which provides information regarding a probe’s location in a known enhancer or DNase I hypersensitive site (DHS) based on experimental data from ENCODE. The reported sensitivity of the Infinium HumanMethylation450 Bead Chip assay is 0.20, thus, probes with a Δβ magnitude 0.20 or greater were considered unlikely to be false positives.

We employed the Hypergeometric Optimization of Motif Enrichment (HOMER) v4.11 (http://homer.ucsd.edu/homer/ accessed on 18 January 2021) to search for known and de novo DNA binding sequences (8–20 bp motifs) with the perl script findMotifGenome.pl using the following criteria: hg38 genome, 200 bp upstream and downstream from each CpG site, and with expected genome-wide distribution of probes in the 450 K array as background.

### 2.4. Gene Ontology Enrichment Analysis

Gene set enrichment analysis (GSEA) was performed using the *javaGSEA* application version 4.0.2 [25]. The gene list output from the differential expression analysis with an eBayes adjusted moderated t-statistic linear regression model was ranked by calculating a rank score of each gene as −log_10_ (*p*-value) × sign (FC), in which FC is the fold change (expressed as log_2_(expression in TYA/expression in other age groups)) and the sign depends on whether the gene is up-regulated or down-regulated. A pre-ranked GSEA analysis was performed using 1000 permutations. The gene sets from *MSigDB* collections, C5: Gene Ontology (GO) database were used for the GSEA analysis [26].

For each subgroup, those DMPs which both satisfied the Bonferroni-adjusted *p*-value threshold and had a change in methylation of 0.20 or greater (lesser) were analysed using the *Gprofiler* GO enrichment analysis to identify possible enriched terms in the biological process (BP) [27].

## 3. Results

An overview of the methods and datasets used in our study is given in Figure S1. We collected clinical information for 2487 MB patients from seven studies following an extensive literature search. We removed all samples from one study [13] as these samples were included in another study [11] and retained patients with age and MB subgroup details. The supplementary figure displays the distribution of molecular subgroups and age across the remaining six studies (Figure S2). The Study 1 comprised of tumour samples from 53 MB patients aged (1–39) years, 74 MB patients aged (3.2–16.7) years in Study 2, 48 MB patients aged (1–49) years in Study 3, 729 MB patients aged (0.24–56.8) years in Study 4, 425 MB patients aged (0.24–15.97) years in Study 5, and 732 MB patients aged (0.1–50.1) years in Study 6. All six studies contained four molecular subgroups, of which, the Group 4 MB subgroup accounted for 43% in Study 4 and 38% in Study 6 (Figure S2). Upon merging data from six studies, SHH tumours were distributed across all age groups, whereas most Group 4 tumours (80%) were distributed between 4 and 15 years of age (Figure S3A). Interestingly, most of the WNT tumours (90%) occurred in MB patients aged less than 19 years old and Group 3 tumours (90%) in patients aged less than 11 years old. The final collection comprised a total of 2061 unique MB patients with a median age of 7.3 years (range: 0.1–56.8), of which 62% were from children and 14% were from TYAs (Figure S3B). The most common molecular subgroup was Group 4 (n = 799, 38.8%). We found a comparable distribution of SHH (n = 584, 28.3%) and Group 3 (n = 495, 24%) subgroups, but a relatively low proportion of the WNT subgroup (n = 183, 8.9%) in the data collection (Figure S3B).

**Figure 1:**
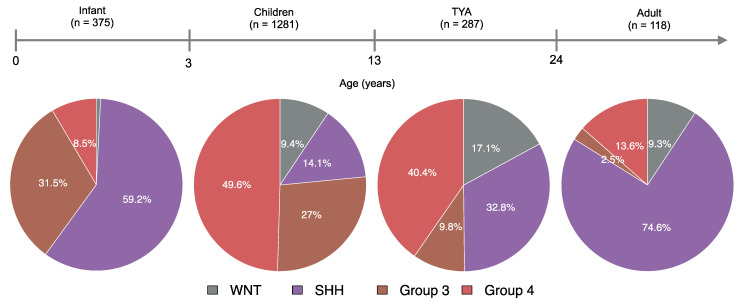
Comparative analysis of medulloblastoma patients by age and molecular subgroups. Four pie charts describing the distribution of molecular subgroups for medulloblastomas by age group: 1) Infant (<3 years), 2) Children (4–12 years), 3) Teenager and young adult, TYA (13–24 years), and 4) Adult (>24 years). Molecular subgroup and age data were collected from published studies that resulted in a total of 2061 medulloblastoma patients.

We found significant differences between age and the MB molecular subgroups (*P* = 3.1 × 10^−47^, Kruskal-Wallis rank-sum test, Figure S4). Molecularly, infants and adults were primarily of the SHH subgroup, with WNT tumours in infants (0.8%) and Group 3 tumours in adults (2.5%) forming a minority of cases (Figure 1). Although well represented, four molecular subgroups differed significantly between children and TYA age groups (*P* < 2.2 × 10^−16^, Fisher’s Exact Test). In TYAs, Group 4 tumours accounted for 40.4% and SHH tumours for 32.8%. When compared with children (27%), Group 3 tumours were less common (9.8%) in the TYA age group. Interestingly, the WNT tumours were more frequent in TYAs when we compared across all age groups (Figure 1).

### 3.1. Gene Expression Analysis Reveals TYA-Specific Transcriptional Profiles in MB 

**Figure 2:**
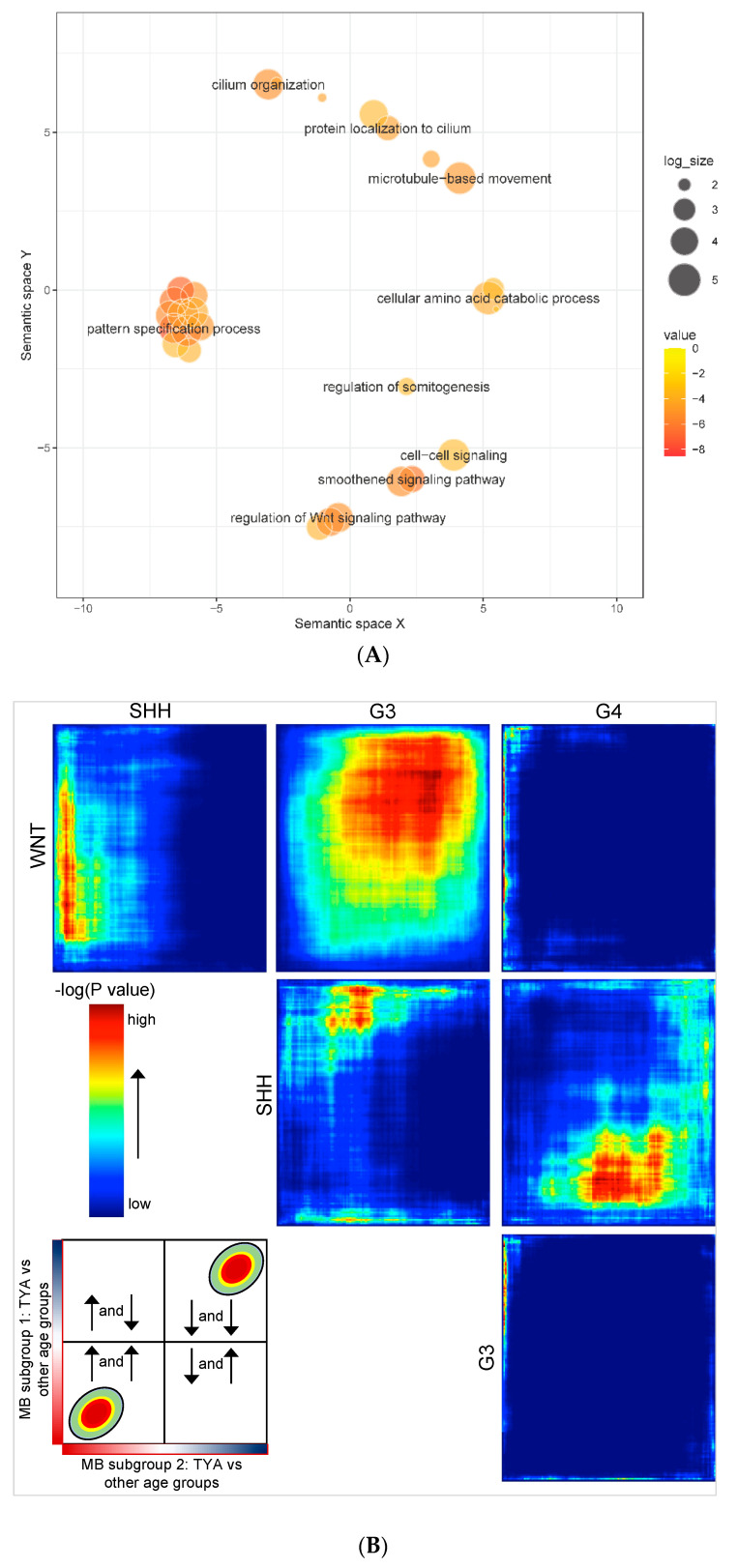

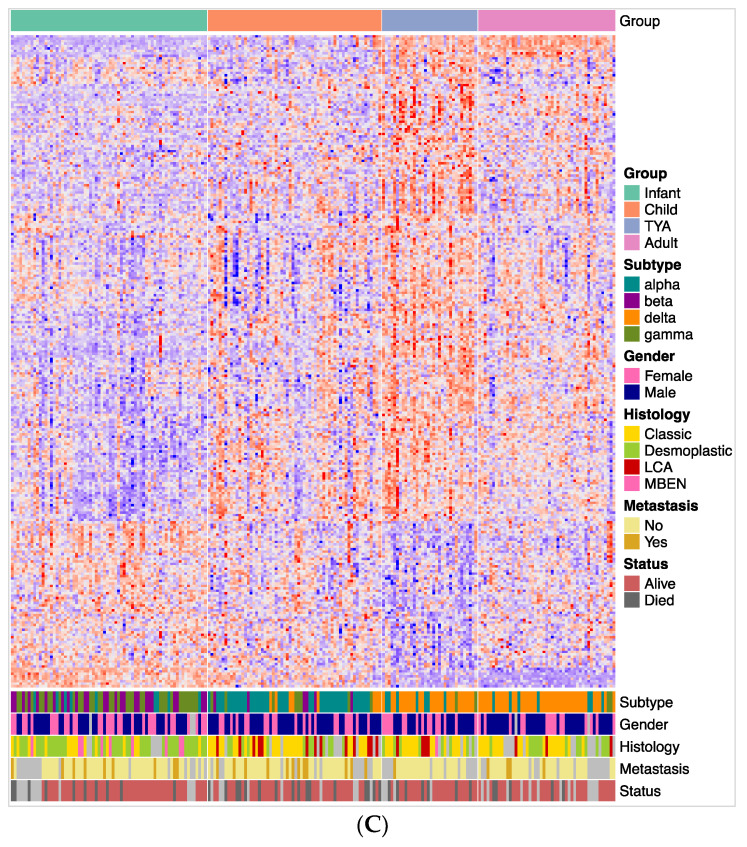
Comparison of differential expression patterns of TYAs in medulloblastoma molecular subgroups. (**A**) Summary of gene ontology (GO) biological processes derived from the enrichment analysis of up-regulated genes in teenagers and young adults (TYA) medulloblastoma. X- and Y-axes represent a two-dimensional annotation space derived from a multi-dimensional scaling procedure used on a matrix of GO terms’ semantic similarities. By employing this visualisation method, similar biological categories will cluster together. Bubble colour represents the *P*-value obtained from GO enrichment analysis (value) and bubble size relates to the frequency of GO terms in the GO annotation database (log_size). (**B**) Rank-rank hypergeometric overlap (RRHO) maps comparing pairs of medulloblastoma (MB) subgroups with expression differences between TYAs and other age groups. Each pixel represents the overlap between the TYA transcriptome of two MB subgroups with the significance of overlap (−log_10_(*p* value) of a hypergeometric test; step size 200) colour coded. G3 and G4 represent Group 3 and Group 4 MB subgroups. Schematic indicating interpretation of RRHO test plots (bottom-left). A hot spot in the bottom-left corner indicates overlap in genes up-regulated in TYA in both MB subgroups 1 and 2. A hot spot in the top-right corner indicates overlap in genes down-regulated in TYAs in both MB subgroups 1 and 2. A hot spot in the top-left indicates overlap in genes up-regulated in TYAs in MB subgroup 2 and down-regulated in TYAs in MB subgroup 1. A hot spot in the bottom-right indicates overlap in genes down-regulated in TYAs in MB subgroup 2 and down-regulated in TYAs in MB subgroup 1. (**C**) Heatmap plot showing expression of genes that are significantly altered between TYAs and other age groups in SHH tumours. Row represents a gene, and the column indicates the sample. The normalised expression value of each gene is indicated by colour intensity, with red/blue representing high/low expression.

To investigate whether MB shows TYA-specific transcriptional differences, we analysed gene expression profiles from 729 MB primary tumours from a single study that comprised 119 infant, 438 children, 108 TYAs, and 64 adult MB patients. Comparing TYAs and other age groups, we found significant transcriptional changes in 1002 genes with 56% showing up-regulation in TYAs (FDR-corrected *p*-value < 0.05, Table S1). Gene Ontology (GO) biological processes enrichment for the up-regulated genes in TYAs revealed that the pattern specification process (FDR = 2.84 × 10^−5^), the smoothened signalling pathway (FDR = 3.02 × 10^−4^), the Wnt signalling pathway (FDR = 1.50 × 10^−3^), the cilium organization (FDR = 1.50 × 10^−3^), and the cellular amino acid catabolic process (FDR = 1.26 × 10^−2^, Figure 2A and Table S2). The cilium landscape genes included intraflagellar transports (*IFT57* and *IFT80*), cilia and flagella associated proteins (*CFAP52*, *CFAP53*, and *CFAP54*), and EvC ciliary-complex subunits (*EVC* and *EVC2*). The Wnt signalling pathway genes that showed significant up-regulation in TYAs included *DKKL1*, *DKK2*, *FZD2*, *FZD10*, *WNT11*, and *WIF1*. Thus, differential expression analysis produced robust results, warranting further refined investigation.

Next, we were interested in a more rigorous comparison of TYAs across MB subgroups to determine (dis)similarities in gene expression patterns and shared pathway and biological processes enrichment. To obtain more detailed information, we employed an unbiased rank-rank hypergeometric overlap (RRHO) analysis, which is a genome-wide approach that compares two equally ranked datasets using a threshold-free algorithm. We applied RRHO, comparing a signed, *p* value-ranked list of differentially expressed genes between TYAs and other age groups in each MB subgroup. We found a high degree of overlap between the WNT and the Group 3 subgroup (maximum Fisher’s exact test *P* < 1 × 10^−160^, Figure 2B); we confirmed this result using Spearman’s correlation. There was a significant positive correlation in expression alternation (fold change on log_2_ scale) for TYA-genes in the WNT subgroup to the expression changes of TYA-genes in the Group 3 subgroup (ρ = 0.16; *P* < 2.2 × 10^−16^). However, in contrast, we found a small degree of overlap of the TYA genes between the Group 3 and the Group 4 subgroups, and between the WNT and the Group 4 subgroups. Interestingly, TYA-genes showed unique expression changes in SHH when compared to WNT, Group 3, and Group 4, particularly, opposite overlapping patterns in Group 3 and Group 4.

Given that the SHH subgroup showed considerable overlap in TYA gene expression patterns with the other three MB subgroups, we next focused exclusively on the SHH subgroup. A direct comparison restricted to only SHH tumours exhibited a distinct TYA transcriptional signature from other age groups (Figure 2C). To gain insight into the biology of TYAs with SHH tumours, we next performed gene set enrichment analysis (GSEA) on differentially expressed genes between TYAs and other age groups with SHH tumours. Similar to our results obtained from the analysis of the entire dataset, GSEA demonstrated significant enrichment for genes associated with the processes of the assembly and arrangement of constituent parts of a cilium (axoneme assembly, microtubule bundle formation) and cilium movement (Table S3). Besides, genes involved in rRNA modification (FDR = 6.02 × 10^−5^), non-coding RNA processing (FDR = 5.42 × 10^−5^), mitochondrial gene expression (FDR = 1.47 × 10^−4^), and smoothened signalling pathway (FDR < 0.041) showed significant enrichment in the SHH tumours of TYAs.

### 3.2. TYA-Specific DNA Methylation Alternations in MB

**Figure 3:**
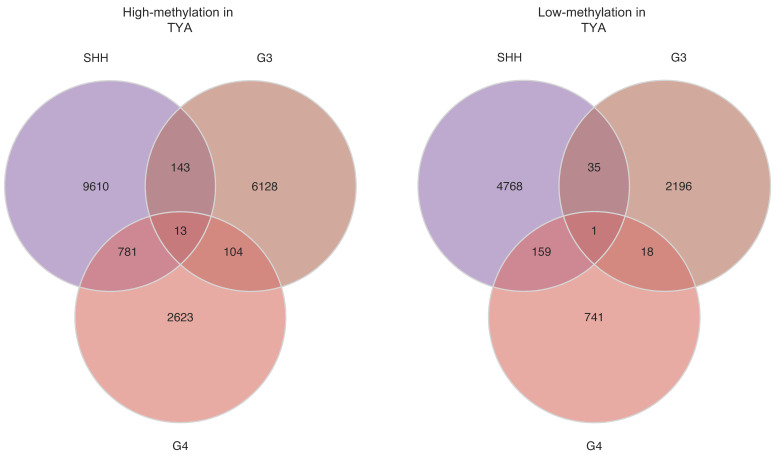

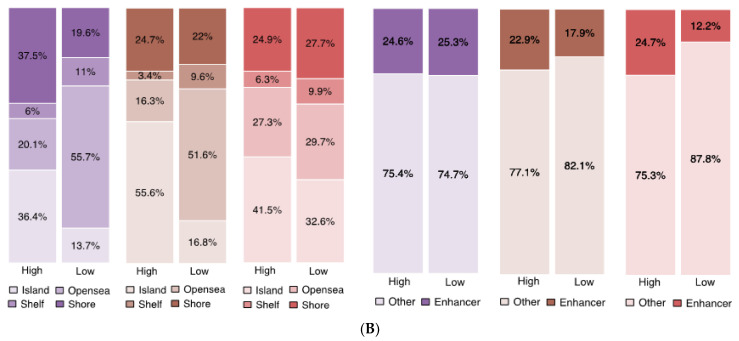
DNA methylation differences in TYAs across medulloblastoma molecular subgroups. (**A**) Intersection of differentially methylated probes (DMPs) in each of the three medulloblastoma (MB) subgroups. DMPs with high methylation levels in TYAs relative to other age groups (left) and DMPs with low methylation levels in TYAs relative to other age groups (right). All probes with a significant FDR adjusted *p*-value (<0.05) were compared to determine those shared between different MB subgroups and those unique to a particular subgroup. (**B**) Distribution of DMPs with high methylation levels in TYAs relative to other age groups (“High”), and with low methylation levels in TYAs (“Low”) in the three MB subgroups. Distribution of probes characterised as belonging to island, shore, shelf, and open-sea regions in the genome (left). Percentages of probes mapped to enhancer regions or elsewhere in the genome (right).

To characterise and compare the landscapes of abnormal DNA methylation in TYAs, we identified cytosine-phosphate-guanine sites (CpGs) that were significantly differentially methylated in TYAs with other age groups in each MB subgroup separately. The number of differentially methylated probes (DMP) varied for each MB subgroup (Figure 3A). The SHH tumours were notable for an extremely large number of DMPs. Of the 15,510 DMPs found in SHH tumours, more than 68% showed high methylation in TYAs compared to other age groups, while 4963 DMPs had low methylation levels (FDR-corrected *p*-value < 0.05). In Group 3, 6388 DMPs with high methylation and 2250 with low methylation were found in TYAs, whereas 3521 high and 919 low methylations TYA-specific DMPs were found in the Group 4 subgroup. 

The majority of DMPs in TYAs relative to other age groups were unique to a particular MB subgroup, although 13 probes significantly showed high methylation levels in TYAs in all three MB subgroups (Table S4). Similarly, a primarily unique set of probes with low methylation characterised TYAs in each subgroup, but all three MB subgroups shared only one probe, cg14223618 (Chr7:72711976). Using the Illumina 450 K probe annotation package, we found six genes (*CACNB2*, *HOXA1*, *NR2E1*, *PHOX2B*, *TACSTD2*, and *UCN*) that were associated with highly methylated DMPs in TYAs in all three MB subgroups. Interestingly, these genes were enriched for several nervous system processes, including neuron differentiation (*HOXA1*, *NR2E1*, *PHOX2B*, and *UCN*). 

We also assessed DMPs for location in the genome (Figure 3B). DMPs with high methylation levels in TYAs were primarily localised to CpG islands across the MB subgroup: 36.4% in SHH, 55.6% in Group 3, and 41.5% in Group 4. In contrast, probes with low methylation in TYAs were primarily located in the open-sea region (>4 kb from CpG islands): 55.7% in SHH and 51.6% in Group 3. Interestingly, CpG probes with low methylation levels in TYAs showed a unique distribution in the Group 4 subgroup: 32.6% in CpG island, 29.7% in open-sea (>4 kb from CpG islands), 27.7% in shores (<2 kb from CpG islands), and 9.9% shelf (2–4 kb from CpG islands). We also assessed the number of DMPs mapped to known enhancer regions to probe the potential functional consequences of TYA-specific differential methylation. Across Group 3 and Group 4 MB subgroups, higher percentages of probes with high methylation were in enhancer regions to low methylation probes in TYAs.

Several DMPs were identified where methylation differed at least 20% between TYAs and other age groups (|Δβ| ≥ 0.20). A total of 3320 DMPs met this criterion in the SHH subgroup, 945 DMPs in Group 3, and 237 DMPs were found in the Group 4 subgroup (Table S5). Of these 3320 DMPs in the SHH subgroup, more than 84% showed high methylation in TYAs and mapped to 642 unique genes enriched in diverse biological processes including developmental process (FDR = 1.05 × 10^−23^), neuron differentiation (FDR = 1.47 × 10^−17^), regionalisation (FDR = 2.69 × 10^−17^), cell fate commitment (FDR = 8.25 × 10^−17^), cell-cell signalling (FDR = 6.44 × 10^−9^), and stem cell differentiation (FDR = 3.37 × 10^−6^, Table S6). In contrast, DMPs with low-methylation in TYAs of the SHH subgroup showed significant enrichment for genes involved in sensory perception, G protein-coupled receptor signalling pathway, cell communication, and signal transduction (Table S6). In the Group 3 subgroup, 460 DMPs showed high methylation and mapped to genes associated with brain development (FDR = 4.88 × 10^−4^), transcription by RNA polymerase II (FDR = 6.26 × 10^−4^), and positive regulation of RNA metabolic process (FDR = 3.27 × 10^−3^), whereas DMPs with low methylation were enriched for extracellular matrix organization (FDR = 3.74 × 10^−5^) genes (Table S7). The majority of DMPs of the Group 4 subgroup (92.4%) showed high methylation in TYAs, however, displaying no significant enrichment with GO biological processes even at a less stringent threshold (FDR < 0.25).

**Figure 4:**
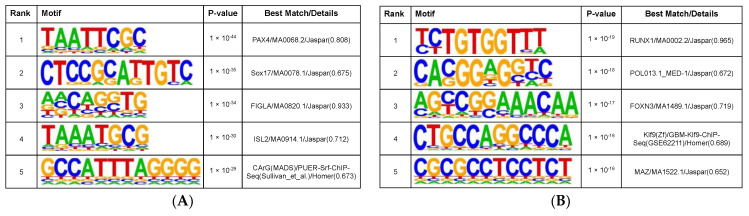
DNA methylation differences in TYAs across medulloblastoma molecular subgroups. (**A**) Top five binding motifs identified by HOMER based on DMPs with high methylation levels in TYAs in the entire dataset. (**B**) Top five binding motifs identified by HOMER based on DMPs with low methylation levels in TYAs in the entire dataset.

To identify sequence-specific transcription factor (TF) binding sites near DMPs, we performed an in-depth motif analysis. We first combined all DMPs that showed high methylation levels in TYAs in three MB subgroups: SHH, Group 3, and Group 4, and identified binding motifs significantly enriched around DMPs (n = 19,402). Across MB subgroups, the motif signatures corresponding to the homeodomain proteins, *PAX4*, *ISL2*, *LHX2*, and *PAX5* were enriched (Figure 4A, https://data.mendeley.com/datasets/f3w4vgwmsx/, accessed on 20 December 2021). The motif signature for the tumour suppressor protein, *SMAD4*, which plays a crucial role in the control of cell cycle, cell differentiation, and TGF-beta signalling pathway, was also significantly enriched (geometric test, *P* = 1.00 × 10^−20^). When we performed motif analyses separately for each MB subgroup, the motif signature for the zinc finger protein, *ZBTB14* (also known as *ZFP161*) was significantly enriched in all three subgroups: SHH (*P* = 1.00 × 10^−19^), Group 3 (*P* = 1.00 × 10^−25^), and Group 4 (*P* = 1.00 × 10^−12^), among DMPs that showed high methylation in TYAs. Besides, we identified several motifs that were enriched in two molecular subgroups, such as *GCM2* in SHH and Group 3, *MED1* motif in SHH and Group 4, and *GCM1* in Group 3 and Group 4 subgroups. The Glial Cell Missing (GCM) TFs, *GCM1* and *GCM2*, form a novel family of TFs with a conserved N-terminal GCM motifs and play important roles in development [28]. Interestingly, the mediator complex subunit 1 (*MED1*), a candidate tumour suppressor gene contains an N-terminal 5-methylcytosine binding domain that allows binding to methylated DNA and are known to be involved in transcriptional repression and chromatin stabilisation [29]. Finally, we found several subgroup-specific motifs significantly enriched in DMPs with high methylation levels in TYAs compared to other age groups. These motifs included those associated with the TFs, *E2F2* (*P* = 1.00 × 10^−18^) in the SHH subgroup; *MEIS3* (*P* = 1.00 × 10^−26^) and *ZIC2* (*P* = 1.00 × 10^−22^) in the Group 3 subgroup; and *SP1* (*P* = 1.00 × 10^−19^) in the Group 4 subgroup. *E2F2* plays a critical role in the control of cell cycle and participates in regulation of numerous genes; *MEIS3* controls the accessibility at Hox-regulated promoters and is involved in the hindbrain developmental program; the transcriptional repressor, *ZIC2*, is a master regulator of neurogenesis and is enriched at the enhancers of both active and poised genes in embryonic stem cells; and the zinc finger TF, SP1 binds to GC-rich motifs of several promoters and is involved in chromatin remodelling, cell growth, cell differentiation, and apoptosis [30,31,32,33].

We next identified significant motifs enriched near DMPs that showed low methylation in TYAs (n = 7918) when compared to other age groups with MBs (Figure 4B, https://data.mendeley.com/datasets/f3w4vgwmsx/, accessed on 20 December 2021). No binding motifs shared by all three MB subgroups were found. Only the motifs associated with TFs, *HIC1* (*P* = 1.00 × 10^−17^ in SHH and *P* = 1.00 × 10^−16^ in Group 4), *HINFP* (*P* = 1.00 × 10^−13^ in SHH and *P* = 1.00 × 10^−14^ in Group 4), *RUNX2* (*P* = 1.00 × 10^−13^ in SHH and *P* = 1.00 × 10^−21^ in Group 4), and *ZNF682* (*P* = 1.00 × 10^−22^ in SHH and *P* = 1.00 × 10^−19^ in Group 4) were found in SHH and Group 4 subgroups. The histone H4 transcription factor (*HINFP*) interacts with methyl-CpG-binding protein-2 (*MBD2*), a component of the MeCP1 histone deacetylase (HDAC) complex and plays a role in DNA methylation and transcription repression [34]. Hypermethylated in cancer 1 (*HIC1*) is an evolutionarily conserved transcriptional repressor that functions as a growth regulatory gene and its knockdown in mice contributes to the formation of MB [35]. We also found several subgroup-specific motifs enriched near low methylated DMPs in TYAs. These motifs included the binding sequence associated with TFs, *SMAD3*, *REST*, *ZIC3*, *FOXD3*, and *TFAP2C* in the SHH subgroup; *ATF1*, *HOXA11*, and *HNF4G* in the Group 3 subgroup; and *ELK4* and *IRF4* in the Group 4 subgroup. TFs such as *SMAD3*, *FOXD3*, *REST*, *ZIC3*, and *TFAP2C* in the SHH subgroup govern multiple developmental events during embryogenesis including somatic stem cell population maintenance and are involved in the generic transcription pathway (*SMAD3* and *TFAP2C*). Oncogenes *ATF1* and *HOXA11*, and *HNF4G* have been implicated in multiple cancers and are involved in the positive regulation of the RNA metabolic process. In the Group 4 subgroup, TFs such as *ELK4* and *IRF4* have been implicated as oncogenes and are involved in cell differentiation, and, along with *GTF2B*, they are involved in histone modification.

### 3.3. Identification of TYA Gene Signature That Correlates with Overall Survival in MB

**Figure 5:**
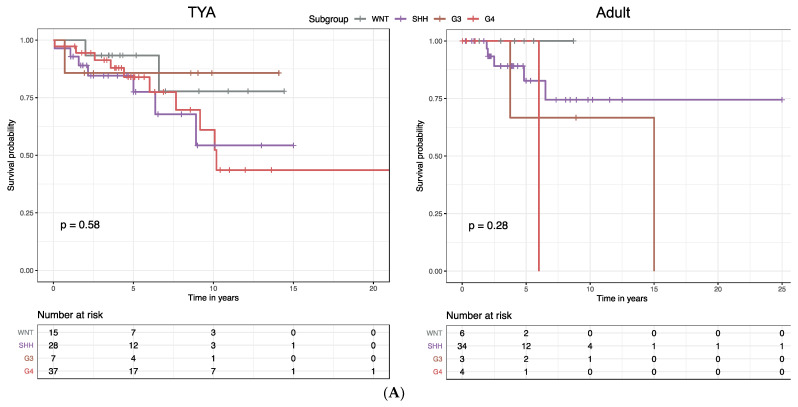

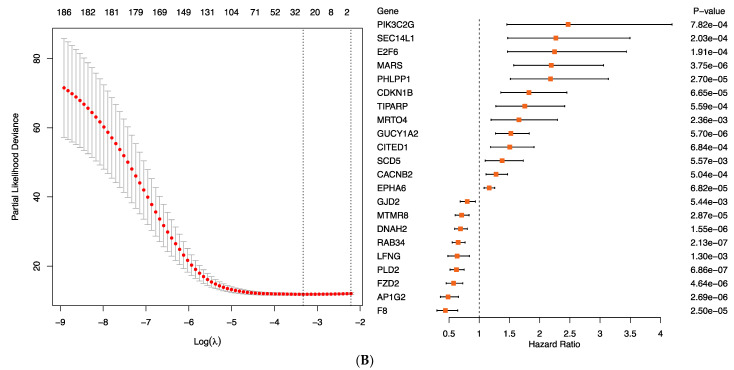
Construction of a prognostic gene expression signature for TYAs with medulloblastoma. (**A**) Kaplan–Meier analysis of overall survival (OS) for medulloblastoma (MB) patients stratified by molecular subgroups in the TYA and adult age groups. (**B**) The risk score model was constructed using the LASSO regression analysis along with 10-fold cross-validation in the entire dataset. The partial likelihood deviance with changing of log (*λ*) was plotted (left). The number corresponded to the point with the smallest cross-verification error was the gene numbers included in the LASSO regression risk model. The hazard ratio (HR) and 95% confidence interval for all 22 genes were calculated by univariate Cox regression analysis.

Molecular subgroups in MB were previously shown to correlate with distinct clinical outcomes: the WNT subgroup was associated with the best survival, the SHH subgroup and Group 4 with intermediate survival; and Group 3 with the poorest survival. When we stratified MB patients by age group, we found significant associations between the overall survival (OS) and molecular subgroups in infants (*P* = 0.026, log-rank test) and children (*P* < 0.0001; Figure S5). In contrast, we found that there were no significant differences in OS by molecular subgroup in the TYA (*P* = 0.58, Figure 5A) and the adult age groups (*P* = 0.28; Figure 5A).

To determine the relationship between gene expression levels and prognosis and to identify new TYA gene signatures, we used gene expression profiles of 729 MB patients with an estimated median OS of four years. Starting with the list of 1002 differentially expressed genes between TYAs and other age groups in the entire dataset, we filtered out genes that showed inconsistent differential expression between TYAs and other age groups in four MB molecular subgroups. The filtering step returned the list of 386 genes, of which 54% showed high expression in TYAs. We next performed univariate Cox regression analysis and identified 186 genes that were significantly associated with patient OS. To identify a smaller set of genes associated with patient survival, a second approach aiming at building sparse prediction models was used. A LASSO penalised Cox regression model was generated, and a predictor consisting of 22 genes was identified (Figure 5B, left and Table S8), of which 13 genes had positive Cox coefficients, indicating that higher expression levels were associated with poor survival outcome (Figure 5B, right). On the other hand, seven genes: *AP1G2*, *DNAH2*, *F8*, *FZD2*, *GJD2*, *LFNG*, *MTMR8*, *PLD2*, and *RAB34* associated with a lower hazard rate, indicating high gene expression levels, were associated with favourable outcomes (Figure 5B, right).

**Figure 6:**
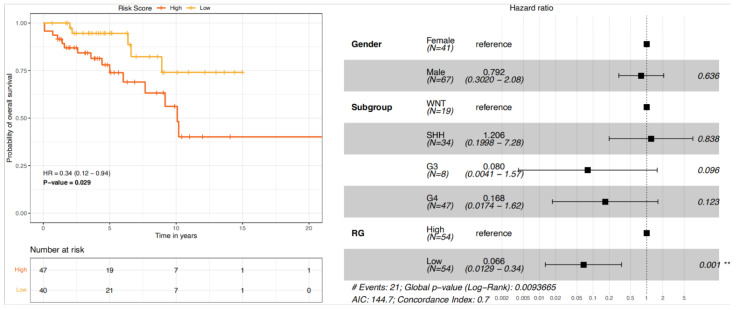
Evaluation of a prognostic gene expression signature for TYAs with medulloblastoma. OS curves of TYA patients with a low- or high-risk of death, according to a combined prognosis score derived from a LASSO analysis. Shown are Kaplan–Meier curves (**left**) of overall survival in TYA patients with low- or high-risk of death. The hazard ratio (HR) and 95% confidence interval of gender, MB subgroups, and risk score were calculated by multivariate regression analysis. The factor in the analysis could be considered as an independent prognostic factor when *p* value was < 0.05 (**right**). ** indicates the *p* value cut-off at 0.001.

We next established the risk scoring system by summarising weighted expression values of the 22 genes. We used the coefficients in the univariate Cox models as weighting factors to compute the risk score. In this way, we calculated the risk score for each patient in the entire cohort. We then selected TYA patients (n = 108) and divided them into high-risk and low-risk groups based on the median risk score, 21.87 (range: 14.62–27.39). Kaplan-Meier survival analysis indicated that high-risk TYA patients were significantly associated with poor survival compared with that of low-risk TYA patients (Figure 6, left). We also used Cox proportional hazards univariate and multivariate modelling to investigate associations of risk-group, molecular subgroups, and gender with OS. In univariate Cox analyses, TYA patients in the low-risk group showed significant association with decreased risk of death (hazard ratio HR = 0.34, *P* = 0.025 from the likelihood ratio test, Table S9). Multivariate Cox analysis further exhibited that the signature retained its independent prognostic value for OS after adjusting for factors such as gender and MB molecular subgroups (Figure 6, right and Table S9). The risk-score based grouping reached univariate C-index (predictive accuracy) of 0.64, which was higher than that of the molecular subgroup (Table S10). A C-index of 1 indicates perfect concordance between predicted risk and actual survival, while a value of 0.5 means random concordance. The multivariate model containing gender, molecular subgroup, and risk-group reached a C-index of 0.70, which remained comparable (C-index = 0.68, Table S10) when we validated the model with 1000 bootstrap internal validation (Table S10).

## 4. Discussion

Integrated molecular profiling has revolutionised the study of MB tumours and showed children with MB are distinct from adults with respect to tumour biology and clinical outcome. TYAs, in the gap between paediatric and adult, represent a vulnerable patient population. Despite the growing interest to address the unmet needs of TYAs, there is a clear paucity of data on TYA patients with MB. We have gathered publicly available data to provide a comprehensive, manually annotated resource cohort of more than 2000 MB tumours with molecular subgroups. Although they are relatively rare in MB, our study accumulated 287 unique TYA cases for interrogation. Using clinical and molecular data from MB patients, we performed comprehensive analyses to uncover TYA-specific molecular landscapes that influence incidence and outcome. This analysis represents the first large-scale analysis of DNA methylation and gene expression in MB, comparing TYAs with other age groups where separate, but parallel, analyses were performed on MB subgroups.

As expected, the SHH and G4 subgroups were found across all age groups in our study. Although Group 4 was the most common in children (49.6%) and TYAs (40.4%), the distribution of MB subgroups varied significantly between these two age groups. The WNT tumours have a higher frequency in TYAs (17.1%) than in infant (0.7%), child (9.4%), and adult (9.3%) populations with MB. Interestingly, the Group 3 tumours in TYAs (9.8%) were more common than in adult (2.5%), but rarer than in infant (31.5%) and children (27%) populations. We found a unique distribution of MB molecular subgroups in TYAs compared to other age groups, which suggests a molecular heterogeneity in the TYA population.

Despite the heterogeneity of MB molecular subgroups, we were able to identify 1002 genes with significant changes in gene expression between TYAs and other age groups. Biological processes related to the pattern specification, cilium organisation, smoothened signalling pathway, axoneme assembly, and wnt signalling pathway were among those enriched for genes that showed high expression in TYAs. Many of these pathways have been previously described as hallmarks of MB [36,37]. To further characterise the observed differentially expressed patterns in TYAs, we examined gene expression changes between TYAs and other age groups in each MB subgroup separately. We decided to employ RRHO to explore the potential overlap in TYA gene expression patterns across MB subgroups. As Group 3 and Group 4 subgroups are known to share some common pathways, it is surprising that we found no overlap in TYA gene expression patterns. In fact, recent medulloblastoma genomic studies show extensive heterogeneity of the Group 3 and Group 4 subtypes and recommend splitting them into as many as eight subtypes, with some subtypes being mixed Group 3 and Group 4 in composition [38]. Indeed, these two subgroups appear as non-WNT/non-SHH in the revised 2016 WHO classification, as the molecular stratification is not conclusive [39]. Age may contribute to the heterogeneity of these subclasses and investigations to account for these differences are now required.

Considering tumour location, histology and frequency of metastasis, the characteristics of the SHH subgroup is different from that of other MB subgroups. When we examined the SHH subgroup, we found an overlap with all three MB subgroups, but the direction of expression changes in TYAs varied with each MB subgroup. The analysis of gene expression patterns in the SHH subgroup showed up-regulation of cilium-related processes in TYAs, which is in line with the observation made with all MB tumours. Besides, genes associated with non-coding RNA processing, mitochondrial gene expression, and smoothened signalling pathways are up regulated, suggest that exclusive sets of genes characterise TYAs with SHH tumours. Indeed, recent integrated analysis of SHH medulloblastomas with RNA-sequencing data demonstrated that each SHH subtype has a unique landscape of non-coding transcripts and biological processes and pathways related to cilium assembly and cell motility and were enriched in the SHHδ subtype [40]. Interestingly, the gene expression analysis of SHH MB tumours revealed that most of the SHHδ subtype was observed not only in adult but also in the TYA population (Figure 2C). Future studies are needed to confirm these findings and to establish concerted gene expression features of TYAs with MB.

Similar to gene expression, we found widespread DNA methylation changes between TYAs and other age groups in the SHH, Group 3, and Group 4 subgroups, but not in the WNT subgroup. Across these three MB subgroups, we found a relatively large number of DMPs with high methylation in TYAs that were primarily located in the CpG islands, which are enriched in gene promoter regions. In contrast, we found that low methylated DMPs in TYAs for the SHH and Group 3 subgroups were primarily located in open-sea regions far from gene promoters. Additionally, DMPs with high methylation levels in TYAs mapped to known enhancer regions at a higher rate in Group 3 and Group 4 subgroups than those with low methylation levels in TYAs. When we focused on identifying possible DNA binding motifs associated with dysregulated methylation in TYAs for each MB subgroup, we found the binding motif for the TF *ZBTB14* in SHH, Group 3 and Group 4 subgroups, significantly enriched in areas associated with DMPs that showed high methylation levels in TYAs. *ZBTB14,* also called *ZFP161,* is a zinc finger protein and belongs to the Kruppel type zinc finger protein family that regulates diverse cellular functions, such as differentiation, transcription, metabolism, apoptosis, and tumorigenesis [41]. It is known to bind to GC-rich site-specific DNA sequences and act as a transcription regulator. The mouse gene homolog, *ZF5*, has been shown to be a repressor of c-Myc and thymidine kinase [42]. It is also interesting that *ZBTB14* activates the ATR signalling pathway under replication stress and maintains genomic stability [43]. Future studies are needed to understand the role of *ZBTB14* in medulloblastoma and its epigenetic regulation with target genes. In addition to *ZBTB14*, we also found several subgroup-specific TF binding sites among both high and low methylated DMPs in TYAs.

MB molecular subgroups have been shown to be associated with disparate prognosis. When we stratified MB patients into different age groups, molecular subgroups showed significant association with the OS only in infants and children, surprisingly, not in the TYA and adult age groups. The lack of prognostic impact of molecular groups warrants the discovery of alternative prognostic markers in TYA medulloblastoma. Our results revealed that the gene signature could successfully classify high-risk and low-risk TYA patients with MB with significant differences in OS. Furthermore, we performed the stratification analysis on TYA patients, and we found that the prognostic power of the gene signature was independent of gender and MB subgroups. Although previous studies have identified multiple prognostic signatures for the general MB population or for the molecular subgroups, to date, there are no such signatures characterising the TYA patient population in MB. Using a machine-learning technique, we identified a 22-gene signature for TYAs based on transcriptional expression profiles from MB patients. Multiple genes in the signature were related to biological functions previously implicated in MB. For example, *FZD2*, a member of the frizzled gene family, encodes a protein that regulates both canonical and non-canonical WNT pathway, which is responsible for embryonic development and is deregulated in MB [44]. Moreover, the aberrant activation of the canonical WNT pathway characterises a subset of MBs, the WNT subgroup [9,36,45]. Similarly, *DNAH2*, a member of an axonemal dynein complex, is involved in motile cilia that plays a vital role in human development and homeostasis [46] and is among the primary cilium-related genes that are enriched in both SHH and Group 3 tumours [11]. Another gene, *LFNG*, encodes for a glycosylating enzyme (O-fucosylpeptide 3-beta-N-acetylglucosaminyltransferase) regulating NOTCH signalling pathway that plays an important role in cell fate assignation and pattern formation during development, cell proliferation, and cell survival, and is previously implicated in MB biology [45]. Besides, genes in the signature are enriched in diverse signalling pathways including, RhoA signalling pathway (*CDKN1B* and *PLD2*), phosphoinositides metabolism (*MTMR8* and *PIK3C2G*), and gap junction (*GJD2* and *GUCY1A2*). The roles, as well as the underlying mechanisms of these genes in the TYA population of MB patients, warrant further investigation.

These results are compelling and represent the first age group-based analysis, particularly the TYAs with MB data. However, we recognise several limitations of this study. First, the published cohorts collectively contain a modest number of TYA patients. We may not have accounted for all molecular heterogeneity with each MB subgroup due, in part, to the availability of molecular data. Second, the retrospective design of the published cohorts, i.e., no access to clinical parameters, such as the extent of resection, treatment, recurrence, and known prognostic factors, may have introduced potential confounding in our model, which we have not been able to control for. Finally, detecting DMPs using 450K data has limitations due to its reduced CpG representation as compared with the whole genome bisulfite sequencing. Future work includes larger sample sizes in TYA patients, validation of our findings in an independent set of MB patients and assesses the impact of molecular subgroups on epigenetic and transcriptional differences. Nonetheless, this work has identified many avenues to pursue to better understand TYA-specific differences in MB biology.

## 5. Conclusions

In summary, our study has delineated the molecular landscape of TYAs with medulloblastoma. It revealed detailed TYA-specific epigenetic and transcriptomic characteristics. Our work also provides insights into the development of the gene expression-based prognostic signature of TYA medulloblastoma. The model stratified TYA patients into two groups, whereby decreased survival was associated with the high-risk group of TYAs. The molecular characteristics of TYAs with medulloblastoma and TYA gene signatures warrant further exploration in future studies. 

## Data Availability

All data utilised in this study are publicly available. See ‘Methods’ for data sources.

## Supplementary Materials

The following supporting information can be downloaded at: https://www.mdpi.com/article/10.3390/cancers14010251/s1, Supplementary Figure S1: An overview of the analyses used in this study, Supplementary Figure S2: Distribution of medulloblastoma molecular subgroups across studies, Supplementary Figure S3: Distribution of medulloblastoma patients by age and molecular subgroups, Supplementary Figure S4: Relationship between age and molecular subgroups in medulloblastoma patients, Supplementary Figure S5: Survival analysis by age groups, Table S1: List of differentially expressed genes between TYAs and other age groups in the entire dataset, Table S2: List of significant gene ontology (GO) biological processes enriched in differentially expressed genes between TYAs and other age groups in the entire dataset, Table S3: List of significant gene ontology (GO) terms enriched in differentially expressed genes between TYAs and other age groups in the SHH tumours by the Gene Set Enrichment Analysis (GSEA), Table S4: List of differentially methylated probes (DMPs) between TYAs and other age groups that are common across three MB subgroups: SHH, Group 3, and Group 4, Table S5: List of differentially methylated probes (DMPs) between TYAs and other age groups that showed at least 20% methylation differences in the SHH subgroup, Table S6: List of significant gene ontology (GO) biological processes enriched in differentially methylated probes (DMPs) between TYAs and other age groups in the SHH tumours and showed high methylation levels in TYAs, Table S7: List of significant gene ontology (GO) biological processes enriched in differentially methylated probes (DMPs) between TYAs and other age groups in the SHH tumours and showed low methylation levels in TYAs, Table S8: Prognostic gene signature, Table S9: Univariate and multivariate Cox proportional hazard ratios for gender, molecular subgroup, and risk score for TYAs with medulloblastoma, Table S10: C-index for univariate and multivariate Cox proportional models for TYAs with medulloblastoma.
