# Supplementary material for: The Molecular Landscape of Medulloblastoma in Teenagers and Young Adults
Source: Cancers (Basel). 2022 Jan 5;14(1):251. doi: 10.3390/cancers14010251 (PMC8750554; doi:10.3390/cancers14010251)
Supplement: Supplementary file 1 [file cancers-14-00251-s001.zip › cancers-1539876-supplementary.pdf]

List and captions for Supplementary Figures, Tables and Materials, including Figures.

The molecular landscape of teenagers and young adults medulloblastoma

Omar Mohammed[1], Maria Estevez Cebrero[1], Omar Ahmad[1], Andrew Peet [2,3], Richard Grundy[1], Anbarasu Lourdusamy[1]

[1]Children's Brain Tumour Research Centre, School of Medicine, University of Nottingham, Nottingham, UK

[2]Institute of Cancer and Genomic Sciences, University of Birmingham, UK

[3]Birmingham Women's and Children's Hospital NHS Foundation Trust, Birmingham, UK

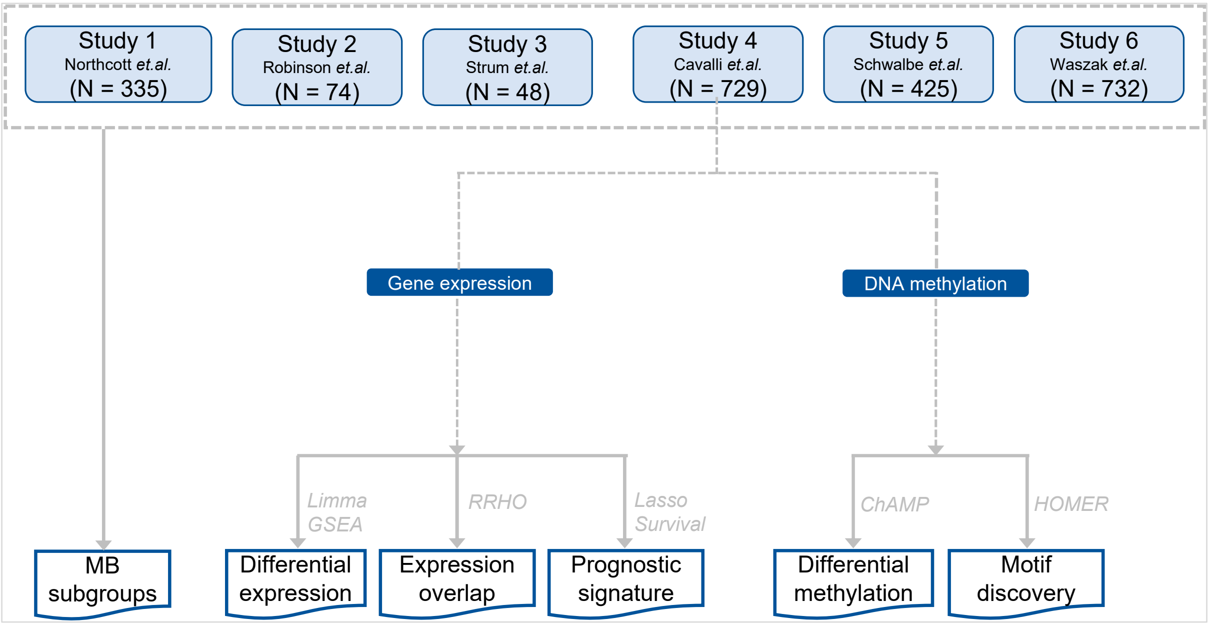

Figure S1. An overview of the analyses used in this study. Briefly, age and molecular subgroup data were collated from six different studies and used for medulloblastoma (MB) subgroup analysis. Microarray gene expression and DNA methylation data was obtained from 729 MB patients and used for transcriptome and epigenetic characterisation of teenagers and young adults in MB.

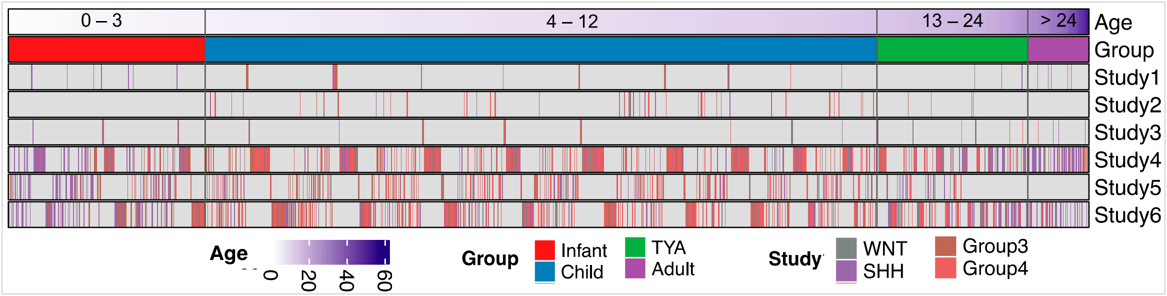

Figure S2. Distribution of medulloblastoma molecular subgroups across studies. Four molecular subgroups: Wingless activated (WNT), Sonic Hedge- 66 hog (SHH), Group 3 and Group 4 from six different published studies, four different age groups: Infants (< 3 years), Children (4 – 12 years), Teenagers and young adults (TYA, 13 – 24 years), and adults (> 24 years), and age at diagnosis (in years) in ascending order are shown in coloured tracks.

A

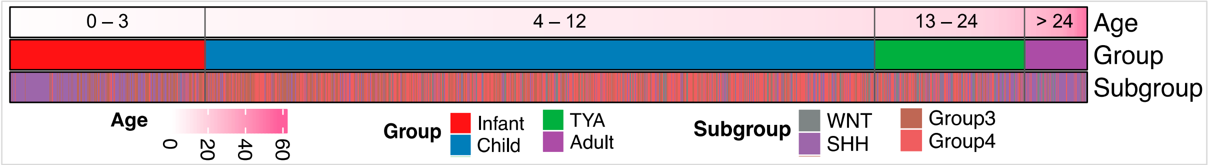

B

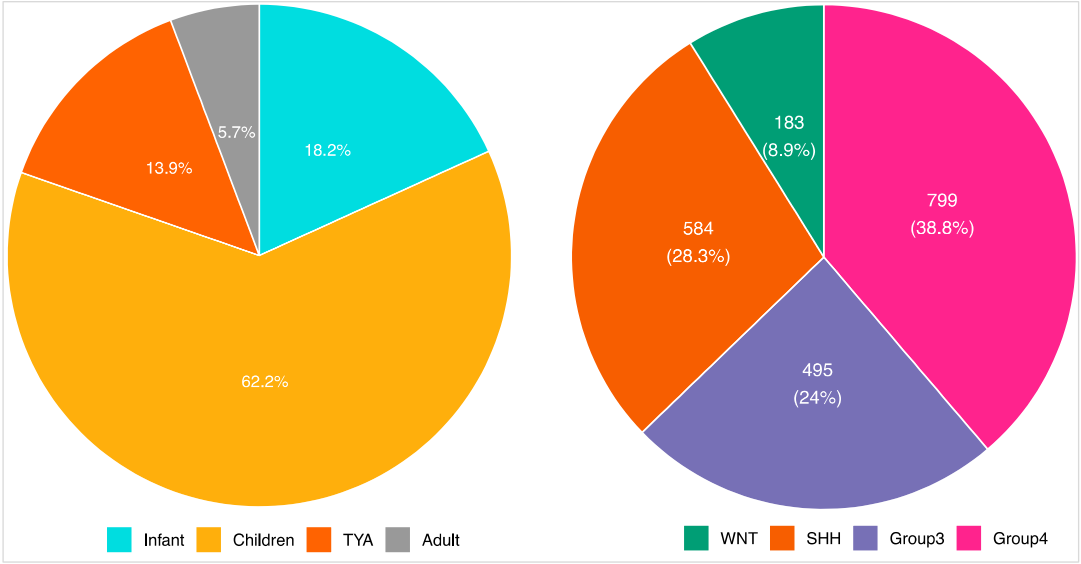

Figure S3. Distribution of medulloblastoma patients by age and molecular subgroups. (A) Stratification of medulloblastoma patients by different age group. Four molecular subgroups: Wingless activated (WNT), Sonic Hedge- 66 hog (SHH), Group 3 and Group 4 of 2061 medulloblastoma patients stratified by four different age groups: Infants (< 3 years), Children (4 – 12 years), Teenagers and young adults (TYA, 13 – 24 years), and adults (> 24 years), and age at diagnosis (in years) in ascending order are shown in coloured tracks. **(B)** Two pie charts describing the distribution of age groups and molecular subgroups for medulloblastomas: 1) Infant (<3 years), 2) Children (4-12 years), 3) Teenager and young adult, TYA (13-24 years), and 4) Adult (>24 years). Molecular subgroup and age data were collected from published studies that resulted in a total of 2,061 medulloblastoma patients.

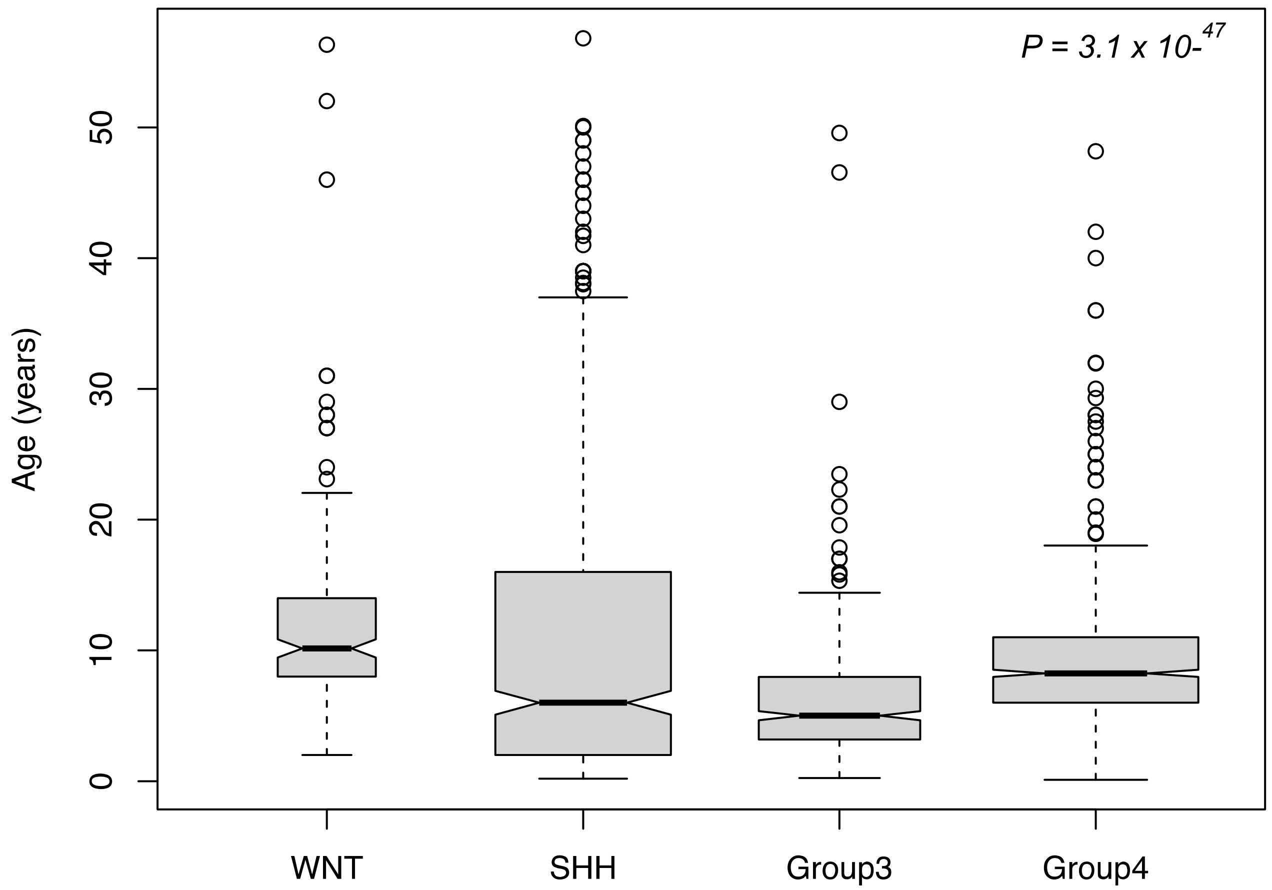

Figure S4. Relationship between age and molecular subgroups in medulloblastoma patients. Box plot describing the distribution of age across four molecular subgroups for medulloblastomas: WNT, SHH, Group 3 and Group 4. Kruskal-Wallis rank-sum test was used to assess the association between age and molecular subgroups.

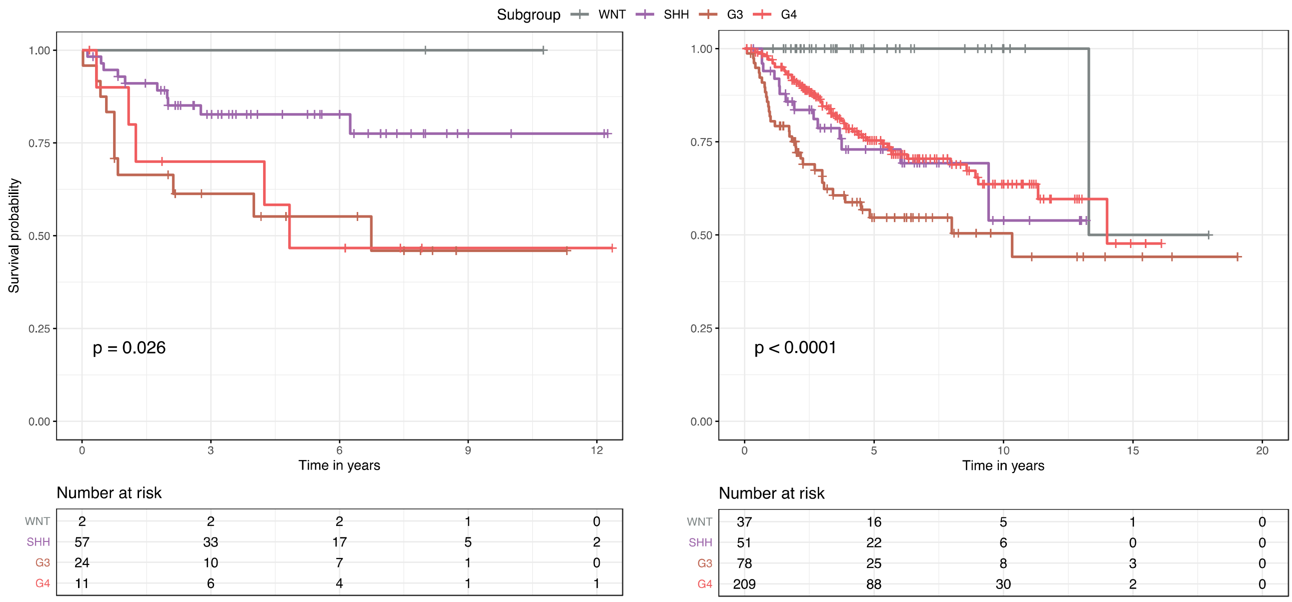

Figure S5. Survival analysis by age groups. The Kaplan-Meier survival curves of four molecular subgroups for medulloblastomas: WNT, SHH, Group 3 and Group 4 in Infants and Children patients.

Table S1: List of differentially expressed genes between TYA and other age groups

Gene	logFC	AveExpr	t	P_Value	FDR
RUNX3	0.53	5.84	7.27	9.17E-13	1.68E-08
SERPIND1	0.56	4.96	6.35	3.69E-10	3.38E-06
C9orf3	0.27	7.33	6.09	1.79E-09	1.09E-05
LINC00476	0.23	6.10	5.73	1.51E-08	5.65E-05
RPL39L	-0.50	4.60	-5.72	1.54E-08	5.65E-05
EMID1	0.53	6.89	5.67	2.08E-08	6.36E-05
GJD4	0.23	5.17	5.54	4.25E-08	1.07E-04
XYLT2	0.24	6.75	5.52	4.67E-08	1.07E-04
TPPP3	0.76	7.76	5.48	5.79E-08	1.14E-04
PRSS23	0.34	5.51	5.47	6.22E-08	1.14E-04
EIF2AK3	-0.27	7.41	-5.44	7.29E-08	1.21E-04
DNAH9	0.25	5.04	5.42	8.25E-08	1.21E-04
GPHN	-0.43	9.51	-5.38	9.82E-08	1.21E-04
DNAH2	0.67	6.09	5.38	9.83E-08	1.21E-04
ACOX2	0.40	6.05	5.38	9.91E-08	1.21E-04
C3orf22	0.46	5.26	5.36	1.13E-07	1.28E-04
PPP1R1B	0.49	6.80	5.35	1.21E-07	1.28E-04
PIFO	0.46	4.62	5.34	1.25E-07	1.28E-04
FBP2	0.31	3.92	5.29	1.65E-07	1.52E-04
FRY	-0.61	8.61	-5.29	1.66E-07	1.52E-04
WNK4	0.32	4.70	5.26	1.88E-07	1.64E-04
THTPA	0.17	7.18	5.21	2.42E-07	2.00E-04
FAM69A	-0.53	7.16	-5.21	2.51E-07	2.00E-04
HOXA6	1.05	6.22	5.19	2.76E-07	2.11E-04
FSTL5	-1.26	7.63	-5.13	3.72E-07	2.69E-04
HOXA5	0.93	5.81	5.12	3.94E-07	2.69E-04
OLFM2	-0.63	9.48	-5.12	3.96E-07	2.69E-04
GPR78	0.46	5.36	5.10	4.42E-07	2.90E-04
IQCA1	0.67	5.77	5.08	4.87E-07	3.08E-04
FIGNL1	-0.34	6.12	-5.05	5.45E-07	3.33E-04
TRIM59	-0.46	5.24	-5.04	5.93E-07	3.51E-04
NMRAL1	0.34	6.87	5.00	7.19E-07	4.12E-04
HOXA7	0.94	7.29	4.99	7.43E-07	4.13E-04
MR1	0.35	5.82	4.98	7.87E-07	4.24E-04
SLC17A5	-0.22	7.48	-4.95	9.19E-07	4.82E-04
GRK6	0.17	7.47	4.93	1.02E-06	5.19E-04
MDGA2	-0.62	5.86	-4.92	1.07E-06	5.28E-04
SLCO1A2	0.60	4.65	4.91	1.11E-06	5.34E-04
SLC13A5	0.30	5.66	4.91	1.14E-06	5.34E-04
RABGAP1L	-0.34	7.78	-4.87	1.36E-06	6.25E-04
C1orf228	0.29	6.30	4.86	1.46E-06	6.52E-04
ADGRD2	0.34	5.93	4.84	1.57E-06	6.85E-04
UROC1	0.29	4.57	4.82	1.76E-06	7.50E-04
C1QTNF4	-0.41	6.89	-4.81	1.85E-06	7.69E-04
C9orf40	-0.15	7.02	-4.79	2.00E-06	8.14E-04
MAGEL2	0.34	5.27	4.78	2.14E-06	8.51E-04
FADS3	0.30	6.97	4.77	2.20E-06	8.59E-04
APC	-0.29	8.35	-4.76	2.35E-06	8.97E-04
MAP3K14-AS1	0.20	5.44	4.75	2.41E-06	9.01E-04

CNTN1	-0.87	9.45	-4.74	2.53E-06	9.28E-04
LPXN	0.19	7.44	4.71	2.95E-06	1.06E-03
SIM2	0.35	5.08	4.70	3.04E-06	1.06E-03
CADM2	-0.74	7.26	-4.70	3.06E-06	1.06E-03
MCCC2	0.17	8.01	4.69	3.21E-06	1.06E-03
PTTG2	0.27	4.71	4.69	3.21E-06	1.06E-03
SEMA3B	0.28	5.99	4.69	3.24E-06	1.06E-03
PPM1B	-0.18	8.52	-4.68	3.42E-06	1.08E-03
LINC01296	0.54	6.82	4.68	3.42E-06	1.08E-03
ADD3	-0.25	7.99	-4.67	3.52E-06	1.09E-03
PAX3	0.88	5.36	4.67	3.62E-06	1.09E-03
ARHGEF19	0.20	6.13	4.67	3.64E-06	1.09E-03
TACR2	0.15	5.22	4.66	3.70E-06	1.10E-03
HOXA4	0.76	4.83	4.66	3.80E-06	1.11E-03
BOD1	0.17	8.96	4.65	4.00E-06	1.12E-03
PPARG	-0.33	5.46	-4.65	4.01E-06	1.12E-03
KLF9	-0.29	6.91	-4.65	4.02E-06	1.12E-03
HEMK1	0.20	7.20	4.61	4.74E-06	1.29E-03
SLITRK3	-0.86	6.34	-4.61	4.77E-06	1.29E-03
MRVI1	0.35	5.66	4.59	5.24E-06	1.39E-03
FAM71D	-0.22	4.45	-4.58	5.54E-06	1.45E-03
LEF1	0.73	8.08	4.57	5.76E-06	1.49E-03
TMEM74	-0.37	7.00	-4.55	6.28E-06	1.60E-03
ACP6	0.28	6.87	4.53	6.76E-06	1.67E-03
GPR19	-0.34	6.99	-4.53	6.80E-06	1.67E-03
TOX3	0.57	8.76	4.53	6.84E-06	1.67E-03
DNAH10	0.28	5.40	4.52	7.26E-06	1.75E-03
PLCB2	0.25	6.30	4.50	7.73E-06	1.83E-03
FOXF1	0.23	5.25	4.50	7.79E-06	1.83E-03
MYCBPAP	0.29	5.25	4.50	7.94E-06	1.84E-03
AKAP5	-0.32	5.08	-4.49	8.16E-06	1.85E-03
LEO1	-0.18	7.55	-4.49	8.17E-06	1.85E-03
CPZ	0.40	5.71	4.49	8.45E-06	1.89E-03
BICD1	-0.25	10.24	-4.48	8.61E-06	1.90E-03
PHLPP1	-0.20	7.57	-4.48	8.86E-06	1.92E-03
TAOK3	-0.33	7.97	-4.47	9.02E-06	1.92E-03
PNKP	0.15	7.68	4.46	9.28E-06	1.92E-03
TBC1D8	-0.45	7.50	-4.46	9.29E-06	1.92E-03
OVOS2	0.24	5.24	4.46	9.33E-06	1.92E-03
KIFC3	-0.25	6.05	-4.46	9.38E-06	1.92E-03
ZSCAN12	-0.44	6.50	-4.46	9.42E-06	1.92E-03
PLPP5	0.25	8.63	4.45	9.77E-06	1.97E-03
FZD2	0.31	7.07	4.45	9.93E-06	1.98E-03
CFAP54	0.46	5.89	4.45	1.01E-05	1.99E-03
DUOX2	0.24	4.78	4.44	1.03E-05	2.00E-03
PRDM16	0.18	4.84	4.44	1.03E-05	2.00E-03
TBC1D14	0.17	8.69	4.44	1.05E-05	2.00E-03
ENO4	0.37	6.09	4.43	1.08E-05	2.04E-03
MOGAT1	0.16	3.58	4.42	1.12E-05	2.04E-03
COA4	0.15	6.91	4.42	1.12E-05	2.04E-03
CECR6	-0.20	7.23	-4.42	1.12E-05	2.04E-03
CACHD1	0.44	9.21	4.42	1.12E-05	2.04E-03
KCTD8	-0.31	6.12	-4.42	1.16E-05	2.08E-03

EFNA4	0.49	5.04	4.41	1.18E-05	2.10E-03
GLB1L	0.16	6.41	4.40	1.24E-05	2.18E-03
ENKUR	0.37	4.94	4.40	1.26E-05	2.18E-03
LCNL1	0.35	6.13	4.40	1.26E-05	2.18E-03
ING3	-0.17	8.53	-4.40	1.27E-05	2.18E-03
REL	-0.20	7.77	-4.39	1.29E-05	2.19E-03
WDR6	0.16	9.56	4.39	1.30E-05	2.19E-03
CMIP	0.38	9.30	4.37	1.41E-05	2.36E-03
LFNG	0.28	5.90	4.36	1.47E-05	2.43E-03
GABARAPL1	-0.48	8.11	-4.35	1.54E-05	2.52E-03
CFAP53	0.25	5.18	4.35	1.55E-05	2.52E-03
CYP3A5	0.50	5.19	4.34	1.61E-05	2.53E-03
LETMD1	0.13	9.14	4.34	1.61E-05	2.53E-03
ATP2C1	-0.20	8.54	-4.34	1.62E-05	2.53E-03
TRIM41	0.13	6.61	4.34	1.64E-05	2.53E-03
LRRC53	0.30	3.62	4.34	1.64E-05	2.53E-03
PLEKHO2	0.29	7.19	4.34	1.65E-05	2.53E-03
PINK1-AS	0.18	6.26	4.34	1.65E-05	2.53E-03
EYS	-1.28	7.54	-4.33	1.73E-05	2.62E-03
POLR3H	0.14	7.62	4.32	1.75E-05	2.62E-03
S100A16	0.42	6.97	4.32	1.75E-05	2.62E-03
ATP2A3	0.54	6.26	4.32	1.80E-05	2.63E-03
HIST1H4L	-0.43	8.76	-4.32	1.80E-05	2.63E-03
HMG20B	0.31	8.41	4.32	1.81E-05	2.63E-03
KIAA1191	0.14	9.79	4.31	1.88E-05	2.71E-03
PAQR5	0.15	4.52	4.28	2.10E-05	3.00E-03
ZC3H12A	0.15	6.18	4.28	2.11E-05	3.00E-03
HOXA3	0.52	5.73	4.28	2.13E-05	3.00E-03
SPG21	0.14	9.57	4.28	2.14E-05	3.00E-03
TMEM27	0.20	5.02	4.27	2.25E-05	3.13E-03
FANK1	0.24	5.20	4.26	2.36E-05	3.26E-03
ACSL3	-0.25	8.41	-4.25	2.39E-05	3.28E-03
PEX10	0.14	7.48	4.25	2.42E-05	3.28E-03
CLDN11	0.60	7.51	4.25	2.46E-05	3.32E-03
DIO3	0.21	5.15	4.24	2.51E-05	3.34E-03
SYN2	-0.53	7.83	-4.24	2.53E-05	3.34E-03
APOBEC3C	0.32	6.26	4.24	2.55E-05	3.34E-03
APOBEC3G	0.19	4.27	4.24	2.55E-05	3.34E-03
HAND2-AS1	0.61	4.79	4.23	2.59E-05	3.37E-03
RASAL2	-0.27	8.54	-4.23	2.67E-05	3.45E-03
SPATA24	0.14	5.22	4.22	2.75E-05	3.53E-03
TDRD9	-0.69	5.37	-4.22	2.78E-05	3.53E-03
MACROD2	-0.39	6.67	-4.21	2.86E-05	3.60E-03
GUCY1A2	-0.34	6.71	-4.21	2.88E-05	3.60E-03
SELENBP1	0.40	7.03	4.21	2.88E-05	3.60E-03
LDLRAD4	-0.47	7.21	-4.20	2.94E-05	3.65E-03
ITGA6	0.49	7.18	4.19	3.17E-05	3.90E-03
S100A6	0.19	5.77	4.18	3.26E-05	3.99E-03
ZNF154	-0.61	7.76	-4.17	3.36E-05	4.08E-03
HMGN5	-0.38	6.81	-4.17	3.44E-05	4.15E-03
SFRP5	0.58	5.82	4.17	3.47E-05	4.16E-03
GRHL1	-0.36	6.46	-4.16	3.51E-05	4.17E-03
SCD5	-0.31	8.24	-4.16	3.54E-05	4.17E-03

MED28	0.15	8.87	4.16	3.55E-05	4.17E-03
ATP6V0E1	0.17	9.87	4.16	3.58E-05	4.18E-03
TEKT1	0.29	4.75	4.16	3.63E-05	4.22E-03
PACRGL	0.16	9.03	4.15	3.72E-05	4.29E-03
LIN28B	-0.53	5.06	-4.15	3.78E-05	4.34E-03
DNAH6	0.31	5.79	4.14	3.82E-05	4.35E-03
HIST1H3J	-0.41	6.56	-4.14	3.86E-05	4.37E-03
FGFR2	0.47	6.38	4.14	3.90E-05	4.38E-03
EDEM3	-0.16	8.19	-4.13	4.07E-05	4.55E-03
KSR2	0.33	5.91	4.12	4.15E-05	4.62E-03
MAEA	0.13	8.54	4.12	4.23E-05	4.67E-03
WNT11	0.34	5.39	4.10	4.52E-05	4.96E-03
DIEXF	0.19	7.53	4.10	4.55E-05	4.97E-03
SLC6A20	0.28	4.97	4.10	4.63E-05	5.02E-03
KCNC2	-0.73	6.91	-4.10	4.66E-05	5.03E-03
BTC	-0.20	5.12	-4.09	4.82E-05	5.17E-03
PRSS12	-0.72	7.50	-4.08	4.92E-05	5.20E-03
PPIE	0.18	7.07	4.08	4.92E-05	5.20E-03
PRRG1	-0.26	6.37	-4.08	4.94E-05	5.20E-03
HERC3	-0.38	8.66	-4.08	4.98E-05	5.21E-03
ARNTL	-0.39	6.23	-4.08	5.00E-05	5.21E-03
PCLO	-0.56	7.66	-4.08	5.09E-05	5.26E-03
SLC35F3	-0.55	7.24	-4.08	5.10E-05	5.26E-03
SERAC1	-0.17	7.25	-4.07	5.19E-05	5.31E-03
ZDHHC1	0.19	7.07	4.07	5.23E-05	5.31E-03
APP	0.37	11.36	4.07	5.24E-05	5.31E-03
DNAJC5	-0.15	7.65	-4.07	5.32E-05	5.36E-03
DISP1	0.17	7.21	4.06	5.36E-05	5.37E-03
NEK3	0.28	7.18	4.05	5.70E-05	5.68E-03
RARRES1	0.23	5.03	4.05	5.78E-05	5.73E-03
MAATS1	0.32	5.50	4.04	5.95E-05	5.87E-03
FBXW8	0.14	8.40	4.04	5.99E-05	5.87E-03
DMD	-0.54	7.15	-4.03	6.17E-05	6.02E-03
TTC28	0.18	9.37	4.03	6.28E-05	6.10E-03
CALHM2	0.32	7.05	4.02	6.36E-05	6.14E-03
DCBLD1	-0.29	6.65	-4.02	6.46E-05	6.18E-03
ATP13A2	-0.20	8.17	-4.02	6.50E-05	6.18E-03
ZNF702P	-0.30	5.74	-4.02	6.52E-05	6.18E-03
SCP2	-0.19	8.93	-4.02	6.56E-05	6.18E-03
CCDC30	0.25	6.80	4.01	6.57E-05	6.18E-03
PTPRK	0.58	8.33	4.01	6.64E-05	6.21E-03
YWHAH	-0.19	9.46	-4.01	6.73E-05	6.26E-03
MRPS5	-0.10	8.79	-4.00	6.91E-05	6.40E-03
IPCEF1	-0.53	7.26	-4.00	7.09E-05	6.53E-03
DUOXA1	0.18	6.39	3.99	7.16E-05	6.56E-03
SYNJ1	-0.26	7.82	-3.99	7.31E-05	6.66E-03
GLCCI1	-0.36	8.84	-3.99	7.34E-05	6.66E-03
YWHAB	-0.22	9.95	-3.99	7.42E-05	6.70E-03
CCDC17	0.15	6.18	3.98	7.54E-05	6.78E-03
CDH6	-0.48	7.34	-3.98	7.62E-05	6.81E-03
SLC4A10	-0.62	7.18	-3.97	7.94E-05	7.06E-03
SRRM4	-0.27	9.32	-3.96	8.06E-05	7.10E-03
DLEC1	0.14	4.85	3.96	8.09E-05	7.10E-03

LIN7A	-0.44	6.42	-3.96	8.10E-05	7.10E-03
TTC21A	0.22	6.11	3.96	8.24E-05	7.19E-03
SFXN3	-0.40	7.73	-3.95	8.41E-05	7.29E-03
ROCK2	-0.20	9.14	-3.95	8.44E-05	7.29E-03
TCTN1	0.17	7.43	3.95	8.46E-05	7.29E-03
KLHL31	0.12	5.06	3.95	8.56E-05	7.33E-03
FAM78A	-0.14	5.28	-3.94	8.86E-05	7.56E-03
ERBB2	0.19	6.91	3.94	8.97E-05	7.61E-03
MFSD11	-0.24	7.92	-3.94	9.05E-05	7.64E-03
C2orf81	0.18	6.04	3.94	9.08E-05	7.64E-03
DKK2	0.75	5.22	3.93	9.15E-05	7.66E-03
TKFC	0.17	7.57	3.93	9.22E-05	7.68E-03
HCAR1	0.67	4.76	3.93	9.30E-05	7.71E-03
RFX5	0.15	8.04	3.92	9.56E-05	7.90E-03
LRP11	-0.13	6.30	-3.92	9.65E-05	7.94E-03
MIR181B1	-0.47	5.93	-3.92	9.72E-05	7.96E-03
PRNP	-0.38	10.49	-3.92	9.84E-05	8.02E-03
MORN5	0.14	4.41	3.91	9.99E-05	8.06E-03
KANSL2	-0.13	7.74	-3.91	9.99E-05	8.06E-03
KAT2A	0.18	8.75	3.91	1.00E-04	8.06E-03
SGK2	0.24	5.04	3.91	1.02E-04	8.14E-03
EARS2	0.11	6.82	3.91	1.03E-04	8.14E-03
PCYT1B	0.41	6.53	3.90	1.03E-04	8.14E-03
CDKN1B	-0.21	8.84	-3.90	1.03E-04	8.14E-03
NHP2	0.17	7.30	3.90	1.04E-04	8.14E-03
CFAP52	0.12	4.41	3.90	1.04E-04	8.14E-03
HCAR3	0.50	3.90	3.90	1.04E-04	8.14E-03
B3GALT2	-0.50	6.49	-3.90	1.05E-04	8.15E-03
ZBTB48	0.09	6.02	3.90	1.06E-04	8.19E-03
RNVU1-3	0.39	5.22	3.90	1.06E-04	8.20E-03
C11orf70	0.57	4.48	3.89	1.09E-04	8.38E-03
CAMSAP3	-0.45	7.67	-3.89	1.10E-04	8.43E-03
HDAC4	0.20	7.23	3.89	1.11E-04	8.44E-03
DKKL1	0.20	5.46	3.88	1.12E-04	8.45E-03
ARPP21	-0.62	6.02	-3.88	1.12E-04	8.45E-03
LRRTM4	-0.57	6.78	-3.88	1.13E-04	8.45E-03
CDC42EP4	0.25	7.03	3.88	1.13E-04	8.45E-03
POMGNT1	0.13	8.20	3.88	1.13E-04	8.45E-03
SPON1	-0.52	8.40	-3.88	1.14E-04	8.46E-03
MAGI2	-0.36	8.21	-3.88	1.14E-04	8.46E-03
ADGRB3	-0.50	7.46	-3.87	1.16E-04	8.56E-03
UPP2	-0.20	5.21	-3.87	1.17E-04	8.56E-03
EML5	-0.41	7.44	-3.87	1.17E-04	8.56E-03
TNR	-0.61	6.23	-3.87	1.18E-04	8.56E-03
PAPOLG	-0.13	9.25	-3.87	1.19E-04	8.56E-03
FOXN2	-0.19	7.88	-3.87	1.19E-04	8.56E-03
GPR179	0.16	5.10	3.86	1.22E-04	8.80E-03
ALPL	0.39	6.41	3.86	1.24E-04	8.86E-03
SLC45A2	0.24	5.28	3.86	1.24E-04	8.86E-03
SPATA6L	0.27	5.99	3.86	1.25E-04	8.88E-03
TNFRSF11B	0.40	4.15	3.85	1.27E-04	9.01E-03
COPA	0.12	10.71	3.85	1.31E-04	9.19E-03
EIF5A2	0.32	6.13	3.85	1.31E-04	9.19E-03

WIF1	0.88	5.14	3.84	1.36E-04	9.53E-03
SLC25A23	-0.17	6.90	-3.83	1.37E-04	9.56E-03
RAVER2	0.32	7.94	3.83	1.39E-04	9.60E-03
HMGN4	0.14	8.95	3.83	1.39E-04	9.60E-03
GPRC5C	0.23	5.86	3.83	1.39E-04	9.60E-03
TNIP3	-0.13	4.06	-3.83	1.40E-04	9.62E-03
ZNF804A	-0.87	7.89	-3.83	1.41E-04	9.62E-03
PAPPA2	-0.70	6.66	-3.83	1.41E-04	9.63E-03
LOC375196	-0.11	5.34	-3.82	1.43E-04	9.66E-03
GABARAPL3	-0.15	4.90	-3.82	1.43E-04	9.66E-03
NOCT	-0.18	6.61	-3.82	1.45E-04	9.73E-03
USP12	-0.13	7.33	-3.82	1.45E-04	9.73E-03
MARS	-0.18	9.08	-3.82	1.45E-04	9.73E-03
FZD1	-0.44	7.50	-3.82	1.47E-04	9.80E-03
AP1S2	-0.26	6.15	-3.81	1.48E-04	9.83E-03
HSPA4L	-0.26	9.18	-3.81	1.49E-04	9.87E-03
OGFRL1	-0.32	8.37	-3.81	1.51E-04	9.94E-03
PRPS2	0.30	7.79	3.81	1.52E-04	1.00E-02
HCN1	-0.66	7.70	-3.81	1.53E-04	1.00E-02
LYST	-0.21	8.86	-3.80	1.56E-04	1.02E-02
AKR7A3	0.13	7.68	3.80	1.58E-04	1.02E-02
RILPL2	0.25	6.79	3.80	1.58E-04	1.02E-02
PHTF2	-0.14	8.40	-3.80	1.59E-04	1.02E-02
PTPRZ1	-0.77	8.26	-3.80	1.59E-04	1.02E-02
HOXA2	0.72	5.45	3.80	1.60E-04	1.02E-02
YAP1	0.40	7.33	3.80	1.60E-04	1.02E-02
NIPAL1	-0.23	5.08	-3.79	1.60E-04	1.02E-02
Sep-08	-0.24	8.46	-3.79	1.61E-04	1.02E-02
HIST1H4F	-0.44	6.57	-3.79	1.62E-04	1.02E-02
DUSP8	-0.25	8.24	-3.79	1.63E-04	1.03E-02
COL27A1	-0.53	7.78	-3.79	1.64E-04	1.03E-02
NADSYN1	0.15	7.07	3.79	1.64E-04	1.03E-02
ARMT1	-0.18	6.95	-3.79	1.65E-04	1.03E-02
NKAPL	-0.33	5.18	-3.78	1.67E-04	1.04E-02
RAB36	0.31	6.67	3.78	1.68E-04	1.04E-02
NBPF3	0.15	6.93	3.78	1.68E-04	1.04E-02
RAB34	0.34	7.25	3.78	1.70E-04	1.04E-02
TTC7A	0.13	6.19	3.78	1.70E-04	1.04E-02
ACTC1	0.76	7.02	3.78	1.70E-04	1.04E-02
KIAA0141	0.14	8.02	3.77	1.74E-04	1.06E-02
KREMEN1	0.35	6.98	3.77	1.75E-04	1.06E-02
FAM207A	0.18	7.28	3.77	1.78E-04	1.07E-02
PARN	0.11	8.73	3.77	1.79E-04	1.07E-02
CSRNP3	-0.46	8.49	-3.77	1.79E-04	1.07E-02
C20orf27	0.18	5.70	3.77	1.79E-04	1.07E-02
SPACA9	0.15	5.94	3.77	1.79E-04	1.07E-02
TMEM43	0.13	9.21	3.76	1.80E-04	1.07E-02
RSPO4	0.21	5.33	3.76	1.80E-04	1.07E-02
RNF135	0.17	6.98	3.76	1.81E-04	1.07E-02
ACSF2	0.29	4.62	3.76	1.81E-04	1.07E-02
RBP1	0.47	7.56	3.76	1.82E-04	1.07E-02
MEMO1	-0.11	7.32	-3.76	1.85E-04	1.08E-02
PHYKPL	0.16	7.53	3.75	1.89E-04	1.10E-02

PDZRN3	0.56	6.44	3.75	1.89E-04	1.10E-02
RIOX1	-0.20	6.93	-3.75	1.89E-04	1.10E-02
DCAF8	0.10	7.84	3.75	1.90E-04	1.10E-02
IGF2BP3	-0.73	6.48	-3.75	1.91E-04	1.10E-02
ATP7B	-0.20	7.01	-3.75	1.92E-04	1.10E-02
CCND1	0.37	7.06	3.75	1.93E-04	1.10E-02
PCDHB9	0.35	10.20	3.75	1.93E-04	1.10E-02
TCERG1L	0.44	6.83	3.74	1.98E-04	1.12E-02
BTBD10	-0.16	9.15	-3.74	1.98E-04	1.12E-02
NEO1	-0.23	9.66	-3.74	2.00E-04	1.13E-02
ERLIN2	0.16	6.56	3.74	2.02E-04	1.14E-02
CLIC3	0.11	5.06	3.73	2.04E-04	1.14E-02
FURIN	-0.13	6.72	-3.73	2.04E-04	1.14E-02
TRMT2A	0.11	7.23	3.73	2.05E-04	1.14E-02
PCGF5	0.40	6.31	3.73	2.05E-04	1.14E-02
PTGER2	-0.58	5.91	-3.73	2.07E-04	1.15E-02
SCRT2	-0.29	6.92	-3.73	2.07E-04	1.15E-02
EPS8	-0.32	9.48	-3.72	2.11E-04	1.16E-02
TMEM51-AS1	0.51	5.01	3.72	2.11E-04	1.16E-02
MRPS21	0.11	10.64	3.72	2.16E-04	1.19E-02
PKP2	0.41	6.64	3.72	2.18E-04	1.19E-02
BOD1L2	0.14	5.62	3.72	2.19E-04	1.19E-02
THEGL	0.15	4.86	3.71	2.19E-04	1.19E-02
CD72	0.17	5.58	3.71	2.21E-04	1.20E-02
ANAPC5	0.11	9.23	3.71	2.21E-04	1.20E-02
PLD2	0.37	7.29	3.71	2.25E-04	1.22E-02
OR2L8	-0.76	6.68	-3.70	2.28E-04	1.22E-02
POFUT2	0.12	6.48	3.70	2.28E-04	1.22E-02
PAAF1	0.15	7.48	3.70	2.28E-04	1.22E-02
PTPRF	-0.30	7.73	-3.70	2.30E-04	1.23E-02
CHIA	0.09	3.66	3.70	2.33E-04	1.24E-02
TMEM98	0.37	8.27	3.70	2.34E-04	1.24E-02
TMEM51	0.49	5.36	3.69	2.37E-04	1.25E-02
NAALAD2	-0.46	4.79	-3.69	2.37E-04	1.25E-02
P3H4	0.13	6.46	3.69	2.39E-04	1.25E-02
TANGO6	-0.24	7.66	-3.69	2.39E-04	1.25E-02
ZNF576	0.10	5.82	3.69	2.40E-04	1.25E-02
GLRA4	0.35	5.36	3.69	2.40E-04	1.25E-02
AKR7L	0.13	6.03	3.69	2.40E-04	1.25E-02
CDNF	0.18	6.60	3.69	2.42E-04	1.26E-02
ATAD2B	-0.14	8.48	-3.69	2.44E-04	1.26E-02
SCLT1	-0.16	7.85	-3.69	2.45E-04	1.26E-02
USP49	-0.23	7.89	-3.68	2.46E-04	1.26E-02
TESK2	-0.33	6.92	-3.68	2.47E-04	1.26E-02
ABCG4	-0.40	6.97	-3.68	2.49E-04	1.27E-02
SGK1	-0.60	8.18	-3.68	2.51E-04	1.27E-02
LRP2	-0.55	5.00	-3.68	2.52E-04	1.27E-02
DYNLRB2	0.14	3.87	3.68	2.52E-04	1.27E-02
HCFC2	-0.27	7.77	-3.68	2.52E-04	1.27E-02
CACNA2D1	-0.58	10.53	-3.67	2.56E-04	1.29E-02
APH1B	0.23	6.79	3.67	2.58E-04	1.29E-02
CDKL5	-0.23	8.15	-3.67	2.58E-04	1.29E-02
NFAM1	0.17	6.15	3.67	2.59E-04	1.29E-02

SPINK1	-0.08	3.31	-3.67	2.60E-04	1.29E-02
LINC00282	-0.23	5.87	-3.67	2.60E-04	1.29E-02
TMEM45A	0.46	5.27	3.67	2.64E-04	1.30E-02
SLC8A1	-0.52	7.81	-3.67	2.64E-04	1.30E-02
AMPH	-0.42	7.64	-3.66	2.72E-04	1.34E-02
NECAP2	0.11	7.88	3.66	2.72E-04	1.34E-02
PTGS2	-0.38	5.90	-3.66	2.74E-04	1.34E-02
HSD17B3	0.29	5.60	3.66	2.75E-04	1.34E-02
KLHL29	-0.33	7.22	-3.65	2.77E-04	1.35E-02
ANKS6	0.25	6.74	3.65	2.79E-04	1.36E-02
NEK8	0.11	5.54	3.65	2.84E-04	1.38E-02
LRRC18	0.36	5.10	3.64	2.87E-04	1.39E-02
MCF2L2	-0.45	6.41	-3.64	2.89E-04	1.39E-02
SHOX2	0.77	6.42	3.64	2.90E-04	1.40E-02
MACROD2-IT1	-0.32	5.07	-3.64	2.93E-04	1.40E-02
DIRAS2	-0.57	7.91	-3.64	2.93E-04	1.40E-02
SPATA2	-0.13	6.81	-3.63	2.98E-04	1.42E-02
FBN3	-0.48	6.95	-3.63	2.99E-04	1.42E-02
CERS2	0.16	9.19	3.63	2.99E-04	1.42E-02
LINC01547	0.12	6.26	3.63	3.03E-04	1.43E-02
FBXO3	-0.15	8.97	-3.63	3.09E-04	1.46E-02
TBX5	0.58	5.91	3.62	3.11E-04	1.46E-02
RASL10A	0.17	5.53	3.62	3.11E-04	1.46E-02
TRIM68	-0.20	7.81	-3.62	3.12E-04	1.46E-02
P2RX6	0.20	5.16	3.62	3.16E-04	1.47E-02
THNSL1	0.19	7.22	3.62	3.16E-04	1.47E-02
RUNX2	0.48	6.16	3.62	3.21E-04	1.49E-02
IFT80	0.23	7.85	3.61	3.24E-04	1.51E-02
GRASP	0.31	6.07	3.61	3.26E-04	1.51E-02
SORBS2	-0.51	7.98	-3.61	3.28E-04	1.52E-02
PLA2G4C	-0.38	5.41	-3.60	3.34E-04	1.54E-02
SPRYD7	-0.17	8.12	-3.60	3.36E-04	1.54E-02
TSC22D3	0.27	7.96	3.60	3.42E-04	1.56E-02
BRINP3	-0.61	5.36	-3.60	3.42E-04	1.56E-02
LOC440700	0.18	3.93	3.60	3.43E-04	1.57E-02
CTRL	0.16	6.19	3.60	3.45E-04	1.57E-02
HCAR2	0.42	4.77	3.59	3.49E-04	1.58E-02
PCDHB10	0.27	8.89	3.59	3.52E-04	1.59E-02
GNPTG	0.10	8.04	3.59	3.53E-04	1.59E-02
PDE6H	-0.50	5.23	-3.59	3.56E-04	1.61E-02
TBC1D1	0.22	8.67	3.58	3.61E-04	1.62E-02
CYB5R2	0.39	5.25	3.58	3.68E-04	1.65E-02
BAGE4	-0.12	8.28	-3.58	3.72E-04	1.67E-02
HMGCR	-0.15	9.06	-3.58	3.73E-04	1.67E-02
PHYHIPL	-0.29	9.17	-3.57	3.77E-04	1.68E-02
MADD	-0.16	9.85	-3.57	3.78E-04	1.68E-02
FAM86JP	0.12	6.74	3.57	3.80E-04	1.68E-02
SLC5A8	-0.36	3.81	-3.57	3.82E-04	1.69E-02
TSPYL5	-0.50	7.93	-3.57	3.84E-04	1.69E-02
INTS11	0.11	8.38	3.57	3.87E-04	1.70E-02
VGLL4	0.14	7.51	3.56	3.89E-04	1.70E-02
IL7R	0.22	4.59	3.56	3.89E-04	1.70E-02
MRTO4	0.16	6.67	3.56	3.93E-04	1.72E-02

IDI1	-0.17	8.70	-3.56	3.95E-04	1.72E-02
CLDN2	0.11	4.60	3.56	3.98E-04	1.73E-02
DOCK3	-0.48	7.87	-3.56	3.99E-04	1.73E-02
PSMB10	0.19	6.66	3.55	4.04E-04	1.75E-02
BEX5	-0.24	5.48	-3.55	4.06E-04	1.75E-02
MDH1B	0.24	5.31	3.55	4.08E-04	1.76E-02
SLC6A17	-0.50	5.84	-3.55	4.16E-04	1.78E-02
INTS6	-0.12	6.95	-3.54	4.18E-04	1.79E-02
DYDC2	0.10	4.57	3.54	4.19E-04	1.79E-02
COG7	0.11	7.87	3.54	4.21E-04	1.80E-02
TWIST2	0.30	5.37	3.54	4.23E-04	1.80E-02
CBLN1	-0.50	8.03	-3.54	4.27E-04	1.81E-02
ALK	0.53	5.95	3.54	4.32E-04	1.83E-02
CLN3	0.12	7.09	3.54	4.33E-04	1.83E-02
UGP2	-0.18	7.77	-3.53	4.40E-04	1.86E-02
EHMT1	0.12	8.02	3.53	4.48E-04	1.88E-02
CACNB2	-0.39	6.91	-3.52	4.51E-04	1.89E-02
ARAP3	0.29	6.69	3.52	4.54E-04	1.90E-02
KIAA1147	-0.24	9.09	-3.52	4.57E-04	1.91E-02
MYLK2	0.28	5.69	3.52	4.58E-04	1.91E-02
METTL26	0.12	7.09	3.52	4.61E-04	1.91E-02
RPS6KL1	-0.28	7.19	-3.52	4.61E-04	1.91E-02
SYCP1	-0.58	3.71	-3.52	4.62E-04	1.91E-02
CMTM5	0.33	5.35	3.51	4.68E-04	1.93E-02
MIR135A2	0.27	2.85	3.51	4.69E-04	1.93E-02
CHRM5	-0.19	5.19	-3.51	4.70E-04	1.93E-02
KCNIP2	-0.35	7.40	-3.51	4.72E-04	1.94E-02
KIAA0513	-0.28	7.27	-3.50	4.99E-04	2.04E-02
CLDN16	0.13	4.38	3.50	5.00E-04	2.04E-02
SHISA7	-0.26	5.10	-3.50	5.01E-04	2.04E-02
HMGCL	0.13	6.41	3.49	5.04E-04	2.05E-02
FIGN	-0.48	5.68	-3.49	5.09E-04	2.06E-02
RSPH6A	0.08	5.66	3.49	5.10E-04	2.06E-02
LMNB1	-0.20	8.45	-3.49	5.15E-04	2.08E-02
SMYD5	0.11	7.49	3.49	5.19E-04	2.09E-02
PRSS53	-0.12	5.90	-3.49	5.19E-04	2.09E-02
CNGA1	-0.35	4.26	-3.49	5.20E-04	2.09E-02
GPD1L	-0.25	5.81	-3.48	5.23E-04	2.09E-02
VPS13A	-0.25	6.87	-3.48	5.25E-04	2.10E-02
ZMAT5	0.11	6.85	3.48	5.28E-04	2.11E-02
GNG8	0.19	5.90	3.48	5.32E-04	2.12E-02
ACKR3	0.22	4.52	3.48	5.34E-04	2.12E-02
NOS1AP	-0.29	6.96	-3.48	5.34E-04	2.12E-02
TRHDE	-0.55	5.41	-3.48	5.36E-04	2.12E-02
DCTD	0.22	8.91	3.48	5.39E-04	2.12E-02
CBY1	0.11	7.12	3.48	5.40E-04	2.12E-02
PPP3R1	-0.13	8.95	-3.47	5.44E-04	2.13E-02
SCGB2B2	0.26	6.56	3.47	5.44E-04	2.13E-02
PLA2G6	0.16	7.10	3.47	5.46E-04	2.13E-02
TCTN3	0.15	8.40	3.47	5.46E-04	2.13E-02
CCDC113	0.26	6.97	3.47	5.48E-04	2.13E-02
JAK1	0.11	9.48	3.47	5.50E-04	2.14E-02
SNTB2	-0.27	8.17	-3.47	5.52E-04	2.14E-02

SMAP1	-0.15	8.83	-3.47	5.54E-04	2.14E-02
TIPARP	-0.18	7.93	-3.47	5.57E-04	2.14E-02
LINC01124	0.32	4.66	3.47	5.59E-04	2.14E-02
ST3GAL6	-0.31	7.20	-3.47	5.59E-04	2.14E-02
NRSN1	-0.38	6.89	-3.47	5.60E-04	2.14E-02
GDPD5	-0.28	7.02	-3.47	5.60E-04	2.14E-02
LPIN3	0.14	4.99	3.47	5.61E-04	2.14E-02
STXBP5	-0.21	8.19	-3.47	5.61E-04	2.14E-02
MLST8	0.11	7.84	3.46	5.62E-04	2.14E-02
ARID3A	-0.13	5.97	-3.46	5.64E-04	2.14E-02
CEP68	0.16	8.33	3.46	5.66E-04	2.14E-02
EVC	0.42	6.49	3.46	5.71E-04	2.16E-02
ESRRB	0.39	5.17	3.46	5.77E-04	2.18E-02
HPD	0.17	4.28	3.46	5.78E-04	2.18E-02
H6PD	0.15	6.41	3.46	5.82E-04	2.19E-02
USP2	-0.54	7.58	-3.45	5.83E-04	2.19E-02
F12	0.10	5.39	3.45	5.86E-04	2.19E-02
ALDH5A1	-0.27	7.19	-3.45	5.88E-04	2.20E-02
SLC25A16	-0.11	7.20	-3.45	6.02E-04	2.24E-02
CASP8AP2	-0.14	8.00	-3.45	6.03E-04	2.24E-02
RCAN1	-0.19	6.63	-3.44	6.05E-04	2.25E-02
MMEL1	0.10	4.35	3.44	6.10E-04	2.25E-02
NOL3	0.16	7.14	3.44	6.11E-04	2.25E-02
PAX5	0.48	7.07	3.44	6.11E-04	2.25E-02
METTL13	0.14	7.33	3.44	6.12E-04	2.25E-02
CELF1	-0.10	9.86	-3.44	6.13E-04	2.25E-02
NQO1	0.24	6.07	3.44	6.16E-04	2.26E-02
PDE9A	-0.31	8.66	-3.44	6.22E-04	2.28E-02
OSCP1	0.15	6.66	3.44	6.24E-04	2.28E-02
ZFAND4	-0.23	7.66	-3.44	6.25E-04	2.28E-02
ZNF346	0.10	7.72	3.43	6.31E-04	2.29E-02
CDS2	-0.12	9.75	-3.43	6.33E-04	2.29E-02
HHLA2	0.16	3.68	3.43	6.34E-04	2.29E-02
DMC1	-0.24	3.72	-3.43	6.38E-04	2.29E-02
GABRA5	-0.71	6.62	-3.43	6.38E-04	2.29E-02
CRYZ	0.39	8.28	3.43	6.39E-04	2.29E-02
PRPH2	-0.63	6.31	-3.43	6.39E-04	2.29E-02
MYO15B	0.31	7.03	3.43	6.40E-04	2.29E-02
NEUROD1	-0.49	10.28	-3.43	6.43E-04	2.30E-02
GNAI2	0.17	9.17	3.43	6.47E-04	2.31E-02
LDHA	-0.28	10.55	-3.43	6.47E-04	2.31E-02
TBC1D26	0.21	5.56	3.43	6.48E-04	2.31E-02
ZNF480	0.13	8.62	3.42	6.56E-04	2.33E-02
SESTD1	-0.16	9.22	-3.42	6.61E-04	2.34E-02
DTNB	-0.16	8.35	-3.42	6.62E-04	2.34E-02
TEX2	-0.29	8.48	-3.42	6.65E-04	2.34E-02
MYCBP2	-0.18	9.14	-3.42	6.65E-04	2.34E-02
TDRD12	0.19	4.14	3.42	6.67E-04	2.35E-02
ENC1	-0.61	8.28	-3.42	6.70E-04	2.35E-02
ATXN7	-0.20	10.33	-3.41	6.75E-04	2.37E-02
MAP3K11	0.12	7.10	3.41	6.77E-04	2.37E-02
SERPINF2	0.19	5.34	3.41	6.82E-04	2.38E-02
LTA4H	0.18	8.89	3.41	6.82E-04	2.38E-02

C9orf50	0.09	4.78	3.41	6.83E-04	2.38E-02
SHROOM1	0.09	6.27	3.41	6.92E-04	2.40E-02
BMP8B	0.24	5.46	3.40	7.00E-04	2.42E-02
DNAH5	0.26	4.97	3.40	7.00E-04	2.42E-02
EPHA6	-0.62	5.90	-3.40	7.02E-04	2.42E-02
IRF3	0.14	6.86	3.40	7.08E-04	2.44E-02
DUOX1	0.25	5.70	3.40	7.22E-04	2.48E-02
C14orf79	0.13	6.22	3.40	7.22E-04	2.48E-02
Sep-06	-0.30	9.26	-3.40	7.23E-04	2.48E-02
MYT1L	-0.55	9.32	-3.39	7.24E-04	2.48E-02
PAQR9	-0.29	5.54	-3.39	7.26E-04	2.48E-02
MLXIP	0.19	8.97	3.39	7.30E-04	2.48E-02
ANXA4	0.31	6.69	3.39	7.30E-04	2.48E-02
SGTB	-0.18	8.24	-3.39	7.33E-04	2.49E-02
CLUAP1	0.17	7.73	3.39	7.39E-04	2.50E-02
FAM234B	-0.25	8.33	-3.39	7.39E-04	2.50E-02
DPH2	0.14	7.06	3.39	7.43E-04	2.51E-02
FZD10	0.37	5.79	3.39	7.44E-04	2.51E-02
TWF2	0.19	7.92	3.38	7.55E-04	2.54E-02
YBEY	0.12	6.11	3.38	7.55E-04	2.54E-02
TMPRSS11B	-0.06	2.72	-3.38	7.59E-04	2.54E-02
CCNO	0.10	5.76	3.38	7.60E-04	2.54E-02
SMURF1	-0.14	8.29	-3.38	7.62E-04	2.55E-02
STRADB	-0.20	10.01	-3.38	7.64E-04	2.55E-02
KATNAL2	0.21	6.48	3.38	7.74E-04	2.58E-02
CDC7	-0.19	6.46	-3.37	7.79E-04	2.58E-02
ZNRF3-AS1	0.12	4.10	3.37	7.80E-04	2.58E-02
LCN12	0.10	5.58	3.37	7.85E-04	2.59E-02
RALGDS	0.17	8.12	3.37	7.86E-04	2.59E-02
ETV1	-0.40	9.02	-3.37	7.86E-04	2.59E-02
FHAD1	0.10	4.44	3.37	7.87E-04	2.59E-02
NKD1	0.51	7.73	3.37	7.89E-04	2.59E-02
FBXW7	-0.22	7.77	-3.37	7.93E-04	2.60E-02
FLJ34503	-0.19	5.07	-3.37	7.95E-04	2.60E-02
ZNF654	-0.15	7.67	-3.37	7.96E-04	2.60E-02
TRUB2	0.13	7.87	3.36	8.09E-04	2.64E-02
ZNRF3	0.26	7.07	3.36	8.11E-04	2.64E-02
MOSPD2	-0.14	7.72	-3.36	8.14E-04	2.64E-02
RAB11A	0.11	9.39	3.36	8.15E-04	2.64E-02
EVL	-0.18	8.80	-3.36	8.15E-04	2.64E-02
CITED1	-0.23	6.19	-3.36	8.16E-04	2.64E-02
KCNQ1	0.16	5.11	3.36	8.21E-04	2.65E-02
POU6F1	-0.18	7.91	-3.36	8.24E-04	2.65E-02
PDE11A	0.56	4.70	3.36	8.26E-04	2.66E-02
PARD3B	0.26	5.81	3.36	8.31E-04	2.66E-02
TRAPPC6A	0.14	6.23	3.36	8.31E-04	2.66E-02
GUCY1B3	-0.29	7.28	-3.35	8.36E-04	2.67E-02
F8	0.16	5.89	3.35	8.37E-04	2.67E-02
CLMN	-0.37	6.49	-3.35	8.41E-04	2.68E-02
RNGTT	-0.15	8.53	-3.35	8.43E-04	2.69E-02
RP1	-0.47	3.87	-3.35	8.47E-04	2.69E-02
VAMP3	0.10	8.43	3.35	8.48E-04	2.69E-02
IMPG1	-0.39	4.67	-3.35	8.51E-04	2.69E-02

RPGRIP1L	0.20	7.62	3.35	8.54E-04	2.70E-02
SSPN	0.22	7.06	3.35	8.59E-04	2.71E-02
SLC39A8	0.25	7.39	3.35	8.59E-04	2.71E-02
TSPAN5	0.28	9.43	3.34	8.66E-04	2.72E-02
CEP126	0.40	6.45	3.34	8.67E-04	2.72E-02
PPM1E	-0.26	7.60	-3.34	8.68E-04	2.72E-02
ABCA7	-0.35	5.86	-3.34	8.70E-04	2.72E-02
NFIA	0.37	11.04	3.34	8.77E-04	2.74E-02
ARL6IP1	-0.12	12.64	-3.34	8.83E-04	2.75E-02
B3GAT2	-0.25	5.78	-3.34	8.85E-04	2.75E-02
KRTAP5-8	0.13	7.31	3.34	8.86E-04	2.75E-02
PLPPR1	-0.37	8.07	-3.34	8.88E-04	2.75E-02
AP1S3	-0.23	5.27	-3.34	8.89E-04	2.75E-02
ANTXR1	0.27	8.49	3.34	8.94E-04	2.76E-02
FJX1	0.27	6.61	3.34	8.95E-04	2.76E-02
LRRC23	0.12	6.65	3.33	8.99E-04	2.77E-02
DICER1	-0.13	9.63	-3.33	9.01E-04	2.77E-02
RANBP6	-0.15	9.04	-3.33	9.03E-04	2.77E-02
ACVR1	-0.25	8.21	-3.33	9.06E-04	2.78E-02
ECEL1	0.24	5.31	3.33	9.09E-04	2.78E-02
ADAM15	0.17	6.87	3.33	9.17E-04	2.80E-02
ZNF688	0.12	6.88	3.33	9.21E-04	2.81E-02
UBXN11	0.16	6.44	3.33	9.22E-04	2.81E-02
HAND2	0.30	4.57	3.33	9.24E-04	2.81E-02
GCNT4	-0.35	4.42	-3.33	9.26E-04	2.81E-02
PAFAH2	0.12	6.80	3.32	9.30E-04	2.82E-02
SAMD7	-0.47	4.57	-3.32	9.32E-04	2.82E-02
ADGRL1	-0.27	8.93	-3.32	9.42E-04	2.84E-02
PDZD7	-0.17	5.78	-3.32	9.51E-04	2.87E-02
ACTR3B	0.22	8.74	3.31	9.63E-04	2.89E-02
PPP2R5E	-0.11	8.49	-3.31	9.63E-04	2.89E-02
C10orf11	0.19	5.00	3.31	9.72E-04	2.92E-02
MEST	0.27	10.70	3.31	9.73E-04	2.92E-02
ASRGL1	-0.18	7.63	-3.31	9.75E-04	2.92E-02
COQ9	0.13	8.80	3.31	9.88E-04	2.95E-02
ZNF860	0.13	3.84	3.31	9.91E-04	2.96E-02
ZBTB11-AS1	0.14	7.09	3.31	9.93E-04	2.96E-02
FKBP10	0.48	7.57	3.30	9.98E-04	2.96E-02
ADCK5	-0.11	5.68	-3.30	9.98E-04	2.96E-02
SLC16A14	-0.37	7.82	-3.30	1.00E-03	2.97E-02
ZNF174	0.09	7.20	3.30	1.00E-03	2.97E-02
RHOU	0.28	8.82	3.30	1.01E-03	2.98E-02
HOXB2	0.41	5.73	3.30	1.01E-03	2.98E-02
CELF2	-0.29	9.20	-3.30	1.01E-03	2.98E-02
PI3	-0.08	4.34	-3.30	1.01E-03	2.98E-02
LINC01879	-0.07	3.60	-3.30	1.02E-03	2.99E-02
NPAP1	0.19	5.77	3.30	1.02E-03	2.99E-02
HOXA11	0.21	3.52	3.30	1.02E-03	2.99E-02
CDK15	0.23	4.12	3.30	1.03E-03	3.00E-02
ZBTB20	0.26	9.98	3.30	1.03E-03	3.01E-02
SCUBE1	0.17	5.70	3.29	1.03E-03	3.01E-02
DIRAS1	-0.16	8.17	-3.29	1.04E-03	3.02E-02
SSX2IP	-0.23	7.78	-3.29	1.04E-03	3.02E-02

PDE6G	-0.40	5.45	-3.29	1.05E-03	3.03E-02
ZNFX1	-0.11	7.78	-3.29	1.06E-03	3.07E-02
GPR139	-0.31	7.28	-3.28	1.07E-03	3.09E-02
RAB3IL1	0.12	5.97	3.28	1.07E-03	3.09E-02
OR6T1	-0.09	4.05	-3.28	1.07E-03	3.09E-02
CCDC148	0.29	4.98	3.28	1.08E-03	3.11E-02
GLCE	0.28	9.81	3.28	1.08E-03	3.11E-02
EVC2	0.29	4.80	3.28	1.10E-03	3.15E-02
ELAVL2	-0.32	9.10	-3.28	1.10E-03	3.15E-02
RORA	-0.25	7.97	-3.27	1.11E-03	3.16E-02
ZNF556	0.17	5.52	3.27	1.11E-03	3.17E-02
TSPAN14	0.18	8.50	3.27	1.11E-03	3.17E-02
INHBB	-0.24	7.11	-3.27	1.12E-03	3.17E-02
MEGF8	0.15	6.58	3.27	1.12E-03	3.17E-02
NHLRC1	-0.20	5.85	-3.27	1.12E-03	3.17E-02
ING2	-0.17	6.48	-3.27	1.12E-03	3.17E-02
WDR45	0.11	6.99	3.27	1.12E-03	3.17E-02
SLC35B4	-0.15	8.38	-3.27	1.13E-03	3.19E-02
COX8C	0.27	6.14	3.27	1.13E-03	3.19E-02
HIST1H4D	-0.44	7.51	-3.27	1.14E-03	3.20E-02
RASGRF2	-0.53	7.38	-3.26	1.15E-03	3.23E-02
ZNF30	-0.14	5.78	-3.26	1.16E-03	3.23E-02
TGFBR3	-0.37	7.25	-3.26	1.16E-03	3.23E-02
SND1-IT1	-0.21	7.93	-3.26	1.16E-03	3.23E-02
GPBP1L1	0.11	8.62	3.26	1.16E-03	3.23E-02
ADCY9	-0.16	7.43	-3.26	1.16E-03	3.23E-02
CACUL1	0.11	8.95	3.26	1.16E-03	3.23E-02
FKTN	0.12	7.03	3.26	1.17E-03	3.23E-02
MYH2	-0.06	3.59	-3.26	1.17E-03	3.23E-02
AOX1	0.21	4.31	3.26	1.17E-03	3.23E-02
CCNDBP1	0.11	7.65	3.26	1.17E-03	3.23E-02
SESN2	-0.16	6.33	-3.26	1.17E-03	3.23E-02
AFMID	0.25	7.46	3.26	1.17E-03	3.23E-02
PRR19	0.11	4.57	3.26	1.18E-03	3.24E-02
RARB	0.41	6.14	3.26	1.18E-03	3.24E-02
H2AFJ	-0.34	6.22	-3.25	1.19E-03	3.26E-02
SLC22A23	-0.37	7.26	-3.25	1.19E-03	3.26E-02
PRKCE	-0.29	6.39	-3.25	1.20E-03	3.27E-02
SERPINE2	0.37	7.05	3.25	1.20E-03	3.27E-02
E2F6	-0.11	7.42	-3.25	1.20E-03	3.27E-02
SWAP70	-0.16	7.13	-3.25	1.21E-03	3.29E-02
LRRK2	-0.19	4.92	-3.25	1.21E-03	3.31E-02
STON2	0.26	6.24	3.25	1.22E-03	3.32E-02
NFKB2	0.22	5.64	3.25	1.22E-03	3.32E-02
ARSD	0.14	7.26	3.24	1.23E-03	3.32E-02
AP1G2	0.17	7.39	3.24	1.23E-03	3.32E-02
TMEM234	0.12	7.24	3.24	1.24E-03	3.33E-02
CD96	0.15	4.24	3.24	1.24E-03	3.33E-02
DOCK9	-0.30	8.65	-3.24	1.24E-03	3.34E-02
PPM1D	-0.22	9.09	-3.24	1.25E-03	3.35E-02
MIR186	0.19	5.71	3.24	1.25E-03	3.35E-02
EFNA2	-0.15	5.83	-3.24	1.25E-03	3.36E-02
ENTPD5	0.11	6.85	3.24	1.26E-03	3.36E-02

ICK	-0.17	9.38	-3.24	1.26E-03	3.36E-02
AKR7A2	0.10	8.60	3.24	1.26E-03	3.36E-02
EDARADD	0.21	4.76	3.24	1.26E-03	3.37E-02
SNX18	-0.13	6.56	-3.23	1.28E-03	3.40E-02
BAGE5	-0.11	8.24	-3.23	1.28E-03	3.41E-02
FGFRL1	0.20	7.33	3.23	1.29E-03	3.42E-02
GCM1	0.30	4.23	3.23	1.29E-03	3.42E-02
MAP3K9	-0.25	7.44	-3.23	1.30E-03	3.43E-02
PDGFC	0.37	7.25	3.23	1.30E-03	3.44E-02
THBS3	0.28	6.95	3.23	1.30E-03	3.44E-02
MAP3K13	-0.37	8.49	-3.23	1.31E-03	3.44E-02
TMEM179	-0.23	6.79	-3.23	1.31E-03	3.44E-02
PALMD	-0.44	7.42	-3.23	1.31E-03	3.44E-02
ULK4	0.22	5.85	3.23	1.31E-03	3.44E-02
AK8	0.17	6.13	3.23	1.31E-03	3.44E-02
MXRA7	-0.21	9.68	-3.22	1.32E-03	3.45E-02
SERPINB8	0.18	5.94	3.22	1.32E-03	3.45E-02
SLC35D2	0.27	6.66	3.22	1.33E-03	3.46E-02
TK2	0.15	7.37	3.22	1.33E-03	3.46E-02
TSPAN17	0.22	7.41	3.22	1.34E-03	3.47E-02
UBXN6	0.11	7.70	3.22	1.34E-03	3.47E-02
LINC01658	-0.14	4.62	-3.22	1.34E-03	3.47E-02
MAF	0.38	5.62	3.22	1.34E-03	3.47E-02
FAM86C1	0.11	6.16	3.22	1.34E-03	3.47E-02
RSRC1	0.22	8.73	3.22	1.35E-03	3.49E-02
NR4A3	-0.32	6.51	-3.22	1.35E-03	3.49E-02
RGL1	0.43	8.01	3.22	1.36E-03	3.50E-02
PGM1	0.41	7.93	3.21	1.37E-03	3.51E-02
DPPA4	-0.07	4.14	-3.21	1.37E-03	3.51E-02
TMEM9	0.12	7.12	3.21	1.37E-03	3.51E-02
KIDINS220	-0.17	9.50	-3.21	1.37E-03	3.51E-02
EXOC3L1	0.25	6.04	3.21	1.37E-03	3.51E-02
SAP30L	0.11	7.61	3.21	1.38E-03	3.53E-02
ABHD13	-0.10	9.51	-3.21	1.39E-03	3.54E-02
PDPN	0.43	6.90	3.21	1.39E-03	3.54E-02
GPR35	0.12	5.82	3.21	1.39E-03	3.54E-02
PRMT2	0.11	7.61	3.21	1.39E-03	3.54E-02
PSD2	0.27	7.08	3.21	1.40E-03	3.55E-02
MYH7	0.14	5.96	3.21	1.40E-03	3.55E-02
RELL1	-0.25	8.21	-3.21	1.40E-03	3.55E-02
ASB13	0.29	7.28	3.21	1.41E-03	3.56E-02
GEMIN5	0.11	7.72	3.20	1.42E-03	3.59E-02
TAB2	-0.10	8.42	-3.20	1.43E-03	3.60E-02
PBXIP1	0.11	5.84	3.20	1.43E-03	3.60E-02
GNAO1	-0.32	10.31	-3.20	1.43E-03	3.60E-02
CETN2	0.12	8.92	3.20	1.44E-03	3.60E-02
WFDC2	0.15	5.43	3.20	1.44E-03	3.60E-02
FAM98A	-0.12	8.61	-3.20	1.44E-03	3.60E-02
SEC14L1	-0.12	7.88	-3.20	1.46E-03	3.63E-02
CKB	-0.34	9.74	-3.20	1.46E-03	3.63E-02
LBR	-0.16	8.69	-3.20	1.46E-03	3.63E-02
ZSWIM6	-0.14	9.94	-3.19	1.47E-03	3.66E-02
CLTC	-0.15	10.17	-3.19	1.47E-03	3.66E-02

ZNF69	-0.17	8.07	-3.19	1.48E-03	3.66E-02
PTER	-0.27	5.56	-3.19	1.48E-03	3.66E-02
ATP1A2	0.32	8.19	3.19	1.48E-03	3.66E-02
FAM81A	-0.18	5.43	-3.19	1.48E-03	3.66E-02
WWP1	-0.14	8.95	-3.19	1.48E-03	3.66E-02
FAIM	0.17	5.87	3.19	1.51E-03	3.72E-02
RSPO1	0.35	5.43	3.18	1.51E-03	3.72E-02
GABRB3	-0.19	9.88	-3.18	1.52E-03	3.72E-02
SNAP91	-0.31	9.45	-3.18	1.52E-03	3.72E-02
ANKRD18B	-0.30	4.80	-3.18	1.53E-03	3.73E-02
EPB41L4B	0.39	7.32	3.18	1.53E-03	3.73E-02
NKX2-5	0.12	6.19	3.18	1.53E-03	3.73E-02
AFG1L	-0.13	7.06	-3.18	1.53E-03	3.74E-02
AMPD2	0.21	6.87	3.18	1.54E-03	3.75E-02
REPS1	-0.18	9.78	-3.18	1.54E-03	3.75E-02
SYTL2	0.19	5.80	3.18	1.54E-03	3.75E-02
SCNN1B	0.22	5.69	3.18	1.55E-03	3.75E-02
TTLL10	0.08	5.76	3.18	1.55E-03	3.75E-02
GPRIN3	-0.37	7.87	-3.18	1.55E-03	3.75E-02
PIK3C2G	-0.09	3.60	-3.18	1.55E-03	3.75E-02
TMEM45B	0.17	5.09	3.18	1.55E-03	3.75E-02
BSPH1	-0.52	4.34	-3.18	1.55E-03	3.75E-02
C4orf33	-0.21	6.97	-3.18	1.56E-03	3.75E-02
JAZF1	-0.29	7.63	-3.18	1.56E-03	3.75E-02
SNX25	-0.15	8.21	-3.17	1.56E-03	3.75E-02
SLC18A1	0.14	4.73	3.17	1.57E-03	3.76E-02
CLCC1	0.08	9.33	3.17	1.57E-03	3.77E-02
ERCC1	0.11	7.95	3.17	1.57E-03	3.77E-02
FOXS1	0.18	6.21	3.17	1.59E-03	3.79E-02
USP50	0.09	4.17	3.17	1.59E-03	3.79E-02
MT1F	0.33	10.64	3.17	1.59E-03	3.79E-02
KCTD10	-0.13	8.35	-3.17	1.59E-03	3.79E-02
BLID	0.44	5.36	3.17	1.60E-03	3.81E-02
ACAT2	-0.17	6.89	-3.17	1.61E-03	3.81E-02
TMEM53	0.15	7.55	3.17	1.61E-03	3.81E-02
DBP	-0.20	7.56	-3.17	1.61E-03	3.81E-02
NUDT6	0.11	6.41	3.17	1.61E-03	3.81E-02
IFT57	0.34	8.27	3.16	1.62E-03	3.82E-02
TRIO	0.15	10.24	3.16	1.62E-03	3.82E-02
LMTK2	-0.14	8.60	-3.16	1.62E-03	3.82E-02
MTFR1	-0.15	8.39	-3.16	1.63E-03	3.82E-02
ZNF691	0.10	6.41	3.16	1.63E-03	3.82E-02
FLT4	0.30	5.31	3.16	1.63E-03	3.82E-02
VAV2	0.20	7.21	3.16	1.64E-03	3.82E-02
PRR5L	0.28	5.94	3.16	1.64E-03	3.82E-02
SLC38A1	-0.42	10.22	-3.16	1.64E-03	3.82E-02
ZNF169	0.10	6.05	3.16	1.64E-03	3.82E-02
TNNC1	0.16	5.53	3.16	1.64E-03	3.82E-02
ALDH1B1	0.18	8.12	3.16	1.64E-03	3.82E-02
PLCD4	0.29	5.27	3.16	1.67E-03	3.87E-02
MRPL24	0.11	7.34	3.16	1.67E-03	3.88E-02
FBXO18	0.13	7.83	3.15	1.67E-03	3.88E-02
KCNJ6	0.48	7.33	3.15	1.68E-03	3.89E-02

ACKR1	-0.29	7.69	-3.15	1.68E-03	3.89E-02
KCTD17	0.18	7.09	3.15	1.68E-03	3.89E-02
TMEM67	0.18	7.42	3.15	1.69E-03	3.90E-02
SVIL	0.31	6.61	3.15	1.70E-03	3.93E-02
SAMD3	-0.69	6.18	-3.15	1.71E-03	3.93E-02
TSNAXIP1	0.11	5.06	3.15	1.73E-03	3.97E-02
CNOT4	-0.12	9.45	-3.14	1.73E-03	3.97E-02
DMRT2	0.25	3.91	3.14	1.73E-03	3.97E-02
FAM169A	-0.31	6.75	-3.14	1.73E-03	3.97E-02
RDH8	-0.20	4.57	-3.14	1.74E-03	3.98E-02
HAPLN4	0.20	5.53	3.14	1.75E-03	3.99E-02
G2E3	-0.14	8.16	-3.14	1.75E-03	3.99E-02
TADA3	0.10	8.27	3.14	1.75E-03	3.99E-02
BIRC6	-0.08	9.45	-3.14	1.76E-03	4.01E-02
FAM124B	0.17	5.11	3.14	1.76E-03	4.01E-02
FBXO5	-0.22	8.10	-3.14	1.76E-03	4.01E-02
GNRHR2	-0.15	6.09	-3.14	1.77E-03	4.01E-02
MYB	-0.34	6.80	-3.14	1.77E-03	4.01E-02
MAML3	0.21	9.56	3.14	1.77E-03	4.01E-02
CEP250	-0.11	6.35	-3.14	1.78E-03	4.03E-02
RASA3	0.24	6.71	3.13	1.79E-03	4.04E-02
PPCS	0.09	6.56	3.13	1.79E-03	4.04E-02
GEMIN8	0.13	7.24	3.13	1.79E-03	4.04E-02
LANCL2	-0.17	9.69	-3.13	1.81E-03	4.08E-02
ELOVL3	-0.07	3.65	-3.13	1.82E-03	4.08E-02
WDR81	0.14	6.54	3.13	1.82E-03	4.09E-02
MYRIP	-0.35	7.76	-3.13	1.83E-03	4.10E-02
USP13	0.17	8.54	3.13	1.83E-03	4.10E-02
C22orf31	0.17	3.77	3.13	1.83E-03	4.10E-02
GAB1	0.41	7.01	3.13	1.84E-03	4.10E-02
LOC151174	-0.12	5.25	-3.13	1.84E-03	4.10E-02
TEAD2	0.28	7.72	3.13	1.84E-03	4.10E-02
ALDH6A1	0.12	8.07	3.12	1.85E-03	4.12E-02
SLC12A4	0.23	6.66	3.12	1.86E-03	4.12E-02
GCGR	0.20	5.38	3.12	1.86E-03	4.12E-02
FBXL7	0.41	7.35	3.12	1.86E-03	4.12E-02
STK40	-0.11	7.40	-3.12	1.86E-03	4.12E-02
LOC648987	0.31	6.36	3.12	1.90E-03	4.19E-02
NDUFA9	-0.13	10.06	-3.12	1.91E-03	4.21E-02
NTM-AS1	-0.14	4.38	-3.12	1.91E-03	4.21E-02
ARL5A	-0.11	7.78	-3.11	1.91E-03	4.21E-02
OR5R1	-0.09	4.36	-3.11	1.92E-03	4.23E-02
BRMS1L	-0.21	5.66	-3.11	1.92E-03	4.23E-02
PEX19	0.10	7.81	3.11	1.93E-03	4.23E-02
ATG4A	-0.13	6.13	-3.11	1.93E-03	4.23E-02
KAT2B	-0.25	8.86	-3.11	1.93E-03	4.23E-02
ROR2	0.45	6.07	3.11	1.94E-03	4.24E-02
BCKDK	0.11	7.74	3.11	1.94E-03	4.24E-02
BDNF	-0.21	4.44	-3.11	1.95E-03	4.25E-02
CPSF4L	0.16	4.58	3.11	1.95E-03	4.25E-02
ADSSL1	0.19	6.27	3.11	1.96E-03	4.27E-02
RACGAP1	-0.17	9.35	-3.11	1.97E-03	4.28E-02
RASSF3	0.25	5.70	3.10	1.98E-03	4.31E-02

GAD2	0.28	4.24	3.10	1.99E-03	4.32E-02
NFATC1	0.42	6.33	3.10	2.00E-03	4.34E-02
EVI5L	-0.19	7.09	-3.10	2.01E-03	4.35E-02
RAX2	-0.18	5.99	-3.10	2.01E-03	4.35E-02
FGF2	0.22	6.79	3.10	2.02E-03	4.35E-02
PTGES2-AS1	0.06	3.60	3.10	2.02E-03	4.35E-02
VSTM2L	-0.23	6.15	-3.10	2.02E-03	4.35E-02
THAP7	0.09	7.17	3.10	2.02E-03	4.35E-02
ANGPTL2	0.54	8.21	3.10	2.02E-03	4.35E-02
LRRC4	-0.31	7.26	-3.10	2.03E-03	4.37E-02
DHX34	0.11	7.19	3.10	2.04E-03	4.37E-02
NAA16	-0.10	8.48	-3.09	2.04E-03	4.37E-02
LZTR1	0.09	8.00	3.09	2.04E-03	4.37E-02
MKRN3	0.18	6.98	3.09	2.05E-03	4.38E-02
RRM2	-0.27	6.95	-3.09	2.05E-03	4.38E-02
DACT1	0.39	7.61	3.09	2.06E-03	4.39E-02
COL11A2	-0.17	6.23	-3.09	2.06E-03	4.39E-02
TMEM63A	0.27	6.87	3.09	2.06E-03	4.39E-02
SKA2	-0.22	9.79	-3.09	2.07E-03	4.39E-02
MRRF	0.12	7.94	3.09	2.07E-03	4.40E-02
BDH1	0.26	6.28	3.09	2.08E-03	4.41E-02
OR5D14	-0.06	3.10	-3.09	2.09E-03	4.42E-02
ACHE	-0.30	6.33	-3.09	2.10E-03	4.44E-02
ZMYM1	0.12	7.16	3.09	2.10E-03	4.44E-02
IGSF8	0.14	7.54	3.09	2.11E-03	4.44E-02
SLC30A10	0.21	6.21	3.08	2.12E-03	4.46E-02
LSR	0.17	6.08	3.08	2.12E-03	4.46E-02
NOTUM	0.28	4.81	3.08	2.13E-03	4.48E-02
LRRC43	0.08	4.34	3.08	2.13E-03	4.48E-02
SFXN2	0.20	7.22	3.08	2.14E-03	4.48E-02
CPNE8	-0.26	5.97	-3.08	2.15E-03	4.51E-02
OR2AK2	-0.44	6.16	-3.08	2.16E-03	4.52E-02
DCLK1	-0.31	9.30	-3.08	2.16E-03	4.52E-02
FTCD	0.07	5.60	3.08	2.17E-03	4.52E-02
ZNF398	-0.14	8.53	-3.08	2.18E-03	4.55E-02
CACNA1D	0.36	6.95	3.07	2.19E-03	4.55E-02
SYNE1	-0.31	7.22	-3.07	2.19E-03	4.56E-02
NSUN4	0.11	7.92	3.07	2.20E-03	4.58E-02
GNAT1	-0.59	5.62	-3.07	2.21E-03	4.58E-02
KRTAP5-9	0.12	5.42	3.07	2.21E-03	4.58E-02
KBTBD2	-0.11	8.54	-3.07	2.21E-03	4.58E-02
GSN-AS1	0.12	4.15	3.07	2.21E-03	4.58E-02
CAMKK1	0.27	7.28	3.07	2.22E-03	4.59E-02
IL2	-0.05	2.86	-3.07	2.23E-03	4.59E-02
CFTR	-0.23	4.02	-3.07	2.23E-03	4.59E-02
Mar-01	-0.32	8.18	-3.07	2.23E-03	4.59E-02
ARSG	-0.21	7.22	-3.07	2.23E-03	4.59E-02
ADAMTS10	-0.25	7.35	-3.07	2.24E-03	4.59E-02
SNORA69	0.12	3.31	3.07	2.24E-03	4.59E-02
FRMD4B	-0.33	8.86	-3.07	2.24E-03	4.59E-02
RNF10	-0.09	9.04	-3.07	2.25E-03	4.59E-02
PDC	-0.29	5.29	-3.07	2.25E-03	4.59E-02
DOK6	-0.55	7.88	-3.07	2.25E-03	4.59E-02

NAPB	-0.23	9.22	-3.07	2.25E-03	4.59E-02
SNRNP48	-0.13	8.11	-3.07	2.25E-03	4.59E-02
CXorf38	0.09	6.85	3.06	2.26E-03	4.61E-02
LHX4	-0.53	8.17	-3.06	2.27E-03	4.62E-02
BEST3	0.23	3.70	3.06	2.27E-03	4.62E-02
CHST11	0.29	7.96	3.06	2.28E-03	4.62E-02
C16orf89	0.25	6.68	3.06	2.28E-03	4.63E-02
INSRR	0.28	6.29	3.06	2.28E-03	4.63E-02
SMUG1	0.11	6.72	3.06	2.29E-03	4.63E-02
RBBP9	0.17	7.34	3.06	2.29E-03	4.63E-02
NUPR1	0.23	6.77	3.06	2.29E-03	4.63E-02
MCF2	-0.22	4.33	-3.06	2.30E-03	4.64E-02
CITED4	0.15	5.53	3.06	2.30E-03	4.64E-02
IGF2BP2-AS1	-0.07	3.13	-3.06	2.30E-03	4.64E-02
TRIB1	-0.23	8.02	-3.06	2.31E-03	4.65E-02
PGBD4	-0.11	7.15	-3.06	2.32E-03	4.66E-02
MPPED2	-0.42	8.41	-3.06	2.33E-03	4.67E-02
SLC15A5	-0.08	3.68	-3.06	2.33E-03	4.67E-02
PARD6B	-0.20	6.90	-3.05	2.34E-03	4.68E-02
ADGRG1	0.43	8.70	3.05	2.34E-03	4.68E-02
SMPD1	-0.13	7.02	-3.05	2.34E-03	4.68E-02
CADM3	-0.25	9.35	-3.05	2.34E-03	4.68E-02
CNGA3	0.50	6.31	3.05	2.35E-03	4.69E-02
COLQ	0.16	6.93	3.05	2.37E-03	4.71E-02
OR2L2	-0.51	5.92	-3.05	2.37E-03	4.71E-02
TDH	-0.08	4.72	-3.05	2.38E-03	4.72E-02
PNPLA8	-0.17	8.07	-3.05	2.38E-03	4.72E-02
VKORC1L1	-0.11	9.21	-3.05	2.38E-03	4.72E-02
PAX7	0.23	5.30	3.05	2.38E-03	4.72E-02
FLJ13224	0.08	5.51	3.05	2.40E-03	4.74E-02
UGT8	0.43	6.44	3.05	2.40E-03	4.74E-02
ANKRD16	0.11	7.26	3.05	2.40E-03	4.74E-02
ZC3H12B	0.32	5.02	3.05	2.40E-03	4.74E-02
SHKBP1	0.17	7.35	3.05	2.41E-03	4.74E-02
MTMR8	0.29	6.16	3.04	2.41E-03	4.74E-02
EXOSC5	0.14	8.12	3.04	2.41E-03	4.74E-02
SNORD56B	0.31	4.30	3.04	2.42E-03	4.74E-02
GAD1	0.58	5.38	3.04	2.42E-03	4.75E-02
HIST1H2BM	-0.42	10.94	-3.04	2.43E-03	4.75E-02
OPRD1	0.14	5.76	3.04	2.43E-03	4.75E-02
OCA2	0.36	5.77	3.04	2.43E-03	4.75E-02
FES	0.11	5.32	3.04	2.45E-03	4.77E-02
RTKN	0.22	6.94	3.04	2.45E-03	4.77E-02
UROD	0.12	8.00	3.04	2.45E-03	4.77E-02
PPP2R2B	-0.46	7.38	-3.04	2.46E-03	4.77E-02
DCAF16	0.15	9.37	3.04	2.46E-03	4.77E-02
FITM2	-0.13	7.18	-3.04	2.46E-03	4.77E-02
EPHX2	0.25	5.13	3.04	2.47E-03	4.77E-02
GJD2	0.34	7.62	3.04	2.47E-03	4.77E-02
DNAJC18	0.12	8.61	3.04	2.47E-03	4.77E-02
RNF2	-0.12	8.79	-3.04	2.47E-03	4.78E-02
GABRA1	-0.49	7.58	-3.04	2.48E-03	4.80E-02
WDR59	0.13	8.56	3.03	2.50E-03	4.82E-02

CHN2	-0.54	9.72	-3.03	2.50E-03	4.82E-02
OPTC	0.17	5.02	3.03	2.50E-03	4.82E-02
FNBP1	-0.25	9.36	-3.03	2.50E-03	4.82E-02
AXIN2	0.27	7.69	3.03	2.51E-03	4.83E-02
MATK	-0.16	5.61	-3.03	2.51E-03	4.83E-02
HECTD4	-0.17	9.38	-3.03	2.52E-03	4.83E-02
BDH2	0.35	5.96	3.03	2.52E-03	4.83E-02
TTLL11	-0.13	6.80	-3.03	2.53E-03	4.83E-02
FCGRT	0.13	7.48	3.03	2.53E-03	4.83E-02
BTBD6	-0.12	6.88	-3.03	2.53E-03	4.83E-02
TNFRSF19	0.44	6.16	3.03	2.54E-03	4.83E-02
GOLPH3L	0.14	8.25	3.03	2.54E-03	4.83E-02
TTC29	-0.33	4.01	-3.03	2.54E-03	4.83E-02
ASMTL	0.21	5.94	3.03	2.55E-03	4.83E-02
DGKD	-0.15	8.39	-3.03	2.55E-03	4.83E-02
WBP11P1	-0.10	5.75	-3.03	2.55E-03	4.83E-02
GOLM1	0.17	10.10	3.03	2.55E-03	4.83E-02
GPLD1	-0.27	6.98	-3.03	2.55E-03	4.83E-02
WDR62	-0.17	6.48	-3.03	2.55E-03	4.83E-02
SOWAHA	-0.10	5.51	-3.03	2.56E-03	4.83E-02
ITPRIP	0.22	6.23	3.03	2.56E-03	4.83E-02
ZNF449	0.11	6.38	3.03	2.56E-03	4.83E-02
COL9A1	-0.13	5.48	-3.03	2.57E-03	4.83E-02
OR52B4	-0.09	4.78	-3.02	2.58E-03	4.85E-02
ZNF671	-0.22	7.14	-3.02	2.58E-03	4.85E-02
PITPNM2	0.22	6.95	3.02	2.58E-03	4.85E-02
STRA6	0.24	5.36	3.02	2.60E-03	4.87E-02
THOC5	0.14	8.83	3.02	2.60E-03	4.87E-02
ZBTB34	-0.14	7.80	-3.02	2.60E-03	4.87E-02
EFTUD2	0.10	9.47	3.02	2.61E-03	4.88E-02
ETV6	0.30	8.24	3.02	2.61E-03	4.88E-02
BRWD3	-0.10	8.78	-3.02	2.61E-03	4.88E-02
IBTK	-0.14	8.55	-3.02	2.62E-03	4.88E-02
CADPS2	-0.52	7.02	-3.02	2.62E-03	4.88E-02
LAP3	0.20	7.25	3.02	2.62E-03	4.88E-02
SST	-0.49	5.33	-3.02	2.63E-03	4.89E-02
OR51B6	-0.10	3.68	-3.02	2.63E-03	4.89E-02
PACSIN2	0.20	8.41	3.02	2.64E-03	4.89E-02
LINC01551	-0.41	5.16	-3.02	2.64E-03	4.89E-02
NDUFV2	0.08	8.32	3.02	2.65E-03	4.89E-02
ZNF597	-0.25	7.23	-3.02	2.65E-03	4.89E-02
DGKE	-0.27	6.81	-3.02	2.65E-03	4.89E-02
SRBD1	-0.13	8.22	-3.02	2.65E-03	4.89E-02
CEND1	0.28	7.01	3.02	2.65E-03	4.89E-02
NADK2	0.13	7.88	3.02	2.65E-03	4.89E-02
SLC7A8	0.31	7.50	3.01	2.69E-03	4.95E-02
SOX13	0.29	6.21	3.01	2.69E-03	4.95E-02
SERPINI1	-0.35	5.24	-3.01	2.70E-03	4.96E-02
PODNL1	0.19	5.65	3.01	2.72E-03	4.99E-02
NPEPPS	-0.13	9.91	-3.01	2.72E-03	4.99E-02
POLH	-0.11	8.38	-3.01	2.73E-03	5.00E-02
RSPH1	0.13	4.89	3.01	2.73E-03	5.00E-02

logFC: fold change in expression on $\log_2$ scale; AveExpr: average expression of a gene across all samples; t: t-statistics from regularized t-test; FDR: false discovery rate.

Table S2. GO Biological processes enriched in TYA in the entire dataset

ID	Name	P-value	FDR	Count in Query List	Count in Genome
GO:0007389	pattern specification process	2.84E-09	1.60E-05	39	506
GO:0048706	embryonic skeletal system development	4.72E-08	1.33E-04	18	143
GO:0048704	embryonic skeletal system morphogenesis	1.68E-07	2.75E-04	15	108
GO:0003002	regionalization	2.31E-07	2.75E-04	30	392
GO:0048568	embryonic organ development	2.43E-07	2.75E-04	36	527
GO:0007224	smoothened signaling pathway	3.20E-07	3.02E-04	18	162
GO:0048562	embryonic organ morphogenesis	5.38E-07	4.34E-04	27	343
GO:0009887	animal organ morphogenesis	9.89E-07	6.73E-04	63	1263
GO:0003007	heart morphogenesis	1.07E-06	6.73E-04	24	292
GO:0009792	embryo development ending in birth or egg hatching	1.73E-06	8.99E-04	47	850
GO:0007507	heart development	1.85E-06	8.99E-04	41	698
GO:0043009	chordate embryonic development	1.91E-06	8.99E-04	46	827
GO:0051216	cartilage development	2.40E-06	1.04E-03	20	224
GO:0001501	skeletal system development	2.61E-06	1.05E-03	36	583
GO:0030178	negative regulation of Wnt signaling pathway	3.15E-06	1.19E-03	20	228
GO:0016055	Wnt signaling pathway	4.66E-06	1.50E-03	34	549
GO:0048598	embryonic morphogenesis	4.99E-06	1.50E-03	40	701
GO:0044782	cilium organization	5.02E-06	1.50E-03	30	455
GO:0198738	cell-cell signaling by wnt	5.04E-06	1.50E-03	34	551
GO:0048705	skeletal system morphogenesis	5.84E-06	1.65E-03	21	258
GO:0003206	cardiac chamber morphogenesis	6.27E-06	1.69E-03	15	143
GO:0030111	regulation of Wnt signaling pathway	7.43E-06	1.91E-03	27	394
GO:0060173	limb development	1.17E-05	2.76E-03	18	208
GO:0048736	appendage development	1.17E-05	2.76E-03	18	208
GO:0007018	microtubule-based movement	1.34E-05	3.03E-03	39	705
GO:0001578	microtubule bundle formation	1.61E-05	3.51E-03	29	459
GO:0042073	intraciliary transport	1.82E-05	3.82E-03	28	438
GO:0035567	non-canonical Wnt signaling pathway	2.10E-05	4.23E-03	15	158
GO:0009790	embryo development	2.85E-05	5.22E-03	60	1316
GO:0061512	protein localization to cilium	2.96E-05	5.22E-03	28	450
GO:0003231	cardiac ventricle development	3.01E-05	5.22E-03	14	144
GO:0007368	determination of left/right symmetry	3.26E-05	5.22E-03	14	145
GO:0060070	canonical Wnt signaling pathway	3.38E-05	5.22E-03	24	358
GO:0060271	cilium assembly	3.51E-05	5.22E-03	27	430
GO:1905349	ciliary transition zone assembly	3.51E-05	5.22E-03	27	430
GO:0035735	intraciliary transport involved in cilium assembly	3.51E-05	5.22E-03	27	430

GO:0035082	axoneme assembly	3.51E-05	5.22E-03	27	430
GO:0097712	vesicle targeting, trans-Golgi to periciliary membrane compartment	3.51E-05	5.22E-03	27	430
GO:0099118	microtubule-based protein transport	3.75E-05	5.30E-03	28	456
GO:0098840	protein transport along microtubule	3.75E-05	5.30E-03	28	456
GO:0090090	negative regulation of canonical Wnt signaling pathway	4.94E-05	6.81E-03	16	190
GO:0003205	cardiac chamber development	5.96E-05	8.02E-03	16	193
GO:0060972	left/right pattern formation	6.23E-05	8.18E-03	6	27
GO:0009855	determination of bilateral symmetry	6.79E-05	8.73E-03	14	155
GO:0009799	specification of symmetry	7.29E-05	9.15E-03	14	156
GO:0048199	vesicle targeting, to, from or within Golgi	7.58E-05	9.31E-03	29	500
GO:0048729	tissue morphogenesis	7.79E-05	9.37E-03	43	874
GO:0060415	muscle tissue morphogenesis	8.32E-05	9.79E-03	10	85
GO:0060828	regulation of canonical Wnt signaling pathway	8.52E-05	9.83E-03	21	309
GO:0072359	circulatory system development	8.83E-05	9.98E-03	59	1340
GO:0002009	morphogenesis of an epithelium	1.16E-04	1.26E-02	35	670
GO:2000050	regulation of non-canonical Wnt signaling pathway	1.17E-04	1.26E-02	6	30
GO:0009063	cellular amino acid catabolic process	1.18E-04	1.26E-02	11	106
GO:0060537	muscle tissue development	1.21E-04	1.26E-02	28	488
GO:0099111	microtubule-based transport	1.23E-04	1.27E-02	31	566
GO:0014706	striated muscle tissue development	1.26E-04	1.27E-02	27	464
GO:1905114	cell surface receptor signaling pathway involved in cell-cell signaling	1.38E-04	1.37E-02	35	676
GO:0003341	cilium movement	1.44E-04	1.38E-02	16	208
GO:0006903	vesicle targeting	1.44E-04	1.38E-02	29	519
GO:0007517	muscle organ development	1.50E-04	1.41E-02	24	394
GO:0048644	muscle organ morphogenesis	1.78E-04	1.65E-02	10	93
GO:0010970	transport along microtubule	1.81E-04	1.65E-02	30	552
GO:0001756	somitogenesis	1.97E-04	1.77E-02	9	77
GO:0061448	connective tissue development	2.10E-04	1.86E-02	20	306
GO:0048645	animal organ formation	2.18E-04	1.89E-02	9	78
GO:0009952	anterior/posterior pattern specification	2.31E-04	1.95E-02	17	239
GO:0061053	somite development	2.31E-04	1.95E-02	10	96
GO:0003208	cardiac ventricle morphogenesis	2.64E-04	2.20E-02	9	80
GO:0008589	regulation of smoothened signaling pathway	2.74E-04	2.24E-02	10	98
GO:0051650	establishment of vesicle localization	3.70E-04	2.99E-02	32	630
GO:0060350	endochondral bone morphogenesis	3.83E-04	3.05E-02	8	67
GO:0048738	cardiac muscle tissue development	4.40E-04	3.46E-02	18	276
GO:0007223	Wnt signaling pathway, calcium modulating pathway	4.54E-04	3.51E-02	6	38
GO:0030705	cytoskeleton-dependent intracellular transport	4.93E-04	3.76E-02	30	586
GO:0060351	cartilage development involved in endochondral bone morphogenesis	5.24E-04	3.92E-02	6	39
GO:0048565	digestive tract development	5.27E-04	3.92E-02	12	146

GO:0007267	cell-cell signaling	5.57E-04	4.09E-02	76	1971
GO:0051648	vesicle localization	5.99E-04	4.18E-02	32	648
GO:0060971	embryonic heart tube left/right pattern formation	5.99E-04	4.18E-02	3	7
GO:0014807	regulation of somitogenesis	5.99E-04	4.18E-02	3	7
GO:0006552	leucine catabolic process	5.99E-04	4.18E-02	3	7
GO:0060349	bone morphogenesis	6.92E-04	4.77E-02	10	110
GO:0006540	glutamate decarboxylation to succinate	7.22E-04	4.91E-02	2	2
GO:0035282	segmentation	7.43E-04	4.95E-02	10	111
GO:0048546	digestive tract morphogenesis	7.44E-04	4.95E-02	7	57

Table S3. GO Biological processes enriched in TYA of SHH tumours by GSEA

NAME	SIZE	ES	NES	FDR
GO_AXONEME_ASSEMBLY	63	0.78	2.42	0.00E+00
GO_CILIUM_MOVEMENT	135	0.67	2.30	0.00E+00
GO_MICROTUBULE_BUNDLE_FORMATION	93	0.68	2.23	0.00E+00
GO_CILIUM_ORGANIZATION	347	0.60	2.23	0.00E+00
GO_CILIUM_OR_FLAGELLUM_DEPENDENT_CELL_MOTILITY	109	0.66	2.21	0.00E+00
GO_RIBOSOME_BIOGENESIS	284	0.59	2.17	9.03E-05
GO_INTRACILIARY_TRANSPORT	50	0.71	2.15	7.74E-05
GO_PROTEIN_LOCALIZATION_TO_CILIUM	51	0.71	2.14	6.77E-05
GO_RRNA_MODIFICATION	36	0.75	2.13	6.02E-05
GO_NCRNA_PROCESSING	358	0.56	2.12	5.42E-05
GO_AXONEMAL_DYNEIN_COMPLEX_ASSEMBLY	28	0.77	2.12	4.93E-05
GO_EXTRACELLULAR_TRANSPORT	26	0.79	2.09	4.52E-05
GO_RRNA_METABOLIC_PROCESS	221	0.57	2.07	1.27E-04
GO_RIBONUCLEOPROTEIN_COMPLEX_BIOGENESIS	417	0.54	2.06	1.58E-04
GO_MITOCHONDRIAL_GENE_EXPRESSION	157	0.58	2.05	1.47E-04
GO_NCRNA_METABOLIC_PROCESS	429	0.54	2.05	1.73E-04
GO_MICROTUBULE_BASED_MOVEMENT	333	0.55	2.04	1.63E-04
GO_NON_MOTILE_CILIUM_ASSEMBLY	49	0.70	2.03	2.15E-04
GO_RNA_MODIFICATION	153	0.58	2.02	3.19E-04
GO_SPECIFICATION_OF_SYMMETRY	126	0.57	1.99	7.47E-04
GO_TRNA_PROCESSING	119	0.58	1.99	7.38E-04
GO_SPERM_MOTILITY	84	0.61	1.99	7.80E-04
GO_INTRACILIARY_TRANSPORT_INVOLVED_IN_CILIUM_ASSEMBLY	40	0.68	1.97	1.13E-03
GO_MITOCHONDRIAL_TRANSLATION	130	0.58	1.97	1.08E-03
GO_RRNA_METHYLATION	24	0.76	1.97	1.04E-03
GO_TRANSLATIONAL_TERMINATION	100	0.59	1.96	1.19E-03
GO_PROTEIN_TRANSPORT_ALONG_MICROTUBULE	64	0.62	1.95	1.43E-03
GO_TRNA_METABOLIC_PROCESS	157	0.55	1.94	1.90E-03
GO_MATURATION_OF_SSU_RRNA	47	0.65	1.92	2.73E-03
GO_MOTILE_CILIUM_ASSEMBLY	20	0.77	1.92	2.88E-03
GO_TRNA_MODIFICATION	83	0.59	1.92	2.89E-03
GO_MICROTUBULE_BASED_TRANSPORT	171	0.53	1.91	3.27E-03

GO_MATURATION_OF_SSU_RRNA_FROM_TRICISTRONIC_RRNA_TRANSCRIPT_SSU_RRNA_5_8S_RRNA_LSU_RRNA	34	0.67	1.89	5.37E-03
GO_CLEAVAGE_INVOLVED_IN_RRNA_PROCESSING	27	0.70	1.89	5.26E-03
GO_MITOCHONDRIAL_TRANSLATIONAL_TERMINATION	86	0.58	1.87	6.53E-03
GO_VENTRICULAR_SYSTEM_DEVELOPMENT	29	0.69	1.86	8.07E-03
GO_RIBOSOMAL_SMALL_SUBUNIT_BIOGENESIS	68	0.59	1.85	9.17E-03
GO_RIBOSOMAL_LARGE_SUBUNIT_BIOGENESIS	69	0.58	1.84	1.12E-02
GO_SPLICEOSOMAL_SNRNP_ASSEMBLY	46	0.62	1.84	1.11E-02
GO_MATURATION_OF_5_8S_RRNA_FROM_TRICISTRONIC_RRNA_TRANSCRIPT_SSU_RRNA_5_8S_RRNA_LSU_RRNA	24	0.70	1.82	1.33E-02
GO_RNA_METHYLATION	76	0.57	1.79	2.47E-02
GO_PSEUDOURIDINE_SYNTHESIS	18	0.73	1.77	3.20E-02
GO_SPERM_AXONEME_ASSEMBLY	16	0.75	1.76	3.65E-02
GO_PREASSEMBLY_OF_GPI_ANCHOR_IN_ER_MEMBRANE	16	0.74	1.75	4.03E-02
GO_EMBRYONIC_SKELETAL_SYSTEM_MORPHOGENESIS	95	0.53	1.75	3.97E-02
GO_SMOOTHENED_SIGNALING_PATHWAY	136	0.51	1.75	4.10E-02
GO_MATURATION_OF_LSU_RRNA	28	0.65	1.74	4.35E-02

Table S4. List of DMPs that are common across three MB subgroups

				SHH		Group 3		Group 4	
Probe	Chr	Position	Gene	delta beta	FDR	delta beta	FDR	delta beta	FDR
cg03522107	1	16553329		0.22	1.11E-03	0.33	1.45E-02	0.10	8.87E-03
cg14429204	1	16553327		0.22	3.04E-03	0.33	1.89E-02	0.09	8.72E-03
cg23279117	1	111813509		0.17	1.41E-02	0.19	4.26E-02	0.07	2.56E-02
cg24851854	1	59042275	TACSTD2	0.26	3.45E-03	0.36	4.76E-02	0.27	3.68E-07
cg07537370	2	27531124	UCN	0.23	4.77E-04	0.15	4.49E-02	0.02	5.92E-03
cg20438306	4	41747092	PHOX2B	0.23	4.43E-03	0.31	4.09E-02	0.19	4.31E-03
cg16105676	6	108497424	NR2E1	0.29	7.72E-04	0.26	4.08E-02	0.14	3.74E-03
cg05809668	7	35301188		0.37	4.54E-04	0.37	4.18E-02	0.18	4.34E-02
cg18177414	7	149389929		0.32	4.51E-04	0.31	2.82E-02	0.28	1.11E-05
cg23865240	7	27134109	HOXA1	0.31	2.02E-04	0.29	4.33E-02	0.20	2.13E-04
cg07262635	10	18689378	CACNB2	0.20	8.33E-03	0.09	7.02E-03	0.06	2.83E-02
cg12289251	10	18689471	CACNB2	0.19	4.66E-02	0.11	1.84E-02	0.10	2.49E-02
cg09290705	15	60285508		0.23	5.53E-03	0.24	3.02E-02	0.16	4.52E-02

Table S5. List of DMPs between TYA and other age groups in SHH tumours

ProbeID	CHR	MAPINFO	Gene	delta Beta	P_Value	FDR
cg01740424	20	3758584	SPEF1	0.32	6.96E-11	1.44E-05
cg24807850	1	3165391	PRDM16	0.29	1.33E-10	1.44E-05
cg05921905	7	27142527	HOXA2	0.35	1.35E-10	1.44E-05
cg04452311	2	176936377		0.29	3.48E-10	2.45E-05
cg02979457	7	27142547	HOXA2	0.32	6.10E-10	2.45E-05
cg06786372	7	27141388	HOXA2	0.34	7.64E-10	2.45E-05
cg04593780	16	3150150		0.35	7.80E-10	2.45E-05
cg21119525	1	2136847	C1orf86	0.31	8.45E-10	2.45E-05
cg04737131	7	27142708	HOXA2	0.34	8.67E-10	2.45E-05
cg13661519	7	27141088	HOXA2	0.33	8.87E-10	2.45E-05

cg04187935	4	571090		0.26	8.88E-10	2.45E-05
cg02803819	7	27141067	HOXA2	0.37	9.78E-10	2.45E-05
cg06769202	7	27142535	HOXA2	0.31	9.94E-10	2.45E-05
cg07522913	7	27150403	HOXA3	0.35	1.20E-09	2.71E-05
cg16296290	1	2136845	C1orf86	0.36	1.27E-09	2.71E-05
cg21464565	7	27141139	HOXA2	0.31	1.53E-09	2.80E-05
cg25598840	7	27142618	HOXA2	0.32	1.53E-09	2.80E-05
cg03010761	12	104608278	TXNRD1	-0.21	1.77E-09	2.80E-05
cg23217283	6	5039003		-0.21	1.85E-09	2.80E-05
cg17800708	7	27148204	HOXA3	0.31	1.94E-09	2.80E-05
cg01429449	2	176936397		0.30	1.99E-09	2.80E-05
cg08955995	19	42503412	GRIK5	0.39	2.00E-09	2.80E-05
cg04599965	4	571042		0.36	2.36E-09	3.15E-05
cg04046931	8	142452416	FLJ43860	-0.35	2.57E-09	3.30E-05
cg06520675	10	102996310	FLJ41350	0.39	3.16E-09	3.90E-05
cg20141652	19	19221574	SLC25A42	0.29	3.59E-09	4.14E-05
cg00845598	8	142452378	FLJ43860	-0.33	3.61E-09	4.14E-05
cg22943986	7	27142700	HOXA2	0.34	4.26E-09	4.61E-05
cg03763508	7	27140942	HOXA2	0.35	4.42E-09	4.61E-05
cg25124406	12	89744662	DUSP6	0.40	4.62E-09	4.61E-05
cg17353412	7	27141774	HOXA2	0.30	4.69E-09	4.61E-05
cg02693607	7	27150598	HOXA3	0.38	4.73E-09	4.61E-05
cg19048532	7	27148002	HOXA3	0.31	4.89E-09	4.62E-05
cg12475590	13	21288062	IL17D	-0.26	5.76E-09	5.16E-05
cg25123566	12	47610734	FAM113B	-0.28	6.03E-09	5.16E-05
cg19585433	12	133658083	ZNF140	-0.23	6.22E-09	5.16E-05
cg20670361	1	25258679	RUNX3	0.23	6.74E-09	5.16E-05
cg04092231	11	122852412	BSX	0.20	6.78E-09	5.16E-05
cg08544331	1	25246922	RUNX3	-0.24	6.96E-09	5.16E-05
cg26297530	19	7680259	KIAA1543	-0.30	7.14E-09	5.16E-05
cg00740368	19	51232007		0.31	7.27E-09	5.16E-05
cg12810626	1	160764664	LY9	-0.33	7.31E-09	5.16E-05
cg05030680	4	111542366	PITX2	0.24	7.39E-09	5.16E-05
cg10777495	19	7680231	KIAA1543	-0.36	7.67E-09	5.16E-05
cg02595575	19	19221316	SLC25A42	0.33	7.69E-09	5.16E-05
cg02078690	11	122852454	BSX	0.26	7.72E-09	5.16E-05
cg04481096	7	27142100	HOXA2	0.35	7.98E-09	5.17E-05
cg09323727	2	176936436		0.40	8.05E-09	5.17E-05
cg03053579	10	102987483	LBX1	0.39	8.32E-09	5.22E-05
cg13366863	5	177505451		-0.22	8.45E-09	5.22E-05
cg20087093	7	27142799	HOXA2	0.30	8.97E-09	5.33E-05
cg04377618	20	25128211	LOC284798	-0.23	9.87E-09	5.46E-05
cg03743720	2	241460002	ANKMY1	0.29	1.00E-08	5.46E-05
cg20747380	7	27142275	HOXA2	0.29	1.00E-08	5.46E-05
cg04965934	2	73151201	EMX1	0.27	1.01E-08	5.46E-05
cg09144964	7	27150262	HOXA3	0.27	1.08E-08	5.69E-05
cg06806862	3	184934396	EHHADH	-0.29	1.10E-08	5.70E-05
cg04524477	17	41363597	NBR1	0.34	1.13E-08	5.78E-05
cg19094872	2	242065898	PASK	-0.22	1.16E-08	5.80E-05
cg26746936	19	42503392	GRIK5	0.34	1.18E-08	5.82E-05
cg00459623	2	176936581		0.41	1.20E-08	5.82E-05
cg16909733	2	241459847	ANKMY1	0.32	1.29E-08	6.17E-05
cg05320183	20	3758973	SPEF1	0.30	1.35E-08	6.34E-05
cg07050616	10	102996197	FLJ41350	0.37	1.36E-08	6.34E-05
cg10997718	2	162275690	TBR1	0.33	1.38E-08	6.34E-05
cg06161964	5	1884513		0.24	1.42E-08	6.40E-05
cg23865994	20	3758499	SPEF1	0.36	1.43E-08	6.40E-05
cg03430526	8	27942055	C8orf80	-0.28	1.56E-08	6.81E-05
cg13112154	10	102996565	FLJ41350	0.35	1.57E-08	6.81E-05
cg07515422	19	40993713	SPTBN4	0.33	1.72E-08	7.22E-05
cg00333483	17	79504180		0.32	1.73E-08	7.22E-05
cg04630448	7	23373488	IGF2BP3	-0.27	1.77E-08	7.30E-05
cg13718729	9	140056619	GRIN1	0.37	1.85E-08	7.49E-05
cg10141717	6	30099252		-0.27	1.87E-08	7.49E-05
cg02367696	10	102996464	FLJ41350	0.37	1.98E-08	7.80E-05
cg03265671	1	156616554	BCAN	0.28	2.04E-08	7.80E-05

cg11720896	17	29914487		0.25	2.06E-08	7.80E-05
cg11342106	11	73020351	ARHGEF17	0.30	2.06E-08	7.80E-05
cg10665321	19	19221392	SLC25A42	0.35	2.07E-08	7.80E-05
cg22316269	4	571371		0.31	2.22E-08	8.01E-05
cg07996594	1	25257505	RUNX3	0.36	2.24E-08	8.01E-05
cg00018128	2	162275746	TBR1	0.37	2.25E-08	8.01E-05
cg02008651	14	29247148	C14orf23	0.33	2.25E-08	8.01E-05
cg17485141	2	42566556		-0.31	2.27E-08	8.01E-05
cg27314569	11	122852461	BSX	0.23	2.29E-08	8.01E-05
cg02498311	11	73020358	ARHGEF17	0.31	2.29E-08	8.01E-05
cg15508809	14	29231309		0.35	2.34E-08	8.07E-05
cg04221877	1	25257515	RUNX3	0.29	2.38E-08	8.14E-05
cg23442209	10	102995987	FLJ41350	0.32	2.41E-08	8.15E-05
cg09871315	7	27142682	HOXA2	0.31	2.44E-08	8.15E-05
cg15731655	12	54357604	HOTAIR	0.35	2.59E-08	8.45E-05
cg27359374	6	158375774		-0.29	2.64E-08	8.45E-05
cg13903070	1	61456527		-0.31	2.66E-08	8.45E-05
cg11051752	6	158375814		-0.24	2.71E-08	8.45E-05
cg05969808	10	102978788		0.30	2.73E-08	8.45E-05
cg26702958	10	102996254	FLJ41350	0.36	2.74E-08	8.45E-05
cg14036830	19	42503207	GRIK5	0.39	2.82E-08	8.56E-05
cg01357135	3	160475128	PPM1L	0.25	2.87E-08	8.56E-05
cg26069745	7	27142200	HOXA2	0.28	2.88E-08	8.56E-05
cg13365524	6	100902093	SIM1	0.36	2.88E-08	8.56E-05
cg24717061	10	102978443		0.23	2.97E-08	8.63E-05
cg12821539	13	100642775		0.34	3.01E-08	8.63E-05
cg19107469	20	55200445		0.26	3.06E-08	8.63E-05
cg23922454	12	113910545	LHX5	0.28	3.06E-08	8.63E-05
cg13298153	7	5123143		-0.32	3.11E-08	8.69E-05
cg24394172	4	46995203	GABRA4	0.37	3.20E-08	8.83E-05
cg09923978	11	58207761	OR5B12	-0.31	3.29E-08	8.83E-05
cg02470874	3	160475336	PPM1L	0.25	3.30E-08	8.83E-05
cg27187375	1	155912569	RXFP4	-0.23	3.32E-08	8.83E-05
cg04074066	9	139565154	EGFL7	-0.26	3.32E-08	8.83E-05
cg26997880	6	32055370	TNXB	0.34	3.33E-08	8.83E-05
cg23206851	7	27143046	HOXA2	0.28	3.42E-08	8.99E-05
cg09169953	4	111555102	PITX2	0.33	3.48E-08	9.10E-05
cg07201017	10	102996571	FLJ41350	0.34	3.53E-08	9.10E-05
cg05574272	5	33938213	RXFP3	0.29	3.54E-08	9.10E-05
cg11538641	6	100902258	SIM1	0.35	3.59E-08	9.16E-05
cg05072470	13	100643858		0.34	3.76E-08	9.26E-05
cg27156584	11	58207784	OR5B12	-0.32	3.79E-08	9.26E-05
cg21873674	3	13689339		-0.24	3.82E-08	9.26E-05
cg00606841	21	40757691	WRB	-0.21	3.82E-08	9.26E-05
cg20234976	6	100895907	SIM1	0.30	3.83E-08	9.26E-05
cg22132509	3	160475102	PPM1L	0.24	3.89E-08	9.29E-05
cg22241820	15	68122574	LBXCOR1	0.23	3.90E-08	9.29E-05
cg04056576	3	160475090	PPM1L	0.27	3.95E-08	9.29E-05
cg06476685	2	241459638	ANKMY1	0.32	3.98E-08	9.29E-05
cg08074534	6	100895143	SIM1	0.37	3.99E-08	9.29E-05
cg19598584	7	27142810	HOXA2	0.30	4.43E-08	1.01E-04
cg09173378	1	196370245	KCNT2	-0.28	4.45E-08	1.01E-04
cg07917609	5	33938225	RXFP3	0.31	4.48E-08	1.01E-04
cg15183092	7	150710447	ATG9B	0.28	4.52E-08	1.01E-04
cg08377924	17	41363628	NBR1	0.37	4.66E-08	1.03E-04
cg15014975	1	25257547	RUNX3	0.36	4.81E-08	1.06E-04
cg11823230	6	32055385	TNXB	0.33	4.83E-08	1.06E-04
cg04804604	7	156140609		-0.35	4.92E-08	1.06E-04
cg09864457	19	42502731	GRIK5	0.33	4.92E-08	1.06E-04
cg14717557	6	100895618	SIM1	0.39	4.95E-08	1.06E-04
cg11100933	5	1174646		0.23	4.96E-08	1.06E-04
cg26684131	6	32410365	HLA-DRA	-0.28	5.10E-08	1.08E-04
cg07080050	12	54413345	HOXC4	0.28	5.18E-08	1.08E-04
cg11174855	10	134598352	NKX6-2	0.39	5.19E-08	1.08E-04
cg07704627	17	77777173		0.25	5.20E-08	1.08E-04
cg03151469	2	176936091		0.22	5.36E-08	1.10E-04

cg21233003	9	140057464	GRIN1	0.38	5.40E-08	1.10E-04
cg24205914	10	62761575	RHOBTB1	0.26	5.43E-08	1.10E-04
cg02179652	11	122852429	BSX	0.21	5.54E-08	1.12E-04
cg18004701	7	157481628	PTPRN2	0.26	5.65E-08	1.13E-04
cg16964025	7	157482215	PTPRN2	0.32	5.71E-08	1.13E-04
cg12641240	8	638330	ERICH1	0.39	5.78E-08	1.14E-04
cg03221366	12	131943548		-0.37	5.92E-08	1.16E-04
cg07153966	7	27147590	HOXA3	0.32	5.96E-08	1.16E-04
cg02646021	2	223176846		0.31	6.12E-08	1.18E-04
cg07211212	20	55200146		0.23	6.30E-08	1.20E-04
cg06055873	7	27142204	HOXA2	0.29	6.40E-08	1.21E-04
cg05483184	6	32055393	TNXB	0.33	6.40E-08	1.21E-04
cg24469980	1	221064953		0.34	6.56E-08	1.23E-04
cg20208133	20	39311637		0.32	6.75E-08	1.26E-04
cg02124887	5	1174666		0.24	6.83E-08	1.26E-04
cg19019849	4	13533145		0.24	6.84E-08	1.26E-04
cg04132452	3	160475035	PPM1L	0.22	7.07E-08	1.28E-04
cg14362312	9	136294076	ADAMTS13	0.37	7.07E-08	1.28E-04
cg04649852	16	29229650		-0.24	7.08E-08	1.28E-04
cg22303045	6	100917022		0.33	7.18E-08	1.29E-04
cg04460816	19	19221572	SLC25A42	0.22	7.30E-08	1.29E-04
cg06622953	12	54357752	HOTAIR	0.25	7.33E-08	1.29E-04
cg05879418	5	54455955	CDC20B	0.39	7.41E-08	1.30E-04
cg01383274	2	7571241		0.30	7.53E-08	1.30E-04
cg05494604	13	23733862		0.39	7.60E-08	1.30E-04
cg05638359	1	190444387	FAM5C	0.30	7.60E-08	1.30E-04
cg18987335	2	177039862		0.25	7.62E-08	1.30E-04
cg08508814	5	54455701	CDC20B	0.37	7.73E-08	1.31E-04
cg16184803	4	111542444	PITX2	0.26	7.80E-08	1.32E-04
cg10440696	11	5809911	OR52N1	-0.31	7.82E-08	1.32E-04
cg10350492	5	33937673	RXFP3	0.33	7.91E-08	1.32E-04
cg05886546	17	40936498	WNK4	0.33	7.93E-08	1.32E-04
cg01242903	12	89744877	DUSP6	0.34	8.15E-08	1.35E-04
cg20134215	6	100441407	MCHR2	0.30	8.19E-08	1.35E-04
cg18875012	3	172165517	GHSR	0.29	8.27E-08	1.36E-04
cg16680547	1	14027567	PRDM2	0.27	8.44E-08	1.37E-04
cg19070088	4	190123606		0.32	8.48E-08	1.37E-04
cg21250721	9	140056227	GRIN1	0.38	8.70E-08	1.38E-04
cg23683674	11	73020562	ARHGEF17	0.33	8.77E-08	1.38E-04
cg06386307	6	100895211	SIM1	0.34	8.78E-08	1.38E-04
cg26248075	6	100895481	SIM1	0.37	8.81E-08	1.38E-04
cg02970384	1	229567560	ACTA1	0.34	8.85E-08	1.38E-04
cg00248242	2	240867059		-0.25	9.06E-08	1.41E-04
cg24624901	7	19184273		0.37	9.13E-08	1.41E-04
cg25399162	18	34824016	BRUNOL4	0.28	9.16E-08	1.41E-04
cg13240116	7	27147513	HOXA3	0.25	9.25E-08	1.41E-04
cg15435730	6	50680222	TFAP2D	0.28	9.45E-08	1.43E-04
cg02225599	7	27143252	HOXA2	0.21	9.60E-08	1.44E-04
cg27262041	11	19654032	NAV2	-0.25	9.62E-08	1.44E-04
cg00525823	18	11148510	FAM38B	0.30	9.89E-08	1.47E-04
cg05677724	12	131125565		-0.23	1.01E-07	1.50E-04
cg11201177	11	2961805		-0.23	1.04E-07	1.52E-04
cg11229513	8	70984559	PRDM14	0.36	1.04E-07	1.52E-04
cg03883784	19	7680105	KIAA1543	-0.32	1.04E-07	1.52E-04
cg00502190	1	156616560	BCAN	0.25	1.05E-07	1.52E-04
cg20054157	10	10837080	SFTA1P	-0.25	1.06E-07	1.54E-04
cg09434500	19	42502897	GRIK5	0.29	1.08E-07	1.56E-04
cg13892750	9	136293722	ADAMTS13	0.29	1.09E-07	1.56E-04
cg22570213	15	91497863	RCCD1	0.23	1.10E-07	1.57E-04
cg19072291	7	65617362	CRCP	-0.24	1.11E-07	1.57E-04
cg07546916	19	51231840		0.33	1.11E-07	1.57E-04
cg22374057	18	11148682	FAM38B	0.28	1.12E-07	1.57E-04
cg20557801	15	68121177	LBXCOR1	0.35	1.12E-07	1.57E-04
cg06615380	1	1072902		0.27	1.13E-07	1.57E-04
cg24104241	1	229567626	ACTA1	0.38	1.13E-07	1.57E-04
cg27087956	5	122430153	PRDM6	0.29	1.13E-07	1.57E-04

cg03830329	6	100897081	SIM1	0.32	1.16E-07	1.59E-04
cg08061745	19	51231557		0.27	1.21E-07	1.66E-04
cg09925572	12	51518419	TFCP2	-0.25	1.22E-07	1.66E-04
cg21460606	6	32055402	TNXB	0.38	1.23E-07	1.66E-04
cg07625430	19	18680126	C19orf50	-0.21	1.23E-07	1.66E-04
cg10477621	7	19184950	FERD3L	0.34	1.23E-07	1.66E-04
cg11141380	11	122852417	BSX	0.21	1.25E-07	1.68E-04
cg25394203	19	11784708	ZNF833	0.27	1.25E-07	1.68E-04
cg19535672	2	27530455	UCN	0.34	1.26E-07	1.68E-04
cg04041960	10	121301765	RGS10	0.36	1.28E-07	1.70E-04
cg04207084	7	157477846	PTPRN2	0.33	1.31E-07	1.73E-04
cg24880701	15	68121563	LBXCOR1	0.34	1.33E-07	1.74E-04
cg07188513	7	156807474		0.20	1.33E-07	1.74E-04
cg19240213	7	27163095	HOXA3	0.31	1.34E-07	1.74E-04
cg20168412	3	13324132		0.33	1.35E-07	1.75E-04
cg05380821	6	108491108	NR2E1	0.33	1.36E-07	1.75E-04
cg20842253	2	27529788	TRIM54	0.36	1.36E-07	1.75E-04
cg04778178	7	27162294	HOXA3	0.33	1.37E-07	1.76E-04
cg08212230	5	54455564	CDC20B	0.34	1.39E-07	1.76E-04
cg16932827	3	193988639		-0.24	1.39E-07	1.76E-04
cg20983112	11	75480246	DGAT2	0.28	1.39E-07	1.76E-04
cg14526718	2	176932840		0.34	1.40E-07	1.77E-04
cg04681600	14	38725536	CLEC14A	0.28	1.44E-07	1.80E-04
cg10812186	6	32055146	TNXB	0.37	1.44E-07	1.80E-04
cg27138293	17	79503701	FSCN2	0.32	1.44E-07	1.80E-04
cg26690915	6	30421218		0.23	1.44E-07	1.80E-04
cg04385765	7	5122887		-0.33	1.46E-07	1.81E-04
cg10567964	1	205312695	KLHDC8A	0.28	1.47E-07	1.82E-04
cg20952901	19	17403529	ABHD8	0.37	1.49E-07	1.83E-04
cg21652122	22	28837586	TTC28	-0.25	1.51E-07	1.84E-04
cg23969554	1	2979311	FLJ42875	0.29	1.52E-07	1.84E-04
cg05769889	12	89744701	DUSP6	0.37	1.52E-07	1.84E-04
cg18222083	17	41364007	TMEM106A	0.39	1.57E-07	1.90E-04
cg08786026	6	126068238		0.37	1.60E-07	1.92E-04
cg23162310	5	172199255	DUSP1	0.34	1.60E-07	1.92E-04
cg00073384	1	248343651	OR2M2	-0.29	1.63E-07	1.94E-04
cg16748008	7	27155002	HOXA3	0.25	1.64E-07	1.94E-04
cg14605874	6	100915395		0.34	1.68E-07	1.98E-04
cg15697476	6	32055474	TNXB	0.33	1.71E-07	1.98E-04
cg20745684	7	29923339	WIPF3	0.22	1.71E-07	1.98E-04
cg14065115	6	100916959		0.33	1.72E-07	1.98E-04
cg14530304	17	77777792		0.26	1.72E-07	1.98E-04
cg04436469	10	134598496	NKX6-2	0.32	1.73E-07	1.99E-04
cg26834680	1	111814208		0.27	1.77E-07	2.01E-04
cg12252135	17	79503877	FSCN2	0.35	1.77E-07	2.01E-04
cg23865240	7	27134109	HOXA1	0.31	1.78E-07	2.02E-04
cg15123573	5	176827082		0.29	1.79E-07	2.02E-04
cg02090762	17	79503859	FSCN2	0.38	1.83E-07	2.05E-04
cg06008356	16	1233872	CACNA1H	0.21	1.84E-07	2.06E-04
cg03236802	6	32055316	TNXB	0.31	1.84E-07	2.06E-04
cg02358323	11	44541718		-0.21	1.85E-07	2.06E-04
cg14934253	6	108436768		0.31	1.86E-07	2.06E-04
cg07707031	17	79504142	FSCN2	0.21	1.90E-07	2.10E-04
cg24055029	6	32055137	TNXB	0.37	1.93E-07	2.12E-04
cg02033258	5	131593261	PDLIM4	0.39	1.95E-07	2.14E-04
cg05509896	16	3063869	CLDN9	0.20	1.97E-07	2.15E-04
cg20287461	17	7339158	TMEM102	0.33	1.97E-07	2.15E-04
cg25527494	13	100641243		0.32	1.99E-07	2.16E-04
cg13572145	2	80529621	CTNNA2	0.30	2.01E-07	2.17E-04
cg24824178	5	141263222		0.33	2.02E-07	2.17E-04
cg25734913	5	172672390		0.33	2.03E-07	2.17E-04
cg09557462	8	26721736	ADRA1A	0.31	2.03E-07	2.17E-04
cg07247190	10	102979058		0.23	2.03E-07	2.17E-04
cg08195247	2	176933286		0.32	2.04E-07	2.17E-04
cg04204526	1	2980937	FLJ42875	0.22	2.04E-07	2.17E-04
cg13527872	9	90114037	DAPK1	0.27	2.11E-07	2.22E-04

cg24593272	10	102977637		0.29	2.12E-07	2.22E-04
cg26220673	1	221064700		0.31	2.12E-07	2.22E-04
cg24008544	17	41363899	TMEM106A	0.31	2.12E-07	2.22E-04
cg27525446	1	17019810	ESPNP	0.29	2.13E-07	2.22E-04
cg26314966	17	79466160		-0.26	2.14E-07	2.23E-04
cg21771200	19	11784761	ZNF833	0.31	2.17E-07	2.25E-04
cg08928408	3	149454106		-0.20	2.18E-07	2.26E-04
cg22638593	5	131593259	PDLIM4	0.35	2.21E-07	2.26E-04
cg20165545	1	3166599	PRDM16	0.24	2.21E-07	2.26E-04
cg18782604	6	100895050	SIM1	0.41	2.23E-07	2.26E-04
cg20028470	2	27530829	UCN	0.33	2.23E-07	2.26E-04
cg06987672	1	181453051	CACNA1E	0.29	2.23E-07	2.26E-04
cg09517106	1	198904195		0.33	2.25E-07	2.26E-04
cg07716052	11	32454748	WT1	0.29	2.26E-07	2.26E-04
cg06536868	2	27530884	UCN	0.36	2.26E-07	2.26E-04
cg07984365	16	739425	WDR24	-0.23	2.26E-07	2.26E-04
cg19427338	2	42566455		-0.28	2.27E-07	2.27E-04
cg04268813	2	42566404		-0.25	2.29E-07	2.28E-04
cg08098964	19	7680443	KIAA1543	-0.32	2.38E-07	2.35E-04
cg02076378	19	7677739	KIAA1543	-0.30	2.40E-07	2.37E-04
cg00899400	7	27154537	HOXA3	0.25	2.42E-07	2.38E-04
cg19378036	17	36105364	HNF1B	0.28	2.44E-07	2.39E-04
cg17689799	2	27529540	TRIM54	0.33	2.49E-07	2.44E-04
cg18501555	4	111554966	PITX2	0.33	2.54E-07	2.46E-04
cg04902729	1	181452707	CACNA1E	0.34	2.54E-07	2.46E-04
cg06486618	19	524262		-0.25	2.55E-07	2.46E-04
cg06332173	17	77777734		0.31	2.56E-07	2.46E-04
cg02963613	2	1425319	TPO	-0.25	2.58E-07	2.48E-04
cg00448752	1	101775370		0.29	2.59E-07	2.48E-04
cg20691205	13	36919674	SPG20	0.35	2.65E-07	2.51E-04
cg17326555	1	119535693		0.33	2.67E-07	2.52E-04
cg06819445	5	172199278	DUSP1	0.27	2.69E-07	2.52E-04
cg15209566	19	11784774	ZNF833	0.30	2.69E-07	2.52E-04
cg02533423	7	27232150		0.29	2.73E-07	2.54E-04
cg01610231	4	111555314	PITX2	0.33	2.73E-07	2.54E-04
cg19335437	2	177039801		0.22	2.79E-07	2.57E-04
cg11805669	2	157178294		0.34	2.79E-07	2.57E-04
cg02488129	16	3058364		0.22	2.88E-07	2.64E-04
cg10636745	1	101775181		0.35	2.90E-07	2.64E-04
cg15329277	6	42980035	MEA1	-0.21	2.90E-07	2.64E-04
cg11152263	7	569642		-0.27	2.92E-07	2.64E-04
cg21382382	4	174452961	NBLA00301	0.26	2.98E-07	2.68E-04
cg24940138	17	41363741	TMEM106A	0.34	2.99E-07	2.68E-04
cg11310863	2	144303521	ARHGAP15	-0.24	2.99E-07	2.68E-04
cg01499197	13	28544814	CDX2	0.27	3.00E-07	2.68E-04
cg01726775	12	7261710	LOC283314	0.23	3.01E-07	2.68E-04
cg10319053	7	27143370	HOXA2	0.21	3.07E-07	2.72E-04
cg05210501	7	27219344	HOXA10	0.22	3.09E-07	2.73E-04
cg06382559	10	102896640	TLX1	0.32	3.11E-07	2.74E-04
cg03538383	12	110399167	GIT2	-0.22	3.13E-07	2.74E-04
cg26608693	1	14027581	PRDM2	0.32	3.14E-07	2.74E-04
cg23949782	14	79452439	NRXN3	-0.24	3.14E-07	2.74E-04
cg13881637	11	64064727	KCNK4	0.22	3.18E-07	2.77E-04
cg07810884	19	16178099	TPM4	-0.27	3.26E-07	2.80E-04
cg22834542	11	88288530	GRM5	-0.27	3.29E-07	2.81E-04
cg19534021	19	16178091	TPM4	-0.25	3.29E-07	2.81E-04
cg09996288	6	32055517	TNXB	0.34	3.30E-07	2.81E-04
cg21769619	11	32454768	WT1	0.27	3.31E-07	2.81E-04
cg03515663	7	157478510	PTPRN2	0.26	3.36E-07	2.84E-04
cg22537474	20	10198659	SNAP25	0.23	3.37E-07	2.84E-04
cg24562669	7	97807699	LMTK2	-0.31	3.38E-07	2.85E-04
cg21468274	17	7339835	TMEM102	0.27	3.39E-07	2.85E-04
cg18185958	2	223176899		0.26	3.44E-07	2.88E-04
cg26922007	11	5080452	OR52E2	-0.31	3.46E-07	2.88E-04
cg19904265	1	3166712	PRDM16	0.21	3.48E-07	2.88E-04
cg14432910	15	68121931	LBXCOR1	0.29	3.49E-07	2.88E-04

cg00977978	7	139409421	HIPK2	-0.20	3.49E-07	2.88E-04
cg13206191	2	189745054		-0.23	3.49E-07	2.88E-04
cg16105870	2	131451075		0.21	3.51E-07	2.89E-04
cg11856810	2	155554961	KCNJ3	0.29	3.55E-07	2.91E-04
cg27557378	16	2286786	DNASE1L2	0.33	3.55E-07	2.91E-04
cg04528477	2	176932458		0.30	3.58E-07	2.92E-04
cg06354193	1	3183072	PRDM16	0.23	3.59E-07	2.92E-04
cg19909239	4	111555503	PITX2	0.27	3.60E-07	2.92E-04
cg19258665	7	157482508	PTPRN2	0.22	3.60E-07	2.92E-04
cg14355911	17	59473456		0.28	3.64E-07	2.94E-04
cg25108022	5	172199313	DUSP1	0.33	3.67E-07	2.94E-04
cg04370314	13	100643312		0.34	3.67E-07	2.94E-04
cg17924415	17	77777053		0.27	3.72E-07	2.97E-04
cg03049782	17	41363891	TMEM106A	0.28	3.73E-07	2.97E-04
cg16288089	7	97361408	TAC1	0.36	3.73E-07	2.97E-04
cg19807376	6	32055447	TNXB	0.33	3.79E-07	2.99E-04
cg11025705	8	145697880	KIFC2	0.25	3.79E-07	2.99E-04
cg24912415	20	33585435	MYH7B	0.23	3.80E-07	2.99E-04
cg19304088	4	111547790	PITX2	0.25	3.81E-07	2.99E-04
cg12434944	7	48031559	SUNC1	0.27	3.81E-07	2.99E-04
cg11694262	13	100644657		0.36	3.82E-07	2.99E-04
cg13662644	19	7677237	KIAA1543	-0.29	3.84E-07	2.99E-04
cg18046394	7	29924165	WIPF3	0.26	3.86E-07	2.99E-04
cg20471413	6	32055135	TNXB	0.34	3.86E-07	2.99E-04
cg24788897	2	164965721		-0.28	3.88E-07	2.99E-04
cg20406482	17	40333521	KCNH4	0.24	3.92E-07	3.01E-04
cg08589077	17	58217038		0.32	3.93E-07	3.01E-04
cg24817154	6	27858279	HIST1H3J	0.29	3.93E-07	3.01E-04
cg19617161	7	13788841		0.24	3.95E-07	3.01E-04
cg26327092	12	130469463		-0.26	3.96E-07	3.01E-04
cg03222323	17	59534903	TBX4	0.24	3.96E-07	3.01E-04
cg21723559	20	44048174	PIGT	-0.22	3.98E-07	3.02E-04
cg04482110	17	41364121	TMEM106A	0.30	4.01E-07	3.02E-04
cg00542880	9	97765043	C9orf3	-0.30	4.01E-07	3.02E-04
cg08118159	19	41055208	SPTBN4	0.35	4.03E-07	3.03E-04
cg14199723	8	145051795	PLEC1	0.28	4.04E-07	3.03E-04
cg10445449	13	100644420		0.37	4.08E-07	3.04E-04
cg09227150	10	102475578		0.26	4.10E-07	3.04E-04
cg15498134	1	25246854	RUNX3	-0.23	4.10E-07	3.04E-04
cg08308801	10	119867973	CASC2	-0.29	4.11E-07	3.05E-04
cg26297005	7	27162090	HOXA3	0.31	4.17E-07	3.08E-04
cg05452406	6	10881891	GCM2	0.34	4.22E-07	3.10E-04
cg00445443	7	27143478	HOXA2	0.24	4.25E-07	3.10E-04
cg00410600	8	145555942	SCRT1	0.27	4.25E-07	3.10E-04
cg04433035	17	36105064	HNF1B	0.20	4.25E-07	3.10E-04
cg17139210	1	115213243	DENND2C	0.30	4.27E-07	3.11E-04
cg13906069	1	115213259	DENND2C	0.30	4.31E-07	3.13E-04
cg23745839	11	20627025	SLC6A5	0.27	4.33E-07	3.14E-04
cg05651283	1	53098912	FAM159A	0.30	4.34E-07	3.14E-04
cg04556550	7	72982668		-0.23	4.36E-07	3.15E-04
cg21504064	17	41363502	NBR1	0.24	4.38E-07	3.16E-04
cg12659883	1	119543057		0.34	4.44E-07	3.19E-04
cg19817912	7	27162768	HOXA3	0.26	4.46E-07	3.20E-04
cg08488945	3	181422202	SOX2OT	0.27	4.61E-07	3.28E-04
cg19095920	4	174458819	NBLA00301	0.28	4.62E-07	3.28E-04
cg00642460	5	176827697	PFN3	0.33	4.63E-07	3.28E-04
cg23536473	7	19184555	FERD3L	0.33	4.63E-07	3.28E-04
cg26922678	6	30420981		0.29	4.64E-07	3.28E-04
cg03171924	1	25258332	RUNX3	0.28	4.68E-07	3.30E-04
cg10599985	22	27068733	MIAT	0.23	4.69E-07	3.30E-04
cg10558887	13	36919409	SPG20	0.32	4.75E-07	3.32E-04
cg19645221	14	38058284		0.28	4.75E-07	3.32E-04
cg02996142	1	53168724		0.29	4.77E-07	3.32E-04
cg00997079	2	80529990	CTNNA2	0.29	4.78E-07	3.32E-04
cg05581415	16	2286492	DNASE1L2	0.28	4.80E-07	3.32E-04
cg03294619	5	172661803	NKX2-5	0.30	4.83E-07	3.33E-04

cg13649103	9	137734338	COL5A1	-0.31	4.87E-07	3.34E-04
cg06327727	3	62354546		0.34	4.88E-07	3.34E-04
cg10838664	22	27068761	MIAT	0.30	4.89E-07	3.34E-04
cg04161236	11	5263432	HBBP1	-0.28	4.89E-07	3.34E-04
cg18629151	4	13539581		0.24	4.90E-07	3.34E-04
cg21378238	1	181452618	CACNA1E	0.24	4.92E-07	3.34E-04
cg17484993	2	164965724		-0.28	4.93E-07	3.34E-04
cg00796424	12	54365966	HOXC11	0.28	4.93E-07	3.34E-04
cg05152300	16	17458534	XYLT1	0.25	4.94E-07	3.34E-04
cg20523861	13	70682324	KLHL1	0.33	4.94E-07	3.34E-04
cg16406967	7	27155036	HOXA3	0.30	4.97E-07	3.36E-04
cg01558916	19	11804727		0.20	4.99E-07	3.36E-04
cg10703786	7	5123074		-0.20	5.06E-07	3.40E-04
cg19165854	15	79725126	KIAA1024	0.31	5.09E-07	3.41E-04
cg02740950	6	32055534	TNXB	0.30	5.10E-07	3.41E-04
cg05482942	10	102899285		0.35	5.11E-07	3.42E-04
cg03499808	11	73020459	ARHGEF17	0.35	5.13E-07	3.42E-04
cg27430293	16	89069935		-0.23	5.24E-07	3.47E-04
cg17243289	18	45458021	SMAD2	0.21	5.27E-07	3.47E-04
cg26510017	12	39299546	CPNE8	0.28	5.31E-07	3.48E-04
cg14363981	13	107186561	EFNB2	0.28	5.34E-07	3.48E-04
cg25529393	6	27858380	HIST1H3J	0.35	5.37E-07	3.49E-04
cg06166490	7	27143334	HOXA2	0.24	5.37E-07	3.49E-04
cg02227188	12	54396354	HOXC9	0.32	5.38E-07	3.49E-04
cg23674469	1	101775142		0.30	5.47E-07	3.53E-04
cg01776188	5	172664082		0.29	5.49E-07	3.53E-04
cg13851466	1	38219973	EPHA10	0.26	5.53E-07	3.55E-04
cg24006721	1	25258225	RUNX3	0.28	5.57E-07	3.56E-04
cg03546977	5	78985489	CMYA5	0.30	5.62E-07	3.58E-04
cg25287482	1	23884847	ID3	0.24	5.74E-07	3.63E-04
cg20866075	17	77777010		0.28	5.75E-07	3.63E-04
cg00087368	6	100896392	SIM1	0.27	5.76E-07	3.63E-04
cg22618215	3	62354489		0.22	5.76E-07	3.63E-04
cg15215612	19	51231736		0.36	5.77E-07	3.63E-04
cg26404422	11	128367010	ETS1	-0.22	5.80E-07	3.63E-04
cg08484671	19	41055297	SPTBN4	0.30	5.80E-07	3.63E-04
cg14789272	5	54456056	CDC20B	0.22	5.81E-07	3.63E-04
cg01144053	2	7571361		0.30	5.82E-07	3.63E-04
cg19432993	7	27143287	HOXA2	0.22	5.83E-07	3.63E-04
cg21406271	1	25258082	RUNX3	0.30	5.84E-07	3.63E-04
cg00326958	10	43892075	HNRNPF	0.31	5.85E-07	3.63E-04
cg01404317	13	36919819	SPG20	0.34	5.88E-07	3.65E-04
cg24100168	11	5667836	TRIM78P	-0.28	5.93E-07	3.65E-04
cg24035245	1	119535928		0.34	5.93E-07	3.65E-04
cg00184452	1	50887477	DMRTA2	0.21	5.93E-07	3.65E-04
cg04771100	8	38832335	HTRA4	0.31	5.94E-07	3.65E-04
cg09845293	10	7567980		-0.30	6.05E-07	3.70E-04
cg06724305	1	50887944	DMRTA2	0.22	6.05E-07	3.70E-04
cg14801038	6	134214307	TCF21	0.22	6.06E-07	3.70E-04
cg06338710	1	92946187	GFI1	0.28	6.07E-07	3.70E-04
cg17965019	6	27858545	HIST1H3J	0.37	6.07E-07	3.70E-04
cg18680977	7	27155039	HOXA3	0.30	6.10E-07	3.71E-04
cg00345344	16	88459714		-0.22	6.18E-07	3.74E-04
cg07135237	10	21808126	C10orf140	0.23	6.24E-07	3.77E-04
cg18178010	6	32055629	TNXB	0.30	6.29E-07	3.78E-04
cg15569451	20	44041324		-0.25	6.35E-07	3.80E-04
cg07847863	13	53419963	PCDH8	0.33	6.41E-07	3.82E-04
cg17744295	19	16178097	TPM4	-0.25	6.43E-07	3.82E-04
cg00014104	22	22220367	MAPK1	0.27	6.43E-07	3.82E-04
cg13655674	1	119522386	TBX15	0.34	6.46E-07	3.83E-04
cg27160241	19	10225927	EIF3G	-0.22	6.52E-07	3.85E-04
cg26590664	19	11784731	ZNF833	0.28	6.54E-07	3.85E-04
cg04027736	7	27143403	HOXA2	0.22	6.55E-07	3.85E-04
cg27052403	11	113185154	TTC12	0.31	6.55E-07	3.85E-04
cg13552867	1	160053689	KCNJ9	0.30	6.61E-07	3.88E-04
cg18771357	13	107186870	EFNB2	0.35	6.63E-07	3.89E-04

cg06324373	10	99734805	CRTAC1	0.30	6.68E-07	3.91E-04
cg13241663	17	33125534		-0.24	6.70E-07	3.91E-04
cg23483886	5	1476274	LPCAT1	0.23	6.70E-07	3.91E-04
cg22305258	17	4643367	CXCL16	0.29	6.77E-07	3.93E-04
cg16264616	12	54390824		0.21	6.79E-07	3.93E-04
cg15575683	1	156358612		0.24	6.82E-07	3.93E-04
cg02305687	5	33937182	RXFP3	0.27	6.82E-07	3.93E-04
cg14022022	9	140055728	GRIN1	0.30	6.83E-07	3.93E-04
cg17576736	13	112908450		-0.21	6.86E-07	3.93E-04
cg17760405	6	100915191		0.36	6.87E-07	3.93E-04
cg05581451	4	111553999	PITX2	0.22	6.87E-07	3.93E-04
cg19521279	7	27144595		0.22	6.87E-07	3.93E-04
cg15907392	12	34494852		-0.29	6.89E-07	3.93E-04
cg01250490	1	12251718	TNFRSF1B	-0.24	6.90E-07	3.93E-04
cg02567289	1	247805255		-0.32	6.93E-07	3.94E-04
cg23547759	19	15237807	ILVBL	-0.24	6.95E-07	3.94E-04
cg18921954	5	33938909		0.28	6.97E-07	3.94E-04
cg01053087	8	637909	ERICH1	0.29	7.09E-07	3.98E-04
cg04044664	5	132150117	ANKRD43	0.27	7.15E-07	4.01E-04
cg07269374	12	66176707	RPSAP52	-0.29	7.16E-07	4.01E-04
cg17346246	12	123214864	GPR81	-0.30	7.20E-07	4.01E-04
cg07872868	2	27530153	TRIM54	0.33	7.22E-07	4.01E-04
cg15509361	7	19183325		0.21	7.29E-07	4.04E-04
cg06071058	9	96716091	BARX1	0.24	7.30E-07	4.04E-04
cg15832180	19	7032924	MBD3L5	-0.22	7.31E-07	4.04E-04
cg13870990	18	28682408	DSC2	0.23	7.32E-07	4.04E-04
cg27183679	4	111562545		0.29	7.33E-07	4.04E-04
cg04159903	5	42949799		0.30	7.38E-07	4.05E-04
cg00980978	20	44098724	WFDC2	0.27	7.40E-07	4.05E-04
cg01302656	14	29254853	C14orf23	0.28	7.40E-07	4.05E-04
cg05459190	10	134110326	STK32C	-0.22	7.41E-07	4.05E-04
cg13603508	12	39299326	CPNE8	0.36	7.42E-07	4.05E-04
cg09403249	12	45444203	DBX2	0.31	7.43E-07	4.05E-04
cg15929573	12	49658188	TUBA1C	0.29	7.44E-07	4.05E-04
cg04351734	7	27155173	HOXA3	0.21	7.45E-07	4.05E-04
cg05974274	4	81123369	PRDM8	0.34	7.47E-07	4.05E-04
cg21239997	2	24398519	C2orf84	-0.23	7.51E-07	4.07E-04
cg05301866	2	162279964	TBR1	0.30	7.55E-07	4.07E-04
cg01217984	7	27143235	HOXA2	0.20	7.55E-07	4.07E-04
cg18008019	13	100641646		0.33	7.62E-07	4.11E-04
cg24463471	1	25257978	RUNX3	0.31	7.63E-07	4.11E-04
cg12053155	13	107186476	EFNB2	0.33	7.65E-07	4.11E-04
cg19537645	5	172199418	DUSP1	0.27	7.85E-07	4.18E-04
cg17001689	6	32055525	TNXB	0.29	7.85E-07	4.18E-04
cg00369438	19	41076542	SPTBN4	0.35	7.86E-07	4.18E-04
cg22614142	8	145698232	KIFC2	0.34	7.87E-07	4.18E-04
cg13436155	13	100644217		0.33	7.87E-07	4.18E-04
cg00241002	10	26931912	LOC731789	-0.27	7.87E-07	4.18E-04
cg13696609	9	100617710	FOXE1	0.28	7.93E-07	4.20E-04
cg02534850	12	66228466	HMGA2	-0.28	7.94E-07	4.20E-04
cg21211480	17	41363774	TMEM106A	0.32	7.98E-07	4.21E-04
cg27111463	2	88751405	FOXI3	0.30	8.07E-07	4.24E-04
cg15545772	1	179561111	TDRD5	0.35	8.09E-07	4.25E-04
cg00964997	4	13531195		0.30	8.14E-07	4.26E-04
cg22802994	19	7680089	KIAA1543	-0.25	8.18E-07	4.27E-04
cg17972352	10	45939668	ALOX5	-0.20	8.29E-07	4.32E-04
cg24944820	11	61723377	BEST1	0.24	8.44E-07	4.35E-04
cg16817826	3	62356323	FEZF2	0.33	8.44E-07	4.35E-04
cg21041775	12	54425418	HOXC4	0.30	8.45E-07	4.35E-04
cg01175020	10	102896475	TLX1	0.31	8.57E-07	4.39E-04
cg12979234	7	44083518	DBNL	-0.29	8.58E-07	4.39E-04
cg18759693	22	27068748	MIAT	0.30	8.65E-07	4.39E-04
cg05826677	7	48031468	SUNC1	0.25	8.65E-07	4.39E-04
cg06942701	2	162280009	TBR1	0.29	8.66E-07	4.39E-04
cg15633390	4	99851211	EIF4E	-0.26	8.67E-07	4.39E-04
cg20572816	10	102896102	TLX1	0.27	8.69E-07	4.39E-04

cg00447052	8	145556707	SCRT1	0.32	8.70E-07	4.39E-04
cg11889730	10	37967356		0.27	8.72E-07	4.39E-04
cg23778943	6	30421189		0.24	8.72E-07	4.39E-04
cg06625816	15	48484302	CTXN2	0.24	8.73E-07	4.39E-04
cg06657243	22	27068716	MIAT	0.24	8.75E-07	4.40E-04
cg10678266	7	24614348	MPP6	0.25	8.76E-07	4.40E-04
cg23856293	20	62795341	MYT1	-0.25	8.77E-07	4.40E-04
cg16024836	17	58216988		0.31	9.02E-07	4.50E-04
cg01027532	7	27155974	HOXA3	0.22	9.05E-07	4.51E-04
cg18177414	7	149389929		0.32	9.06E-07	4.51E-04
cg21977377	20	21492823	NKX2-2	0.31	9.09E-07	4.52E-04
cg09877947	5	131593287	PDLIM4	0.34	9.13E-07	4.53E-04
cg05260466	3	27756661		0.34	9.18E-07	4.54E-04
cg05809668	7	35301188		0.37	9.19E-07	4.54E-04
cg23217386	11	5653254	TRIM34	-0.33	9.20E-07	4.54E-04
cg23879118	11	67414373	ACY3	-0.23	9.30E-07	4.58E-04
cg15556886	4	81123306	PRDM8	0.31	9.31E-07	4.58E-04
cg21064916	11	44340839		0.28	9.34E-07	4.59E-04
cg14091896	9	137667343	COL5A1	-0.25	9.37E-07	4.59E-04
cg10837843	5	172199244	DUSP1	0.27	9.39E-07	4.60E-04
cg07018580	11	59480501	OR10V1	-0.27	9.43E-07	4.61E-04
cg16467262	5	140705984		0.27	9.51E-07	4.63E-04
cg15982700	7	27154911	HOXA3	0.27	9.52E-07	4.63E-04
cg27282530	3	125898680	ALDH1L1	0.27	9.58E-07	4.65E-04
cg17861161	1	3184434	PRDM16	0.20	9.62E-07	4.66E-04
cg22489321	13	100643388		0.34	9.68E-07	4.68E-04
cg25940392	21	38064699		0.32	9.72E-07	4.70E-04
cg13954667	10	88295472		0.23	9.77E-07	4.71E-04
cg24182470	12	39299844	CPNE8	0.36	9.77E-07	4.71E-04
cg22694818	1	75595970	LHX8	0.32	9.81E-07	4.72E-04
cg03966514	13	36919738	SPG20	0.31	9.85E-07	4.73E-04
cg24361385	7	27164709	HOXA3	0.26	9.90E-07	4.73E-04
cg27307257	10	102978358		0.25	9.91E-07	4.73E-04
cg22995692	5	141263164		0.35	9.91E-07	4.73E-04
cg24640735	12	10065986	CLEC2A	-0.28	9.97E-07	4.75E-04
cg26680132	6	50685247	TFAP2D	0.30	1.00E-06	4.76E-04
cg12824139	13	99405265	SLC15A1	-0.21	1.00E-06	4.76E-04
cg26811372	5	140772182	PCDHGA4	0.23	1.01E-06	4.77E-04
cg06667761	4	134073627	PCDH10	0.31	1.01E-06	4.77E-04
cg23646360	6	10886130	SYCP2L	0.20	1.01E-06	4.77E-04
cg16700000	3	27756844		0.35	1.01E-06	4.77E-04
cg23279355	5	78985592	CMYA5	0.34	1.01E-06	4.77E-04
cg19526568	13	100643087		0.28	1.01E-06	4.77E-04
cg09415272	2	88751158	FOXI3	0.23	1.02E-06	4.77E-04
cg21784036	12	39299726	CPNE8	0.35	1.02E-06	4.77E-04
cg20072442	2	80530255	CTNNA2	0.36	1.02E-06	4.77E-04
cg22845159	4	26858873		0.22	1.02E-06	4.77E-04
cg11729679	2	111877065	BCL2L11	0.30	1.03E-06	4.77E-04
cg01454215	2	27530363	UCN	0.33	1.03E-06	4.77E-04
cg07537370	2	27531124	UCN	0.23	1.03E-06	4.77E-04
cg09643136	2	223176683		0.29	1.03E-06	4.77E-04
cg26479533	7	75864324	SRRM3	0.22	1.03E-06	4.78E-04
cg12277666	1	179560870	TDRD5	0.34	1.04E-06	4.78E-04
cg23663774	4	111555440	PITX2	0.36	1.05E-06	4.79E-04
cg22471075	22	49765229		-0.32	1.05E-06	4.79E-04
cg08671647	4	30724564	PCDH7	0.28	1.05E-06	4.79E-04
cg01963134	12	39299364	CPNE8	0.37	1.05E-06	4.79E-04
cg08461339	2	241459227	ANKMY1	0.23	1.05E-06	4.79E-04
cg05431842	4	30724915	PCDH7	0.27	1.05E-06	4.79E-04
cg02426178	19	10747142	SLC44A2	-0.22	1.06E-06	4.79E-04
cg19887502	2	219761853		0.25	1.06E-06	4.80E-04
cg01959730	1	119548825		0.33	1.06E-06	4.80E-04
cg08832603	7	96652481	DLX5	0.29	1.06E-06	4.81E-04
cg12438547	13	100642971		0.30	1.06E-06	4.81E-04
cg10233654	12	89744621	DUSP6	0.28	1.07E-06	4.81E-04
cg04568492	1	229568252	ACTA1	0.36	1.08E-06	4.82E-04

cg18888655	19	17403176	ABHD8	0.31	1.08E-06	4.82E-04
cg05609536	19	41019285	SPTBN4	0.20	1.08E-06	4.82E-04
cg15575671	19	15236792	ILVBL	0.29	1.08E-06	4.82E-04
cg09484032	12	54329854		0.25	1.09E-06	4.84E-04
cg05409218	3	27756851		0.35	1.09E-06	4.84E-04
cg05127217	11	113185802	TTC12	0.28	1.09E-06	4.86E-04
cg18649133	5	93410528	FAM172A	-0.26	1.09E-06	4.86E-04
cg26398921	1	221065480		0.29	1.09E-06	4.86E-04
cg27068490	11	113185166	TTC12	0.32	1.09E-06	4.86E-04
cg22881652	4	13532951		0.29	1.10E-06	4.88E-04
cg21230493	8	26722496	ADRA1A	0.29	1.10E-06	4.89E-04
cg15054725	2	223176606		0.31	1.11E-06	4.90E-04
cg02721902	17	46825371		0.25	1.11E-06	4.90E-04
cg19547319	10	102987993	LBX1	0.26	1.12E-06	4.92E-04
cg02170386	14	70316972		0.26	1.12E-06	4.92E-04
cg12287976	11	5728046	TRIM22	-0.25	1.14E-06	4.98E-04
cg02576468	19	41074006	SPTBN4	0.34	1.15E-06	4.98E-04
cg10353539	11	113185349	TTC12	0.29	1.15E-06	4.98E-04
cg16686158	22	30476098	HORMAD2	0.31	1.15E-06	4.98E-04
cg19385331	13	100641867		0.34	1.15E-06	4.98E-04
cg11438310	5	78985434	CMYA5	0.28	1.15E-06	4.98E-04
cg19904425	14	94984530	SERPINA12	-0.28	1.16E-06	4.98E-04
cg19640526	15	70233758		0.23	1.16E-06	4.98E-04
cg11279444	1	53099053	FAM159A	0.30	1.16E-06	4.98E-04
cg00772407	12	89744471	DUSP6	0.29	1.16E-06	4.98E-04
cg17786697	5	122430509	PRDM6	0.24	1.16E-06	4.98E-04
cg11395799	1	115213248	DENND2C	0.26	1.16E-06	4.98E-04
cg21121336	12	12849068	GPR19	0.28	1.17E-06	4.99E-04
cg19605773	16	1435582		-0.25	1.17E-06	5.00E-04
cg17508941	7	19183280		0.30	1.18E-06	5.01E-04
cg17189639	17	58216551		0.28	1.18E-06	5.01E-04
cg07846297	4	174439990		0.26	1.18E-06	5.01E-04
cg17437939	7	97361252	TAC1	0.37	1.18E-06	5.01E-04
cg09121339	2	7571733		0.23	1.18E-06	5.01E-04
cg05987823	12	54428592	HOXC4	0.31	1.19E-06	5.04E-04
cg08823027	21	35831958	KCNE1	0.31	1.19E-06	5.04E-04
cg24596898	1	41849189		-0.21	1.19E-06	5.04E-04
cg03740167	10	23463746		0.29	1.20E-06	5.04E-04
cg01710897	15	91497902	RCCD1	0.28	1.20E-06	5.05E-04
cg26682904	11	101787410	ANGPTL5	-0.20	1.20E-06	5.05E-04
cg22717014	4	111542473	PITX2	0.21	1.20E-06	5.05E-04
cg24642523	14	65009265	HSPA2	0.27	1.21E-06	5.06E-04
cg16711011	2	568198		-0.26	1.22E-06	5.09E-04
cg19777067	4	174440103		0.31	1.22E-06	5.09E-04
cg27268556	1	119542629		0.28	1.23E-06	5.13E-04
cg09236819	11	64876055	C11orf2	-0.22	1.24E-06	5.15E-04
cg04587910	2	220025744	NHEJ1	0.24	1.25E-06	5.17E-04
cg09655403	5	78985495	CMYA5	0.33	1.25E-06	5.17E-04
cg18928153	9	37030203	PAX5	0.29	1.25E-06	5.17E-04
cg26856924	1	119542573		0.27	1.25E-06	5.17E-04
cg10993442	1	25257587	RUNX3	0.22	1.26E-06	5.17E-04
cg12556555	15	48484060	CTXN2	0.25	1.26E-06	5.17E-04
cg17491947	4	46994956	GABRA4	0.21	1.26E-06	5.17E-04
cg01993208	9	97808906	C9orf3	-0.22	1.27E-06	5.20E-04
cg18758796	5	131593413	PDLIM4	0.32	1.27E-06	5.20E-04
cg05317714	8	145698116	KIFC2	0.30	1.27E-06	5.20E-04
cg25280938	7	6543803	GRID2IP	0.23	1.27E-06	5.20E-04
cg14712531	6	108485043		0.34	1.28E-06	5.20E-04
cg06444282	1	20669975	VWA5B1	0.31	1.28E-06	5.20E-04
cg13628577	5	122431121	PRDM6	0.29	1.28E-06	5.20E-04
cg23967544	5	172672684		0.33	1.29E-06	5.21E-04
cg04552206	10	102896546	TLX1	0.25	1.29E-06	5.22E-04
cg05124129	12	39299501	CPNE8	0.29	1.30E-06	5.24E-04
cg02788731	1	247751498	OR2G2	-0.26	1.30E-06	5.26E-04
cg02829688	1	119527008	TBX15	0.29	1.31E-06	5.26E-04
cg01047586	2	176933407		0.27	1.31E-06	5.26E-04

cg18944010	1	119522855	TBX15	0.31	1.31E-06	5.26E-04
cg10257870	5	78985484	CMYA5	0.30	1.33E-06	5.33E-04
cg23345004	18	44526430	KATNAL2	0.28	1.34E-06	5.36E-04
cg06775930	6	126069089		0.32	1.34E-06	5.37E-04
cg07563793	1	75596336	LHX8	0.32	1.35E-06	5.39E-04
cg07162097	8	82634090	ZFAND1	-0.29	1.35E-06	5.39E-04
cg18301423	5	131593218	PDLIM4	0.34	1.35E-06	5.40E-04
cg22851295	6	10881949	GCM2	0.27	1.35E-06	5.40E-04
cg02269388	12	49658421	TUBA1C	0.31	1.36E-06	5.40E-04
cg04013595	6	30484429		0.27	1.37E-06	5.45E-04
cg06795233	2	219737392	WNT6	0.23	1.37E-06	5.45E-04
cg06718723	19	15236813	ILVBL	0.30	1.38E-06	5.47E-04
cg17842959	5	172664285		0.29	1.40E-06	5.51E-04
cg26582457	11	5067183	OR52J3	-0.23	1.40E-06	5.52E-04
cg20288916	11	56545039		-0.23	1.40E-06	5.52E-04
cg21890667	22	30476089	HORMAD2	0.31	1.41E-06	5.53E-04
cg05105138	11	5153834	OR52A5	-0.28	1.41E-06	5.54E-04
cg14845909	5	851183	ZDHHC11	-0.21	1.41E-06	5.54E-04
cg03051384	15	65197883		0.29	1.42E-06	5.55E-04
cg20898829	20	3229660		0.29	1.44E-06	5.63E-04
cg06109894	2	131185499		0.31	1.45E-06	5.65E-04
cg03564727	19	49577206	KCNA7	-0.26	1.45E-06	5.65E-04
cg11637712	17	36665844	ARHGAP23	0.22	1.46E-06	5.65E-04
cg23504411	6	27859160	HIST1H3J	0.34	1.46E-06	5.65E-04
cg08663152	19	48244519	EHD2	-0.25	1.47E-06	5.69E-04
cg12222588	18	34823808	BRUNOL4	0.30	1.48E-06	5.71E-04
cg03541338	10	121301343	RGS10	0.31	1.48E-06	5.71E-04
cg03650946	7	27154562	HOXA3	0.29	1.48E-06	5.73E-04
cg01322405	21	40757317	WRB	-0.24	1.50E-06	5.77E-04
cg22045225	12	114841792	TBX5	0.25	1.51E-06	5.79E-04
cg04873098	1	3165191	PRDM16	0.24	1.52E-06	5.79E-04
cg06235653	16	2287161	DNASE1L2	0.29	1.52E-06	5.79E-04
cg04528771	17	27893087	ABHD15	0.24	1.52E-06	5.79E-04
cg02161900	10	28035631	MKX	0.30	1.52E-06	5.79E-04
cg23639368	6	108485182		0.31	1.52E-06	5.79E-04
cg11843238	5	131593191	PDLIM4	0.37	1.52E-06	5.79E-04
cg17222164	17	43339476	LOC100133991	0.28	1.53E-06	5.79E-04
cg18146843	1	53099248	FAM159A	0.28	1.53E-06	5.79E-04
cg27093637	11	5410452	OR51M1	-0.29	1.53E-06	5.79E-04
cg12760869	12	49727110	C1QL4	0.24	1.53E-06	5.79E-04
cg04715503	16	3062795	CLDN9	0.24	1.53E-06	5.79E-04
cg14657532	2	219762543		0.23	1.53E-06	5.79E-04
cg12674357	6	106957945		0.28	1.55E-06	5.84E-04
cg05100070	6	100896760	SIM1	0.24	1.55E-06	5.85E-04
cg02352370	6	32054845	TNXB	0.23	1.56E-06	5.85E-04
cg14000361	6	10882336	GCM2	0.31	1.56E-06	5.85E-04
cg00297721	20	55200767		0.26	1.56E-06	5.85E-04
cg24064266	13	100642451		0.33	1.56E-06	5.85E-04
cg17390301	11	111250093	POU2AF1	-0.23	1.58E-06	5.88E-04
cg26981309	6	100442002	MCHR2	0.26	1.58E-06	5.89E-04
cg01189783	6	100441581	MCHR2	0.30	1.59E-06	5.91E-04
cg13289364	11	48510647	OR4A47	-0.22	1.59E-06	5.91E-04
cg01287088	5	176827392	PFN3	0.31	1.60E-06	5.91E-04
cg24690231	1	18437431	IGSF21	0.27	1.60E-06	5.91E-04
cg16799111	8	10589359	SOX7	0.28	1.60E-06	5.91E-04
cg21581821	1	190444666	FAM5C	0.28	1.60E-06	5.91E-04
cg14639847	12	54389982		0.32	1.62E-06	5.98E-04
cg00625776	12	53297384	KRT8	0.24	1.63E-06	5.99E-04
cg16688434	13	110768551		0.27	1.63E-06	5.99E-04
cg13356117	11	113185561	TTC12	0.29	1.64E-06	6.01E-04
cg18430152	7	27162401	HOXA3	0.26	1.64E-06	6.01E-04
cg15611336	15	75248496	RPP25	0.31	1.65E-06	6.02E-04
cg11990630	13	100641978		0.33	1.65E-06	6.02E-04
cg01550055	1	119543216		0.29	1.66E-06	6.03E-04
cg21581745	4	13538908		0.22	1.66E-06	6.03E-04
cg11815682	21	44486381	CBS	-0.30	1.67E-06	6.04E-04

cg02596153	6	108497141	NR2E1	0.30	1.67E-06	6.04E-04
cg26364809	7	27145159		0.28	1.68E-06	6.08E-04
cg26777836	3	26665815	LRRC3B	0.29	1.68E-06	6.08E-04
cg18866599	2	208635723		0.26	1.69E-06	6.08E-04
cg13509195	12	39299716	CPNE8	0.34	1.69E-06	6.08E-04
cg14409414	17	77777649		0.23	1.69E-06	6.08E-04
cg26206948	5	172663731	NKX2-5	0.25	1.70E-06	6.09E-04
cg26338453	15	82340023		0.21	1.70E-06	6.09E-04
cg06118609	11	7982706	NLRP10	-0.21	1.70E-06	6.09E-04
cg19068510	13	100642106		0.31	1.70E-06	6.10E-04
cg04007303	9	100618041	FOXE1	0.25	1.71E-06	6.10E-04
cg07891179	7	29923883	WIPF3	0.26	1.71E-06	6.10E-04
cg20598190	2	240866924		-0.24	1.71E-06	6.10E-04
cg04105342	4	30724841	PCDH7	0.26	1.71E-06	6.10E-04
cg26561681	1	21376633	EIF4G3	-0.21	1.73E-06	6.13E-04
cg06116636	1	101775294		0.31	1.73E-06	6.13E-04
cg15864691	7	27217606	HOXA10	-0.26	1.73E-06	6.14E-04
cg01166759	17	21934487		0.21	1.76E-06	6.20E-04
cg12139725	1	38219781	EPHA10	0.31	1.76E-06	6.22E-04
cg19628469	19	856107	ELANE	-0.20	1.77E-06	6.22E-04
cg11528176	7	42928293		0.28	1.77E-06	6.22E-04
cg10788217	11	113185148	TTC12	0.30	1.78E-06	6.22E-04
cg01790002	7	27283545	EVX1	0.32	1.78E-06	6.22E-04
cg26848718	11	32454975	WT1	0.30	1.78E-06	6.22E-04
cg22796922	7	48031800	SUNC1	0.26	1.78E-06	6.22E-04
cg07268332	11	10476494	AMPD3	0.24	1.78E-06	6.22E-04
cg13421062	7	35301069		0.29	1.79E-06	6.23E-04
cg02081006	5	122430434	PRDM6	0.30	1.80E-06	6.25E-04
cg02063759	1	119543295		0.33	1.80E-06	6.26E-04
cg04149978	1	10764896	CASZ1	-0.21	1.81E-06	6.27E-04
cg21729992	20	55200370		0.31	1.81E-06	6.27E-04
cg11916478	12	213776	IQSEC3	-0.25	1.81E-06	6.27E-04
cg25533993	19	50550284	ZNF473	0.27	1.82E-06	6.29E-04
cg21871952	1	111814146		0.22	1.83E-06	6.29E-04
cg19848940	12	112250795		-0.27	1.83E-06	6.29E-04
cg14892281	11	113185247	TTC12	0.20	1.83E-06	6.29E-04
cg16565394	6	100896674	SIM1	0.25	1.83E-06	6.29E-04
cg01310750	19	52996548	ZNF578	0.20	1.84E-06	6.29E-04
cg21134232	7	27155548	HOXA3	0.28	1.84E-06	6.29E-04
cg25775494	6	108490895	NR2E1	0.31	1.84E-06	6.29E-04
cg13702536	12	123215553	GPR81	-0.29	1.85E-06	6.29E-04
cg08832842	21	15197291		0.21	1.85E-06	6.29E-04
cg03148184	4	111562513		0.28	1.85E-06	6.29E-04
cg00395210	12	48592465		0.28	1.85E-06	6.29E-04
cg25334575	13	100641744		0.32	1.85E-06	6.30E-04
cg15134714	8	37558448		0.21	1.86E-06	6.30E-04
cg00318947	7	27154914	HOXA3	0.26	1.86E-06	6.30E-04
cg22772747	7	27164285	HOXA3	0.28	1.86E-06	6.30E-04
cg25650956	17	10422077	MYH1	-0.22	1.87E-06	6.31E-04
cg00611789	5	78985432	CMYA5	0.33	1.88E-06	6.34E-04
cg16047053	11	57972613	OR1S2	-0.22	1.88E-06	6.34E-04
cg04624601	12	113910385	LHX5	0.23	1.89E-06	6.35E-04
cg11602901	3	62354878		0.25	1.89E-06	6.35E-04
cg19797536	7	91909243	ANKIB1	-0.24	1.89E-06	6.35E-04
cg19186614	12	214194	IQSEC3	-0.22	1.89E-06	6.35E-04
cg22840780	13	58207650	PCDH17	0.31	1.89E-06	6.35E-04
cg08553601	4	53729461	RASL11B	0.24	1.90E-06	6.35E-04
cg24546155	18	44786426		0.28	1.90E-06	6.37E-04
cg08425070	7	75864319	SRRM3	0.24	1.91E-06	6.39E-04
cg18244817	5	42949610		0.30	1.91E-06	6.39E-04
cg22871653	1	119535817		0.31	1.93E-06	6.43E-04
cg18829827	5	122422500		0.31	1.93E-06	6.43E-04
cg02343962	15	70233653		0.22	1.93E-06	6.43E-04
cg14620572	9	127215669	GPR144	0.26	1.94E-06	6.45E-04
cg22664614	7	129991187	CPA5	-0.25	1.95E-06	6.46E-04
cg19258034	19	41055125	SPTBN4	0.30	1.95E-06	6.47E-04

cg22864077	2	131185333		0.23	1.97E-06	6.51E-04
cg10959198	15	93632896	RGMA	0.35	1.97E-06	6.51E-04
cg00295794	13	100641409		0.36	1.98E-06	6.55E-04
cg02235529	11	56311179	OR8U8	-0.29	1.99E-06	6.56E-04
cg22685215	1	178232856	RASAL2	-0.24	1.99E-06	6.56E-04
cg11337945	5	2755158	C5orf38	0.32	2.00E-06	6.57E-04
cg08461949	19	52104632		0.30	2.00E-06	6.57E-04
cg23245905	10	99735133	CRTAC1	0.26	2.01E-06	6.59E-04
cg06119874	1	207225424	PFKFB2	-0.25	2.05E-06	6.70E-04
cg02504902	8	53326922		-0.27	2.06E-06	6.70E-04
cg14326413	4	81123380	PRDM8	0.34	2.06E-06	6.70E-04
cg01978237	6	100896803	SIM1	0.28	2.06E-06	6.70E-04
cg02699218	5	132150128	ANKRD43	0.29	2.06E-06	6.70E-04
cg25579180	7	71098623	WBSCR17	0.22	2.07E-06	6.70E-04
cg03719428	12	54376094		0.23	2.07E-06	6.70E-04
cg23680922	19	52104997		0.21	2.07E-06	6.70E-04
cg12520549	2	162280741	TBR1	0.26	2.08E-06	6.72E-04
cg07465387	14	38058263		0.28	2.08E-06	6.72E-04
cg06654537	13	100641317		0.30	2.08E-06	6.72E-04
cg17740822	12	89744609	DUSP6	0.29	2.09E-06	6.73E-04
cg16890796	19	49937087	SLC17A7	0.23	2.09E-06	6.73E-04
cg24565742	13	21650374		0.23	2.09E-06	6.73E-04
cg02026204	22	30663007	OSM	-0.20	2.10E-06	6.74E-04
cg08579858	22	29709964	RASL10A	0.26	2.11E-06	6.77E-04
cg02750262	18	72916776	ZADH2	0.28	2.12E-06	6.77E-04
cg21849844	13	100611865		0.32	2.12E-06	6.77E-04
cg20729747	12	14928083	H2AFJ	0.30	2.13E-06	6.79E-04
cg01070065	1	32714023	FAM167B	0.21	2.13E-06	6.80E-04
cg22154659	7	27134369	HOXA1	0.28	2.14E-06	6.82E-04
cg12061531	17	21729912		0.22	2.15E-06	6.84E-04
cg11273947	15	68120757	LBXCOR1	0.30	2.15E-06	6.84E-04
cg27564792	3	62360162	FEZF2	0.27	2.15E-06	6.84E-04
cg14450231	5	172199425	DUSP1	0.24	2.17E-06	6.88E-04
cg05656688	1	25254088	RUNX3	-0.21	2.17E-06	6.89E-04
cg04352304	10	102475896		0.26	2.17E-06	6.89E-04
cg02719608	17	77776428		0.26	2.19E-06	6.92E-04
cg19519964	2	223290012	SGPP2	0.28	2.20E-06	6.94E-04
cg12177743	11	113185079	TTC12	0.32	2.20E-06	6.95E-04
cg13425960	19	50550359	ZNF473	0.22	2.21E-06	6.96E-04
cg21158087	17	58216415		0.31	2.21E-06	6.96E-04
cg08906307	1	221065434		0.23	2.22E-06	6.98E-04
cg22498143	19	40993991	SPTBN4	0.28	2.23E-06	7.02E-04
cg23842796	1	156358404		0.26	2.24E-06	7.05E-04
cg06530563	12	115132889		0.21	2.25E-06	7.05E-04
cg10977643	14	97082103		-0.26	2.26E-06	7.05E-04
cg00402417	13	100649423		0.30	2.26E-06	7.05E-04
cg26123256	2	27530670	UCN	0.23	2.26E-06	7.05E-04
cg06008480	11	113185089	TTC12	0.33	2.26E-06	7.05E-04
cg14621323	5	176827161	PFN3	0.26	2.27E-06	7.06E-04
cg21516287	7	150020855	ACTR3C	0.24	2.27E-06	7.06E-04
cg13537061	7	35298720		0.22	2.27E-06	7.06E-04
cg13888838	2	220025712	NHEJ1	0.25	2.28E-06	7.07E-04
cg27478313	6	1624720	GMDS	0.27	2.29E-06	7.08E-04
cg08929002	6	108496991	NR2E1	0.25	2.30E-06	7.08E-04
cg10050816	16	86549237		0.26	2.33E-06	7.15E-04
cg17651959	20	21498100		0.30	2.33E-06	7.16E-04
cg02454982	1	3689201	LOC388588	0.28	2.34E-06	7.16E-04
cg18827332	12	103344506		0.22	2.35E-06	7.19E-04
cg08458733	8	37555577	ZNF703	0.23	2.35E-06	7.19E-04
cg02390319	1	3310268	PRDM16	0.30	2.36E-06	7.20E-04
cg03592166	11	70668691	SHANK2	-0.24	2.37E-06	7.21E-04
cg11772171	11	2292544	ASCL2	0.29	2.37E-06	7.21E-04
cg11873482	7	97361244	TAC1	0.33	2.37E-06	7.21E-04
cg23684204	15	91497937	RCCD1	0.28	2.39E-06	7.25E-04
cg01277155	7	103086237	SLC26A5	0.26	2.39E-06	7.25E-04
cg15579587	2	238600061	LRRFIP1	0.26	2.41E-06	7.31E-04

cg10811961	12	54090346		0.26	2.45E-06	7.41E-04
cg17893742	15	99967055		-0.21	2.45E-06	7.41E-04
cg06000330	7	19183231		0.27	2.46E-06	7.42E-04
cg17359076	12	49658802	TUBA1C	0.30	2.46E-06	7.42E-04
cg02995271	8	144816322	FAM83H	0.28	2.47E-06	7.43E-04
cg04193160	1	228400419	OBSCN	0.25	2.47E-06	7.43E-04
cg06820006	12	131488926	GPR133	-0.22	2.48E-06	7.45E-04
cg26952803	10	63926610		-0.26	2.48E-06	7.45E-04
cg01814191	12	89744524	DUSP6	0.29	2.48E-06	7.45E-04
cg21837192	12	54398765		0.26	2.48E-06	7.45E-04
cg15383322	7	98100419		0.24	2.49E-06	7.45E-04
cg17210929	1	17034180	ESPNP	0.24	2.49E-06	7.46E-04
cg16762841	13	100646453		0.33	2.50E-06	7.47E-04
cg05546863	16	66613220	CMTM2	0.24	2.52E-06	7.51E-04
cg15925940	14	105330630	KIAA0284	0.24	2.52E-06	7.51E-04
cg01387972	19	15550968	WIZ	-0.21	2.52E-06	7.51E-04
cg17918002	10	28035719	MKX	0.29	2.53E-06	7.52E-04
cg24235037	2	160919532	PLA2R1	0.23	2.55E-06	7.54E-04
cg04744409	6	105388191		0.26	2.55E-06	7.54E-04
cg22778421	12	12849229	GPR19	0.33	2.55E-06	7.54E-04
cg23130075	11	67418365	ACY3	-0.21	2.56E-06	7.56E-04
cg01323148	2	206551212	NRP2	0.31	2.56E-06	7.56E-04
cg27555365	17	7338618	TMEM102	0.29	2.56E-06	7.56E-04
cg25814096	17	73782472	UNK	0.21	2.58E-06	7.60E-04
cg00860808	2	171670500		0.28	2.61E-06	7.67E-04
cg09236658	6	108485152		0.30	2.61E-06	7.67E-04
cg20180370	6	50691003	TFAP2D	0.28	2.62E-06	7.69E-04
cg04639421	6	32411897	HLA-DRA	-0.27	2.63E-06	7.69E-04
cg14896559	19	3481957	C19orf77	0.20	2.63E-06	7.69E-04
cg01832036	17	48050274	DLX4	0.31	2.63E-06	7.69E-04
cg10170049	15	94774288		0.30	2.63E-06	7.69E-04
cg02119840	7	150020900	ACTR3C	0.20	2.64E-06	7.69E-04
cg17980283	2	45156642		0.22	2.64E-06	7.70E-04
cg07145683	12	53297443	KRT8	0.28	2.65E-06	7.71E-04
cg16105676	6	108497424	NR2E1	0.29	2.65E-06	7.72E-04
cg15006175	8	145698660	KIFC2	0.35	2.66E-06	7.72E-04
cg13985518	7	27143788	HOXA2	0.25	2.66E-06	7.72E-04
cg07239716	12	89744488	DUSP6	0.30	2.66E-06	7.72E-04
cg12386646	15	41787261	ITPKA	0.28	2.67E-06	7.73E-04
cg22560214	4	13531213		0.28	2.67E-06	7.74E-04
cg21493516	12	54446033	HOXC4	0.30	2.68E-06	7.77E-04
cg08668790	19	58220662	ZNF154	0.31	2.69E-06	7.77E-04
cg20025656	1	229570230	ACTA1	0.23	2.70E-06	7.78E-04
cg00157796	6	159590231	FNDC1	0.27	2.72E-06	7.85E-04
cg02091103	9	79637730		0.24	2.73E-06	7.85E-04
cg09975995	4	175899203	ADAM29	-0.27	2.73E-06	7.86E-04
cg07270153	7	19185260	FERD3L	0.27	2.74E-06	7.87E-04
cg04154027	5	78985588	CMYA5	0.32	2.74E-06	7.87E-04
cg13980454	14	36985905	NKX2-1	0.21	2.74E-06	7.87E-04
cg14266527	4	151501298	LRBA	0.27	2.75E-06	7.87E-04
cg22410026	6	30483923		0.26	2.75E-06	7.87E-04
cg10542584	3	62356223	FEZF2	0.25	2.75E-06	7.87E-04
cg03255379	2	85531470	TCF7L1	-0.22	2.75E-06	7.87E-04
cg14832317	4	13526597		0.21	2.76E-06	7.87E-04
cg11644586	11	66360200	CCDC87	0.23	2.76E-06	7.88E-04
cg03913859	2	177399921		-0.30	2.77E-06	7.88E-04
cg09516476	6	10881925	GCM2	0.28	2.77E-06	7.89E-04
cg15042866	3	27755970		0.31	2.77E-06	7.89E-04
cg09936190	10	43892956	HNRNPF	0.26	2.78E-06	7.91E-04
cg13193239	4	111539479	PITX2	0.24	2.79E-06	7.91E-04
cg26801014	11	88241769	GRM5	0.23	2.79E-06	7.91E-04
cg00158122	10	101290029		0.23	2.79E-06	7.91E-04
cg26195829	14	61122155		0.24	2.80E-06	7.92E-04
cg06776146	13	100649131		0.31	2.80E-06	7.92E-04
cg09513416	6	71319075		-0.22	2.80E-06	7.92E-04
cg24157892	17	59473707		0.33	2.81E-06	7.93E-04

cg20433275	1	156358408		0.29	2.81E-06	7.93E-04
cg01218896	15	96911988		0.21	2.81E-06	7.93E-04
cg04387835	17	4649076	ZMYND15	0.28	2.81E-06	7.93E-04
cg06759189	17	77777947		0.23	2.83E-06	7.93E-04
cg17180237	1	119545275		0.24	2.83E-06	7.93E-04
cg01950845	15	93632730	RGMA	0.33	2.83E-06	7.93E-04
cg09068158	1	53098641	FAM159A	0.25	2.84E-06	7.93E-04
cg13417420	12	12849159	GPR19	0.32	2.84E-06	7.93E-04
cg22776912	15	81630789	TMC3	-0.26	2.86E-06	7.95E-04
cg06928993	19	49313421	BCAT2	-0.21	2.86E-06	7.95E-04
cg09825979	7	27179432		0.26	2.87E-06	7.95E-04
cg07751277	13	100642487		0.34	2.87E-06	7.95E-04
cg23724858	6	32055711	TNXB	0.26	2.87E-06	7.95E-04
cg09854515	4	111554385	PITX2	0.22	2.88E-06	7.98E-04
cg21858255	12	104609609	TXNRD1	0.31	2.89E-06	7.98E-04
cg01456368	3	52351859	DNAH1	0.27	2.89E-06	7.99E-04
cg04285666	5	122422863		0.28	2.89E-06	7.99E-04
cg00116092	1	217313044		0.30	2.90E-06	7.99E-04
cg20695936	1	25257624	RUNX3	0.24	2.91E-06	7.99E-04
cg06928346	12	12849096	GPR19	0.33	2.91E-06	7.99E-04
cg01792403	13	100626995		0.30	2.92E-06	8.03E-04
cg15957748	4	146267426		-0.28	2.95E-06	8.10E-04
cg18573327	12	115123875		0.24	2.96E-06	8.11E-04
cg07501233	13	100641900		0.33	2.97E-06	8.14E-04
cg09907170	2	111877177	BCL2L11	0.26	2.99E-06	8.17E-04
cg27624162	2	111876008		0.25	3.02E-06	8.22E-04
cg22747650	7	150020218	LRRC61	0.21	3.02E-06	8.22E-04
cg06158650	1	119522637	TBX15	0.31	3.02E-06	8.23E-04
cg20249584	1	233462803	KIAA1804	0.28	3.04E-06	8.27E-04
cg21165027	5	172663784	NKX2-5	0.25	3.04E-06	8.27E-04
cg05971894	12	54399254		0.21	3.04E-06	8.27E-04
cg22765426	6	30483837		0.22	3.05E-06	8.27E-04
cg02293817	7	35808294	LOC442293	-0.24	3.06E-06	8.28E-04
cg11856078	2	157178165		0.33	3.07E-06	8.28E-04
cg15037137	7	27144302		0.25	3.07E-06	8.28E-04
cg21748207	2	26395359	FAM59B	0.24	3.07E-06	8.28E-04
cg20114732	6	166579135	T	0.30	3.07E-06	8.28E-04
cg23319696	5	2111870		-0.24	3.08E-06	8.28E-04
cg22197708	11	59855616	MS4A2	-0.26	3.10E-06	8.31E-04
cg09284655	14	70317228		0.26	3.10E-06	8.31E-04
cg26027669	1	92952467	GFI1	0.34	3.10E-06	8.31E-04
cg02019988	3	50311211	SEMA3B	0.21	3.11E-06	8.32E-04
cg07541701	12	54389934		0.26	3.13E-06	8.35E-04
cg22288577	15	91498497	RCCD1	0.29	3.14E-06	8.37E-04
cg26314330	3	27772633		0.31	3.14E-06	8.37E-04
cg05650846	16	3078142	CCDC64B	0.26	3.15E-06	8.37E-04
cg20489026	1	160053931	KCNJ9	0.28	3.15E-06	8.37E-04
cg11431402	9	126135408	CRB2	-0.23	3.15E-06	8.37E-04
cg09143673	5	172664439		0.30	3.15E-06	8.37E-04
cg14042889	7	27169208	HOXA4	0.23	3.16E-06	8.38E-04
cg12062819	1	156358231		0.34	3.16E-06	8.38E-04
cg11689813	1	229570052	ACTA1	0.30	3.17E-06	8.40E-04
cg09662411	1	92946132	GFI1	0.21	3.18E-06	8.40E-04
cg04250451	1	25257784	RUNX3	0.26	3.18E-06	8.40E-04
cg03729251	4	151501035	LRBA	0.31	3.18E-06	8.40E-04
cg22322465	4	174459201	NBLA00301	0.27	3.19E-06	8.40E-04
cg14308479	11	2292677	ASCL2	0.24	3.19E-06	8.40E-04
cg05907933	5	77268888		0.31	3.21E-06	8.45E-04
cg01268824	19	58220818	ZNF154	0.30	3.22E-06	8.46E-04
cg11092487	20	33585188	MYH7B	0.26	3.22E-06	8.46E-04
cg14435720	9	139565087	EGFL7	-0.21	3.24E-06	8.48E-04
cg26249834	14	52479816	NID2	-0.20	3.25E-06	8.50E-04
cg13987489	14	99664107	BCL11B	-0.22	3.26E-06	8.51E-04
cg13213810	22	51158720	SHANK3	0.29	3.28E-06	8.55E-04
cg17775899	7	27179426		0.21	3.30E-06	8.60E-04
cg18087266	1	25257629	RUNX3	0.21	3.31E-06	8.63E-04

cg02539083	7	97361148	TAC1	0.27	3.32E-06	8.64E-04
cg00062245	17	42462260	ITGA2B	0.23	3.33E-06	8.64E-04
cg22236681	4	146267610		-0.29	3.33E-06	8.64E-04
cg01830023	5	134374439		0.23	3.34E-06	8.66E-04
cg26489413	11	10476976	AMPD3	0.22	3.35E-06	8.68E-04
cg22886139	5	140306985	PCDHAC1	0.20	3.36E-06	8.69E-04
cg09232478	1	119548852		0.29	3.37E-06	8.70E-04
cg07060505	12	116756136		-0.23	3.39E-06	8.74E-04
cg15368905	6	78172337	HTR1B	0.32	3.39E-06	8.74E-04
cg19968840	15	45409319	DUOXA2	0.32	3.43E-06	8.80E-04
cg08640790	15	91498075	RCCD1	0.29	3.45E-06	8.85E-04
cg08287724	21	35832141	KCNE1	0.27	3.45E-06	8.85E-04
cg08302771	16	56710256	MT1IP	0.21	3.48E-06	8.90E-04
cg01287975	7	97361241	TAC1	0.34	3.48E-06	8.90E-04
cg24632241	2	80530431	CTNNA2	0.35	3.49E-06	8.93E-04
cg07645844	8	26722635	ADRA1A	0.26	3.50E-06	8.93E-04
cg27632435	12	49658821	TUBA1C	0.33	3.50E-06	8.93E-04
cg04740898	17	56402669	BZRAP1	0.20	3.51E-06	8.94E-04
cg05270922	1	101775452		0.34	3.51E-06	8.94E-04
cg11907262	19	34310911		0.24	3.53E-06	8.98E-04
cg23426238	7	103086367	SLC26A5	0.28	3.53E-06	8.98E-04
cg22129545	2	27531320	UCN	0.30	3.54E-06	8.99E-04
cg15683295	7	27245542		0.26	3.56E-06	9.04E-04
cg08048194	12	55641538	OR6C74	-0.24	3.56E-06	9.04E-04
cg16167240	11	122852284	BSX	0.25	3.57E-06	9.05E-04
cg08452061	5	172199642	DUSP1	0.25	3.58E-06	9.07E-04
cg21966075	1	156358066		0.22	3.58E-06	9.07E-04
cg23461714	11	113184990	TTC12	0.31	3.61E-06	9.12E-04
cg19289461	16	30394812	Sep-01	-0.23	3.62E-06	9.12E-04
cg18120446	4	13531076		0.29	3.63E-06	9.12E-04
cg22409854	6	88877301		0.31	3.63E-06	9.12E-04
cg09287190	13	100640914		0.33	3.63E-06	9.12E-04
cg21831481	4	41880604		0.24	3.63E-06	9.12E-04
cg23049226	15	102182534	TM2D3	-0.20	3.64E-06	9.12E-04
cg07496173	7	57472222		-0.22	3.65E-06	9.12E-04
cg08762750	15	91497995	RCCD1	0.26	3.66E-06	9.13E-04
cg27402429	3	72788294		0.28	3.66E-06	9.13E-04
cg25923207	11	62192276		0.27	3.68E-06	9.17E-04
cg08124910	17	43339515	LOC100133991	0.25	3.69E-06	9.19E-04
cg07660236	6	28367883	ZSCAN12	0.25	3.70E-06	9.20E-04
cg03701266	5	10649678	ANKRD33B	0.22	3.72E-06	9.21E-04
cg25600049	13	53419729	PCDH8	0.28	3.73E-06	9.22E-04
cg07845581	17	56326510	LPO	0.21	3.73E-06	9.22E-04
cg10094489	7	155599026	SHH	0.23	3.75E-06	9.25E-04
cg17158913	1	10764886	CASZ1	-0.24	3.76E-06	9.27E-04
cg12564285	5	131593104	PDLIM4	0.27	3.77E-06	9.27E-04
cg21752601	2	27531310	UCN	0.26	3.78E-06	9.29E-04
cg03379552	10	99734662	CRTAC1	0.25	3.78E-06	9.29E-04
cg11374425	3	62359677	FEZF2	0.33	3.80E-06	9.33E-04
cg23461615	7	103086074	SLC26A5	0.28	3.86E-06	9.43E-04
cg21963535	20	44098285	WFDC2	0.24	3.88E-06	9.46E-04
cg26885268	7	149389941		0.27	3.88E-06	9.46E-04
cg09591524	7	27150031	HOXA3	0.26	3.89E-06	9.47E-04
cg15026277	17	41363789	TMEM106A	0.27	3.89E-06	9.47E-04
cg09022993	4	52917875	SPATA18	0.30	3.92E-06	9.53E-04
cg01701415	10	74870311	NUDT13	0.31	3.93E-06	9.54E-04
cg15837109	15	32964208	SCG5	-0.24	3.95E-06	9.58E-04
cg13884376	9	130505349	SH2D3C	0.25	3.96E-06	9.59E-04
cg03536885	7	27163820	HOXA3	0.25	3.97E-06	9.61E-04
cg16027403	1	247978791	OR14A16	-0.26	4.02E-06	9.71E-04
cg18805066	7	27134259	HOXA1	0.28	4.04E-06	9.74E-04
cg04518342	5	131593106	PDLIM4	0.35	4.05E-06	9.76E-04
cg06635722	4	13532622		0.25	4.05E-06	9.76E-04
cg10534720	10	102898675		0.21	4.06E-06	9.77E-04
cg01689239	12	48592635		0.21	4.08E-06	9.80E-04
cg07741162	5	122429966	PRDM6	0.24	4.09E-06	9.80E-04

cg02083234	3	62357769	FEZF2	0.24	4.09E-06	9.80E-04
cg16701896	7	27233411		0.28	4.09E-06	9.80E-04
cg19951244	5	172672766		0.28	4.10E-06	9.81E-04
cg26402660	7	27179268		0.29	4.10E-06	9.81E-04
cg24361162	1	156358116		0.26	4.11E-06	9.82E-04
cg11241627	7	19185165	FERD3L	0.27	4.12E-06	9.83E-04
cg02621030	16	29151700		-0.24	4.12E-06	9.83E-04
cg14888916	8	99951420		0.25	4.13E-06	9.84E-04
cg06665391	20	39312290		0.30	4.13E-06	9.84E-04
cg05950877	19	11784647	ZNF833	0.28	4.14E-06	9.84E-04
cg03795847	3	44596512	ZNF167	0.23	4.14E-06	9.84E-04
cg24019564	1	25257566	RUNX3	0.25	4.14E-06	9.84E-04
cg03776662	5	122430677	PRDM6	0.24	4.17E-06	9.91E-04
cg11101117	10	102997865	FLJ41350	0.25	4.19E-06	9.95E-04
cg01557798	1	228400628	OBSCN	0.24	4.19E-06	9.95E-04
cg00507738	12	49658678	TUBA1C	0.30	4.20E-06	9.95E-04
cg11355215	20	10198044	SNAP25	0.32	4.21E-06	9.96E-04
cg25741215	2	63287082		0.32	4.21E-06	9.96E-04
cg24865270	6	10422447		0.27	4.21E-06	9.96E-04
cg22704068	1	3689178	LOC388588	0.26	4.22E-06	9.96E-04
cg02624701	19	49937176	SLC17A7	0.26	4.25E-06	1.00E-03
cg05120944	12	54090254		0.24	4.27E-06	1.00E-03
cg06834912	16	86547203	FOXF1	0.26	4.29E-06	1.01E-03
cg01393632	12	103344685		0.32	4.31E-06	1.01E-03
cg17837894	7	27531429		-0.22	4.32E-06	1.01E-03
cg24542751	17	43339589	LOC100133991	0.29	4.33E-06	1.01E-03
cg11248896	2	177003747		0.22	4.34E-06	1.01E-03
cg23248357	5	2755376	C5orf38	0.25	4.34E-06	1.01E-03
cg02776659	21	47717406	C21orf57	0.27	4.35E-06	1.01E-03
cg02356551	8	53013726		0.31	4.38E-06	1.02E-03
cg00856041	6	27798455		0.34	4.38E-06	1.02E-03
cg09469610	15	68113110		0.23	4.40E-06	1.02E-03
cg12118269	10	102893980	TLX1	0.32	4.43E-06	1.03E-03
cg07211140	11	32455025	WT1	0.30	4.45E-06	1.03E-03
cg25597138	6	28511790		0.21	4.45E-06	1.03E-03
cg27110886	10	99734912	CRTAC1	0.24	4.47E-06	1.03E-03
cg11472521	17	48050512	DLX4	0.23	4.48E-06	1.03E-03
cg27343836	16	86549678		0.23	4.51E-06	1.04E-03
cg22477343	20	39312069		0.29	4.51E-06	1.04E-03
cg13705391	6	32118441	PRRT1	0.23	4.53E-06	1.04E-03
cg04927889	3	62360464	FEZF2	0.27	4.55E-06	1.04E-03
cg19227130	3	62359392	FEZF2	0.31	4.55E-06	1.04E-03
cg00399175	2	26395448	FAM59B	0.23	4.56E-06	1.04E-03
cg07436152	6	50819236		0.22	4.57E-06	1.05E-03
cg27544000	1	17034075	ESPNP	0.23	4.58E-06	1.05E-03
cg02627455	7	27157818	HOXA3	0.26	4.61E-06	1.05E-03
cg20100910	10	102475429		0.31	4.62E-06	1.05E-03
cg19675142	1	116107015		0.26	4.64E-06	1.06E-03
cg17395064	12	104609476	TXNRD1	0.31	4.65E-06	1.06E-03
cg09978906	14	74345331	PTGR2	-0.21	4.66E-06	1.06E-03
cg12629515	6	27859811	HIST1H3J	0.27	4.66E-06	1.06E-03
cg01429039	4	52918065	SPATA18	0.29	4.68E-06	1.06E-03
cg18803655	12	12849748	GPR19	0.34	4.68E-06	1.06E-03
cg12765028	4	13526659		0.21	4.69E-06	1.06E-03
cg26168907	6	32977223	HLA-DOA	0.20	4.70E-06	1.06E-03
cg14471364	2	157177936		0.27	4.71E-06	1.06E-03
cg00682734	20	55200973		0.26	4.72E-06	1.06E-03
cg11796095	21	44486452	CBS	-0.29	4.76E-06	1.07E-03
cg16966315	7	149112318		0.28	4.76E-06	1.07E-03
cg23221090	10	64892741	NRBF2	-0.21	4.77E-06	1.07E-03
cg10128511	3	98452940	ST3GAL6	0.27	4.79E-06	1.07E-03
cg13029423	16	86549276		0.27	4.79E-06	1.07E-03
cg14483142	3	8811758	OXTR	-0.22	4.80E-06	1.07E-03
cg25386234	1	119542159		0.24	4.81E-06	1.07E-03
cg00817501	4	138440719	PCDH18	-0.23	4.81E-06	1.07E-03
cg02732252	16	3068014	CLDN6	0.27	4.82E-06	1.07E-03

cg21038785	15	53087068		0.28	4.82E-06	1.07E-03
cg08815081	10	28035731	MKX	0.28	4.84E-06	1.08E-03
cg27641141	10	43892910	HNRNPF	0.22	4.84E-06	1.08E-03
cg25236050	12	55794783	OR6C65	-0.20	4.86E-06	1.08E-03
cg24217984	15	93633278	RGMA	0.26	4.90E-06	1.09E-03
cg04365224	3	72788183		0.28	4.91E-06	1.09E-03
cg24144440	1	119526882	TBX15	0.26	4.91E-06	1.09E-03
cg06625730	20	18456304	POLR3F	-0.23	4.93E-06	1.09E-03
cg08465346	12	54441458	HOXC4	0.29	4.93E-06	1.09E-03
cg19837259	13	28527437		0.23	4.93E-06	1.09E-03
cg22474464	20	21492914	NKX2-2	0.32	4.94E-06	1.09E-03
cg19360852	12	12848977	GPR19	0.25	4.95E-06	1.09E-03
cg14979301	5	42994123		0.27	4.97E-06	1.09E-03
cg26742995	17	43339594	LOC100133991	0.26	4.98E-06	1.09E-03
cg27072012	16	3078339	CCDC64B	0.22	5.01E-06	1.10E-03
cg19132462	11	10476608	AMPD3	0.24	5.01E-06	1.10E-03
cg09680447	4	1037615		-0.22	5.02E-06	1.10E-03
cg00218620	2	219866460	MIR375	0.26	5.03E-06	1.10E-03
cg00192136	11	20178496	DBX1	0.31	5.05E-06	1.10E-03
cg10784928	19	19971578		0.23	5.06E-06	1.10E-03
cg09989644	1	156358119		0.21	5.06E-06	1.10E-03
cg16551732	4	169799087	PALLD	0.28	5.07E-06	1.11E-03
cg13001868	17	43339223	C17orf46	0.31	5.07E-06	1.11E-03
cg15263666	3	27756317		0.21	5.08E-06	1.11E-03
cg14906455	17	2628088		0.29	5.09E-06	1.11E-03
cg04865531	22	51159147	SHANK3	0.27	5.09E-06	1.11E-03
cg12506930	19	58220718	ZNF154	0.30	5.10E-06	1.11E-03
cg00073837	2	223177008		0.25	5.10E-06	1.11E-03
cg03522107	1	16553329		0.22	5.11E-06	1.11E-03
cg00007466	19	7585086	ZNF358	-0.24	5.11E-06	1.11E-03
cg08871722	17	21495791		-0.21	5.12E-06	1.11E-03
cg21355828	7	149389814		0.24	5.13E-06	1.11E-03
cg00778084	1	173681595		-0.23	5.14E-06	1.11E-03
cg14377594	9	1051505	DMRT2	0.23	5.14E-06	1.11E-03
cg23054426	7	19185267	FERD3L	0.24	5.14E-06	1.11E-03
cg20272962	17	56326765	LPO	0.24	5.15E-06	1.11E-03
cg03234186	19	58220657	ZNF154	0.31	5.16E-06	1.11E-03
cg22798849	7	27155234	HOXA3	0.21	5.21E-06	1.12E-03
cg00339682	4	13531163		0.30	5.23E-06	1.12E-03
cg06723863	16	68270129	ESRP2	0.30	5.25E-06	1.13E-03
cg07280807	14	70317239		0.24	5.27E-06	1.13E-03
cg05583831	3	27772705		0.30	5.28E-06	1.13E-03
cg19923650	5	172659730	NKX2-5	0.32	5.32E-06	1.14E-03
cg21647227	1	119527111	TBX15	0.27	5.32E-06	1.14E-03
cg07066898	19	17717094	UNC13A	0.30	5.33E-06	1.14E-03
cg11367354	7	96651983	DLX5	0.24	5.34E-06	1.14E-03
cg10397527	6	32977217	HLA-DOA	0.22	5.34E-06	1.14E-03
cg04965987	21	38378539	DSCR6	0.25	5.36E-06	1.14E-03
cg05886811	6	17280740	RBM24	0.23	5.36E-06	1.14E-03
cg06838283	5	111090142	C5orf13	0.24	5.36E-06	1.14E-03
cg11594833	12	54444985	HOXC4	0.29	5.38E-06	1.15E-03
cg07447742	13	49793170	MLNR	0.21	5.38E-06	1.15E-03
cg22893494	3	140952551	ACPL2	0.21	5.39E-06	1.15E-03
cg18468354	14	36993517		0.23	5.40E-06	1.15E-03
cg08860190	19	6719342	C3	-0.26	5.40E-06	1.15E-03
cg11706983	7	27233454		0.30	5.42E-06	1.15E-03
cg22730140	5	172660996	NKX2-5	0.26	5.44E-06	1.15E-03
cg08247376	17	43339358	C17orf46	0.26	5.44E-06	1.15E-03
cg03740978	18	24127875	KCTD1	0.29	5.44E-06	1.15E-03
cg17593584	2	220025719	NHEJ1	0.26	5.44E-06	1.15E-03
cg05160381	7	156740259		-0.22	5.45E-06	1.15E-03
cg23217622	1	156405438		0.28	5.45E-06	1.15E-03
cg15580417	1	160053947	KCNJ9	0.24	5.45E-06	1.15E-03
cg07153665	16	66613206	CMTM2	0.25	5.45E-06	1.15E-03
cg11760198	18	34854936	BRUNOL4	0.23	5.46E-06	1.15E-03
cg10624054	12	53297547	KRT8	0.31	5.46E-06	1.15E-03

cg21177821	14	61121712		0.21	5.48E-06	1.15E-03
cg01959416	7	150020818	ACTR3C	0.20	5.55E-06	1.17E-03
cg01989621	7	57472709		-0.24	5.56E-06	1.17E-03
cg00138474	6	30484334		0.27	5.56E-06	1.17E-03
cg18646521	2	111875858		0.24	5.59E-06	1.17E-03
cg21606036	5	78985900	CMYA5	0.21	5.62E-06	1.18E-03
cg04539503	19	34311327		0.30	5.63E-06	1.18E-03
cg18898125	8	24770381	NEFM	0.30	5.67E-06	1.18E-03
cg08932256	12	116008258		0.23	5.68E-06	1.18E-03
cg22173131	11	75480171	DGAT2	0.23	5.68E-06	1.18E-03
cg08589354	16	86548895		0.27	5.69E-06	1.18E-03
cg12069276	11	10476242	AMPD3	0.23	5.70E-06	1.19E-03
cg27615366	5	131592974	PDLIM4	0.20	5.72E-06	1.19E-03
cg15540602	14	61122911		0.24	5.74E-06	1.19E-03
cg08489883	20	21498294		0.23	5.78E-06	1.20E-03
cg27177554	10	16562102	C1QL3	0.31	5.79E-06	1.20E-03
cg00193963	7	29923832	WIPF3	0.25	5.83E-06	1.21E-03
cg03490331	5	172672817		0.26	5.84E-06	1.21E-03
cg05329352	10	112838983	ADRA2A	0.21	5.86E-06	1.21E-03
cg07769790	15	83317281	CPEB1	0.24	5.87E-06	1.21E-03
cg14069333	2	16443899		0.23	5.88E-06	1.21E-03
cg05000994	7	27131807		0.28	5.90E-06	1.21E-03
cg21669441	15	83349864	AP3B2	0.24	5.90E-06	1.21E-03
cg12477370	6	45391852	RUNX2	0.25	5.91E-06	1.21E-03
cg12305431	7	27157855	HOXA3	0.26	5.93E-06	1.22E-03
cg10858480	14	61122774		0.23	5.95E-06	1.22E-03
cg02931225	4	96446177	UNC5C	0.23	5.95E-06	1.22E-03
cg01627847	17	43339512	LOC100133991	0.27	5.95E-06	1.22E-03
cg16282892	3	193987757		-0.22	5.96E-06	1.22E-03
cg01480180	7	90896329	FZD1	0.28	5.97E-06	1.22E-03
cg07659054	7	27134225	HOXA1	0.26	6.02E-06	1.23E-03
cg02345961	18	34854574	BRUNOL4	0.28	6.02E-06	1.23E-03
cg00814328	19	22992509		-0.21	6.05E-06	1.23E-03
cg17455088	2	206551466	NRP2	0.27	6.06E-06	1.23E-03
cg23954268	5	172672454		0.30	6.06E-06	1.23E-03
cg25147604	6	7540971	DSP	0.25	6.07E-06	1.23E-03
cg18857759	1	248110661	OR2L8	-0.25	6.07E-06	1.23E-03
cg08065408	6	29520774		0.24	6.08E-06	1.23E-03
cg22120714	17	40332420	KCNH4	0.24	6.08E-06	1.23E-03
cg12285605	15	68785674		-0.21	6.11E-06	1.24E-03
cg21488066	6	28367898	ZSCAN12	0.25	6.11E-06	1.24E-03
cg22893248	7	150020751	ACTR3C	0.24	6.12E-06	1.24E-03
cg13412545	5	172175765		0.24	6.14E-06	1.24E-03
cg07727358	4	1008738	FGFRL1	-0.21	6.15E-06	1.24E-03
cg05901357	2	27529325	TRIM54	0.24	6.16E-06	1.24E-03
cg25828185	12	54455656	FLJ12825	0.20	6.20E-06	1.25E-03
cg26856578	13	79360870		-0.25	6.21E-06	1.25E-03
cg22368476	11	123814163	OR6T1	-0.25	6.23E-06	1.25E-03
cg04297067	16	86550450		0.27	6.24E-06	1.25E-03
cg18117600	15	53097649		0.23	6.25E-06	1.25E-03
cg03040165	2	131185700		0.25	6.26E-06	1.25E-03
cg22466678	12	89749033		0.24	6.29E-06	1.26E-03
cg04552470	1	20669792	VWA5B1	0.28	6.30E-06	1.26E-03
cg17636752	6	45391973	RUNX2	0.21	6.31E-06	1.26E-03
cg06388730	7	35297699		0.29	6.33E-06	1.26E-03
cg25391162	9	118091	FOXD4	0.21	6.33E-06	1.26E-03
cg23442112	11	5663329	TRIM34	-0.26	6.33E-06	1.26E-03
cg07851675	5	2755540		0.28	6.38E-06	1.27E-03
cg24563094	2	26395458	FAM59B	0.26	6.38E-06	1.27E-03
cg09042448	8	23260443	LOXL2	0.27	6.39E-06	1.27E-03
cg15304464	11	132830180	OPCML	-0.22	6.40E-06	1.27E-03
cg08144172	1	41849203		-0.22	6.40E-06	1.27E-03
cg03838635	17	2628322		0.27	6.41E-06	1.27E-03
cg26532627	17	43339744	LOC100133991	0.22	6.42E-06	1.27E-03
cg21951797	6	32026605	TNXB	-0.23	6.42E-06	1.27E-03
cg09019936	2	162283434		0.31	6.44E-06	1.27E-03

cg09247506	22	24820655		0.26	6.44E-06	1.27E-03
cg23398318	14	99664135	BCL11B	-0.25	6.47E-06	1.27E-03
cg04230699	12	12849443	GPR19	0.33	6.48E-06	1.28E-03
cg04874562	11	5462540	OR51I1	-0.21	6.49E-06	1.28E-03
cg24968786	11	66360800	CCDC87	0.23	6.49E-06	1.28E-03
cg07550362	7	97361201	TAC1	0.28	6.49E-06	1.28E-03
cg04057288	9	130504189	SH2D3C	0.21	6.51E-06	1.28E-03
cg02152475	9	37029364	PAX5	0.27	6.51E-06	1.28E-03
cg21533331	19	31848049		0.31	6.53E-06	1.28E-03
cg12912293	1	150849805	ARNT	-0.24	6.54E-06	1.28E-03
cg00288806	7	8476521	NXPH1	0.26	6.54E-06	1.28E-03
cg17566936	4	48271107	TEC	0.24	6.55E-06	1.28E-03
cg09701582	2	177017797	HOXD4	0.25	6.57E-06	1.28E-03
cg10013501	1	25257813	RUNX3	0.25	6.57E-06	1.28E-03
cg21275838	15	59608124	MYO1E	-0.23	6.61E-06	1.29E-03
cg10904986	8	145555806	SCRT1	0.23	6.65E-06	1.29E-03
cg09339763	4	174452612	NBLA00301	0.31	6.65E-06	1.29E-03
cg13600716	12	10223804	CLEC1A	-0.27	6.67E-06	1.29E-03
cg16823535	13	100611510		0.29	6.68E-06	1.29E-03
cg01497613	17	48041301		0.28	6.69E-06	1.29E-03
cg01414185	2	177017449	HOXD4	0.30	6.69E-06	1.29E-03
cg02439266	7	27150042	HOXA3	0.25	6.69E-06	1.29E-03
cg23748923	17	2628248		0.30	6.71E-06	1.29E-03
cg05353884	6	26226203		0.31	6.72E-06	1.30E-03
cg09389280	3	170141005	CLDN11	-0.20	6.73E-06	1.30E-03
cg02047547	16	19126242	ITPRIPL2	0.25	6.74E-06	1.30E-03
cg04422256	10	88123169	GRID1	0.25	6.78E-06	1.30E-03
cg19855470	22	40060836	CACNA1I	0.21	6.81E-06	1.31E-03
cg24426691	13	96204669	CLDN10	0.28	6.82E-06	1.31E-03
cg04165720	19	7677604	KIAA1543	-0.20	6.82E-06	1.31E-03
cg25774643	11	627175	SCT	0.33	6.83E-06	1.31E-03
cg04282694	6	5996412		0.27	6.84E-06	1.31E-03
cg07849581	4	48272077	TEC	0.29	6.85E-06	1.31E-03
cg11896633	8	144816335	FAM83H	0.24	6.86E-06	1.31E-03
cg10526374	11	2292525	ASCL2	0.27	6.88E-06	1.31E-03
cg23331981	10	7574780		-0.26	6.91E-06	1.32E-03
cg26275431	5	140773122	PCDHGA4	0.22	6.93E-06	1.32E-03
cg24049888	11	111250129	POU2AF1	-0.21	6.93E-06	1.32E-03
cg20732304	13	113752657	MCF2L	0.23	6.94E-06	1.32E-03
cg05033952	16	34255535		-0.25	6.95E-06	1.32E-03
cg18182148	1	92952533	GFI1	0.25	7.02E-06	1.33E-03
cg18664514	2	219851181	FEV	0.22	7.03E-06	1.33E-03
cg09997546	11	8931473	C11orf17	0.22	7.03E-06	1.33E-03
cg19548479	17	41363737	TMEM106A	0.25	7.04E-06	1.33E-03
cg24360871	7	27163929	HOXA3	0.28	7.05E-06	1.33E-03
cg06842116	22	17182794		-0.23	7.09E-06	1.34E-03
cg25616869	3	48443867		-0.22	7.10E-06	1.34E-03
cg16860712	19	48244461	EHD2	-0.23	7.11E-06	1.34E-03
cg05764839	6	32118298	PRRT1	0.25	7.11E-06	1.34E-03
cg04598224	19	11784514	ZNF833	0.24	7.14E-06	1.34E-03
cg05898591	5	174805715		-0.26	7.17E-06	1.35E-03
cg10958002	3	12200324	TIMP4	0.23	7.20E-06	1.35E-03
cg05231837	7	156411247		0.22	7.20E-06	1.35E-03
cg24138857	6	32055079	TNXB	0.24	7.21E-06	1.35E-03
cg23590202	7	27188770	HOXA6	-0.23	7.22E-06	1.36E-03
cg03400421	10	23460371		0.26	7.25E-06	1.36E-03
cg21770322	7	97807741	LMTK2	-0.24	7.29E-06	1.36E-03
cg23618344	12	54423428	HOXC4	0.26	7.29E-06	1.36E-03
cg01239735	12	214224	IQSEC3	-0.22	7.30E-06	1.36E-03
cg13053653	2	177037631	HOXD3	0.23	7.35E-06	1.37E-03
cg17497608	7	90896701	FZD1	0.29	7.37E-06	1.37E-03
cg09481121	5	78985425	CMYA5	0.24	7.40E-06	1.38E-03
cg04873577	7	121006948	FAM3C	-0.23	7.41E-06	1.38E-03
cg02750098	5	172656021		0.25	7.42E-06	1.38E-03
cg12946214	19	41882234	TMEM91	0.22	7.48E-06	1.39E-03
cg23164183	5	2754894	C5orf38	0.25	7.51E-06	1.39E-03

cg11278506	3	72787955		0.27	7.52E-06	1.39E-03
cg13031251	5	77268734		0.29	7.54E-06	1.39E-03
cg00705280	2	219866493	MIR375	0.29	7.56E-06	1.40E-03
cg20125920	10	21804624	C10orf140	0.23	7.57E-06	1.40E-03
cg27114640	1	18437320	IGSF21	0.24	7.62E-06	1.41E-03
cg20675389	3	181421951	SOX2OT	0.24	7.63E-06	1.41E-03
cg20173059	11	113185285	TTC12	0.30	7.63E-06	1.41E-03
cg26411441	20	3733040	HSPA12B	0.25	7.64E-06	1.41E-03
cg10400652	19	46996516	PNMAL2	0.25	7.68E-06	1.41E-03
cg19521832	21	35831996	KCNE1	0.27	7.72E-06	1.42E-03
cg21836370	12	85667451		0.25	7.73E-06	1.42E-03
cg18851100	22	51158550	SHANK3	0.26	7.74E-06	1.42E-03
cg02928699	11	88241946	GRM5	0.24	7.78E-06	1.42E-03
cg19058685	12	54440806	HOXC4	0.23	7.79E-06	1.42E-03
cg00282510	9	100611516		0.29	7.80E-06	1.42E-03
cg10804678	3	72788309		0.26	7.80E-06	1.42E-03
cg09357935	11	20626264	SLC6A5	0.27	7.81E-06	1.42E-03
cg13080379	1	119527156	TBX15	0.25	7.81E-06	1.42E-03
cg12646649	10	102987257	LBX1	0.31	7.82E-06	1.43E-03
cg21615583	2	219866512	MIR375	0.31	7.83E-06	1.43E-03
cg22400166	14	29242040	C14orf23	0.21	7.89E-06	1.44E-03
cg07799386	5	122430821	PRDM6	0.27	7.91E-06	1.44E-03
cg18685243	12	58015069	SLC26A10	0.26	7.91E-06	1.44E-03
cg01909921	9	135854246	GFI1B	0.24	7.96E-06	1.45E-03
cg08462924	1	41848221		-0.26	7.97E-06	1.45E-03
cg18010131	11	10476620	AMPD3	0.22	7.99E-06	1.45E-03
cg22814884	11	66360803	CCDC87	0.23	8.05E-06	1.46E-03
cg02346713	15	51387736	TNFAIP8L3	0.21	8.06E-06	1.46E-03
cg15746719	9	90113813	DAPK1	0.25	8.07E-06	1.46E-03
cg16202470	17	8907213		0.25	8.09E-06	1.46E-03
cg06368558	1	26735884	LIN28	0.23	8.09E-06	1.46E-03
cg15323626	15	35274878	ZNF770	-0.21	8.10E-06	1.46E-03
cg22616881	7	96642096	DLX6AS	0.29	8.10E-06	1.46E-03
cg16627829	19	36394482	HCST	0.21	8.12E-06	1.46E-03
cg18450930	11	5444223	OR51Q1	-0.26	8.20E-06	1.47E-03
cg02242681	2	111876361		0.23	8.21E-06	1.47E-03
cg15391433	7	98547209	TRRAP	-0.21	8.22E-06	1.48E-03
cg10323725	1	156405495		0.22	8.22E-06	1.48E-03
cg18343474	1	229566908		0.25	8.23E-06	1.48E-03
cg22378919	1	119522188	TBX15	0.28	8.23E-06	1.48E-03
cg22584618	7	35298212		0.25	8.34E-06	1.49E-03
cg05040232	3	194117403	GP5	0.20	8.35E-06	1.49E-03
cg00311984	8	145051668	PLEC1	0.21	8.41E-06	1.50E-03
cg10246081	12	49726893	C1QL4	0.26	8.41E-06	1.50E-03
cg03443455	2	171671795	GAD1	0.22	8.42E-06	1.50E-03
cg14431443	12	114846431	TBX5	0.20	8.43E-06	1.50E-03
cg25551362	22	36894069	FOXRED2	0.21	8.46E-06	1.51E-03
cg20125091	1	92952641	GFI1	0.21	8.46E-06	1.51E-03
cg05697695	19	14584181	PTGER1	0.22	8.47E-06	1.51E-03
cg02051616	5	172661132	NKX2-5	0.25	8.57E-06	1.52E-03
cg07904475	4	151500931	LRBA	0.27	8.57E-06	1.52E-03
cg06732439	2	239140318	LOC151174	0.28	8.58E-06	1.52E-03
cg15526081	10	119310404		0.23	8.58E-06	1.52E-03
cg14263391	17	37894014	GRB7	0.30	8.58E-06	1.52E-03
cg07333191	4	13526769		0.29	8.59E-06	1.52E-03
cg23928165	19	46996713	PNMAL2	0.29	8.64E-06	1.53E-03
cg06359703	1	41847297		-0.22	8.65E-06	1.53E-03
cg15396367	9	139972779	UAP1L1	0.23	8.68E-06	1.53E-03
cg18755783	13	36919905	SPG20	0.26	8.69E-06	1.53E-03
cg00064261	1	229570057	ACTA1	0.20	8.69E-06	1.53E-03
cg07997493	17	37761947	NEUROD2	0.32	8.74E-06	1.54E-03
cg03048083	17	43339497	LOC100133991	0.31	8.75E-06	1.54E-03
cg26529911	4	166795773	TLL1	0.29	8.78E-06	1.55E-03
cg21186576	17	79466077		-0.22	8.79E-06	1.55E-03
cg12991125	2	160919431	PLA2R1	0.28	8.86E-06	1.56E-03
cg03308652	3	62354991		0.26	8.89E-06	1.56E-03

cg12157438	10	94835255	CYP26A1	0.22	8.89E-06	1.56E-03
cg19264571	18	10454085	APCDD1	0.21	8.94E-06	1.56E-03
cg03159396	6	50808813	TFAP2B	0.25	8.96E-06	1.57E-03
cg09351315	17	77775823		0.25	8.97E-06	1.57E-03
cg09636715	7	27217057	HOXA10	-0.26	8.99E-06	1.57E-03
cg17206555	7	90227371		0.20	9.00E-06	1.57E-03
cg26201952	12	54446253	HOXC4	0.26	9.02E-06	1.57E-03
cg16993043	1	200008026	NR5A2	0.27	9.02E-06	1.57E-03
cg27138204	12	54446100	HOXC4	0.26	9.05E-06	1.58E-03
cg00961932	1	110691765	SLC6A17	0.22	9.05E-06	1.58E-03
cg16194233	6	108440953		0.25	9.06E-06	1.58E-03
cg04662532	12	54330125		0.21	9.08E-06	1.58E-03
cg18161956	12	115112626	TBX3	0.20	9.15E-06	1.59E-03
cg16880946	7	27188465	HOXA6	-0.21	9.17E-06	1.59E-03
cg14592399	4	41881933		0.24	9.19E-06	1.59E-03
cg03701965	19	49889044		-0.24	9.19E-06	1.59E-03
cg25204094	9	100747473	ANP32B	0.23	9.20E-06	1.59E-03
cg13106389	1	25257626	RUNX3	0.21	9.20E-06	1.59E-03
cg18963509	17	40936150	WNK4	0.23	9.22E-06	1.59E-03
cg24436715	19	31848130		0.29	9.23E-06	1.59E-03
cg21214521	7	569979		-0.22	9.26E-06	1.60E-03
cg24683414	1	92952581	GFI1	0.27	9.28E-06	1.60E-03
cg02993069	1	36786777	C1orf113	0.21	9.30E-06	1.60E-03
cg13917662	13	100548513	CLYBL	0.30	9.31E-06	1.60E-03
cg16413687	12	85672623	ALX1	0.29	9.31E-06	1.60E-03
cg17654091	19	41073716	SPTBN4	0.30	9.32E-06	1.60E-03
cg08299265	12	54354414		0.21	9.32E-06	1.60E-03
cg13604794	17	48050338	DLX4	0.20	9.33E-06	1.60E-03
cg04827692	12	63543831	AVPR1A	0.27	9.33E-06	1.60E-03
cg13637654	10	103538719		0.24	9.33E-06	1.60E-03
cg27425612	1	201368843	LAD1	0.27	9.34E-06	1.60E-03
cg18689332	12	114837666	TBX5	0.29	9.35E-06	1.60E-03
cg21336435	12	54398561		0.22	9.35E-06	1.60E-03
cg02884181	5	158689508	UBLCP1	-0.25	9.39E-06	1.60E-03
cg26562532	19	55864365	COX6B2	-0.23	9.39E-06	1.60E-03
cg16019434	18	76736785		0.21	9.39E-06	1.60E-03
cg03132556	12	54366760	HOXC11	0.28	9.40E-06	1.60E-03
cg01199603	12	65718113	MSRB3	-0.24	9.42E-06	1.61E-03
cg13478928	1	75596724	LHX8	0.30	9.42E-06	1.61E-03
cg16300419	17	36666154	ARHGAP23	0.21	9.45E-06	1.61E-03
cg08071609	6	117086040	FAM162B	0.29	9.46E-06	1.61E-03
cg26444528	13	58207859	PCDH17	0.31	9.47E-06	1.61E-03
cg24079051	2	239140485	LOC643387	0.20	9.50E-06	1.61E-03
cg03793625	2	171669862		0.26	9.53E-06	1.62E-03
cg22647546	2	26394518	FAM59B	0.21	9.54E-06	1.62E-03
cg27065003	5	122429449	PRDM6	0.27	9.54E-06	1.62E-03
cg11905488	12	10123604	CLEC12A	-0.24	9.58E-06	1.62E-03
cg06866232	11	5020582	OR51L1	-0.21	9.59E-06	1.62E-03
cg08252579	3	62359773	FEZF2	0.29	9.60E-06	1.62E-03
cg07598464	1	248366883	OR2M3	-0.22	9.65E-06	1.63E-03
cg10893278	12	54377225		0.26	9.65E-06	1.63E-03
cg12608247	2	239140369	LOC643387	0.25	9.67E-06	1.63E-03
cg09128751	21	44486205	CBS	-0.24	9.69E-06	1.63E-03
cg13672200	12	54387530		0.25	9.75E-06	1.64E-03
cg21216477	12	54440773	HOXC4	0.28	9.76E-06	1.64E-03
cg00741609	10	102893925	TLX1	0.28	9.76E-06	1.64E-03
cg11144641	5	134871566	NEUROG1	0.25	9.76E-06	1.64E-03
cg26386624	12	54366859	HOXC11	0.24	9.77E-06	1.64E-03
cg14486477	12	117256672	RNFT2	-0.21	9.77E-06	1.64E-03
cg13891768	4	111536024		0.24	9.78E-06	1.64E-03
cg02746187	15	53076188	ONECUT1	0.28	9.79E-06	1.64E-03
cg02264459	16	3199771		0.21	9.85E-06	1.64E-03
cg09887220	13	95356202		0.25	9.86E-06	1.64E-03
cg27324426	19	58220837	ZNF154	0.29	9.86E-06	1.64E-03
cg11685510	1	113286397		0.23	9.87E-06	1.64E-03
cg24360747	11	129230721		-0.24	9.93E-06	1.65E-03

cg07777790	1	229570188	ACTA1	0.25	9.98E-06	1.65E-03
cg18601714	17	25485531		-0.23	9.98E-06	1.65E-03
cg10420161	10	102899983		0.28	9.98E-06	1.65E-03
cg25850845	1	231155632	FAM89A	-0.22	9.98E-06	1.65E-03
cg23413104	2	118982263		0.26	9.99E-06	1.65E-03
cg13318787	5	50262177		0.21	1.00E-05	1.66E-03
cg07227926	12	104609526	TXNRD1	0.23	1.00E-05	1.66E-03
cg04697454	15	93633408	RGMA	0.21	1.00E-05	1.66E-03
cg06798642	4	48272082	TEC	0.29	1.01E-05	1.66E-03
cg22289831	21	38076709	SIM2	0.24	1.01E-05	1.66E-03
cg21097354	7	27179161		0.29	1.01E-05	1.66E-03
cg17229173	5	67483875		-0.24	1.01E-05	1.67E-03
cg20303399	8	26722965	ADRA1A	0.27	1.02E-05	1.67E-03
cg16556145	13	96205184	CLDN10	0.28	1.02E-05	1.68E-03
cg02940362	11	5009201	MMP26	-0.24	1.02E-05	1.68E-03
cg02729822	11	56510971	OR9G4	-0.27	1.02E-05	1.68E-03
cg13577505	22	50706248	MAPK11	0.26	1.02E-05	1.68E-03
cg13651483	19	48566587	PLA2G4C	-0.21	1.02E-05	1.68E-03
cg12140851	7	26897612	SKAP2	0.27	1.02E-05	1.68E-03
cg00947782	6	30039142	RNF39	0.28	1.03E-05	1.68E-03
cg24817358	6	29275285	OR14J1	-0.21	1.03E-05	1.68E-03
cg09867598	5	122429517	PRDM6	0.29	1.04E-05	1.70E-03
cg16277169	12	115112189	TBX3	0.22	1.04E-05	1.70E-03
cg18709382	5	42972820		0.23	1.04E-05	1.70E-03
cg16202331	4	13532655		0.26	1.05E-05	1.71E-03
cg06707343	3	62360240	FEZF2	0.23	1.05E-05	1.71E-03
cg14950829	13	53419617	PCDH8	0.28	1.05E-05	1.71E-03
cg07921625	13	58208350	PCDH17	0.27	1.06E-05	1.71E-03
cg13720705	10	37990370		0.23	1.06E-05	1.71E-03
cg03978169	6	33091357	HLA-DPB2	-0.23	1.06E-05	1.71E-03
cg13888593	2	74642470	C2orf81	0.27	1.06E-05	1.72E-03
cg23480619	21	35831871	KCNE1	0.29	1.06E-05	1.72E-03
cg23020891	3	180502503		-0.21	1.06E-05	1.72E-03
cg02274869	19	11784955	ZNF833	0.22	1.07E-05	1.72E-03
cg24641276	5	170743715		0.25	1.07E-05	1.73E-03
cg23520574	11	4928572	OR51A7	-0.28	1.08E-05	1.73E-03
cg17858328	1	92952521	GFI1	0.30	1.08E-05	1.74E-03
cg19219624	7	97362523	TAC1	0.29	1.08E-05	1.74E-03
cg09375620	6	159590155	FNDC1	0.21	1.09E-05	1.74E-03
cg06419659	1	153600132	S100A13	0.22	1.09E-05	1.74E-03
cg08005165	8	55380663		0.24	1.09E-05	1.74E-03
cg20257553	2	160919462	PLA2R1	0.23	1.09E-05	1.74E-03
cg09853898	19	591536	HCN2	0.23	1.09E-05	1.75E-03
cg15812348	3	62359390	FEZF2	0.28	1.09E-05	1.75E-03
cg07897690	2	200328645		0.22	1.10E-05	1.75E-03
cg26346796	6	32054867	TNXB	0.22	1.10E-05	1.75E-03
cg10668926	13	28527255		0.24	1.10E-05	1.75E-03
cg00249511	11	627152	SCT	0.30	1.10E-05	1.75E-03
cg06338499	18	34854300	BRUNOL4	0.25	1.10E-05	1.76E-03
cg18902978	9	6703949		0.23	1.11E-05	1.76E-03
cg10648139	2	206551364	NRP2	0.27	1.11E-05	1.76E-03
cg04290346	11	20177852	DBX1	0.30	1.11E-05	1.76E-03
cg09404551	8	121136951	COL14A1	0.23	1.11E-05	1.76E-03
cg26777456	12	54454250	FLJ12825	0.24	1.11E-05	1.76E-03
cg15382095	17	37762367	NEUROD2	0.30	1.11E-05	1.76E-03
cg24472375	6	108485102		0.31	1.11E-05	1.76E-03
cg03972745	1	4222311		-0.20	1.11E-05	1.76E-03
cg08814405	7	19183753		0.30	1.12E-05	1.77E-03
cg12492670	11	50231261		-0.23	1.12E-05	1.77E-03
cg18358869	10	102808077		0.23	1.12E-05	1.77E-03
cg26606286	4	174440471		0.27	1.13E-05	1.78E-03
cg08624472	7	98971852	ARPC1B	0.26	1.13E-05	1.78E-03
cg01151135	19	34311174		0.26	1.13E-05	1.78E-03
cg12174341	8	41424342		0.23	1.13E-05	1.78E-03
cg02339752	16	86549705		0.21	1.13E-05	1.78E-03
cg17981058	2	223176398		0.26	1.13E-05	1.78E-03

cg22683010	2	2570283		-0.21	1.13E-05	1.78E-03
cg06284231	14	38724255	CLEC14A	0.21	1.14E-05	1.78E-03
cg15831743	5	122421924		0.21	1.14E-05	1.79E-03
cg27032146	7	96652115	DLX5	0.28	1.14E-05	1.79E-03
cg23289079	5	122430476	PRDM6	0.24	1.14E-05	1.79E-03
cg10419709	17	58216651		0.28	1.14E-05	1.79E-03
cg14496753	9	124979184	LHX6	0.23	1.14E-05	1.79E-03
cg11317199	9	100850391	TRIM14	0.24	1.15E-05	1.79E-03
cg04103761	1	26735606		0.23	1.15E-05	1.80E-03
cg21475544	12	12849588	GPR19	0.26	1.16E-05	1.81E-03
cg08932654	17	38084459	ORMDL3	0.22	1.16E-05	1.81E-03
cg01297756	13	100548415	CLYBL	0.26	1.16E-05	1.81E-03
cg08747277	6	96048126	MANEA	-0.24	1.16E-05	1.81E-03
cg02334722	11	44342177		0.22	1.17E-05	1.81E-03
cg00697992	2	139537131	NXPH2	0.27	1.17E-05	1.81E-03
cg08101303	7	96652222	DLX5	0.30	1.17E-05	1.81E-03
cg10414006	16	34984121		-0.29	1.17E-05	1.81E-03
cg08949655	2	176940301		0.24	1.17E-05	1.81E-03
cg21990700	12	7260776	LOC283314	0.21	1.17E-05	1.82E-03
cg22709192	12	54366909	HOXC11	0.23	1.17E-05	1.82E-03
cg23890008	11	20229545		0.25	1.18E-05	1.83E-03
cg15877129	12	103218238		0.26	1.19E-05	1.83E-03
cg26422151	10	88136949		0.24	1.19E-05	1.83E-03
cg07831351	20	3229402		0.27	1.19E-05	1.83E-03
cg26348514	20	44098358	WFDC2	0.25	1.19E-05	1.84E-03
cg01755009	1	247978690	OR14A16	-0.22	1.20E-05	1.84E-03
cg26527487	1	99469819	LPPR5	0.24	1.20E-05	1.84E-03
cg14034325	10	43891459	HNRNPF	0.23	1.20E-05	1.85E-03
cg00303982	8	145556952	SCRT1	0.27	1.20E-05	1.85E-03
cg12590051	12	54398891		0.21	1.21E-05	1.85E-03
cg10133465	8	97166862	GDF6	0.26	1.21E-05	1.85E-03
cg14151783	20	44098409	WFDC2	0.27	1.21E-05	1.85E-03
cg10542123	10	1686470	ADARB2	-0.25	1.21E-05	1.85E-03
cg12854371	17	62776373	LOC146880	0.22	1.21E-05	1.85E-03
cg02893231	1	190444761	FAM5C	0.26	1.22E-05	1.86E-03
cg23606421	9	140330621	ENTPD8	-0.21	1.22E-05	1.86E-03
cg13530938	2	239140190	LOC151174	0.28	1.23E-05	1.87E-03
cg03585731	6	30483882		0.25	1.23E-05	1.88E-03
cg16626067	16	66613266	CMTM2	0.28	1.24E-05	1.88E-03
cg12060123	11	5668206	TRIM78P	-0.22	1.24E-05	1.88E-03
cg10745499	6	100906685	SIM1	0.27	1.24E-05	1.88E-03
cg22783363	8	23021553	TNFRSF10D	0.24	1.24E-05	1.88E-03
cg02930996	18	29265842	B4GALT6	0.26	1.24E-05	1.88E-03
cg26888836	4	48272120	TEC	0.28	1.24E-05	1.88E-03
cg13578408	7	8475484	NXPH1	0.23	1.25E-05	1.88E-03
cg16477690	18	30051572	FAM59A	0.24	1.25E-05	1.88E-03
cg05898953	10	74870240	NUDT13	0.27	1.25E-05	1.88E-03
cg15908921	17	77776587		0.27	1.25E-05	1.88E-03
cg04046669	22	30476206	HORMAD2	0.28	1.25E-05	1.88E-03
cg23291280	19	31847970		0.29	1.25E-05	1.88E-03
cg11849086	17	62776530	LOC146880	0.23	1.25E-05	1.89E-03
cg07027513	18	29265840	B4GALT6	0.23	1.25E-05	1.89E-03
cg00814752	18	56941706	RAX	0.22	1.26E-05	1.89E-03
cg02282631	5	42953543		0.29	1.27E-05	1.91E-03
cg02101486	7	96652245	DLX5	0.27	1.27E-05	1.91E-03
cg11246938	12	115112433	TBX3	0.23	1.27E-05	1.91E-03
cg23008606	8	26722933	ADRA1A	0.27	1.27E-05	1.91E-03
cg23685155	12	54396440	HOXC9	0.25	1.28E-05	1.91E-03
cg06421774	5	2066690		0.20	1.28E-05	1.91E-03
cg02824888	12	115129011		0.25	1.28E-05	1.92E-03
cg16922039	18	12287385		0.21	1.28E-05	1.92E-03
cg01460436	20	44687454	SLC12A5	0.24	1.29E-05	1.92E-03
cg16204205	18	19753432	GATA6	0.21	1.29E-05	1.92E-03
cg20027296	5	170737396	TLX3	0.23	1.29E-05	1.93E-03
cg25178784	11	64875750	C11orf2	-0.22	1.29E-05	1.93E-03
cg22268510	6	32118420	PRRT1	0.28	1.29E-05	1.93E-03

cg04010915	12	110547053		-0.21	1.29E-05	1.93E-03
cg19404832	6	50681114	TFAP2D	0.25	1.30E-05	1.93E-03
cg18988435	18	12287275		0.22	1.30E-05	1.94E-03
cg02456087	7	27291541		0.24	1.30E-05	1.94E-03
cg17104824	2	177014959	HOXD4	0.27	1.31E-05	1.94E-03
cg06640593	2	568321		-0.22	1.31E-05	1.94E-03
cg06581175	7	96632635	DLX6AS	0.27	1.31E-05	1.94E-03
cg23497383	2	239140182	LOC151174	0.27	1.32E-05	1.95E-03
cg08940787	17	62777648	LOC146880	0.25	1.32E-05	1.95E-03
cg19095187	6	108437051		0.26	1.32E-05	1.95E-03
cg01216882	20	31804277	C20orf71	-0.21	1.33E-05	1.96E-03
cg02027878	7	150711138	ATG9B	0.25	1.33E-05	1.96E-03
cg16108230	6	117584815		0.24	1.35E-05	1.98E-03
cg26099134	6	100897309	SIM1	0.20	1.35E-05	1.98E-03
cg13160874	2	242549431	THAP4	0.23	1.35E-05	1.98E-03
cg05121480	3	187387601	SST	0.27	1.35E-05	1.98E-03
cg19519747	2	177014555	MIR10B	0.23	1.36E-05	1.98E-03
cg05774915	3	27755417		0.31	1.37E-05	2.00E-03
cg18042724	11	20627327	SLC6A5	0.24	1.37E-05	2.00E-03
cg21127537	11	113184936	TTC12	0.26	1.37E-05	2.00E-03
cg16110759	12	54330320		0.23	1.37E-05	2.00E-03
cg19657174	22	19746268	TBX1	0.30	1.37E-05	2.00E-03
cg09744068	10	125732787		0.23	1.37E-05	2.00E-03
cg08454824	8	637468	ERICH1	0.21	1.38E-05	2.01E-03
cg04622024	1	38201001	EPHA10	0.22	1.39E-05	2.01E-03
cg11571761	5	95298041	ELL2	0.22	1.39E-05	2.02E-03
cg09737314	17	6899359	ALOX12	0.25	1.39E-05	2.02E-03
cg03094519	17	37761662	NEUROD2	0.21	1.39E-05	2.02E-03
cg01438291	11	71567175		-0.22	1.39E-05	2.02E-03
cg02787917	2	175190821		0.24	1.40E-05	2.02E-03
cg18007463	4	166795913	TLL1	0.24	1.40E-05	2.02E-03
cg21332729	5	95170798		0.28	1.40E-05	2.02E-03
cg21332500	7	27233480		0.21	1.40E-05	2.02E-03
cg25100880	11	5878019	OR52E8	-0.22	1.40E-05	2.02E-03
cg19164987	12	54413000	HOXC4	0.26	1.40E-05	2.02E-03
cg23037321	1	181003203	MR1	-0.27	1.42E-05	2.04E-03
cg02078520	1	84944298	RPF1	-0.22	1.42E-05	2.04E-03
cg22306928	2	219866424	MIR375	0.26	1.42E-05	2.04E-03
cg20521702	3	98452812	ST3GAL6	0.25	1.42E-05	2.04E-03
cg27346037	6	106442129		0.21	1.42E-05	2.04E-03
cg10113212	14	92790798	SLC24A4	0.21	1.42E-05	2.04E-03
cg26263234	3	26666060	LRRC3B	0.27	1.43E-05	2.05E-03
cg13455326	19	10043341	OLFM2	-0.25	1.43E-05	2.05E-03
cg13677149	7	27284789	EVX1	0.29	1.44E-05	2.06E-03
cg06825512	18	10453969	APCDD1	0.21	1.44E-05	2.06E-03
cg17304496	2	37572289	QPCT	0.27	1.45E-05	2.07E-03
cg27339437	10	16478863	PTER	0.27	1.45E-05	2.07E-03
cg07747336	11	125649398	PATE2	-0.20	1.47E-05	2.09E-03
cg08873424	6	28945492		0.22	1.47E-05	2.09E-03
cg14677698	5	3197730		0.26	1.47E-05	2.09E-03
cg25310824	5	42812334	SEPP1	-0.25	1.47E-05	2.09E-03
cg25596754	6	32026610	TNXB	-0.22	1.47E-05	2.09E-03
cg07104958	10	46168551	ANUBL1	0.20	1.48E-05	2.09E-03
cg19416570	19	58715677	ZNF274	0.22	1.48E-05	2.10E-03
cg16547027	18	24127588	KCTD1	0.28	1.48E-05	2.10E-03
cg05000331	16	79623678		0.24	1.49E-05	2.10E-03
cg13719746	9	100747559	ANP32B	0.29	1.49E-05	2.10E-03
cg03618016	1	247769804	OR2G3	-0.27	1.49E-05	2.11E-03
cg07636194	6	27107282	HIST1H2BK	0.31	1.49E-05	2.11E-03
cg06666025	16	66613278	CMTM2	0.27	1.50E-05	2.11E-03
cg07485198	8	95246699		0.21	1.50E-05	2.12E-03
cg00481951	3	187387650	SST	0.25	1.53E-05	2.14E-03
cg21100077	13	100627366		0.28	1.53E-05	2.14E-03
cg11375458	2	177003996		0.24	1.53E-05	2.14E-03
cg11329058	17	78549358	RPTOR	0.22	1.53E-05	2.14E-03
cg12296751	7	116166734	CAV1	0.23	1.53E-05	2.14E-03

cg02335804	17	36105239	HNF1B	0.22	1.54E-05	2.15E-03
cg03555836	8	41422764		0.21	1.55E-05	2.15E-03
cg20432507	20	21689861	PAX1	0.23	1.55E-05	2.15E-03
cg00976453	20	48099479	KCNB1	0.28	1.55E-05	2.15E-03
cg17160382	6	121758980	GJA1	0.30	1.55E-05	2.16E-03
cg04413320	13	58208929	PCDH17	0.27	1.55E-05	2.16E-03
cg27606396	2	113542117	IL1A	-0.26	1.55E-05	2.16E-03
cg11236429	4	174459412	NBLA00301	0.26	1.55E-05	2.16E-03
cg15684962	6	99273315		0.24	1.55E-05	2.16E-03
cg07323141	2	239140169	LOC151174	0.24	1.55E-05	2.16E-03
cg14983771	14	61201189	MNAT1	-0.21	1.57E-05	2.17E-03
cg10411590	13	21900810		0.21	1.57E-05	2.18E-03
cg11051055	8	11058145	XKR6	0.29	1.58E-05	2.18E-03
cg22352078	8	41424982		0.26	1.58E-05	2.18E-03
cg06456154	10	119310356		0.21	1.58E-05	2.18E-03
cg25576818	7	27233141		0.25	1.59E-05	2.19E-03
cg07753583	7	150020206	LRRC61	0.20	1.59E-05	2.19E-03
cg27505504	15	32964155	SCG5	-0.22	1.61E-05	2.20E-03
cg04424930	10	43892790	HNRNPF	0.23	1.61E-05	2.20E-03
cg17634797	6	75945095		-0.20	1.61E-05	2.21E-03
cg22849665	6	32118375	PRRT1	0.25	1.61E-05	2.21E-03
cg00014998	2	177014962	HOXD4	0.27	1.62E-05	2.21E-03
cg26554505	3	55516711	WNT5A	0.25	1.62E-05	2.21E-03
cg25730298	1	245581404	KIF26B	-0.25	1.62E-05	2.21E-03
cg02077084	1	248549941	OR2T6	-0.23	1.62E-05	2.21E-03
cg03278726	1	1109832	TTLL10	0.22	1.62E-05	2.21E-03
cg09183124	1	173092323		0.20	1.62E-05	2.21E-03
cg04676482	4	111536216		0.21	1.63E-05	2.22E-03
cg20229534	6	10882250	GCM2	0.29	1.64E-05	2.23E-03
cg21063716	11	2292787	ASCL2	0.21	1.64E-05	2.23E-03
cg12710648	1	7308091	CAMTA1	-0.25	1.64E-05	2.23E-03
cg08053137	12	54385436	MIR196A2	0.24	1.65E-05	2.23E-03
cg01392518	6	166583226	T	0.20	1.65E-05	2.23E-03
cg15347342	6	108490485	NR2E1	0.25	1.65E-05	2.24E-03
cg26645401	11	9686514	SWAP70	0.26	1.66E-05	2.25E-03
cg05408649	12	54446019	HOXC4	0.30	1.67E-05	2.25E-03
cg14207728	1	67217886	TCTEX1D1	0.25	1.67E-05	2.25E-03
cg04032071	12	54376241		0.20	1.67E-05	2.26E-03
cg09422450	16	79623618		0.26	1.68E-05	2.26E-03
cg01229998	11	60049447	MS4A4A	-0.21	1.68E-05	2.26E-03
cg06630413	12	54366875	HOXC11	0.25	1.68E-05	2.26E-03
cg07216425	12	53297819	KRT8	0.24	1.68E-05	2.26E-03
cg11847222	5	77268624		0.26	1.69E-05	2.27E-03
cg23712018	6	30039132	RNF39	0.24	1.69E-05	2.27E-03
cg15732658	6	10410042	TFAP2A	0.22	1.70E-05	2.28E-03
cg16057826	21	36041334	CLIC6	0.22	1.71E-05	2.29E-03
cg15828364	11	20626786	SLC6A5	0.23	1.71E-05	2.29E-03
cg23385248	15	93579332		0.23	1.71E-05	2.29E-03
cg24961583	3	27765409		0.32	1.72E-05	2.29E-03
cg19403541	4	174438312		0.29	1.72E-05	2.30E-03
cg04707408	7	27233073		0.26	1.72E-05	2.30E-03
cg08178940	6	50808627	TFAP2B	0.28	1.73E-05	2.30E-03
cg09656020	15	33009409	GREM1	0.22	1.73E-05	2.31E-03
cg00303183	2	162283559		0.24	1.74E-05	2.32E-03
cg00690271	12	49658716	TUBA1C	0.25	1.74E-05	2.32E-03
cg15234400	4	174453166	NBLA00301	0.21	1.75E-05	2.32E-03
cg13278353	2	208635942		0.22	1.75E-05	2.32E-03
cg03423957	22	31555670	RNF185	-0.23	1.75E-05	2.32E-03
cg05307533	7	27531652		-0.20	1.76E-05	2.33E-03
cg01663018	15	53097777		0.28	1.76E-05	2.33E-03
cg09325156	12	46660749	SLC38A1	0.26	1.76E-05	2.33E-03
cg09577115	20	21497180		0.20	1.77E-05	2.34E-03
cg06232130	2	157177008		0.23	1.77E-05	2.34E-03
cg13774019	9	37029805	PAX5	0.23	1.77E-05	2.34E-03
cg03557857	10	103589693	KCNIP2	0.23	1.77E-05	2.34E-03
cg27160395	2	177014949	HOXD4	0.25	1.77E-05	2.34E-03

cg12097325	1	1076431		0.21	1.78E-05	2.34E-03
cg00169725	5	176543901		0.22	1.78E-05	2.35E-03
cg10024909	4	174452985	NBLA00301	0.23	1.78E-05	2.35E-03
cg10604851	12	103344336		0.22	1.79E-05	2.35E-03
cg05852276	5	140579470	PCDHB11	0.22	1.79E-05	2.35E-03
cg16971668	3	27764810	EOMES	0.29	1.80E-05	2.36E-03
cg10188253	19	43908315	TEX101	-0.21	1.80E-05	2.36E-03
cg13718418	12	85672285		0.24	1.80E-05	2.36E-03
cg00674679	2	176931680		0.26	1.80E-05	2.36E-03
cg10881225	2	9984929	TAF1B	0.21	1.81E-05	2.37E-03
cg25845367	13	100627175		0.32	1.82E-05	2.38E-03
cg12749132	1	151811364	LOC100132111	0.31	1.82E-05	2.38E-03
cg17935422	4	2733397	FAM193A	-0.22	1.82E-05	2.38E-03
cg21237687	17	6899380	ALOX12	0.24	1.82E-05	2.38E-03
cg02397571	8	28175463	PNOC	-0.22	1.83E-05	2.38E-03
cg08614290	7	158938491	VIPR2	0.29	1.83E-05	2.38E-03
cg10077468	15	91498367	RCCD1	0.26	1.83E-05	2.39E-03
cg06680397	5	135527376	LOC389332	0.24	1.83E-05	2.39E-03
cg01405004	3	68980539	FAM19A4	0.22	1.86E-05	2.41E-03
cg06148812	22	19746368	TBX1	0.25	1.86E-05	2.41E-03
cg12307823	15	67143737		0.22	1.86E-05	2.42E-03
cg16297569	1	92952517	GFI1	0.28	1.86E-05	2.42E-03
cg03915638	3	62356042	FEZF2	0.23	1.87E-05	2.42E-03
cg02393380	20	1304919	SDCBP2	-0.22	1.88E-05	2.43E-03
cg17866732	2	110372875	Sep-10	0.22	1.88E-05	2.43E-03
cg08158662	12	34489995		-0.21	1.88E-05	2.43E-03
cg06248560	18	35104681	BRUNOL4	0.24	1.88E-05	2.43E-03
cg25301173	2	190984844		-0.22	1.89E-05	2.43E-03
cg05713954	16	49518536		0.23	1.89E-05	2.43E-03
cg11012584	2	242549943	THAP4	0.21	1.89E-05	2.43E-03
cg13694927	7	27184712	HOXA5	0.21	1.89E-05	2.43E-03
cg00527440	2	242549864	THAP4	0.27	1.89E-05	2.43E-03
cg21027124	4	144831061		0.21	1.90E-05	2.45E-03
cg06396119	13	49792767		0.21	1.91E-05	2.45E-03
cg07495357	6	43021181	CUL7	0.27	1.91E-05	2.45E-03
cg01571001	3	128215433		0.31	1.91E-05	2.45E-03
cg26492368	10	22634733	SPAG6	0.25	1.91E-05	2.45E-03
cg23244790	5	140811102	PCDHGA4	0.21	1.92E-05	2.46E-03
cg11668749	1	229568360	ACTA1	0.23	1.93E-05	2.47E-03
cg08468911	21	35832180	KCNE1	0.25	1.93E-05	2.47E-03
cg07553761	3	160167977	TRIM59	0.21	1.95E-05	2.49E-03
cg03730533	7	26897685	SKAP2	0.27	1.95E-05	2.49E-03
cg02128244	19	31848008		0.25	1.95E-05	2.49E-03
cg09502207	8	133774222	TMEM71	-0.21	1.95E-05	2.49E-03
cg17204129	7	117119601	CFTR	0.26	1.95E-05	2.49E-03
cg18771937	12	54385517	MIR196A2	0.21	1.96E-05	2.49E-03
cg10517650	3	113235015	CCDC52	-0.24	1.96E-05	2.49E-03
cg09854734	16	66613273	CMTM2	0.26	1.96E-05	2.49E-03
cg19815327	3	32023353	ZNF860	0.22	1.97E-05	2.50E-03
cg01344452	17	54912143	DGKE	0.28	1.97E-05	2.50E-03
cg19961480	6	88877216	CNR1	0.26	1.97E-05	2.50E-03
cg01581974	12	49658713	TUBA1C	0.27	1.97E-05	2.50E-03
cg12390344	18	21270348	LAMA3	0.20	1.97E-05	2.50E-03
cg12569013	8	11549572		0.21	1.97E-05	2.50E-03
cg20817902	4	81124121	PRDM8	0.26	1.98E-05	2.51E-03
cg14314744	6	100897445	SIM1	0.23	1.98E-05	2.51E-03
cg05179921	12	9905753	CD69	-0.25	1.98E-05	2.51E-03
cg03722396	4	111537907		0.28	1.98E-05	2.51E-03
cg20232746	4	174440598		0.23	1.98E-05	2.51E-03
cg18575740	4	174457224	NBLA00301	0.24	1.99E-05	2.51E-03
cg04317962	16	79623625		0.25	2.00E-05	2.52E-03
cg15083015	11	20625714	SLC6A5	0.25	2.01E-05	2.54E-03
cg16764274	2	223290168	SGPP2	0.24	2.01E-05	2.54E-03
cg26963367	15	89157841		0.22	2.02E-05	2.54E-03
cg04473302	7	107301217	SLC26A4	0.22	2.02E-05	2.54E-03
cg02081733	15	68120412	LBXCOR1	0.25	2.03E-05	2.55E-03

cg26310483	6	30421376		0.22	2.03E-05	2.55E-03
cg10414946	11	59856358	MS4A2	-0.21	2.03E-05	2.55E-03
cg20380689	5	37836392	GDNF	0.21	2.03E-05	2.55E-03
cg20540714	6	10415873	TFAP2A	0.22	2.03E-05	2.55E-03
cg14501219	17	62775713	LOC146880	0.21	2.04E-05	2.55E-03
cg03407747	17	6899364	ALOX12	0.25	2.04E-05	2.56E-03
cg07024458	16	56390600	GNAO1	0.22	2.04E-05	2.56E-03
cg09803262	7	96622608	DLX6AS	0.28	2.04E-05	2.56E-03
cg05361489	16	86610434		0.23	2.05E-05	2.56E-03
cg10265690	5	170744027		0.24	2.05E-05	2.56E-03
cg23767994	7	96621323	DLX6AS	0.22	2.06E-05	2.57E-03
cg09965996	16	56390429	GNAO1	0.25	2.08E-05	2.59E-03
cg01445809	20	48099468	KCNB1	0.23	2.08E-05	2.59E-03
cg24005685	4	111549880	PITX2	0.29	2.08E-05	2.59E-03
cg15540138	4	166795622	TLL1	0.28	2.10E-05	2.61E-03
cg20399509	21	47717575	C21orf57	0.24	2.11E-05	2.62E-03
cg26640601	6	10883866		0.24	2.14E-05	2.64E-03
cg02179455	7	19183732		0.29	2.14E-05	2.64E-03
cg16236672	13	23949979	SACS	0.21	2.14E-05	2.64E-03
cg13558371	9	126135610	CRB2	-0.22	2.14E-05	2.64E-03
cg10601940	2	223159305	PAX3	0.20	2.16E-05	2.66E-03
cg21272465	2	239140296	LOC151174	0.26	2.16E-05	2.66E-03
cg00738309	13	114748624	RASA3	-0.24	2.16E-05	2.66E-03
cg22451910	7	100701852	MUC17	0.20	2.16E-05	2.66E-03
cg12788467	17	36105335	HNF1B	0.22	2.17E-05	2.66E-03
cg15899032	7	98547122	TRRAP	-0.24	2.17E-05	2.66E-03
cg22595460	2	119617128		0.20	2.17E-05	2.67E-03
cg05848545	17	29335528		0.23	2.18E-05	2.67E-03
cg18698767	10	128213855		-0.23	2.18E-05	2.67E-03
cg06829968	15	53076235	ONECUT1	0.23	2.19E-05	2.68E-03
cg27313642	8	65291542	MIR124-2	0.22	2.19E-05	2.68E-03
cg27149471	11	56043514	OR5T1	-0.21	2.19E-05	2.68E-03
cg24205065	11	50238107		-0.25	2.20E-05	2.69E-03
cg00161955	8	82608057	SLC10A5	-0.21	2.21E-05	2.69E-03
cg17922359	4	13530923		0.28	2.21E-05	2.69E-03
cg01609275	14	37049091		0.24	2.21E-05	2.69E-03
cg02387679	5	75699865	IQGAP2	0.23	2.22E-05	2.69E-03
cg22383888	3	27764816	EOMES	0.29	2.22E-05	2.70E-03
cg17802949	22	40060891	CACNA1I	0.20	2.22E-05	2.70E-03
cg18543610	13	100627929		0.27	2.23E-05	2.70E-03
cg03783663	13	70682628	KLHL1	0.21	2.23E-05	2.71E-03
cg24690731	8	10589093	SOX7	0.24	2.23E-05	2.71E-03
cg00508975	3	26666151	LRRC3B	0.20	2.24E-05	2.71E-03
cg05224741	6	10422322		0.33	2.24E-05	2.71E-03
cg09714919	16	85981947		0.21	2.25E-05	2.72E-03
cg18002116	13	53419596	PCDH8	0.26	2.25E-05	2.72E-03
cg05873146	17	29335669		0.27	2.25E-05	2.72E-03
cg21300072	12	12850082	GPR19	0.28	2.25E-05	2.72E-03
cg18105665	14	93257417		-0.23	2.26E-05	2.72E-03
cg17824240	10	43892819	HNRNPF	0.23	2.26E-05	2.73E-03
cg18930910	6	30039151	RNF39	0.22	2.26E-05	2.73E-03
cg00965023	19	11785188	ZNF833	0.21	2.27E-05	2.73E-03
cg15437053	19	34311123		0.26	2.27E-05	2.74E-03
cg14142965	12	104609432	TXNRD1	0.26	2.28E-05	2.74E-03
cg13826247	12	54424203	HOXC4	0.24	2.28E-05	2.74E-03
cg10180281	7	26897714	SKAP2	0.23	2.30E-05	2.76E-03
cg01308409	13	33591937	KL	0.22	2.30E-05	2.76E-03
cg03019360	16	61330754		-0.21	2.30E-05	2.76E-03
cg03293507	6	30038975	RNF39	0.25	2.31E-05	2.76E-03
cg10297473	16	51184152	SALL1	0.20	2.31E-05	2.77E-03
cg02892898	1	53169070		0.22	2.31E-05	2.77E-03
cg22282405	6	50810682	TFAP2B	0.27	2.32E-05	2.77E-03
cg01550307	17	36204463	LOC284100	0.21	2.32E-05	2.77E-03
cg22697853	1	147737207		0.22	2.33E-05	2.78E-03
cg07217341	2	3128823		-0.22	2.33E-05	2.78E-03
cg02211735	11	5142602	OR52A4	-0.23	2.33E-05	2.78E-03

cg14684709	13	100547249	CLYBL	0.26	2.34E-05	2.79E-03
cg20691140	2	137522667		0.22	2.34E-05	2.79E-03
cg17800654	1	50888590	DMRTA2	0.28	2.34E-05	2.79E-03
cg17755352	9	127215714	GPR144	0.24	2.34E-05	2.79E-03
cg04451736	5	10333413		0.26	2.35E-05	2.79E-03
cg09784335	4	41875193		0.20	2.36E-05	2.81E-03
cg09032310	5	141262801		0.23	2.36E-05	2.81E-03
cg08802841	12	89748726		0.22	2.37E-05	2.81E-03
cg12124386	1	190444776	FAM5C	0.28	2.37E-05	2.81E-03
cg24757553	7	27127501		0.23	2.37E-05	2.81E-03
cg19451167	7	96655981		0.20	2.38E-05	2.82E-03
cg16705744	3	27756039		0.25	2.38E-05	2.82E-03
cg03477792	4	77819574	ANKRD56	0.22	2.39E-05	2.82E-03
cg18786593	2	157178731		0.28	2.41E-05	2.84E-03
cg02876925	4	77869590	Sep-11	0.20	2.41E-05	2.85E-03
cg01257975	22	40058706	CACNA1I	0.21	2.41E-05	2.85E-03
cg17718302	6	27858637	HIST1H3J	0.27	2.41E-05	2.85E-03
cg04468543	5	77268331		0.22	2.42E-05	2.85E-03
cg26330518	8	24770342	NEFM	0.27	2.42E-05	2.85E-03
cg05825244	20	2730488	EBF4	0.29	2.42E-05	2.85E-03
cg25761793	1	63782639		0.23	2.42E-05	2.85E-03
cg03639749	12	133562363	ZNF26	-0.23	2.43E-05	2.86E-03
cg18031675	7	29602763	PRR15	0.21	2.44E-05	2.87E-03
cg22764436	2	223168727	CCDC140	0.22	2.45E-05	2.88E-03
cg05649108	18	34854722	BRUNOL4	0.31	2.46E-05	2.88E-03
cg03711485	10	101296036	NKX2-3	0.25	2.47E-05	2.89E-03
cg26719629	11	2163299	IGF2AS	0.20	2.47E-05	2.90E-03
cg03341413	15	68125566	LBXCOR1	0.25	2.49E-05	2.91E-03
cg26800915	11	5324199	OR51B4	-0.26	2.50E-05	2.92E-03
cg12041387	7	96650171	DLX5	0.25	2.51E-05	2.93E-03
cg00700485	20	44098223	WFDC2	0.21	2.51E-05	2.93E-03
cg07888234	18	59992252	TNFRSF11A	0.20	2.51E-05	2.93E-03
cg05690394	14	73958888	HEATR4	0.28	2.53E-05	2.94E-03
cg15244786	12	54446279	HOXC4	0.24	2.53E-05	2.94E-03
cg04436818	10	102589545	PAX2	0.28	2.53E-05	2.94E-03
cg09935994	2	235406540	ARL4C	0.20	2.53E-05	2.95E-03
cg26960333	13	92051705	GPC5	0.21	2.54E-05	2.95E-03
cg24385632	11	16838615	PLEKHA7	-0.21	2.55E-05	2.96E-03
cg26825532	3	62355103		0.21	2.55E-05	2.96E-03
cg05328547	7	19185139	FERD3L	0.28	2.58E-05	2.99E-03
cg10572355	1	119549145		0.26	2.58E-05	2.99E-03
cg14430542	9	79637457		0.21	2.59E-05	2.99E-03
cg03982801	7	27168688	HOXA4	0.20	2.59E-05	2.99E-03
cg12736877	6	30039010	RNF39	0.28	2.59E-05	2.99E-03
cg09236434	2	99553642	C2orf55	0.24	2.59E-05	2.99E-03
cg22609784	4	4863678	MSX1	0.23	2.59E-05	2.99E-03
cg25756406	10	134597884		0.21	2.60E-05	2.99E-03
cg05592245	10	65280473	REEP3	0.22	2.60E-05	3.00E-03
cg07681935	4	174452461	NBLA00301	0.30	2.61E-05	3.00E-03
cg16559708	1	7130495	CAMTA1	0.21	2.62E-05	3.01E-03
cg02998620	2	223171473		0.24	2.63E-05	3.02E-03
cg07882836	6	43276573	CRIP3	0.26	2.63E-05	3.02E-03
cg00170003	6	108490371	NR2E1	0.28	2.64E-05	3.03E-03
cg02743713	2	157178647		0.26	2.64E-05	3.03E-03
cg16495212	15	83317306	CPEB1	0.21	2.65E-05	3.03E-03
cg09164580	8	97157878	GDF6	0.22	2.65E-05	3.03E-03
cg19990785	11	67232403	TMEM134	0.22	2.65E-05	3.04E-03
cg00213052	18	77397557		0.20	2.66E-05	3.04E-03
cg19929355	4	48272059	TEC	0.24	2.66E-05	3.04E-03
cg05380916	3	179437788	USP13	-0.22	2.67E-05	3.04E-03
cg00215432	2	219866437	MIR375	0.30	2.67E-05	3.04E-03
cg19674065	4	26858928		0.20	2.67E-05	3.04E-03
cg23429510	2	176936942		0.22	2.67E-05	3.04E-03
cg14429204	1	16553327		0.22	2.67E-05	3.04E-03
cg05036656	4	41875470		0.27	2.68E-05	3.05E-03
cg22512438	3	129693489	TRH	0.27	2.69E-05	3.06E-03

cg22533683	17	29887049	MIR193A	0.23	2.69E-05	3.06E-03
cg16345311	16	26505221		-0.21	2.70E-05	3.06E-03
cg22735222	10	101295724	NKX2-3	0.28	2.70E-05	3.06E-03
cg13036381	3	159942495	LOC401097	0.20	2.70E-05	3.06E-03
cg06740893	1	12251869	TNFRSF1B	-0.21	2.71E-05	3.07E-03
cg01049674	19	41073425	SPTBN4	0.21	2.72E-05	3.08E-03
cg16137147	1	221067896		0.29	2.73E-05	3.09E-03
cg15262505	7	1428806		0.20	2.74E-05	3.09E-03
cg21146503	11	20178677	DBX1	0.26	2.74E-05	3.09E-03
cg13219590	14	97492191		0.21	2.74E-05	3.09E-03
cg04917276	17	36104585	HNF1B	0.22	2.75E-05	3.09E-03
cg09317554	4	151505084	LRBA	0.24	2.75E-05	3.09E-03
cg20603637	8	42010142	AP3M2	0.25	2.76E-05	3.11E-03
cg12848808	15	68125599	LBXCOR1	0.27	2.77E-05	3.11E-03
cg00142402	17	75956190		0.22	2.78E-05	3.11E-03
cg16991515	6	27107019	HIST1H2BK	0.30	2.78E-05	3.11E-03
cg15611809	5	67483833		-0.20	2.79E-05	3.12E-03
cg04322572	10	43447618		0.20	2.80E-05	3.13E-03
cg09175843	2	70313417	PCBP1	0.26	2.80E-05	3.13E-03
cg01644927	6	88877413		0.22	2.80E-05	3.13E-03
cg20815297	4	13541663		0.21	2.80E-05	3.13E-03
cg25032595	13	96204978	CLDN10	0.30	2.81E-05	3.13E-03
cg12262378	17	6899522	ALOX12	0.24	2.82E-05	3.14E-03
cg27631599	1	1108820	TTLL10	0.21	2.83E-05	3.15E-03
cg10239163	2	25527366	DNMT3A	0.22	2.84E-05	3.15E-03
cg01757168	3	128215565		0.27	2.84E-05	3.15E-03
cg07362328	1	50886393	DMRTA2	0.21	2.84E-05	3.15E-03
cg27615095	12	63049980	PPM1H	-0.22	2.85E-05	3.15E-03
cg18294646	5	33937876	RXFP3	0.20	2.87E-05	3.17E-03
cg12258228	12	88866007		-0.21	2.87E-05	3.17E-03
cg23291886	4	174440681		0.26	2.87E-05	3.17E-03
cg11221524	1	84969091	GNG5	0.20	2.88E-05	3.17E-03
cg20704772	4	53617882		0.23	2.88E-05	3.17E-03
cg06014401	3	27765232	EOMES	0.30	2.89E-05	3.18E-03
cg22512163	5	73205678	RGNEF	0.23	2.90E-05	3.19E-03
cg08823110	10	102589840		0.25	2.90E-05	3.19E-03
cg23136139	10	43697918	RASGEF1A	0.22	2.91E-05	3.20E-03
cg19559537	17	36204489	LOC284100	0.25	2.91E-05	3.20E-03
cg16886987	21	38378634	DSCR6	0.26	2.91E-05	3.20E-03
cg24967701	17	37894165	GRB7	0.24	2.92E-05	3.20E-03
cg16611654	15	68113386		0.24	2.93E-05	3.21E-03
cg26465391	19	58220773	ZNF154	0.27	2.93E-05	3.21E-03
cg04153838	12	115132015		0.22	2.94E-05	3.22E-03
cg21964551	1	201368791	LAD1	0.24	2.96E-05	3.23E-03
cg05722981	4	111533509		0.26	2.96E-05	3.24E-03
cg18770231	1	102119095		0.22	2.96E-05	3.24E-03
cg15591930	8	61765391	CHD7	-0.25	2.97E-05	3.24E-03
cg10276465	22	42470494	FAM109B	0.24	2.98E-05	3.24E-03
cg21697851	21	38076869	SIM2	0.27	2.98E-05	3.25E-03
cg25266629	10	102894148	TLX1	0.28	2.98E-05	3.25E-03
cg05185040	8	41425077		0.21	2.99E-05	3.25E-03
cg17535691	10	102881324	TLX1NB	0.30	2.99E-05	3.25E-03
cg07197092	2	79008089		-0.23	2.99E-05	3.25E-03
cg12352124	15	53097840		0.25	3.00E-05	3.26E-03
cg00254681	6	32118457	PRRT1	0.23	3.01E-05	3.26E-03
cg07475394	17	79466438		-0.21	3.01E-05	3.26E-03
cg11391335	1	119521928	TBX15	0.25	3.01E-05	3.26E-03
cg22465516	17	54912286	DGKE	0.25	3.04E-05	3.28E-03
cg01804925	6	27107588	HIST1H2BK	0.29	3.05E-05	3.29E-03
cg08681924	3	62359924	FEZF2	0.28	3.05E-05	3.29E-03
cg05573844	12	54354590		0.24	3.07E-05	3.31E-03
cg00434010	6	100903691	SIM1	0.25	3.08E-05	3.32E-03
cg17031543	4	52918471	SPATA18	0.22	3.10E-05	3.33E-03
cg20390613	1	12678355	DHRS3	0.23	3.10E-05	3.33E-03
cg24008908	1	19615453	AKR7A3	0.20	3.11E-05	3.33E-03
cg25386707	10	131748428	EBF3	0.24	3.14E-05	3.36E-03

cg03155112	7	47556928	TNS3	-0.23	3.15E-05	3.37E-03
cg09766710	1	247767976	OR2G3	-0.24	3.16E-05	3.38E-03
cg13783434	13	100610616		0.21	3.16E-05	3.38E-03
cg12541454	4	174437915		0.24	3.17E-05	3.38E-03
cg19849728	4	111536767		0.24	3.17E-05	3.39E-03
cg19605788	19	40729386	CNTD2	0.23	3.18E-05	3.39E-03
cg13783725	6	117589651	VGLL2	0.22	3.18E-05	3.39E-03
cg05055001	14	30649039		-0.23	3.18E-05	3.39E-03
cg03614916	18	22007271	IMPACT	0.23	3.20E-05	3.41E-03
cg01085362	19	31848310		0.25	3.21E-05	3.41E-03
cg25610702	6	43276744	CRIP3	0.20	3.21E-05	3.42E-03
cg01709766	11	5640318	TRIM6-TRIM34	-0.20	3.22E-05	3.42E-03
cg10095226	1	201368738	LAD1	0.26	3.22E-05	3.42E-03
cg10972873	1	200007592	NR5A2	0.21	3.22E-05	3.42E-03
cg02079831	12	58014709	SLC26A10	0.20	3.23E-05	3.42E-03
cg02919615	19	1295656	EFNA2	-0.21	3.23E-05	3.42E-03
cg20885782	4	41875606		0.26	3.24E-05	3.43E-03
cg18920097	15	35045695	GJD2	0.23	3.24E-05	3.43E-03
cg10954469	2	71115370		0.27	3.24E-05	3.43E-03
cg02466602	17	16290349		0.22	3.25E-05	3.43E-03
cg23490887	6	117584596		0.25	3.25E-05	3.44E-03
cg24426405	7	16844852	AGR2	-0.22	3.26E-05	3.44E-03
cg19179099	10	106390667		-0.25	3.26E-05	3.44E-03
cg11528849	2	182542901	NEUROD1	0.29	3.26E-05	3.44E-03
cg00743094	13	100547968	CLYBL	0.30	3.26E-05	3.44E-03
cg11107212	7	98100224		0.25	3.26E-05	3.44E-03
cg02471153	8	99440331	KCNS2	0.21	3.28E-05	3.45E-03
cg24851854	1	59042275	TACSTD2	0.26	3.28E-05	3.45E-03
cg21781979	13	100547292	CLYBL	0.30	3.28E-05	3.45E-03
cg22724854	11	55737072	OR10AG1	-0.20	3.29E-05	3.46E-03
cg05952572	11	72295425	PDE2A	0.24	3.29E-05	3.46E-03
cg17199800	7	96632777	DLX6AS	0.25	3.30E-05	3.46E-03
cg08520746	2	172945932		0.23	3.30E-05	3.46E-03
cg26251506	7	96642163	DLX6AS	0.25	3.34E-05	3.50E-03
cg02537491	12	54354162		0.22	3.35E-05	3.50E-03
cg21367972	7	116166824	CAV1	0.21	3.35E-05	3.50E-03
cg12464395	11	41644475		-0.20	3.36E-05	3.51E-03
cg10243196	19	23945996	RPSAP58	0.20	3.36E-05	3.51E-03
cg04502985	5	77268452		0.26	3.38E-05	3.52E-03
cg00860379	15	53097822		0.24	3.40E-05	3.52E-03
cg06764239	1	119544772		0.23	3.40E-05	3.53E-03
cg19861741	5	170742608		0.25	3.41E-05	3.53E-03
cg26330116	15	37173000	LOC145845	0.26	3.41E-05	3.53E-03
cg27053299	13	100548780	CLYBL	0.29	3.41E-05	3.53E-03
cg26772540	19	11784672	ZNF833	0.20	3.43E-05	3.55E-03
cg05282518	14	20344920	OR4K2	0.22	3.43E-05	3.55E-03
cg19854915	3	27770995		0.24	3.45E-05	3.56E-03
cg06088918	3	27755600		0.27	3.45E-05	3.56E-03
cg02441618	17	40936570	WNK4	0.21	3.45E-05	3.56E-03
cg26494929	16	3017495	KREMEN2	0.21	3.48E-05	3.58E-03
cg01214340	13	50070499	PHF11	0.21	3.49E-05	3.59E-03
cg25707676	7	96643539	DLX6AS	0.21	3.49E-05	3.59E-03
cg16027343	5	180632948	TRIM7	0.24	3.49E-05	3.59E-03
cg15367453	2	63287288		0.27	3.50E-05	3.60E-03
cg25819275	1	248684766	OR2G6	-0.23	3.51E-05	3.60E-03
cg00350835	11	16838596	PLEKHA7	-0.22	3.51E-05	3.60E-03
cg05854694	14	61123243		0.25	3.52E-05	3.60E-03
cg10227331	1	8181647		-0.20	3.52E-05	3.61E-03
cg13683361	9	103793011	LPPR1	0.21	3.53E-05	3.61E-03
cg00346208	1	20669905	VWA5B1	0.23	3.54E-05	3.62E-03
cg08160331	11	75140865	KLHL35	0.20	3.55E-05	3.63E-03
cg12440105	2	239756128	TWIST2	0.21	3.55E-05	3.63E-03
cg15638414	19	31847893		0.24	3.56E-05	3.63E-03
cg15214183	11	67232269	TMEM134	0.24	3.57E-05	3.64E-03
cg23207676	19	41073603	SPTBN4	0.25	3.57E-05	3.64E-03
cg02838178	1	201368613	LAD1	0.23	3.58E-05	3.64E-03

cg00169792	20	50418554	SALL4	0.24	3.58E-05	3.64E-03
cg25386676	5	172175721		0.25	3.58E-05	3.64E-03
cg24894584	12	1772230		0.20	3.59E-05	3.65E-03
cg10643691	8	95246514		0.25	3.59E-05	3.65E-03
cg20611276	6	133561866	EYA4	0.20	3.59E-05	3.65E-03
cg05787790	16	86550238		0.21	3.60E-05	3.65E-03
cg02861380	1	75596758	LHX8	0.25	3.62E-05	3.67E-03
cg09802835	11	71952131	PHOX2A	0.27	3.64E-05	3.68E-03
cg12215739	2	177014966	HOXD4	0.25	3.65E-05	3.69E-03
cg07250758	11	61049251	VWCE	0.23	3.66E-05	3.70E-03
cg26930448	1	218337485		0.23	3.67E-05	3.71E-03
cg08491487	6	30039130	RNF39	0.27	3.67E-05	3.71E-03
cg00566759	18	28682345	DSC2	0.23	3.68E-05	3.71E-03
cg18857062	6	43276478	CRIP3	0.22	3.68E-05	3.71E-03
cg17101450	10	102900365		0.30	3.69E-05	3.71E-03
cg16644023	7	27154845	HOXA3	0.21	3.70E-05	3.71E-03
cg02512920	10	23460457		0.24	3.70E-05	3.71E-03
cg13596049	18	30051538	FAM59A	0.21	3.71E-05	3.72E-03
cg04406454	6	10398494	TFAP2A	0.20	3.71E-05	3.72E-03
cg20343048	21	36041527	CLIC6	0.23	3.72E-05	3.73E-03
cg11028201	4	41867562		0.29	3.72E-05	3.73E-03
cg20072171	3	62356962	FEZF2	0.29	3.73E-05	3.74E-03
cg00292701	4	10463590		0.22	3.74E-05	3.74E-03
cg20099830	12	114842031	TBX5	0.23	3.74E-05	3.74E-03
cg19685491	12	2800278	CACNA1C	0.22	3.76E-05	3.75E-03
cg03477112	10	125034571		0.21	3.76E-05	3.75E-03
cg05068866	3	66633374		-0.21	3.77E-05	3.75E-03
cg04674755	7	44643793		-0.20	3.78E-05	3.77E-03
cg09231171	16	49518414		0.25	3.79E-05	3.77E-03
cg08648317	11	59807830	PLAC1L	-0.24	3.80E-05	3.78E-03
cg20756891	5	170743951		0.26	3.81E-05	3.78E-03
cg19416417	2	223290324	SGPP2	0.23	3.82E-05	3.79E-03
cg21201099	17	59482446	TBX2	0.21	3.82E-05	3.79E-03
cg10434261	1	156358059		0.22	3.83E-05	3.80E-03
cg06355129	1	156646293	NES	0.21	3.84E-05	3.80E-03
cg12062220	15	53087232		0.25	3.85E-05	3.81E-03
cg07416237	13	100548078	CLYBL	0.24	3.85E-05	3.81E-03
cg09591329	2	102388664	MAP4K4	-0.22	3.86E-05	3.82E-03
cg11079129	5	172669916		0.23	3.87E-05	3.82E-03
cg14161821	9	6955559	KDM4C	-0.22	3.89E-05	3.83E-03
cg03953660	2	45179787		0.27	3.89E-05	3.83E-03
cg18104354	10	106390703		-0.22	3.89E-05	3.83E-03
cg03052128	12	85671811		0.24	3.90E-05	3.84E-03
cg21843594	22	30476285	HORMAD2	0.25	3.90E-05	3.84E-03
cg16108059	14	81421989	TSHR	0.24	3.93E-05	3.86E-03
cg20658918	2	131185698		0.22	3.95E-05	3.87E-03
cg13230606	17	36105337	HNF1B	0.22	3.98E-05	3.89E-03
cg09701702	6	100915002		0.25	3.98E-05	3.89E-03
cg03217995	7	27203430	HOXA9	-0.27	3.99E-05	3.90E-03
cg04028695	10	80008319		-0.21	3.99E-05	3.90E-03
cg10138970	2	223172052		0.24	4.00E-05	3.90E-03
cg08717880	2	177014849	HOXD4	0.22	4.00E-05	3.90E-03
cg08973839	11	49862012		-0.21	4.00E-05	3.90E-03
cg03805618	2	175198664	SP9	0.26	4.02E-05	3.91E-03
cg21046967	17	37762455	NEUROD2	0.26	4.08E-05	3.96E-03
cg26987660	11	71952431	PHOX2A	0.24	4.08E-05	3.96E-03
cg10043037	7	19185407	FERD3L	0.27	4.09E-05	3.96E-03
cg01244342	6	32709470	HLA-DQA2	-0.24	4.11E-05	3.97E-03
cg15209808	22	30476254	HORMAD2	0.27	4.12E-05	3.98E-03
cg21188820	3	27765546		0.28	4.12E-05	3.98E-03
cg20065569	14	73958280	HEATR4	0.26	4.14E-05	4.00E-03
cg23639692	7	27175152		0.20	4.14E-05	4.00E-03
cg01926238	6	117584715		0.25	4.14E-05	4.00E-03
cg10188897	17	42392436	RUNDC3A	0.24	4.14E-05	4.00E-03
cg17594860	1	19615358	AKR7A3	0.23	4.15E-05	4.00E-03
cg00421848	21	35832164	KCNE1	0.22	4.16E-05	4.00E-03

cg16582419	6	30038998	RNF39	0.23	4.16E-05	4.00E-03
cg12689806	1	248042285	TRIM58	-0.24	4.16E-05	4.00E-03
cg22345576	19	36000517	DMKN	0.22	4.16E-05	4.00E-03
cg08638184	7	19183563		0.25	4.17E-05	4.01E-03
cg03540794	5	2112109		-0.25	4.17E-05	4.01E-03
cg19925204	10	119307692	EMX2	0.26	4.17E-05	4.01E-03
cg11861970	5	67484008		-0.24	4.18E-05	4.01E-03
cg02736908	17	29887376		0.21	4.19E-05	4.02E-03
cg08862373	1	17478571		-0.21	4.19E-05	4.02E-03
cg10348193	7	150020240	LRRC61	0.21	4.20E-05	4.03E-03
cg15359163	5	122429178	PRDM6	0.26	4.20E-05	4.03E-03
cg22275864	8	37822562	ADRB3	0.22	4.21E-05	4.03E-03
cg10106804	1	35258594	GJA4	0.25	4.21E-05	4.03E-03
cg19666287	13	45945197	LOC100190939	0.21	4.22E-05	4.03E-03
cg01250603	10	122709029		0.24	4.23E-05	4.04E-03
cg22686881	5	170737255	TLX3	0.22	4.23E-05	4.04E-03
cg15087376	3	149113539		-0.21	4.24E-05	4.04E-03
cg05492170	4	111536341		0.21	4.25E-05	4.04E-03
cg17803993	3	71276214	FOXP1	-0.20	4.26E-05	4.05E-03
cg00928397	2	175198952	SP9	0.21	4.26E-05	4.05E-03
cg06195987	7	98100169		0.25	4.27E-05	4.06E-03
cg02530022	7	96642708	DLX6AS	0.25	4.28E-05	4.06E-03
cg22272282	6	27107670	HIST1H2BK	0.30	4.28E-05	4.06E-03
cg05845757	1	36039885	TFAP2E	0.23	4.28E-05	4.06E-03
cg18135771	3	13323959		0.25	4.28E-05	4.06E-03
cg01817364	5	43037411		0.25	4.28E-05	4.06E-03
cg27151303	7	27184821		0.22	4.29E-05	4.06E-03
cg13019143	7	96633060	DLX6AS	0.23	4.29E-05	4.06E-03
cg16640855	2	182543233	NEUROD1	0.29	4.31E-05	4.07E-03
cg13354988	2	263656	ACP1	0.27	4.31E-05	4.07E-03
cg09682128	13	100613240		0.25	4.32E-05	4.08E-03
cg22606869	12	49658703	TUBA1C	0.25	4.33E-05	4.08E-03
cg07598580	1	86620525	COL24A1	0.21	4.33E-05	4.08E-03
cg21322654	7	107301048	SLC26A4	0.22	4.34E-05	4.09E-03
cg21495139	14	105070749	TMEM179	0.21	4.35E-05	4.09E-03
cg13768290	2	63285491		0.21	4.35E-05	4.09E-03
cg09486778	2	223169609	CCDC140	0.25	4.36E-05	4.10E-03
cg13637269	19	22697330		-0.22	4.36E-05	4.10E-03
cg20643952	12	54398809		0.22	4.36E-05	4.10E-03
cg15541062	11	5531199	UBQLN3	-0.24	4.37E-05	4.10E-03
cg24725789	10	119310288		0.25	4.37E-05	4.10E-03
cg17592360	1	119543590		0.28	4.37E-05	4.10E-03
cg10293403	6	10421670		0.27	4.38E-05	4.11E-03
cg18698884	11	16632724		0.21	4.40E-05	4.12E-03
cg09854003	5	170743459		0.24	4.41E-05	4.13E-03
cg01994275	7	8480586	NXPH1	0.20	4.44E-05	4.14E-03
cg11053663	11	58034457	OR10W1	-0.21	4.45E-05	4.15E-03
cg10394788	9	124360547	DAB2IP	0.23	4.45E-05	4.15E-03
cg08453263	11	16838675	PLEKHA7	-0.22	4.46E-05	4.16E-03
cg22370252	12	54446289	HOXC4	0.23	4.47E-05	4.16E-03
cg02444978	7	16438128	ISPD	-0.21	4.49E-05	4.17E-03
cg06637517	3	52407344	DNAH1	0.25	4.49E-05	4.17E-03
cg11168433	1	41283979	KCNQ4	0.23	4.50E-05	4.18E-03
cg11930400	4	111533883		0.25	4.50E-05	4.18E-03
cg26033466	7	96644782	DLX6AS	0.22	4.50E-05	4.18E-03
cg22381351	3	100424830		-0.21	4.51E-05	4.18E-03
cg02599464	6	27107097	HIST1H2BK	0.29	4.51E-05	4.18E-03
cg00400448	15	60285691		0.25	4.51E-05	4.18E-03
cg23874561	4	81124484	PRDM8	0.23	4.51E-05	4.18E-03
cg08315770	6	39281885	KCNK17	0.28	4.52E-05	4.18E-03
cg09774842	20	3052483	OXT	0.22	4.52E-05	4.18E-03
cg07390647	3	98453086	ST3GAL6	0.22	4.52E-05	4.18E-03
cg05569742	5	122429414	PRDM6	0.24	4.52E-05	4.19E-03
cg05279513	11	9880619	SBF2	-0.20	4.53E-05	4.19E-03
cg13870102	1	33772519		0.23	4.54E-05	4.20E-03
cg20216139	11	16632509		0.20	4.55E-05	4.20E-03

cg11330740	5	172660884	NKX2-5	0.21	4.55E-05	4.21E-03
cg01089249	2	171676553	GAD1	0.22	4.56E-05	4.21E-03
cg15666214	6	108438316		0.25	4.57E-05	4.22E-03
cg08205700	8	80696168		0.25	4.58E-05	4.22E-03
cg21124375	2	232765392		0.23	4.59E-05	4.23E-03
cg19108747	2	225266263	FAM124B	-0.24	4.60E-05	4.23E-03
cg09093485	6	30038882	RNF39	0.21	4.61E-05	4.23E-03
cg25138715	3	157217414	VEPH1	0.25	4.63E-05	4.25E-03
cg22284043	13	92051576	GPC5	0.25	4.63E-05	4.25E-03
cg03157027	8	99959723	OSR2	0.20	4.64E-05	4.25E-03
cg14106308	3	157217286	VEPH1	0.25	4.65E-05	4.26E-03
cg02081701	2	139537197	NXPH2	0.26	4.67E-05	4.27E-03
cg20402783	12	54381013	HOXC10	0.26	4.69E-05	4.29E-03
cg27400447	13	100549017	CLYBL	0.28	4.71E-05	4.30E-03
cg24789723	10	102501346		0.23	4.73E-05	4.32E-03
cg23651585	7	70252448	AUTS2	0.22	4.74E-05	4.32E-03
cg11447335	5	865350	BRD9	-0.23	4.75E-05	4.33E-03
cg17733616	17	73083887	SLC16A5	0.25	4.76E-05	4.33E-03
cg20647118	13	92051786	GPC5	0.21	4.77E-05	4.33E-03
cg16013925	6	30483908		0.21	4.80E-05	4.35E-03
cg22531904	7	99595276		0.25	4.81E-05	4.36E-03
cg14911395	3	50311213	SEMA3B	0.21	4.81E-05	4.36E-03
cg21848524	3	27771053		0.23	4.82E-05	4.36E-03
cg18980554	15	70233856		0.26	4.84E-05	4.37E-03
cg18958126	13	100547367	CLYBL	0.29	4.84E-05	4.37E-03
cg24340935	19	3877286		0.20	4.85E-05	4.38E-03
cg27369096	5	2169769		-0.26	4.86E-05	4.38E-03
cg20264732	16	68269763	ESRP2	0.22	4.87E-05	4.39E-03
cg19871388	20	50419082	SALL4	0.23	4.90E-05	4.41E-03
cg25640822	5	134871645	NEUROG1	0.21	4.90E-05	4.41E-03
cg20438306	4	41747092	PHOX2B	0.23	4.93E-05	4.43E-03
cg15422685	3	62356774	FEZF2	0.22	4.93E-05	4.43E-03
cg09058099	12	10853263	CSDA	-0.21	4.94E-05	4.43E-03
cg07561547	1	201368783	LAD1	0.24	4.95E-05	4.43E-03
cg13020870	15	37173017	LOC145845	0.25	4.95E-05	4.43E-03
cg20139247	8	65286989		0.25	4.95E-05	4.44E-03
cg09781994	4	111548118	PITX2	0.22	4.96E-05	4.44E-03
cg07159802	1	247921568	OR1C1	-0.23	4.96E-05	4.44E-03
cg02155658	20	21690036	PAX1	0.22	4.99E-05	4.46E-03
cg16706324	11	5685914	TRIM78P	-0.24	4.99E-05	4.46E-03
cg02167020	10	102899949		0.25	4.99E-05	4.46E-03
cg12535715	8	38831693	HTRA4	0.21	5.00E-05	4.46E-03
cg06756211	1	223936799	CAPN2	0.26	5.00E-05	4.46E-03
cg07056644	16	86546785	FOXF1	0.25	5.01E-05	4.47E-03
cg19814934	5	42950152		0.27	5.02E-05	4.47E-03
cg03344149	13	28535559		0.21	5.03E-05	4.48E-03
cg26066361	12	10282621	CLEC7A	-0.25	5.03E-05	4.48E-03
cg03744763	7	27184737	HOXA5	0.23	5.03E-05	4.48E-03
cg16919569	4	174452835	NBLA00301	0.23	5.04E-05	4.48E-03
cg04787024	2	99553442	C2orf55	0.28	5.04E-05	4.48E-03
cg00673286	15	83349613	AP3B2	0.21	5.06E-05	4.49E-03
cg10996039	7	27178861		0.21	5.06E-05	4.49E-03
cg08741898	10	102881276	TLX1NB	0.27	5.07E-05	4.49E-03
cg00894757	3	170137240	CLDN11	0.24	5.07E-05	4.49E-03
cg03977089	8	65287005		0.23	5.08E-05	4.50E-03
cg17236169	11	64480683	NRXN2	0.22	5.09E-05	4.51E-03
cg24740026	5	10333868		0.29	5.12E-05	4.52E-03
cg23619769	11	71952356	PHOX2A	0.25	5.12E-05	4.52E-03
cg22669060	21	34774882	IFNGR2	0.20	5.14E-05	4.53E-03
cg11982736	6	43276570	CRIP3	0.23	5.14E-05	4.54E-03
cg07035659	4	4859975	MSX1	0.21	5.16E-05	4.54E-03
cg01076272	16	86320281		0.20	5.17E-05	4.55E-03
cg17176016	6	108441207		0.21	5.18E-05	4.56E-03
cg03171003	2	111875934		0.21	5.19E-05	4.56E-03
cg14013695	7	27184176	HOXA5	0.23	5.19E-05	4.56E-03
cg14679255	12	54385526	MIR196A2	0.20	5.19E-05	4.57E-03

cg23358625	19	23945641	RPSAP58	0.29	5.20E-05	4.57E-03
cg06829299	8	65286632		0.22	5.21E-05	4.57E-03
cg03895540	11	5841482	OR52N2	-0.21	5.21E-05	4.57E-03
cg26193694	7	6543354	GRID2IP	0.27	5.23E-05	4.58E-03
cg09992746	15	60285821		0.21	5.26E-05	4.60E-03
cg18352935	11	4843021	OR51F2	-0.21	5.33E-05	4.65E-03
cg02018902	15	79576149	ANKRD34C	0.23	5.34E-05	4.66E-03
cg01177854	16	68270387	ESRP2	0.20	5.36E-05	4.66E-03
cg00647046	10	134734612		0.20	5.39E-05	4.68E-03
cg12974637	18	52626668	CCDC68	0.21	5.39E-05	4.68E-03
cg14466251	9	134704033		-0.22	5.41E-05	4.69E-03
cg26837870	12	49658878	TUBA1C	0.21	5.41E-05	4.69E-03
cg14080155	2	45179727		0.23	5.41E-05	4.69E-03
cg13519918	12	89748985		0.22	5.45E-05	4.72E-03
cg27553955	2	42720326	KCNG3	0.22	5.45E-05	4.72E-03
cg20374595	13	100627956		0.29	5.45E-05	4.72E-03
cg16840358	3	27765484		0.28	5.45E-05	4.72E-03
cg21493633	4	166795596	TLL1	0.22	5.47E-05	4.72E-03
cg14819504	2	160761413	LY75	0.25	5.47E-05	4.72E-03
cg06303772	19	17717530	UNC13A	0.26	5.48E-05	4.73E-03
cg21306329	17	73083876	SLC16A5	0.22	5.48E-05	4.73E-03
cg16406892	12	115118777	TBX3	0.22	5.49E-05	4.74E-03
cg07150255	6	29324414	OR5V1	-0.24	5.51E-05	4.74E-03
cg15823495	12	123214554	GPR81	-0.23	5.51E-05	4.74E-03
cg17082225	7	56673049		-0.20	5.51E-05	4.74E-03
cg12687069	11	5474001	OR51I2	-0.21	5.52E-05	4.75E-03
cg03780851	8	102504564	GRHL2	0.20	5.52E-05	4.75E-03
cg20857455	12	39300142	CPNE8	0.27	5.53E-05	4.75E-03
cg03051777	12	115131789		0.24	5.54E-05	4.76E-03
cg05741937	12	104609797	TXNRD1	0.23	5.55E-05	4.76E-03
cg13030332	12	115117351	TBX3	0.23	5.56E-05	4.76E-03
cg24481868	13	50070504	PHF11	0.23	5.56E-05	4.76E-03
cg06856720	20	19870217	RIN2	-0.22	5.57E-05	4.76E-03
cg25318296	13	100547785	CLYBL	0.24	5.57E-05	4.76E-03
cg02245378	2	223161771	PAX3	0.23	5.58E-05	4.77E-03
cg26550710	8	97167878	GDF6	0.22	5.58E-05	4.77E-03
cg12784175	10	103589988	KCNIP2	0.23	5.59E-05	4.78E-03
cg12883629	6	25042497		0.22	5.59E-05	4.78E-03
cg15383276	22	29709282	RASL10A	0.26	5.61E-05	4.79E-03
cg20169823	12	116354837		0.23	5.61E-05	4.79E-03
cg24570303	10	102900491		0.26	5.63E-05	4.80E-03
cg11539674	7	127291444	SND1	0.22	5.65E-05	4.81E-03
cg18358020	5	170744407		0.22	5.65E-05	4.81E-03
cg25093813	2	45030336		0.23	5.67E-05	4.81E-03
cg02721000	12	54441213	HOXC4	0.23	5.68E-05	4.82E-03
cg07790170	4	111537570		0.28	5.69E-05	4.82E-03
cg14173147	9	27528300	MOBKL2B	0.24	5.70E-05	4.83E-03
cg11176990	2	39187533	LOC375196	0.20	5.70E-05	4.83E-03
cg02987928	1	16553456		0.24	5.72E-05	4.84E-03
cg22029297	1	26736798	LIN28	0.22	5.72E-05	4.84E-03
cg22461835	8	26723365	ADRA1A	0.27	5.72E-05	4.84E-03
cg21618333	15	62359139	C2CD4A	0.23	5.75E-05	4.86E-03
cg02472998	13	45945362	LOC100190939	0.22	5.79E-05	4.89E-03
cg27579609	1	20003062	HTR6	0.23	5.82E-05	4.90E-03
cg23533419	12	54090519		0.21	5.82E-05	4.90E-03
cg12499235	11	2293173	ASCL2	0.23	5.82E-05	4.90E-03
cg22900415	13	20736075	GJA3	0.27	5.86E-05	4.92E-03
cg14486338	8	99440279	KCNS2	0.28	5.87E-05	4.93E-03
cg24812837	10	102894120	TLX1	0.28	5.88E-05	4.93E-03
cg02407720	6	10421372		0.28	5.88E-05	4.93E-03
cg17645823	12	114846321	TBX5	0.22	5.89E-05	4.94E-03
cg06379721	10	102590401		0.24	5.90E-05	4.94E-03
cg24840099	4	4864430	MSX1	0.23	5.92E-05	4.95E-03
cg11697427	10	102590168		0.25	5.93E-05	4.96E-03
cg23913421	6	50674663		0.26	5.95E-05	4.97E-03
cg07870307	5	140743780	PCDHGA4	0.22	5.95E-05	4.97E-03

cg01891966	7	27202344	HOXA9	-0.26	5.97E-05	4.98E-03
cg24913868	10	101296069	NKX2-3	0.24	5.97E-05	4.98E-03
cg18705773	5	170743564		0.25	5.99E-05	5.00E-03
cg23652859	19	59092692	MGC2752	0.20	6.00E-05	5.00E-03
cg23370306	2	242823485		0.21	6.00E-05	5.00E-03
cg12115991	6	108438047		0.22	6.01E-05	5.00E-03
cg18030960	4	13530948		0.25	6.02E-05	5.00E-03
cg27439292	17	7670719	DNAH2	0.20	6.02E-05	5.01E-03
cg25049698	6	50692605	TFAP2D	0.24	6.02E-05	5.01E-03
cg11412853	12	115129801		0.24	6.03E-05	5.01E-03
cg16265599	17	40936658	WNK4	0.23	6.06E-05	5.03E-03
cg00960305	20	22558297	C20orf56	0.24	6.07E-05	5.03E-03
cg07382435	1	50880864		0.20	6.13E-05	5.07E-03
cg17474422	1	36039866	TFAP2E	0.22	6.13E-05	5.08E-03
cg16701059	15	101513798	LRRK1	0.21	6.18E-05	5.11E-03
cg21383487	10	22624094		0.24	6.20E-05	5.12E-03
cg16391973	5	158689566	UBLCP1	-0.21	6.21E-05	5.13E-03
cg16290866	8	97167768	GDF6	0.25	6.22E-05	5.13E-03
cg07063351	19	41060190	SPTBN4	0.24	6.22E-05	5.13E-03
cg27123256	14	99664279	BCL11B	-0.21	6.23E-05	5.14E-03
cg04555220	5	9546695	SEMA5A	0.23	6.26E-05	5.15E-03
cg22660542	12	54402431	HOXC8	0.20	6.27E-05	5.15E-03
cg01331772	2	131094827		0.28	6.32E-05	5.18E-03
cg00059699	12	10455788	KLRD1	-0.25	6.33E-05	5.19E-03
cg02504521	12	46660819	SLC38A1	0.27	6.33E-05	5.19E-03
cg26382551	10	11047284	CUGBP2	0.23	6.34E-05	5.19E-03
cg13457165	1	40105079	HEYL	0.21	6.34E-05	5.19E-03
cg06444452	4	619635	PDE6B	-0.20	6.36E-05	5.20E-03
cg23161999	2	162283189		0.24	6.37E-05	5.20E-03
cg26305817	5	172656055		0.25	6.38E-05	5.21E-03
cg09230996	10	102498419		0.21	6.39E-05	5.21E-03
cg01636650	3	38040315	VILL	0.25	6.39E-05	5.21E-03
cg20403938	12	54377457	HOXC10	0.22	6.42E-05	5.23E-03
cg17180386	15	51386452	TNFAIP8L3	0.22	6.46E-05	5.25E-03
cg24700222	1	86622682	COL24A1	0.25	6.46E-05	5.25E-03
cg00394180	8	97167170	GDF6	0.27	6.48E-05	5.25E-03
cg23893898	4	6247571		0.20	6.48E-05	5.26E-03
cg11673291	1	36787145		0.22	6.49E-05	5.26E-03
cg11891395	7	96652153	DLX5	0.27	6.50E-05	5.26E-03
cg22158769	2	39187539	LOC375196	0.22	6.51E-05	5.27E-03
cg16670554	15	68118985	LBXCOR1	0.24	6.52E-05	5.27E-03
cg05959932	6	2382734		-0.22	6.52E-05	5.27E-03
cg17968795	2	99553406	C2orf55	0.27	6.53E-05	5.27E-03
cg06572465	5	140800929	PCDHGA4	0.22	6.54E-05	5.28E-03
cg03778029	18	52626773	CCDC68	0.21	6.54E-05	5.28E-03
cg04273871	10	102894227	TLX1	0.25	6.54E-05	5.28E-03
cg12552771	22	29709320	RASL10A	0.24	6.57E-05	5.30E-03
cg14820199	4	11429415	HS3ST1	0.22	6.57E-05	5.30E-03
cg11947493	8	23021515	TNFRSF10D	0.21	6.58E-05	5.30E-03
cg26104752	1	119527377	TBX15	0.22	6.59E-05	5.31E-03
cg14218861	10	119443973		-0.21	6.60E-05	5.31E-03
cg25637829	10	77155545		0.24	6.60E-05	5.31E-03
cg19780352	4	169798931	PALLD	0.22	6.61E-05	5.31E-03
cg01901107	7	150748284	ACCN3	0.24	6.62E-05	5.32E-03
cg20008148	14	37053169	NKX2-8	0.21	6.63E-05	5.32E-03
cg24029517	4	11429531	HS3ST1	0.23	6.65E-05	5.33E-03
cg24875593	21	45153009	PDXK	-0.22	6.66E-05	5.34E-03
cg06303238	20	50418959	SALL4	0.23	6.67E-05	5.34E-03
cg13510849	6	164572718		-0.24	6.67E-05	5.34E-03
cg24735307	10	102881114	TLX1NB	0.24	6.67E-05	5.34E-03
cg04924185	16	56710098	MT1IP	0.21	6.70E-05	5.36E-03
cg09782540	6	10398698	TFAP2A	0.23	6.71E-05	5.37E-03
cg01974001	16	1297276		-0.22	6.74E-05	5.38E-03
cg09349604	4	41880747		0.25	6.74E-05	5.39E-03
cg07977620	11	56380614	OR5M1	-0.21	6.76E-05	5.39E-03
cg17386710	7	70252335	AUTS2	0.26	6.79E-05	5.41E-03

cg25995955	4	151500631	LRBA	0.23	6.81E-05	5.42E-03
cg04436554	5	172669976		0.21	6.83E-05	5.43E-03
cg08064488	21	38068043		0.21	6.83E-05	5.43E-03
cg09951047	9	97848306	C9orf3	-0.23	6.84E-05	5.43E-03
cg02683759	2	157177072		0.24	6.84E-05	5.43E-03
cg21737226	8	53326668		-0.20	6.85E-05	5.44E-03
cg21858614	17	8907340		0.23	6.87E-05	5.44E-03
cg20206984	6	1381511		0.20	6.87E-05	5.44E-03
cg12973118	2	223168941	CCDC140	0.22	6.88E-05	5.45E-03
cg24811352	5	140529499	PCDHB6	0.21	6.91E-05	5.46E-03
cg18209835	7	6543114	GRID2IP	0.26	6.91E-05	5.46E-03
cg12813724	1	26690537	ZNF683	0.23	6.99E-05	5.50E-03
cg01502588	2	119615889		0.26	7.00E-05	5.51E-03
cg24941387	19	48491941	BSPH1	-0.23	7.02E-05	5.51E-03
cg09886946	13	33590048	KL	0.22	7.04E-05	5.53E-03
cg09290705	15	60285508		0.23	7.05E-05	5.53E-03
cg18161327	13	100627703		0.28	7.05E-05	5.53E-03
cg05721365	17	62777693	LOC146880	0.21	7.07E-05	5.54E-03
cg08871964	19	41025863	SPTBN4	0.20	7.10E-05	5.56E-03
cg16600991	12	54389477		0.27	7.10E-05	5.56E-03
cg14515571	11	20178627	DBX1	0.23	7.11E-05	5.56E-03
cg18557185	7	27128169		0.24	7.12E-05	5.56E-03
cg14395444	12	2800182	CACNA1C	0.23	7.12E-05	5.57E-03
cg24464265	4	809452	CPLX1	-0.21	7.16E-05	5.58E-03
cg17223809	1	86620676	COL24A1	0.22	7.17E-05	5.58E-03
cg15777781	10	11047271	CUGBP2	0.21	7.20E-05	5.60E-03
cg24330456	6	30038955	RNF39	0.26	7.23E-05	5.63E-03
cg19598416	1	223936812	CAPN2	0.26	7.27E-05	5.65E-03
cg24053070	21	17962124	C21orf34	-0.25	7.29E-05	5.66E-03
cg19187831	11	67232316	TMEM134	0.21	7.30E-05	5.66E-03
cg10186366	17	71898817		0.22	7.31E-05	5.67E-03
cg03312366	2	45179416		0.23	7.34E-05	5.68E-03
cg26818625	8	72471153		0.24	7.36E-05	5.70E-03
cg14684854	2	241901865		-0.21	7.37E-05	5.70E-03
cg03001942	2	71115787		0.25	7.37E-05	5.70E-03
cg03814390	17	77776716		0.24	7.37E-05	5.70E-03
cg14311811	18	29077863	DSG2	0.22	7.38E-05	5.70E-03
cg20018806	11	59633874	TCN1	-0.22	7.38E-05	5.70E-03
cg08935125	16	3068085	CLDN6	0.22	7.39E-05	5.70E-03
cg17555373	13	53775108		0.25	7.40E-05	5.71E-03
cg07400503	10	134110222	STK32C	-0.21	7.40E-05	5.71E-03
cg08184792	12	48592270		0.21	7.41E-05	5.71E-03
cg20818806	13	110885926	COL4A1	-0.24	7.41E-05	5.71E-03
cg12214451	4	114900175	ARSJ	0.23	7.41E-05	5.71E-03
cg24845578	5	95170808		0.23	7.45E-05	5.73E-03
cg20454887	2	54935920		0.22	7.46E-05	5.73E-03
cg16680438	1	16553549		0.21	7.58E-05	5.79E-03
cg22482278	17	80289631	SECTM1	-0.22	7.58E-05	5.79E-03
cg06272038	11	2292512	ASCL2	0.23	7.59E-05	5.80E-03
cg03320827	6	10887367	SYCP2L	0.24	7.60E-05	5.80E-03
cg03269050	10	102881545	TLX1NB	0.23	7.61E-05	5.81E-03
cg13759674	9	140051205	GRIN1	0.28	7.63E-05	5.82E-03
cg04181892	2	54936027		0.22	7.70E-05	5.85E-03
cg03201044	20	55203291	TFAP2C	0.25	7.71E-05	5.86E-03
cg08658621	10	133999287	DPYSL4	0.21	7.73E-05	5.87E-03
cg16168014	7	42928245		0.25	7.76E-05	5.88E-03
cg12458970	20	44656372	SLC12A5	0.21	7.78E-05	5.90E-03
cg22524061	8	99959430	OSR2	0.24	7.79E-05	5.90E-03
cg13345318	9	127216008	GPR144	0.25	7.80E-05	5.90E-03
cg03126579	19	3822189	ZFR2	0.26	7.80E-05	5.90E-03
cg00736459	6	100904376	SIM1	0.22	7.80E-05	5.90E-03
cg13330341	11	2292625	ASCL2	0.21	7.81E-05	5.91E-03
cg16458436	12	114846412	TBX5	0.23	7.82E-05	5.91E-03
cg22968167	11	4945873	OR51G1	-0.25	7.82E-05	5.91E-03
cg27649239	15	68120393	LBXCOR1	0.27	7.83E-05	5.91E-03
cg10281002	12	114846399	TBX5	0.27	7.85E-05	5.92E-03

cg19713196	1	201368390	LAD1	0.23	7.86E-05	5.93E-03
cg11193411	13	30098324	SLC7A1	-0.21	7.89E-05	5.94E-03
cg03737788	10	79492045		-0.21	7.94E-05	5.97E-03
cg26394220	2	219867925	MIR375	0.26	7.95E-05	5.97E-03
cg00034468	1	229568077	ACTA1	0.20	7.98E-05	5.99E-03
cg20129210	13	100547192	CLYBL	0.29	8.00E-05	6.00E-03
cg07462756	4	4859985	MSX1	0.22	8.01E-05	6.00E-03
cg12276019	8	11057947	XKR6	0.25	8.02E-05	6.01E-03
cg18918321	8	41424524		0.21	8.03E-05	6.01E-03
cg00654159	16	32858241		-0.21	8.06E-05	6.02E-03
cg15855970	6	99273338		0.23	8.07E-05	6.03E-03
cg27291698	6	43276578	CRIP3	0.23	8.07E-05	6.03E-03
cg14900124	2	177017630	HOXD4	0.21	8.07E-05	6.03E-03
cg00947032	13	36919960	SPG20	0.23	8.11E-05	6.05E-03
cg14201467	11	115372348	CADM1	0.21	8.12E-05	6.05E-03
cg13918518	17	59488240	C17orf82	0.24	8.14E-05	6.06E-03
cg21960859	4	166795649	TLL1	0.23	8.17E-05	6.07E-03
cg05155840	4	174448841	HAND2	0.22	8.18E-05	6.07E-03
cg16360659	6	28484320	GPX6	-0.24	8.22E-05	6.09E-03
cg18241829	2	176952696		0.20	8.22E-05	6.09E-03
cg15199678	7	96642510	DLX6AS	0.23	8.23E-05	6.10E-03
cg03160883	6	36628713		0.23	8.24E-05	6.10E-03
cg26465602	16	1098847		0.23	8.26E-05	6.11E-03
cg02053964	12	124950784	NCOR2	0.21	8.28E-05	6.12E-03
cg17463745	1	16554402		0.25	8.29E-05	6.12E-03
cg19470379	15	74425092	ISLR2	0.21	8.31E-05	6.13E-03
cg09515953	19	38747355	PPP1R14A	0.22	8.32E-05	6.14E-03
cg17453840	15	83317526	CPEB1	0.21	8.36E-05	6.15E-03
cg15724945	1	119548527		0.22	8.36E-05	6.15E-03
cg14554846	6	99273218		0.21	8.37E-05	6.15E-03
cg19656950	13	100607772		0.22	8.37E-05	6.16E-03
cg17820365	8	97157856	GDF6	0.22	8.38E-05	6.16E-03
cg24263062	20	2730191	EBF4	0.23	8.38E-05	6.16E-03
cg14657834	8	67875441		0.21	8.40E-05	6.17E-03
cg18473733	19	16437362	KLF2	0.21	8.40E-05	6.17E-03
cg19904058	10	135279010	LOC619207	0.21	8.43E-05	6.18E-03
cg07638650	10	43697849	RASGEF1A	0.24	8.45E-05	6.19E-03
cg06921011	10	102589467	PAX2	0.23	8.45E-05	6.19E-03
cg18240400	10	46168597	ANUBL1	0.21	8.46E-05	6.19E-03
cg13191808	1	55505327	PCSK9	0.22	8.48E-05	6.20E-03
cg16407924	2	1452260	TPO	-0.21	8.50E-05	6.21E-03
cg16594007	19	57641963	USP29	-0.21	8.53E-05	6.22E-03
cg14038391	10	102900130		0.28	8.54E-05	6.23E-03
cg03762994	17	6899332	ALOX12	0.20	8.55E-05	6.23E-03
cg21376799	6	30094960		0.21	8.59E-05	6.25E-03
cg22190114	19	56459234	NLRP8	-0.20	8.62E-05	6.27E-03
cg08871608	19	40729274	CNTD2	0.23	8.63E-05	6.27E-03
cg09829319	6	10882238	GCM2	0.25	8.63E-05	6.27E-03
cg21790626	19	58220494	ZNF154	0.27	8.67E-05	6.29E-03
cg11655243	5	140778396	PCDHGA4	0.20	8.68E-05	6.29E-03
cg08049853	4	41875784		0.20	8.71E-05	6.31E-03
cg16501625	16	54628759		0.24	8.73E-05	6.31E-03
cg07783843	2	176997311		0.21	8.76E-05	6.33E-03
cg17259358	6	99273385		0.22	8.78E-05	6.34E-03
cg03399905	15	79576060	ANKRD34C	0.26	8.79E-05	6.34E-03
cg12392473	20	48099331	KCNB1	0.26	8.80E-05	6.35E-03
cg16561266	17	62777777	LOC146880	0.20	8.83E-05	6.36E-03
cg27140058	4	102712216	BANK1	0.23	8.86E-05	6.37E-03
cg01962676	1	18436969	IGSF21	0.24	8.87E-05	6.38E-03
cg11989330	1	65468827		0.25	8.88E-05	6.38E-03
cg09234616	11	32452592	WT1	0.26	8.89E-05	6.39E-03
cg08925169	11	5664261	TRIM34	-0.26	8.92E-05	6.40E-03
cg17139085	1	36787356		0.24	8.95E-05	6.43E-03
cg20536041	2	45029004		0.25	8.95E-05	6.43E-03
cg07625194	6	50683782	TFAP2D	0.25	8.96E-05	6.43E-03
cg04895288	7	98099949		0.24	8.98E-05	6.44E-03

cg13279673	6	50674522		0.23	8.98E-05	6.44E-03
cg02794451	12	2800446	CACNA1C	0.25	8.98E-05	6.44E-03
cg18978493	7	27225855	HOXA11AS	0.21	8.98E-05	6.44E-03
cg19127138	7	65196881		0.23	8.99E-05	6.44E-03
cg26190890	6	27107718	HIST1H2BK	0.25	9.01E-05	6.45E-03
cg22049858	11	94884121		0.22	9.01E-05	6.45E-03
cg24338833	8	10592227		0.21	9.02E-05	6.45E-03
cg01714811	3	27764473	EOMES	0.21	9.03E-05	6.45E-03
cg06531379	1	47911449		0.25	9.08E-05	6.48E-03
cg15244049	6	10883376	GCM2	0.25	9.12E-05	6.50E-03
cg14365570	14	73958272	HEATR4	0.24	9.12E-05	6.50E-03
cg15084543	1	79472408	ELTD1	0.21	9.13E-05	6.50E-03
cg17964844	12	49367911		0.22	9.13E-05	6.50E-03
cg04848682	14	77492060	C14orf4	0.21	9.14E-05	6.50E-03
cg10692467	3	50313021	SEMA3B	0.24	9.16E-05	6.51E-03
cg13968218	9	127216047	GPR144	0.25	9.18E-05	6.52E-03
cg05092308	2	63285365		0.22	9.19E-05	6.53E-03
cg15442907	12	2800463	CACNA1C	0.25	9.24E-05	6.55E-03
cg14250833	6	10882240	GCM2	0.27	9.26E-05	6.56E-03
cg12999453	17	59482319	TBX2	0.22	9.30E-05	6.58E-03
cg16136262	1	169429604		0.21	9.31E-05	6.58E-03
cg12206199	2	39187543	LOC375196	0.23	9.31E-05	6.58E-03
cg25139877	1	247691111	LOC148824	-0.24	9.31E-05	6.58E-03
cg24984523	13	50070436	PHF11	0.20	9.32E-05	6.58E-03
cg08169827	1	32054766		0.25	9.33E-05	6.58E-03
cg10374009	11	44341361		0.22	9.34E-05	6.59E-03
cg15929797	5	172660796	NKX2-5	0.28	9.36E-05	6.60E-03
cg20908789	8	11551964		0.20	9.40E-05	6.62E-03
cg24375409	1	38200920	EPHA10	0.24	9.40E-05	6.62E-03
cg23268208	22	30476345	HORMAD2	0.23	9.41E-05	6.62E-03
cg10375078	10	22624374		0.23	9.41E-05	6.62E-03
cg17320707	10	119307931	EMX2	0.22	9.41E-05	6.62E-03
cg11511443	12	16761406	LMO3	0.21	9.44E-05	6.63E-03
cg22022798	1	221065961		0.21	9.44E-05	6.63E-03
cg18362509	14	101925705		0.24	9.45E-05	6.63E-03
cg04085768	4	113333052	ALPK1	0.22	9.47E-05	6.65E-03
cg19280586	15	68125197	LBXCOR1	0.22	9.53E-05	6.67E-03
cg02909158	13	100616256	ZIC5	0.27	9.54E-05	6.68E-03
cg16568084	7	93811563		-0.22	9.58E-05	6.70E-03
cg03343571	6	30039175	RNF39	0.25	9.59E-05	6.70E-03
cg04568981	1	53125850		-0.20	9.60E-05	6.70E-03
cg05048976	3	55516243	WNT5A	0.24	9.65E-05	6.74E-03
cg18491080	15	60286463		0.20	9.68E-05	6.75E-03
cg10128164	14	31344535	COCH	0.23	9.68E-05	6.75E-03
cg04651603	12	54321302		0.20	9.69E-05	6.76E-03
cg24923509	10	102498399		0.22	9.71E-05	6.77E-03
cg02719154	4	111533823		0.24	9.71E-05	6.77E-03
cg05477457	4	169799308	PALLD	0.27	9.71E-05	6.77E-03
cg08867241	15	53087487		0.20	9.72E-05	6.77E-03
cg09359114	7	96650096	DLX5	0.23	9.75E-05	6.78E-03
cg27236629	15	96877720	NR2F2	0.22	9.76E-05	6.79E-03
cg24115264	13	43149043	TNFSF11	0.23	9.77E-05	6.79E-03
cg00260802	20	21372203		0.22	9.78E-05	6.80E-03
cg11966450	18	59992157	TNFRSF11A	0.21	9.78E-05	6.80E-03
cg03365496	15	60286384		0.22	9.78E-05	6.80E-03
cg01323381	7	27184264	HOXA5	0.23	9.79E-05	6.80E-03
cg10456990	21	38077597	SIM2	0.24	9.80E-05	6.80E-03
cg09656363	13	53775035		0.21	9.81E-05	6.81E-03
cg20747577	7	26897253	SKAP2	0.22	9.83E-05	6.81E-03
cg01604429	11	18208699		-0.23	9.87E-05	6.83E-03
cg03207151	12	54442288	HOXC4	0.22	9.93E-05	6.86E-03
cg00689580	6	10420798	TFAP2A	0.25	9.93E-05	6.86E-03
cg01131038	8	10592118		0.22	9.94E-05	6.87E-03
cg22083053	4	13541444		0.24	9.95E-05	6.87E-03
cg00875272	1	47695321	TAL1	0.22	9.96E-05	6.87E-03
cg02449166	10	131748481	EBF3	0.20	9.97E-05	6.88E-03

cg11940285	3	129693385	TRH	0.26	0.000100019	6.89E-03
cg27049766	19	58220516	ZNF154	0.27	0.00010024	6.91E-03
cg13717446	17	62777761	LOC146880	0.20	0.000100342	6.91E-03
cg19893751	2	45029285		0.27	0.000100645	6.92E-03
cg08988797	8	102505513	GRHL2	0.22	0.000100905	6.94E-03
cg16961816	7	96633530	DLX6AS	0.23	0.000101003	6.94E-03
cg04859726	6	100894513	SIM1	0.21	0.000101114	6.94E-03
cg00953355	17	70114129		0.21	0.000101459	6.96E-03
cg00049664	16	66613334	CMTM2	0.20	0.000101614	6.96E-03
cg23646776	4	111536970		0.24	0.00010163	6.96E-03
cg25916711	5	172660080	NKX2-5	0.25	0.000101666	6.96E-03
cg25149238	2	45030384		0.23	0.000101955	6.98E-03
cg02457328	12	103355190		0.23	0.000102083	6.98E-03
cg08981421	7	27199956		-0.23	0.000102333	6.99E-03
cg04223006	5	82845599	VCAN	-0.23	0.000102405	6.99E-03
cg00385863	1	41847974		-0.21	0.000102837	7.01E-03
cg01383890	15	62358982	C2CD4A	0.21	0.000103089	7.02E-03
cg21104276	7	90896572	FZD1	0.25	0.000103098	7.02E-03
cg00096104	13	100547897	CLYBL	0.23	0.000103266	7.03E-03
cg12451631	10	43698141	RASGEF1A	0.25	0.000103336	7.03E-03
cg16749785	8	12958083	DLC1	-0.21	0.000103414	7.04E-03
cg17412442	11	5652129	TRIM34	-0.23	0.000103555	7.04E-03
cg27122725	5	142781723	NR3C1	0.21	0.000103666	7.04E-03
cg02700891	3	129693586	TRH	0.25	0.00010367	7.04E-03
cg22099441	6	170531660		-0.20	0.000104085	7.06E-03
cg03546235	10	102590290		0.23	0.000104477	7.07E-03
cg12687157	5	68710912	MARVELD2	0.23	0.000104481	7.07E-03
cg16176379	16	55543049	LPCAT2	0.22	0.000104564	7.08E-03
cg11416290	4	111532410		0.23	0.000104623	7.08E-03
cg01419831	2	162283705		0.26	0.000104883	7.09E-03
cg06456738	11	66361081	CCDC87	0.20	0.000105108	7.10E-03
cg18198545	1	160255804	PEX19	-0.21	0.000105441	7.11E-03
cg22027433	17	62208049	ERN1	0.21	0.000105495	7.11E-03
cg07909759	10	118429813	C10orf82	0.25	0.000106566	7.17E-03
cg16940012	5	174159564		0.23	0.000106887	7.19E-03
cg00400832	7	96650323	DLX5	0.23	0.000107301	7.20E-03
cg23599771	1	190444825	FAM5C	0.21	0.000107306	7.20E-03
cg06370862	6	50680748	TFAP2D	0.26	0.000107455	7.21E-03
cg21538208	4	4864488	MSX1	0.26	0.000107456	7.21E-03
cg17526229	11	20177746		0.21	0.000107512	7.21E-03
cg16713262	16	79623751		0.21	0.00010756	7.21E-03
cg21805936	12	116008178		0.22	0.000108002	7.23E-03
cg11195797	13	100547331	CLYBL	0.29	0.000108088	7.24E-03
cg02196834	6	108484512		0.21	0.000108193	7.24E-03
cg26156687	10	102586168	PAX2	0.24	0.000108207	7.24E-03
cg00908833	6	50810432	TFAP2B	0.20	0.000108927	7.28E-03
cg25586361	11	32455444	WT1	0.21	0.000109028	7.29E-03
cg16010717	1	158609685	SPTA1	-0.21	0.00010975	7.32E-03
cg07629965	3	50312389	SEMA3B	0.21	0.0001098	7.32E-03
cg25730098	19	56346628	NLRP11	-0.24	0.00011014	7.33E-03
cg26686732	20	22562737	FOXA2	0.25	0.000110664	7.35E-03
cg07963990	2	223170370		0.21	0.000111192	7.37E-03
cg04304978	10	8809772		-0.21	0.000111339	7.38E-03
cg09918657	16	14397207	MIR193B	0.22	0.000111375	7.38E-03
cg08835113	7	96650192	DLX5	0.24	0.000111749	7.39E-03
cg23740882	20	44098387	WFDC2	0.23	0.000112923	7.45E-03
cg05469285	11	20178979	DBX1	0.26	0.000113274	7.46E-03
cg06347739	11	2292636	ASCL2	0.23	0.000113375	7.47E-03
cg02747379	16	34983912		-0.28	0.000113421	7.47E-03
cg26048630	1	170640675	PRRX1	0.21	0.00011345	7.47E-03
cg18481230	2	155555347	KCNJ3	0.21	0.000113766	7.48E-03
cg27363141	6	30038929	RNF39	0.25	0.000113776	7.48E-03
cg13667243	5	157001561	ADAM19	0.22	0.000114523	7.52E-03
cg27190239	12	10085174	CLEC2A	-0.22	0.000114804	7.54E-03
cg05166022	12	2800471	CACNA1C	0.23	0.000115019	7.54E-03
cg04637478	5	140753655	PCDHGA4	0.20	0.000115252	7.55E-03

cg13176012	2	157176971		0.21	0.000115328	7.56E-03
cg20817073	21	15071306		0.21	0.000115407	7.56E-03
cg17718728	10	117897141	GFRA1	-0.21	0.000115868	7.58E-03
cg27458485	12	99139571	ANKS1B	0.21	0.000116021	7.58E-03
cg26195178	5	1885716		0.21	0.000116159	7.59E-03
cg22894896	17	29886890	MIR193A	0.27	0.000116406	7.60E-03
cg03781931	12	85673200	ALX1	0.24	0.000116788	7.61E-03
cg01402347	4	96472269		0.22	0.000117334	7.64E-03
cg10164738	15	87667552		-0.24	0.000117437	7.64E-03
cg06982190	7	27199815		-0.25	0.000117591	7.64E-03
cg02123091	12	66261320	HMGA2	-0.23	0.000117724	7.64E-03
cg01841641	2	223169965		0.26	0.000117798	7.64E-03
cg17737621	20	21372480		0.24	0.000117867	7.65E-03
cg12435629	1	75598039	LHX8	0.20	0.000117957	7.65E-03
cg00839584	2	113542091	IL1A	-0.23	0.000118011	7.65E-03
cg00567703	12	54413101	HOXC4	0.22	0.000118783	7.68E-03
cg19046933	6	30038922	RNF39	0.28	0.000118827	7.68E-03
cg20981086	14	101923256		0.23	0.000119372	7.70E-03
cg08431693	7	99680287	ZNF3	0.21	0.000119652	7.71E-03
cg16009970	17	7198721	YBX2	0.20	0.000119717	7.71E-03
cg15571730	7	99680278	ZNF3	0.23	0.000119731	7.71E-03
cg26611765	7	27199916		-0.23	0.000120361	7.74E-03
cg04908380	1	47915862		0.23	0.000120512	7.75E-03
cg13776285	6	108490264	NR2E1	0.23	0.00012074	7.76E-03
cg21938179	3	103930387	MIR548A3	-0.20	0.000120783	7.76E-03
cg20156240	7	25891970		0.26	0.00012097	7.77E-03
cg16595261	2	242823679		0.21	0.00012129	7.79E-03
cg14160518	20	50418952	SALL4	0.23	0.000121519	7.80E-03
cg02658690	13	110885846	COL4A1	-0.24	0.000121545	7.80E-03
cg07419021	2	162274436	TBR1	0.21	0.000121852	7.81E-03
cg06660332	1	47009707		0.22	0.000122018	7.81E-03
cg05268203	2	118981748		0.25	0.000122368	7.83E-03
cg00293245	11	94884652		0.21	0.000122653	7.84E-03
cg18866529	15	62359103	C2CD4A	0.23	0.000122845	7.85E-03
cg13422850	9	135116388	NTNG2	0.22	0.00012347	7.87E-03
cg17544177	10	103589245	KCNIP2	0.21	0.000123494	7.87E-03
cg11088422	17	42392697	RUNDC3A	0.20	0.000123615	7.88E-03
cg21269897	6	27107002	HIST1H2BK	0.24	0.000124191	7.91E-03
cg10268509	16	89120300		-0.21	0.000125376	7.96E-03
cg06264984	2	38300885	CYP1B1	0.22	0.000126112	8.00E-03
cg03576469	19	46917061	CCDC8	0.25	0.00012618	8.00E-03
cg11931762	20	9488920		0.20	0.000126673	8.02E-03
cg14144305	11	44325960	ALX4	0.22	0.000126781	8.02E-03
cg02469871	12	103218581		0.25	0.000127024	8.03E-03
cg26444951	4	13530269		0.23	0.000127453	8.05E-03
cg02613827	4	41754857		0.23	0.000127576	8.05E-03
cg01089319	2	171676809	GAD1	0.21	0.000128701	8.10E-03
cg25036779	6	1615236		0.21	0.000128961	8.11E-03
cg20285974	11	526362		-0.23	0.00012898	8.11E-03
cg16996144	1	67217950	TCTEX1D1	0.21	0.000129045	8.11E-03
cg23713176	4	134074369	PCDH10	0.20	0.000129052	8.11E-03
cg13945224	9	98111970		0.21	0.000129086	8.11E-03
cg14882265	7	27184375	HOXA5	0.20	0.000129531	8.12E-03
cg07190012	11	16633647		0.21	0.000129675	8.12E-03
cg10818833	12	99139797	ANKS1B	0.20	0.000129752	8.12E-03
cg26724949	16	86610230		0.25	0.000129801	8.13E-03
cg05833851	13	100548910	CLYBL	0.26	0.000130044	8.14E-03
cg16368146	6	29943425	HCG9	0.20	0.000130533	8.16E-03
cg20403557	5	115299088	LVRN	0.21	0.000130771	8.17E-03
cg22009488	5	172656166		0.27	0.000130987	8.18E-03
cg04977834	17	70114623		0.21	0.000131998	8.22E-03
cg16718760	3	55721067	ERC2	-0.21	0.000132159	8.23E-03
cg09813525	13	53420386	PCDH8	0.22	0.000133556	8.28E-03
cg22511262	11	32455192	WT1	0.24	0.000133828	8.30E-03
cg20941258	3	46618668	LRRC2	0.21	0.000134059	8.31E-03
cg01747862	4	174457371	NBLA00301	0.23	0.000134099	8.31E-03

cg04555312	12	54389114		0.20	0.000134545	8.33E-03
cg13623495	6	10426099		0.24	0.000134609	8.33E-03
cg22557662	19	38747374	PPP1R14A	0.24	0.000134681	8.33E-03
cg22425474	6	29818086		0.23	0.000134682	8.33E-03
cg20382146	13	100617055	ZIC5	0.23	0.000134868	8.34E-03
cg04137405	14	62414451		-0.21	0.000134976	8.34E-03
cg07721569	6	10420287	TFAP2A	0.24	0.000135467	8.37E-03
cg10568624	9	100619991		0.26	0.00013569	8.37E-03
cg05474605	5	42812244	SEPP1	-0.22	0.000135721	8.37E-03
cg16904960	2	182549928		0.20	0.000135926	8.38E-03
cg24146331	10	102590225		0.23	0.000135951	8.38E-03
cg26864230	1	47911508		0.23	0.000136709	8.41E-03
cg05297666	11	128400310	ETS1	-0.22	0.000136744	8.41E-03
cg20785459	11	5265155	HBBP1	-0.25	0.000137467	8.45E-03
cg24947764	4	41881359		0.24	0.000137891	8.47E-03
cg23032993	2	3128880		-0.21	0.000137996	8.47E-03
cg08082788	14	101618406		-0.21	0.000138057	8.47E-03
cg04194947	2	177012191		0.22	0.000138602	8.49E-03
cg00539908	2	152954216	CACNB4	0.20	0.000138698	8.49E-03
cg24798398	2	176937524		0.22	0.000138832	8.49E-03
cg17014009	2	152954970	CACNB4	0.20	0.00013885	8.49E-03
cg24399712	22	39784796		0.21	0.000139813	8.54E-03
cg11627582	19	30938273	ZNF536	0.20	0.000140143	8.55E-03
cg14189391	2	25527347	DNMT3A	0.21	0.000140311	8.56E-03
cg17684657	6	10886967	SYCP2L	0.23	0.000140823	8.59E-03
cg26090775	11	16633582		0.24	0.000141001	8.60E-03
cg00457403	3	187387734	SST	0.22	0.000141243	8.60E-03
cg04897683	5	134871462	NEUROG1	0.23	0.000141482	8.61E-03
cg06530490	2	118981783		0.26	0.000141581	8.61E-03
cg05155043	2	220196620	RESP18	0.21	0.000141804	8.62E-03
cg19476053	2	223167463	CCDC140	0.21	0.000142063	8.63E-03
cg15555527	4	80885981	ANTXR2	0.21	0.000142705	8.66E-03
cg19522467	12	10270398	CLEC7A	-0.21	0.000143056	8.67E-03
cg00471645	2	11270192		0.20	0.000143206	8.67E-03
cg14072515	10	125732604		0.21	0.000143305	8.68E-03
cg00876273	10	94452311	HHEX	0.20	0.000143447	8.68E-03
cg05770946	6	100901371	SIM1	0.21	0.000143968	8.70E-03
cg12276123	2	155555157	KCNJ3	0.23	0.000144332	8.71E-03
cg05901765	5	68710818	MARVELD2	0.22	0.00014443	8.71E-03
cg17545418	16	56709853	MT1IP	0.21	0.000144439	8.71E-03
cg11747081	13	76444823		-0.24	0.000144893	8.73E-03
cg01532771	19	7827560	CLEC4M	-0.22	0.00014506	8.74E-03
cg19883813	10	8098005	GATA3	0.22	0.000145118	8.74E-03
cg08371772	19	42828621	MEGF8	0.25	0.000145932	8.77E-03
cg08750504	2	172946193		0.21	0.000146214	8.78E-03
cg24401870	13	70682525	KLHL1	0.20	0.000146777	8.80E-03
cg01574513	16	85981720		0.23	0.000146839	8.81E-03
cg06702880	4	155663094		0.21	0.000148017	8.85E-03
cg13512268	7	27183973	HOXA5	0.21	0.000148064	8.85E-03
cg22682811	20	21372462		0.24	0.000148123	8.85E-03
cg08188903	14	100680826		0.23	0.000148635	8.87E-03
cg22473770	17	29647977	EVI2A	-0.21	0.000149509	8.91E-03
cg02120658	2	223160297	PAX3	0.21	0.000150071	8.94E-03
cg01840333	6	26225767	HIST1H3E	0.22	0.000150418	8.95E-03
cg17039236	18	77548049		0.24	0.000150495	8.95E-03
cg00934355	9	1049244	DMRT2	0.24	0.000150672	8.95E-03
cg13574488	10	119306246	EMX2	0.24	0.000152117	9.01E-03
cg02955548	13	108028239	FAM155A	-0.20	0.000152585	9.02E-03
cg23793213	6	30039006	RNF39	0.25	0.000152993	9.04E-03
cg02668248	19	16437789	KLF2	0.23	0.000153016	9.04E-03
cg26303165	1	228401758	OBSCN	0.25	0.000153462	9.06E-03
cg24534742	13	43149281	TNFSF11	0.22	0.000154032	9.09E-03
cg16997642	7	27184159	HOXA5	0.24	0.000154355	9.10E-03
cg17814014	10	134119998	STK32C	-0.20	0.000154792	9.12E-03
cg20314548	15	74426273	ISLR2	0.20	0.000155456	9.14E-03
cg06215107	4	151504725	LRBA	0.21	0.000155947	9.15E-03

cg14845689	10	119306193	EMX2	0.20	0.000156121	9.16E-03
cg07691152	12	103355123		0.21	0.000156134	9.16E-03
cg08832220	3	55516184	WNT5A	0.23	0.000156286	9.16E-03
cg06014958	6	10425553		0.23	0.000156294	9.16E-03
cg15339231	7	96650407	DLX5	0.23	0.000156702	9.18E-03
cg21249376	9	27528432	MOBKL2B	0.21	0.000157131	9.19E-03
cg10474712	22	42062693		0.22	0.000157864	9.23E-03
cg08866876	8	20170359		-0.21	0.000158561	9.25E-03
cg10574365	1	32054836		0.20	0.000158677	9.26E-03
cg11306628	7	560119	PDGFA	0.22	0.000158735	9.26E-03
cg13060157	1	113286610		0.23	0.000158928	9.27E-03
cg20916523	3	10184584	VHL	0.23	0.000158974	9.27E-03
cg06464008	10	102881508	TLX1NB	0.24	0.000159205	9.27E-03
cg00347563	1	78511600	GIPC2	0.25	0.000159456	9.28E-03
cg01488770	17	59474078		0.24	0.000159893	9.30E-03
cg05650260	6	50692762	TFAP2D	0.23	0.000160079	9.30E-03
cg14298457	2	177012117		0.22	0.000160112	9.30E-03
cg18746336	3	128216122		0.22	0.000160196	9.30E-03
cg13936452	1	47911336		0.24	0.000160231	9.30E-03
cg12127282	2	177014685	HOXD4	0.21	0.00016063	9.32E-03
cg00126148	4	111533517		0.23	0.00016197	9.36E-03
cg20090108	11	88242488	GRM5	0.22	0.00016263	9.39E-03
cg25901381	7	27225485	HOXA11AS	0.21	0.000163054	9.41E-03
cg23244913	6	29943401	HCG9	0.22	0.000163093	9.41E-03
cg21144922	1	109204168	C1orf59	0.23	0.000163217	9.41E-03
cg14947429	10	28036151	MKX	0.22	0.000164581	9.47E-03
cg08297751	4	174448705	HAND2	0.23	0.000164645	9.47E-03
cg19554457	12	93774772	NUDT4	0.22	0.000164802	9.48E-03
cg08506931	2	223168861	CCDC140	0.21	0.000165494	9.51E-03
cg21275135	6	99273679		0.21	0.000165707	9.52E-03
cg02798576	10	101290168		0.25	0.000165781	9.52E-03
cg23845450	1	75597887	LHX8	0.25	0.000166081	9.52E-03
cg27299406	2	43020013	HAAO	0.21	0.000167128	9.56E-03
cg05379541	7	27252541		0.20	0.000167418	9.57E-03
cg10413944	2	224702734	AP1S3	0.25	0.000167654	9.57E-03
cg13916459	12	85673221	ALX1	0.24	0.000167817	9.58E-03
cg25310592	11	5174413	OR52A1	-0.24	0.000168286	9.59E-03
cg27587125	1	109204207	C1orf59	0.24	0.000168436	9.60E-03
cg13834146	15	77927305		0.22	0.000169156	9.63E-03
cg08941355	7	27133106	HOXA1	0.22	0.000169436	9.64E-03
cg00868875	18	24127237	KCTD1	0.26	0.000169958	9.66E-03
cg03709307	17	37760652	NEUROD2	0.20	0.000170028	9.66E-03
cg18775634	3	27755341		0.26	0.000170083	9.66E-03
cg06393354	1	248366332	OR2M3	-0.20	0.000170357	9.68E-03
cg09765089	7	27291346		0.23	0.000170359	9.68E-03
cg12174804	8	18244861		0.24	0.000171894	9.75E-03
cg26846424	1	3310103	PRDM16	0.21	0.000172098	9.76E-03
cg05529816	17	4802735	CHRNE	0.23	0.000172755	9.79E-03
cg13681468	4	90227879	GPRIN3	0.20	0.00017372	9.83E-03
cg07540421	2	71115680		0.23	0.000174591	9.86E-03
cg00681106	1	75599532	LHX8	0.22	0.000175242	9.88E-03
cg12002303	15	68113478		0.23	0.000175433	9.89E-03
cg08582485	20	22557518	C20orf56	0.23	0.00017566	9.90E-03
cg00187686	11	59634218	TCN1	-0.23	0.000176119	9.91E-03
cg26078436	11	5265992	HBBP1	-0.20	0.000176199	9.92E-03
cg18862481	3	129693370	TRH	0.26	0.000176649	9.93E-03
cg03026373	17	36715787	SRCIN1	0.25	0.000177176	9.95E-03
cg02185052	10	94452480	HHEX	0.20	0.000177283	9.95E-03
cg14925024	1	228291705	C1orf35	-0.20	0.000177396	9.95E-03
cg08243612	2	219763536		0.21	0.000177449	9.95E-03
cg05768558	1	26737005	LIN28	0.20	0.000177717	9.96E-03
cg05774229	11	76033097		0.20	0.000178261	9.98E-03
cg14218275	10	11047435	CUGBP2	0.24	0.000178556	1.00E-02
cg00325866	15	62359130	C2CD4A	0.22	0.000179097	1.00E-02
cg03656099	17	59481954	TBX2	0.24	0.000179244	1.00E-02
cg06606199	12	115174773		0.22	0.000179682	1.00E-02

cg27258399	8	38832267	HTRA4	0.20	0.000179858	1.00E-02
cg04079760	16	68269694	ESRP2	0.22	0.000180202	1.01E-02
cg14185918	9	110249966	KLF4	0.21	0.000180411	1.01E-02
cg04134240	14	100680625		0.22	0.000181046	1.01E-02
cg16386396	1	3132458	PRDM16	-0.21	0.000181704	1.01E-02
cg11809668	4	154435522	KIAA0922	-0.24	0.00018178	1.01E-02
cg19204131	6	10385320		0.24	0.000181938	1.01E-02
cg14658493	7	27184077	HOXA5	0.21	0.000182025	1.01E-02
cg03448548	16	33604228		0.20	0.000182468	1.01E-02
cg06422471	22	51169500	SHANK3	0.20	0.000183	1.01E-02
cg19143639	8	66472813		0.21	0.000183489	1.02E-02
cg26720125	21	38077673	SIM2	0.22	0.000184056	1.02E-02
cg04621893	10	22624687		0.23	0.000184174	1.02E-02
cg10638793	3	169027604	MECOM	-0.22	0.000184561	1.02E-02
cg15655935	11	59815177	PLAC1L	-0.24	0.00018539	1.02E-02
cg25304107	2	177017331	HOXD4	0.21	0.000185443	1.02E-02
cg27148952	6	49518347	C6orf141	0.21	0.000185686	1.02E-02
cg17651821	6	27841683	HIST1H4L	0.24	0.000185869	1.02E-02
cg19237691	1	1141617	TNFRSF18	0.23	0.000185899	1.02E-02
cg06418871	5	68710831	MARVELD2	0.22	0.000186008	1.02E-02
cg12571423	13	37006748	CCNA1	0.20	0.000186126	1.02E-02
cg26043322	1	159507162	OR10J5	-0.20	0.000186499	1.03E-02
cg16520288	12	58014857	SLC26A10	0.22	0.000186535	1.03E-02
cg06813578	15	51386254	TNFAIP8L3	0.23	0.000186844	1.03E-02
cg20899581	6	27841230	HIST1H4L	0.21	0.000186908	1.03E-02
cg12055395	7	96642605	DLX6AS	0.23	0.000186934	1.03E-02
cg13647052	12	2800382	CACNA1C	0.21	0.000187501	1.03E-02
cg25158147	10	54073150	DKK1	0.22	0.000187574	1.03E-02
cg12015737	7	27184030	HOXA5	0.24	0.000188415	1.03E-02
cg21251125	9	126771781		0.21	0.000188476	1.03E-02
cg05131488	7	14031822	ETV1	-0.23	0.000190125	1.04E-02
cg03074925	6	10425625		0.21	0.000190273	1.04E-02
cg22078710	15	53087599		0.21	0.000190647	1.04E-02
cg02924487	1	3649344	TP73	0.20	0.000191625	1.04E-02
cg11294513	19	58220295	ZNF154	0.22	0.000191921	1.04E-02
cg08292023	11	88242253	GRM5	0.22	0.000193241	1.05E-02
cg06710082	6	29943408	HCG9	0.22	0.000193438	1.05E-02
cg08962452	12	54366816	HOXC11	0.21	0.000193613	1.05E-02
cg02636041	10	43698008	RASGEF1A	0.26	0.000194123	1.05E-02
cg17485021	4	13530094		0.25	0.000194166	1.05E-02
cg20278291	14	57272340	OTX2	0.23	0.000194398	1.05E-02
cg19909865	4	134074421	PCDH10	0.22	0.000194496	1.05E-02
cg19643053	7	27184271	HOXA5	0.23	0.000194533	1.05E-02
cg23806894	4	111535528		0.27	0.000195498	1.06E-02
cg11145302	10	134556533	INPP5A	-0.21	0.00019571	1.06E-02
cg25399352	12	114837572	TBX5	0.22	0.000196747	1.06E-02
cg04797936	12	49659154	TUBA1C	0.21	0.000197177	1.06E-02
cg10828127	21	41550814	DSCAM	0.22	0.000198241	1.07E-02
cg17163168	1	3600735	TP73	0.20	0.000199078	1.07E-02
cg15984661	19	46916520	CCDC8	0.23	0.000199191	1.07E-02
cg14184866	9	82185137		0.21	0.000199708	1.07E-02
cg02365780	2	101437872	NPAS2	0.20	0.000199796	1.07E-02
cg25458175	19	59025570	ZBTB45	0.22	0.000200347	1.07E-02
cg05026135	19	30713427		0.20	0.000200666	1.07E-02
cg23810282	5	43037519		0.23	0.000200979	1.07E-02
cg18676790	7	97680731		0.20	0.000201693	1.08E-02
cg13557804	6	33117976		-0.23	0.000201734	1.08E-02
cg19318393	1	223936508	CAPN2	0.25	0.0002018	1.08E-02
cg26667091	11	65352232	EHBP1L1	0.21	0.000202092	1.08E-02
cg20755170	5	145720024	POU4F3	0.20	0.000202444	1.08E-02
cg10386298	1	221050491		0.20	0.000202787	1.08E-02
cg09481404	15	53087316		0.20	0.000203089	1.08E-02
cg12851717	13	100517009	CLYBL	0.25	0.000203552	1.08E-02
cg04309093	14	92980608	RIN3	0.21	0.000203864	1.08E-02
cg07304175	3	181414139	SOX2OT	0.21	0.000204243	1.08E-02
cg18537730	19	49250080	IZUMO1	0.22	0.000204532	1.08E-02

cg15279308	12	2800500	CACNA1C	0.22	0.00020503	1.09E-02
cg18356785	11	47611780	C1QTNF4	0.23	0.000205474	1.09E-02
cg03898577	5	140787013	PCDHGA4	0.21	0.000205934	1.09E-02
cg02622316	6	28367410	ZSCAN12	0.22	0.000206071	1.09E-02
cg14560430	3	32863175	TRIM71	0.23	0.00020743	1.10E-02
cg15296664	12	89748773		0.22	0.000207465	1.10E-02
cg16328007	1	41119988	RIMS3	0.22	0.000207943	1.10E-02
cg25984050	4	13530357		0.21	0.000208251	1.10E-02
cg10343447	16	68544200		0.25	0.000209556	1.10E-02
cg09533428	6	30038897	RNF39	0.20	0.000211082	1.11E-02
cg09748975	4	4864532	MSX1	0.23	0.000211488	1.11E-02
cg06799422	15	41952235	MGA	0.21	0.00021175	1.11E-02
cg14058329	7	27183946	HOXA5	0.20	0.000211808	1.11E-02
cg23267558	13	70682604	KLHL1	0.21	0.000212624	1.11E-02
cg19250799	1	47910456		0.23	0.000213304	1.11E-02
cg18698431	12	105956397		-0.20	0.000214511	1.12E-02
cg07046369	10	102586254	PAX2	0.23	0.000214663	1.12E-02
cg18343292	11	60146078	MS4A7	-0.22	0.000215156	1.12E-02
cg03102494	1	147801721		0.21	0.000215652	1.12E-02
cg07260927	4	11428822	HS3ST1	0.21	0.000217263	1.13E-02
cg04603812	16	1429265	UNKL	0.21	0.000217584	1.13E-02
cg22702772	3	48699012	CELSR3	0.20	0.000217725	1.13E-02
cg19049194	2	175193754		0.21	0.000217985	1.13E-02
cg01089498	4	11428985	HS3ST1	0.24	0.000218614	1.13E-02
cg22134372	15	52455211	GNB5	-0.23	0.000219424	1.13E-02
cg14601621	9	97848457	C9orf3	-0.22	0.000220195	1.13E-02
cg02553516	21	38078767	SIM2	0.21	0.000220549	1.14E-02
cg14227486	4	41867415		0.25	0.000221298	1.14E-02
cg07615497	4	147554144		0.21	0.000221418	1.14E-02
cg12905085	1	32054510		0.22	0.000221762	1.14E-02
cg07126525	6	166218519		0.24	0.00022214	1.14E-02
cg04294437	16	50875204		0.22	0.000223004	1.14E-02
cg09124518	10	134119682	STK32C	-0.22	0.000224378	1.15E-02
cg03607708	1	215966091	USH2A	0.20	0.000225674	1.15E-02
cg12408601	6	27841665	HIST1H4L	0.24	0.000226603	1.16E-02
cg20611680	1	16554171		0.21	0.000226762	1.16E-02
cg01141459	22	30476452	HORMAD2	0.21	0.00022945	1.17E-02
cg01738522	4	8690035		0.20	0.000229903	1.17E-02
cg04074010	10	130227803		-0.20	0.000233685	1.18E-02
cg11265749	7	560499	PDGFA	0.21	0.000233792	1.18E-02
cg12349884	6	27106988	HIST1H2BK	0.25	0.000234246	1.18E-02
cg00455424	1	65468815		0.24	0.000235476	1.18E-02
cg13492103	1	75596827	LHX8	0.21	0.000236236	1.19E-02
cg13364230	1	35258779	GJA4	0.21	0.000236779	1.19E-02
cg02331910	1	153599831	S100A13	0.21	0.000237588	1.19E-02
cg17400476	7	27231491		0.22	0.000238997	1.19E-02
cg18610930	21	46237456	SUMO3	0.22	0.00023965	1.20E-02
cg16786102	4	41867462		0.22	0.000239798	1.20E-02
cg08390877	2	119615998		0.24	0.000240198	1.20E-02
cg26092675	6	26225258	HIST1H3E	0.27	0.000240241	1.20E-02
cg17446896	6	27841633	HIST1H4L	0.23	0.000240908	1.20E-02
cg08793877	2	71115994		0.21	0.000240991	1.20E-02
cg26668276	7	96633311	DLX6AS	0.23	0.000241346	1.20E-02
cg09780241	4	111535597		0.27	0.00024257	1.21E-02
cg25541968	11	7848142	OR5P3	-0.22	0.000244252	1.21E-02
cg03043296	15	83349420	AP3B2	0.20	0.000244573	1.21E-02
cg02571816	19	38747378	PPP1R14A	0.21	0.00024525	1.22E-02
cg16933440	3	157217435	VEPH1	0.23	0.000245666	1.22E-02
cg18157027	1	228400693	OBSCN	0.21	0.000245771	1.22E-02
cg09784307	15	75248768	RPP25	0.22	0.000246437	1.22E-02
cg22969763	12	10607538	KLRC1	-0.22	0.00024783	1.22E-02
cg24932457	1	175568559	TNR	0.24	0.000247956	1.22E-02
cg21587238	5	42953590		0.25	0.000249263	1.23E-02
cg25320749	6	28494732	GPX5	-0.21	0.000252028	1.23E-02
cg21611810	12	114841870	TBX5	0.22	0.00025284	1.24E-02
cg18211066	17	75956222		0.23	0.000252974	1.24E-02

cg25957967	1	55505148	PCSK9	0.21	0.000254387	1.24E-02
cg16312002	1	16554558		0.21	0.000255129	1.24E-02
cg21968580	1	78470640	DNAJB4	0.23	0.000255161	1.24E-02
cg25628429	11	50005457	OR4C12	-0.21	0.000255492	1.25E-02
cg18494190	10	102589532	PAX2	0.23	0.000257141	1.25E-02
cg11784990	12	103218665		0.23	0.000258188	1.25E-02
cg12621514	10	54073047	DKK1	0.24	0.000258748	1.26E-02
cg24804544	7	6544107	GRID2IP	0.26	0.000262608	1.27E-02
cg06419850	17	4688683	VMO1	0.22	0.000262948	1.27E-02
cg21007852	7	27203546	HOXA9	-0.20	0.00026813	1.28E-02
cg22784386	6	100903817	SIM1	0.22	0.000268513	1.28E-02
cg18757468	11	65352168	EHBP1L1	0.22	0.000268781	1.28E-02
cg15706223	1	1141934	TNFRSF18	0.21	0.000269076	1.29E-02
cg14098847	14	101923218		0.20	0.000269121	1.29E-02
cg18425434	10	118429648	C10orf82	0.22	0.00027049	1.29E-02
cg01920334	21	48087978		0.22	0.000270542	1.29E-02
cg24292761	6	117584665		0.21	0.000272795	1.30E-02
cg27095915	6	1624442	GMDS	0.24	0.000275237	1.30E-02
cg03928961	3	196693980	PIGZ	0.23	0.000275397	1.30E-02
cg25365934	10	77155376		0.23	0.000275898	1.31E-02
cg04268624	5	43037285		0.23	0.000276746	1.31E-02
cg04838988	8	70984437	PRDM14	0.22	0.000276754	1.31E-02
cg18384926	15	45722598	C15orf48	0.22	0.000277897	1.31E-02
cg14509403	22	30476281	HORMAD2	0.22	0.000278267	1.31E-02
cg27017658	14	21093081		0.22	0.000278742	1.32E-02
cg19216792	7	26897202	SKAP2	0.21	0.000279543	1.32E-02
cg17536595	6	43612908	RSPH9	0.21	0.000282299	1.33E-02
cg23238231	18	22928586	ZNF521	0.21	0.000282481	1.33E-02
cg06059069	15	32964277	SCG5	-0.21	0.000283051	1.33E-02
cg25800765	12	85673347	ALX1	0.23	0.000284669	1.33E-02
cg27205487	21	17961996	C21orf34	-0.23	0.000285913	1.34E-02
cg09264282	1	86622395	COL24A1	0.21	0.000286765	1.34E-02
cg04623837	6	29943414	HCG9	0.21	0.000287948	1.34E-02
cg27527108	1	116711075		0.23	0.000288415	1.34E-02
cg26371771	6	49518360	C6orf141	0.20	0.000289992	1.35E-02
cg01912955	6	100906383	SIM1	0.22	0.000290834	1.35E-02
cg11172629	10	11047354	CUGBP2	0.23	0.000293096	1.36E-02
cg14909329	10	134120029	STK32C	-0.25	0.000294595	1.36E-02
cg04374711	16	31008960	STX1B	0.24	0.000294728	1.36E-02
cg00337916	7	96643686	DLX6AS	0.20	0.000296492	1.37E-02
cg21654383	2	85641076		0.23	0.000296785	1.37E-02
cg07984133	13	28544592	CDX2	0.23	0.000297233	1.37E-02
cg26051413	11	2292902	ASCL2	0.23	0.000297797	1.37E-02
cg04151062	1	116710943		0.20	0.000298467	1.37E-02
cg05136263	2	45236019	SIX2	0.23	0.000301296	1.38E-02
cg07211768	12	115102290		0.21	0.000301303	1.38E-02
cg19560588	7	25892614		0.20	0.000302587	1.38E-02
cg27552679	15	74425757	ISLR2	0.22	0.000302793	1.38E-02
cg13546414	14	37053664		0.21	0.000302808	1.38E-02
cg03972071	18	72917163	ZADH2	0.25	0.000303001	1.39E-02
cg14962509	1	36039655	TFAP2E	0.25	0.000304035	1.39E-02
cg18794404	10	22542024		0.21	0.000304772	1.39E-02
cg01997272	14	103740476		0.21	0.000305134	1.39E-02
cg00294684	11	5602123	OR52B6	-0.22	0.000306291	1.39E-02
cg04373435	2	79738582	CTNNA2	0.22	0.000307579	1.40E-02
cg21608600	2	45228716		0.20	0.000309853	1.40E-02
cg22721579	1	36787932	FAM176B	0.23	0.00030991	1.40E-02
cg09465698	5	140787623	PCDHGA4	0.20	0.000312468	1.41E-02
cg16838838	2	85641023		0.20	0.000312528	1.41E-02
cg13292042	4	111535334		0.25	0.00031261	1.41E-02
cg27565555	20	55839663	BMP7	0.25	0.000313603	1.42E-02
cg25751371	12	49659053	TUBA1C	0.21	0.00031478	1.42E-02
cg10074727	6	10883105	GCM2	0.21	0.000318207	1.43E-02
cg01947943	6	29012859	OR2W1	-0.21	0.00031875	1.43E-02
cg09880291	7	27184109	HOXA5	0.20	0.000320513	1.43E-02
cg14434755	12	115107540		0.20	0.000320562	1.43E-02

cg00804587	3	190038679	CLDN1	-0.20	0.000321189	1.44E-02
cg06097659	1	1141991	TNFRSF18	0.24	0.000323248	1.44E-02
cg25851651	19	14939300	OR7A5	-0.20	0.000323687	1.44E-02
cg05020759	8	16240240		-0.22	0.000325674	1.45E-02
cg07258300	2	127963590	CYP27C1	-0.21	0.000325861	1.45E-02
cg18950778	11	32455362	WT1	0.21	0.000325966	1.45E-02
cg02194223	7	27285433	EVX1	0.20	0.000326137	1.45E-02
cg08832624	7	98099917		0.20	0.000326542	1.45E-02
cg14996220	12	85673270	ALX1	0.21	0.000329229	1.46E-02
cg26109568	2	105697685	MRPS9	0.20	0.00032991	1.46E-02
cg24113409	6	10884314		0.20	0.000330321	1.46E-02
cg13740135	19	44006432	PHLDB3	0.20	0.000332179	1.46E-02
cg20238681	15	94443233		0.21	0.000332686	1.47E-02
cg13622450	9	91147514		0.21	0.000333471	1.47E-02
cg23680821	1	109204316	C1orf59	0.20	0.000333626	1.47E-02
cg10947408	10	77155404		0.21	0.000334608	1.47E-02
cg04352272	17	3289798		0.21	0.000334854	1.47E-02
cg13910040	1	153438468		0.22	0.000336776	1.48E-02
cg01833675	2	225266876	FAM124B	-0.21	0.000337881	1.48E-02
cg25339566	1	67218165	TCTEX1D1	0.25	0.000338051	1.48E-02
cg09426383	7	26897182	SKAP2	0.22	0.00034231	1.49E-02
cg09052548	10	11047368	CUGBP2	0.23	0.000342701	1.49E-02
cg06747432	19	46916741	CCDC8	0.24	0.000343406	1.49E-02
cg21596313	6	74233269		0.22	0.000343516	1.49E-02
cg06504526	6	100894629	SIM1	0.23	0.000343614	1.49E-02
cg22367191	6	10425648		0.23	0.000345433	1.50E-02
cg11798857	20	22566289	FOXA2	0.22	0.000345467	1.50E-02
cg20377305	7	96650668	DLX5	0.23	0.000346354	1.50E-02
cg20254225	2	38301438	CYP1B1	0.21	0.000347392	1.50E-02
cg12433473	2	220406756	CHPF	0.20	0.000347864	1.50E-02
cg27404023	2	162283994		0.20	0.000348369	1.51E-02
cg20998319	2	157177345		0.22	0.000349362	1.51E-02
cg02373784	4	13537302		0.21	0.000350779	1.51E-02
cg02780919	11	9026308	NRIP3	0.22	0.000352552	1.52E-02
cg26365623	11	5144101	OR52A4	-0.22	0.000359252	1.53E-02
cg11789534	22	37655614		0.21	0.000361164	1.54E-02
cg14987431	10	94455650		0.21	0.000362324	1.54E-02
cg09072162	1	55505180	PCSK9	0.20	0.000363119	1.55E-02
cg17286640	4	55991202	KDR	0.22	0.000366471	1.55E-02
cg18562243	3	181414189	SOX2OT	0.21	0.000366578	1.55E-02
cg20386316	8	72470982		0.21	0.000366902	1.55E-02
cg06374962	4	111531926		0.22	0.000368919	1.56E-02
cg01871214	4	41881417		0.25	0.00036906	1.56E-02
cg23234847	14	20611696	OR4N5	0.20	0.000370233	1.56E-02
cg26196480	12	114846347	TBX5	0.23	0.000371956	1.57E-02
cg23741006	11	59634191	TCN1	-0.21	0.000372604	1.57E-02
cg07615087	21	38077347	SIM2	0.21	0.000372769	1.57E-02
cg06911121	12	114847164	TBX5	0.24	0.000373034	1.57E-02
cg14886269	1	1141927	TNFRSF18	0.22	0.00037355	1.57E-02
cg23586322	8	121138232	COL14A1	0.22	0.000374511	1.58E-02
cg16898193	12	54377550	HOXC10	0.23	0.000377984	1.58E-02
cg22387369	15	85360691	ALPK3	0.20	0.000379062	1.59E-02
cg01869219	2	224702789	AP1S3	0.21	0.0003849	1.60E-02
cg12126857	1	35384669		0.21	0.000388412	1.61E-02
cg13443627	1	59043070	TACSTD2	0.22	0.000390305	1.62E-02
cg17481703	13	76444831		-0.21	0.00039141	1.62E-02
cg10933003	4	174448341	HAND2	0.22	0.000392886	1.62E-02
cg13406860	9	132881336	GPR107	0.20	0.000393881	1.63E-02
cg08971667	7	27201728		-0.21	0.000395601	1.63E-02
cg17284804	1	67218135	TCTEX1D1	0.22	0.000395817	1.63E-02
cg00748875	5	140175116	PCDHA1	0.20	0.000396491	1.63E-02
cg26325335	3	50402333	CACNA2D2	0.20	0.000398847	1.64E-02
cg21429725	3	196693809	PIGZ	0.25	0.000400288	1.64E-02
cg01518889	3	128202493	GATA2	0.20	0.00040131	1.65E-02
cg02455820	16	51147769		0.24	0.000402883	1.65E-02
cg12040278	2	37572396	QPCT	0.22	0.000403353	1.65E-02

cg09496464	2	208904568		-0.20	0.000404542	1.65E-02
cg04922681	13	43149234	TNFSF11	0.22	0.000404703	1.66E-02
cg10272601	9	95947146	WNK2	0.24	0.000404744	1.66E-02
cg12738347	15	53096854		0.21	0.000410619	1.67E-02
cg19525418	8	61765432	CHD7	-0.23	0.00041082	1.67E-02
cg06691343	8	53326305		-0.20	0.000412355	1.67E-02
cg17355294	19	51416098		0.21	0.000417418	1.69E-02
cg16201038	13	28544760	CDX2	0.23	0.000421765	1.70E-02
cg06282446	5	140174554	PCDHA1	0.23	0.000422287	1.70E-02
cg08915171	2	105273376		-0.21	0.000423636	1.70E-02
cg19693890	6	168377714	HGC6.3	-0.22	0.000426968	1.71E-02
cg02272795	19	15279244	NOTCH3	-0.23	0.000430169	1.72E-02
cg10425861	15	60286540		0.20	0.000430515	1.72E-02
cg25200152	1	147790458		0.21	0.000433241	1.73E-02
cg03728046	7	6566428	GRID2IP	0.21	0.000435706	1.74E-02
cg23063647	4	206339	ZNF876P	0.20	0.000437413	1.74E-02
cg25412017	5	175560449		0.21	0.000437441	1.74E-02
cg03585912	6	10385776		0.22	0.000437585	1.74E-02
cg21854286	17	40700858		0.21	0.000437821	1.74E-02
cg02369542	1	41119940	RIMS3	0.21	0.000439496	1.74E-02
cg26947626	8	72469430		0.23	0.000439661	1.74E-02
cg15400671	7	34004484	BMPER	-0.21	0.000442766	1.75E-02
cg02348751	4	183064035	MGC45800	0.22	0.000443342	1.75E-02
cg23588462	13	95357223		0.21	0.000445371	1.76E-02
cg09415754	6	10385392		0.22	0.000445907	1.76E-02
cg05844937	6	1614141		0.24	0.000449221	1.77E-02
cg24873592	1	32042092	TINAGL1	0.22	0.000449549	1.77E-02
cg13836098	6	26225268	HIST1H3E	0.25	0.000449612	1.77E-02
cg24177393	5	43037517		0.22	0.000450205	1.77E-02
cg07915921	12	54321502		0.23	0.000450501	1.77E-02
cg20615879	10	102892391	TLX1NB	0.21	0.000450795	1.77E-02
cg05661282	19	58220370	ZNF154	0.22	0.000454806	1.78E-02
cg20274304	8	28176295	PNOC	-0.22	0.000459374	1.79E-02
cg12062156	10	2487194		-0.20	0.000460421	1.79E-02
cg04227922	2	119615496		0.24	0.000463272	1.80E-02
cg20616414	9	95947396	WNK2	0.21	0.000468651	1.81E-02
cg08540622	11	59806898	PLAC1L	-0.20	0.000472934	1.82E-02
cg06792065	13	20711179		0.20	0.000474381	1.83E-02
cg20806345	2	182543706	NEUROD1	0.24	0.000482391	1.85E-02
cg19905856	10	118924620		0.22	0.000484157	1.85E-02
cg08505647	11	59807594	PLAC1L	-0.22	0.000488135	1.86E-02
cg27178293	12	54371246		0.21	0.00049376	1.87E-02
cg24446586	7	27225078	HOXA11AS	0.21	0.000495858	1.88E-02
cg02478379	5	170814123	NPM1	0.20	0.000496121	1.88E-02
cg12259469	7	39017305	POU6F2	-0.22	0.000496726	1.88E-02
cg15470836	13	100648306		0.21	0.000498034	1.88E-02
cg02767242	2	162274424	TBR1	0.21	0.00049857	1.89E-02
cg23971170	1	165326269	LMX1A	0.20	0.000504007	1.90E-02
cg17080163	2	123418757		0.21	0.000505248	1.90E-02
cg09861917	20	22566877	FOXA2	0.22	0.000505562	1.90E-02
cg17976229	11	5653336	TRIM34	-0.23	0.000507038	1.91E-02
cg19370653	4	111550040	PITX2	0.23	0.000512154	1.92E-02
cg22160073	11	5653405	TRIM34	-0.22	0.000512815	1.92E-02
cg25480336	20	50720908	ZFP64	0.23	0.000514159	1.92E-02
cg18653451	19	46917018	CCDC8	0.21	0.000516945	1.93E-02
cg09938227	1	156390124	C1orf61	0.20	0.000520213	1.94E-02
cg24335456	8	58106155		-0.22	0.000523829	1.95E-02
cg18417953	18	55097461		0.21	0.000525368	1.95E-02
cg22876812	2	71116188		0.22	0.000526731	1.96E-02
cg23352271	11	49915274		-0.21	0.000530356	1.97E-02
cg14270434	9	100613647		0.20	0.000534241	1.98E-02
cg08808042	16	88053104	BANP	0.21	0.000537978	1.98E-02
cg01838004	9	100070007	KIAA1529	0.21	0.000539095	1.99E-02
cg14409810	9	140172908	C9orf167	0.21	0.000541703	1.99E-02
cg15553770	11	5531524	UBQLN3	-0.22	0.000550498	2.01E-02
cg08667128	17	29886885	MIR193A	0.25	0.000551663	2.02E-02

cg19250101	19	54666215	TMC4	0.20	0.000555461	2.03E-02
cg09495769	7	27225342	HOXA11AS	0.20	0.000556886	2.03E-02
cg24376337	11	56430801	OR5AR1	-0.22	0.000557865	2.03E-02
cg02764478	6	100904316	SIM1	0.21	0.000559267	2.03E-02
cg21775675	5	87441487		0.20	0.000559866	2.04E-02
cg02513017	16	68544286		0.21	0.000561675	2.04E-02
cg06994420	2	66672553	MEIS1	0.20	0.000563572	2.04E-02
cg19513071	5	87441918		0.21	0.000565864	2.05E-02
cg24267283	3	181414021	SOX2OT	0.20	0.000572487	2.06E-02
cg11469908	2	66918305		0.21	0.000580915	2.09E-02
cg05857996	20	2675418	EBF4	0.23	0.000581271	2.09E-02
cg01544903	16	56697229		0.24	0.000583348	2.09E-02
cg05325193	20	22558233	C20orf56	0.23	0.000588692	2.10E-02
cg12548057	7	73389660		-0.20	0.000591446	2.11E-02
cg18913890	2	79738619	CTNNA2	0.21	0.000597845	2.12E-02
cg11053632	16	88053094	BANP	0.20	0.000598019	2.12E-02
cg04685570	12	114841202	TBX5	0.21	0.000598612	2.12E-02
cg16591182	8	77602161	ZFHX4	-0.22	0.000602433	2.13E-02
cg26081162	6	49518449	C6orf141	0.21	0.000605261	2.14E-02
cg21377260	7	1856979	MAD1L1	-0.21	0.000614444	2.16E-02
cg09502015	5	87441873		0.21	0.000614753	2.16E-02
cg14632030	11	60144545	MS4A7	-0.22	0.000616944	2.17E-02
cg00880018	13	43149171	TNFSF11	0.21	0.000620883	2.18E-02
cg15310637	12	14927542	H2AFJ	0.23	0.000621162	2.18E-02
cg05835982	3	129693791	TRH	0.22	0.00062462	2.18E-02
cg01872719	14	106744392		-0.20	0.000627026	2.19E-02
cg11930908	13	23500752		0.23	0.000631305	2.20E-02
cg24722073	4	41882163		0.21	0.000633062	2.20E-02
cg15160263	5	76923943		0.20	0.000637738	2.21E-02
cg06091881	21	48087589		0.21	0.000638974	2.22E-02
cg26224671	13	43148935	TNFSF11	0.23	0.000640322	2.22E-02
cg00469602	2	172955930		0.20	0.00065278	2.25E-02
cg17929238	4	111545628	PITX2	0.21	0.00065598	2.25E-02
cg14862207	17	36715798	SRCIN1	0.23	0.000658356	2.26E-02
cg04249522	6	166218512		0.21	0.000658972	2.26E-02
cg07991112	6	27841423	HIST1H4L	0.24	0.000671137	2.28E-02
cg18960642	6	39281694	KCNK17	0.23	0.000677724	2.30E-02
cg21453451	3	181414237	SOX2OT	0.20	0.000690563	2.33E-02
cg14871932	7	44185323	GCK	0.20	0.000693651	2.34E-02
cg01064265	16	55363058	IRX6	0.21	0.000693889	2.34E-02
cg11841394	12	114841536	TBX5	0.23	0.000704215	2.36E-02
cg21033440	11	65409861	SIPA1	0.22	0.000710993	2.37E-02
cg24354581	21	38079615	SIM2	0.21	0.000711453	2.37E-02
cg24362812	18	11148769	FAM38B	0.21	0.000729708	2.41E-02
cg00181125	12	22095182		0.20	0.000733592	2.42E-02
cg06276084	6	168377594	HGC6.3	-0.20	0.000745591	2.45E-02
cg11836236	10	118893224	VAX1	0.22	0.00075431	2.46E-02
cg04178787	2	123418787		0.21	0.00075533	2.47E-02
cg10464312	2	66672688	MEIS1	0.20	0.000758392	2.47E-02
cg24452128	6	10390919		0.24	0.000763557	2.48E-02
cg01340542	1	65468273		0.21	0.000773799	2.51E-02
cg08394412	16	50875140		0.20	0.000780261	2.52E-02
cg03610852	8	65287050		0.21	0.000781829	2.52E-02
cg22790931	7	39017455	POU6F2	-0.20	0.000790493	2.54E-02
cg11614451	3	160167729	TRIM59	0.20	0.000791695	2.54E-02
cg09339818	6	100914480		0.23	0.000798497	2.55E-02
cg10263682	3	181413847	SOX2OT	0.21	0.000798699	2.55E-02
cg05106770	4	128545193		0.24	0.000813438	2.58E-02
cg11988964	10	54073189	DKK1	0.22	0.000851698	2.66E-02
cg00890363	4	13540673		0.22	0.000858972	2.67E-02
cg04619381	7	29603121	PRR15	0.21	0.000860307	2.68E-02
cg05555207	12	114847641	TBX5	0.22	0.000863537	2.68E-02
cg00231504	6	100906448	SIM1	0.20	0.000872456	2.70E-02
cg15781504	11	4871411	OR51S1	-0.20	0.000874133	2.70E-02
cg09427998	4	13530470		0.21	0.000874869	2.70E-02
cg17627629	5	179517140		0.21	0.000875909	2.71E-02

cg21200539	4	147562073	POU4F2	0.20	0.000882682	2.72E-02
cg22989843	2	223154201	PAX3	0.21	0.000899423	2.75E-02
cg27539060	6	137112464	MAP3K5	0.20	0.000901638	2.75E-02
cg13672078	5	87439429		0.23	0.000911608	2.77E-02
cg08460435	1	109203610	C1orf59	0.20	0.000922456	2.79E-02
cg11685316	17	8702564	MFSD6L	0.21	0.000923807	2.79E-02
cg04751149	2	66673449	MEIS1	0.21	0.000928177	2.80E-02
cg05385513	2	56150478	EFEMP1	0.21	0.000929071	2.80E-02
cg05789359	6	168377723	HGC6.3	-0.20	0.000934386	2.81E-02
cg01680010	7	96647117		0.23	0.000965313	2.87E-02
cg06482019	16	51147902		0.22	0.000974907	2.89E-02
cg16357224	16	86530053		0.20	0.000986055	2.91E-02
cg04822330	6	126069172		0.22	0.000986515	2.91E-02
cg12065840	8	121138133	COL14A1	0.21	0.00099243	2.92E-02
cg26728390	8	145777781	KIAA1688	-0.21	0.000996206	2.92E-02
cg05905176	15	51385916	TNFAIP8L3	0.22	0.0010036	2.94E-02
cg21545862	5	174159294		0.21	0.001014592	2.95E-02
cg18205770	5	87439353		0.23	0.001015333	2.96E-02
cg01176947	6	10385279		0.21	0.001024712	2.97E-02
cg02909688	1	147752823		0.20	0.001034855	2.99E-02
cg17078427	3	170137552	CLDN11	0.20	0.001036179	2.99E-02
cg02966475	16	65105516	CDH11	-0.20	0.001053461	3.03E-02
cg25887236	18	74844781	MBP	0.21	0.001054566	3.03E-02
cg07915434	10	102894342	TLX1	0.20	0.001059379	3.03E-02
cg13082654	21	47811350	PCNT	0.21	0.001064942	3.04E-02
cg22964918	7	27286075	EVX1	0.21	0.00106652	3.05E-02
cg04688351	2	223154140	PAX3	0.22	0.001076922	3.06E-02
cg05353869	11	75139544	KLHL35	0.21	0.00109651	3.10E-02
cg00486340	4	4860026	MSX1	0.20	0.001107008	3.12E-02
cg00110654	6	10385489		0.21	0.001123288	3.15E-02
cg27300028	13	100312810	CLYBL	0.21	0.001137087	3.17E-02
cg11738701	8	97167470	GDF6	0.21	0.001138096	3.17E-02
cg05065507	1	59042931	TACSTD2	0.20	0.001140367	3.17E-02
cg08900101	12	114833043	TBX5	0.20	0.001191076	3.26E-02
cg02551743	2	66673428	MEIS1	0.20	0.001251433	3.36E-02
cg21485303	12	22094699		0.23	0.001264398	3.38E-02
cg16423505	13	92051400	GPC5	0.22	0.001281047	3.41E-02
cg26695758	6	32063607	TNXB	0.20	0.001284365	3.41E-02
cg10705379	10	99080932	FRAT1	0.21	0.001289503	3.42E-02
cg08530237	12	22094563		0.23	0.001293399	3.42E-02
cg26626663	8	121137879	COL14A1	0.20	0.001293809	3.42E-02
cg04586407	16	31008574	STX1B	0.20	0.001295759	3.43E-02
cg21811204	5	132158625	SHROOM1	0.21	0.001326297	3.48E-02
cg24336989	6	10385903		0.22	0.00133277	3.49E-02
cg13300273	1	200842890	GPR25	0.23	0.00133458	3.49E-02
cg07312880	2	172430789		0.21	0.001347327	3.51E-02
cg14491820	10	102986808	LBX1	0.23	0.00140364	3.59E-02
cg09488203	10	95327884	GPR120	0.22	0.001424892	3.63E-02
cg26714388	12	34488729		-0.21	0.001435175	3.64E-02
cg06279276	16	67184164	B3GNT9	0.20	0.001437429	3.65E-02
cg19463256	19	46915351	CCDC8	0.21	0.001458565	3.68E-02
cg27555582	12	22094009		0.24	0.001466761	3.69E-02
cg04194674	17	36715953	SRCIN1	0.22	0.001467163	3.69E-02
cg08380311	19	3435252	NFIC	0.22	0.001467702	3.69E-02
cg14578879	6	100904055	SIM1	0.21	0.001503095	3.74E-02
cg09849405	16	66612955	CMTM1	0.21	0.001521037	3.77E-02
cg12288941	15	37175117	LOC145845	0.20	0.001521045	3.77E-02
cg12997720	7	27225058	HOXA11AS	0.21	0.001527498	3.78E-02
cg25461801	13	100630316		0.25	0.001551549	3.81E-02
cg02094500	6	62737607	KHDRBS2	-0.21	0.001572361	3.84E-02
cg06369992	10	119494494		0.20	0.001591919	3.87E-02
cg14269191	11	5346249	OR51B2	-0.20	0.001667418	3.97E-02
cg05990544	6	27841314	HIST1H4L	0.24	0.001680268	3.99E-02
cg25485294	6	27841324	HIST1H4L	0.22	0.001681737	4.00E-02
cg17242937	4	111561114		0.22	0.00168879	4.01E-02
cg25845597	6	27841122	HIST1H4L	0.21	0.001755544	4.11E-02

cg01085837	12	22094461		0.22	0.001765064	4.12E-02
cg11901043	12	22093960		0.21	0.001810438	4.19E-02
cg17611686	20	22547532	C20orf56	0.20	0.001851945	4.25E-02
cg03393426	10	102986686		0.20	0.001854457	4.25E-02
cg09550083	2	66672337	MEIS1	0.22	0.001867641	4.27E-02
cg01171339	6	27831894	HIST1H2AL	0.20	0.001870635	4.28E-02
cg21507095	22	19754448	TBX1	0.20	0.001940654	4.37E-02
cg06323023	6	27841352	HIST1H4L	0.21	0.001994247	4.45E-02
cg00804179	3	13083974	IQSEC1	-0.20	0.002339062	4.90E-02
cg25524962	13	20735819	GJA3	0.20	0.002406883	4.99E-02

Table S6. GO Biological processes enriched in DMPs in TYA of SHH tumours DMPs with High Methylation in TYA

Term_ID	Term_Name	Term_Size	Intersection_Size	FDR
GO:0009790	embryo development	1035	107	2.17E-26
GO:0048856	anatomical structure development	6106	310	1.39E-24
GO:0048598	embryonic morphogenesis	595	77	3.46E-24
GO:0032502	developmental process	6628	325	1.05E-23
GO:0007275	multicellular organism development	5613	289	8.55E-23
GO:0048731	system development	5052	266	2.23E-21
GO:0048568	embryonic organ development	432	62	2.47E-21
GO:0006357	regulation of transcription by RNA polymerase II	2555	170	3.25E-21
GO:0007389	pattern specification process	442	62	8.65E-21
GO:0007399	nervous system development	2449	164	1.56E-20
GO:0009653	anatomical structure morphogenesis	2797	177	7.46E-20
GO:0006366	transcription by RNA polymerase II	2723	172	5.19E-19
GO:0045944	positive regulation of transcription by RNA polymerase II	1197	103	6.33E-19
GO:0009887	animal organ morphogenesis	1073	96	1.36E-18
GO:0048562	embryonic organ morphogenesis	292	48	1.68E-18
GO:0030182	neuron differentiation	1412	111	1.47E-17
GO:0051254	positive regulation of RNA metabolic process	1714	125	1.73E-17
GO:0003002	regionalization	338	50	2.69E-17
GO:0048513	animal organ development	3672	205	2.97E-17
GO:0045165	cell fate commitment	267	44	8.25E-17
GO:0045893	positive regulation of transcription, DNA-templated	1617	118	2.73E-16
GO:1903508	positive regulation of nucleic acid-templated transcription	1617	118	2.73E-16
GO:1902680	positive regulation of RNA biosynthetic process	1618	118	2.87E-16
GO:0048699	generation of neurons	1559	115	3.89E-16
GO:0048869	cellular developmental process	4428	228	1.77E-15
GO:0030154	cell differentiation	4350	225	2.08E-15
GO:0032501	multicellular organismal process	7946	344	2.22E-15
GO:0022008	neurogenesis	1675	118	4.42E-15
GO:0006355	regulation of transcription, DNA-templated	3534	193	9.90E-15
GO:1903506	regulation of nucleic acid-templated transcription	3535	193	1.02E-14
GO:0009952	anterior/posterior pattern specification	211	37	1.04E-14
GO:2001141	regulation of RNA biosynthetic process	3540	193	1.20E-14
GO:0045935	positive regulation of nucleobase-containing compound metabolic process	1887	126	1.79E-14
GO:0051252	regulation of RNA metabolic process	3822	203	1.86E-14
GO:0010556	regulation of macromolecule biosynthetic process	4102	213	2.34E-14
GO:0010557	positive regulation of macromolecule biosynthetic process	1851	124	2.73E-14
GO:0009888	tissue development	2107	135	2.76E-14
GO:2000112	regulation of cellular macromolecule biosynthetic process	4071	210	1.03E-13
GO:0048706	embryonic skeletal system development	123	28	1.06E-13
GO:0031326	regulation of cellular biosynthetic process	4273	217	1.30E-13

GO:0006351	transcription, DNA-templated	3714	196	2.06E-13
GO:0097659	nucleic acid-templated transcription	3715	196	2.12E-13
GO:0009889	regulation of biosynthetic process	4361	219	3.16E-13
GO:0009891	positive regulation of biosynthetic process	2004	128	3.26E-13
GO:0032774	RNA biosynthetic process	3731	196	3.39E-13
GO:0031328	positive regulation of cellular biosynthetic process	1967	126	4.85E-13
GO:0048736	appendage development	176	32	6.57E-13
GO:0060173	limb development	176	32	6.57E-13
GO:0019219	regulation of nucleobase-containing compound metabolic process	4091	207	1.80E-12
GO:0009792	embryo development ending in birth or egg hatching	635	61	3.60E-12
GO:0030326	embryonic limb morphogenesis	119	26	4.11E-12
GO:0035113	embryonic appendage morphogenesis	119	26	4.11E-12
GO:0043009	chordate embryonic development	616	59	1.24E-11
GO:0000122	negative regulation of transcription by RNA polymerase II	901	74	1.27E-11
GO:0048704	embryonic skeletal system morphogenesis	94	23	1.27E-11
GO:0001708	cell fate specification	94	23	1.27E-11
GO:0035107	appendage morphogenesis	141	27	3.78E-11
GO:0035108	limb morphogenesis	141	27	3.78E-11
GO:0035270	endocrine system development	130	26	3.92E-11
GO:0001501	skeletal system development	504	51	1.14E-10
GO:0034654	nucleobase-containing compound biosynthetic process	4199	205	1.29E-10
GO:0048646	anatomical structure formation involved in morphogenesis	1202	86	1.85E-10
GO:0048468	cell development	2100	124	3.84E-10
GO:0018130	heterocycle biosynthetic process	4269	205	7.21E-10
GO:0035295	tube development	1146	82	7.66E-10
GO:0019438	aromatic compound biosynthetic process	4278	205	8.95E-10
GO:0007417	central nervous system development	1032	76	1.51E-09
GO:0031327	negative regulation of cellular biosynthetic process	1707	106	1.58E-09
GO:0060485	mesenchyme development	291	36	2.02E-09
GO:2000113	negative regulation of cellular macromolecule biosynthetic process	1628	102	2.77E-09
GO:0045892	negative regulation of transcription, DNA-templated	1337	89	3.80E-09
GO:1903507	negative regulation of nucleic acid-templated transcription	1339	89	4.13E-09
GO:0010558	negative regulation of macromolecule biosynthetic process	1639	102	4.17E-09
GO:0009893	positive regulation of metabolic process	3783	185	4.31E-09
GO:1902679	negative regulation of RNA biosynthetic process	1341	89	4.49E-09
GO:0048522	positive regulation of cellular process	5757	253	4.82E-09
GO:0009890	negative regulation of biosynthetic process	1740	106	5.23E-09
GO:0048705	skeletal system morphogenesis	226	31	5.24E-09
GO:1901362	organic cyclic compound biosynthetic process	4439	208	5.25E-09
GO:0007267	cell-cell signaling	1722	105	6.44E-09
GO:0050793	regulation of developmental process	2605	140	9.28E-09
GO:0051173	positive regulation of nitrogen compound metabolic process	3083	158	1.05E-08
GO:0031325	positive regulation of cellular metabolic process	3274	165	1.13E-08
GO:0048729	tissue morphogenesis	667	56	1.44E-08
GO:0060429	epithelium development	1325	87	1.48E-08
GO:0045595	regulation of cell differentiation	1679	102	1.77E-08
GO:0051253	negative regulation of RNA metabolic process	1445	92	1.83E-08
GO:0051239	regulation of multicellular organismal process	2861	148	3.36E-08
GO:0035239	tube morphogenesis	950	69	3.70E-08
GO:0009059	macromolecule biosynthetic process	5114	228	3.99E-08
GO:0048666	neuron development	1149	78	4.32E-08
GO:0072359	circulatory system development	1194	80	4.40E-08

GO:0010468	regulation of gene expression	5196	230	6.38E-08
GO:0048518	positive regulation of biological process	6363	268	8.97E-08
GO:0007423	sensory organ development	567	49	1.18E-07
GO:0034645	cellular macromolecule biosynthetic process	5051	224	1.23E-07
GO:0010604	positive regulation of macromolecule metabolic process	3483	169	1.31E-07
GO:0045934	negative regulation of nucleobase-containing compound metabolic process	1548	94	1.43E-07
GO:0050794	regulation of cellular process	11473	420	1.68E-07
GO:0060322	head development	798	60	2.26E-07
GO:0045596	negative regulation of cell differentiation	697	55	2.41E-07
GO:0045664	regulation of neuron differentiation	202	27	2.54E-07
GO:0021953	central nervous system neuron differentiation	188	26	2.62E-07
GO:0060021	roof of mouth development	88	18	2.91E-07
GO:0048762	mesenchymal cell differentiation	234	29	3.30E-07
GO:0051093	negative regulation of developmental process	977	68	3.41E-07
GO:0051171	regulation of nitrogen compound metabolic process	5848	248	5.53E-07
GO:0007420	brain development	754	57	5.61E-07
GO:0044271	cellular nitrogen compound biosynthetic process	5017	220	6.51E-07
GO:0000902	cell morphogenesis	1037	70	6.80E-07
GO:0007507	heart development	578	48	7.31E-07
GO:0002009	morphogenesis of an epithelium	560	47	7.94E-07
GO:0016070	RNA metabolic process	4710	209	8.97E-07
GO:0060562	epithelial tube morphogenesis	329	34	1.23E-06
GO:0048732	gland development	436	40	1.32E-06
GO:0097485	neuron projection guidance	284	31	1.89E-06
GO:0048858	cell projection morphogenesis	682	52	2.69E-06
GO:0061564	axon development	523	44	2.76E-06
GO:0048812	neuron projection morphogenesis	664	51	3.05E-06
GO:0048863	stem cell differentiation	258	29	3.37E-06
GO:0031175	neuron projection development	1012	67	3.79E-06
GO:0080090	regulation of primary metabolic process	6053	251	4.22E-06
GO:0051094	positive regulation of developmental process	1341	81	4.81E-06
GO:0007409	axonogenesis	478	41	5.89E-06
GO:0120039	plasma membrane bounded cell projection morphogenesis	678	51	6.21E-06
GO:0032990	cell part morphogenesis	699	52	6.29E-06
GO:0050789	regulation of biological process	12091	431	6.35E-06
GO:0007411	axon guidance	283	30	7.10E-06
GO:0040011	locomotion	2028	108	9.01E-06
GO:0007610	behavior	586	46	1.04E-05
GO:0031323	regulation of cellular metabolic process	6269	256	1.21E-05
GO:0048663	neuron fate commitment	64	14	1.32E-05
GO:0042127	regulation of cell population proliferation	1774	97	1.80E-05
GO:0048645	animal organ formation	66	14	2.02E-05
GO:0032989	cellular component morphogenesis	791	55	2.33E-05
GO:0048667	cell morphogenesis involved in neuron differentiation	602	46	2.39E-05
GO:0071542	dopaminergic neuron differentiation	38	11	2.40E-05
GO:0043583	ear development	218	25	3.05E-05
GO:0072001	renal system development	302	30	3.22E-05
GO:0045665	negative regulation of neuron differentiation	70	14	4.51E-05
GO:0030878	thyroid gland development	25	9	6.19E-05
GO:0021545	cranial nerve development	51	12	6.77E-05
GO:0042391	regulation of membrane potential	442	37	6.80E-05
GO:0030900	forebrain development	385	34	6.80E-05
GO:0021872	forebrain generation of neurons	62	13	7.96E-05

GO:0001656	metanephros development	86	15	9.89E-05
GO:0009058	biosynthetic process	6340	254	1.03E-04
GO:1901576	organic substance biosynthetic process	6248	251	1.05E-04
GO:0023052	signaling	6810	269	1.05E-04
GO:0021879	forebrain neuron differentiation	53	12	1.08E-04
GO:0001655	urogenital system development	337	31	1.11E-04
GO:0006928	movement of cell or subcellular component	2311	115	1.14E-04
GO:1905330	regulation of morphogenesis of an epithelium	64	13	1.19E-04
GO:0001709	cell fate determination	44	11	1.30E-04
GO:0008283	cell population proliferation	2054	105	1.33E-04
GO:0048332	mesoderm morphogenesis	76	14	1.36E-04
GO:0090304	nucleic acid metabolic process	5226	217	1.37E-04
GO:0007154	cell communication	6829	269	1.40E-04
GO:0065007	biological regulation	12809	445	1.50E-04
GO:0000904	cell morphogenesis involved in differentiation	749	51	1.61E-04
GO:0014706	striated muscle tissue development	380	33	1.62E-04
GO:0044249	cellular biosynthetic process	6154	247	1.67E-04
GO:0042471	ear morphogenesis	116	17	1.84E-04
GO:0061061	muscle structure development	645	46	1.90E-04
GO:0060255	regulation of macromolecule metabolic process	6603	261	2.05E-04
GO:0009880	embryonic pattern specification	67	13	2.11E-04
GO:0001822	kidney development	293	28	2.23E-04
GO:0072080	nephron tubule development	92	15	2.51E-04
GO:1904888	cranial skeletal system development	69	13	3.05E-04
GO:0035136	forelimb morphogenesis	38	10	3.08E-04
GO:0042733	embryonic digit morphogenesis	58	12	3.13E-04
GO:0045597	positive regulation of cell differentiation	879	56	3.34E-04
GO:0048523	negative regulation of cellular process	5139	212	3.82E-04
GO:0061326	renal tubule development	95	15	3.89E-04
GO:0098916	anterograde trans-synaptic signaling	705	48	3.98E-04
GO:0007268	chemical synaptic transmission	705	48	3.98E-04
GO:0001704	formation of primary germ layer	123	17	4.40E-04
GO:0055123	digestive system development	138	18	4.72E-04
GO:0003007	heart morphogenesis	251	25	4.91E-04
GO:0060537	muscle tissue development	399	33	5.06E-04
GO:0035019	somatic stem cell population maintenance	72	13	5.14E-04
GO:0035115	embryonic forelimb morphogenesis	31	9	5.18E-04
GO:0007369	gastrulation	185	21	5.23E-04
GO:0099537	trans-synaptic signaling	713	48	5.55E-04
GO:2000026	regulation of multicellular organismal development	1447	79	5.92E-04
GO:0099536	synaptic signaling	738	49	6.26E-04
GO:0001707	mesoderm formation	74	13	7.17E-04
GO:0009798	axis specification	87	14	7.85E-04
GO:0072028	nephron morphogenesis	75	13	8.44E-04
GO:0010463	mesenchymal cell proliferation	42	10	8.59E-04
GO:0048565	digestive tract development	129	17	8.82E-04
GO:0045666	positive regulation of neuron differentiation	101	15	8.86E-04
GO:0042476	odontogenesis	130	17	9.87E-04
GO:0009948	anterior/posterior axis specification	53	11	1.01E-03
GO:0003205	cardiac chamber development	161	19	1.07E-03
GO:0090596	sensory organ morphogenesis	262	25	1.11E-03
GO:0060017	parathyroid gland development	7	5	1.13E-03
GO:0001755	neural crest cell migration	54	11	1.23E-03

GO:0023061	signal release	496	37	1.23E-03
GO:0003279	cardiac septum development	104	15	1.31E-03
GO:0019222	regulation of metabolic process	7148	274	1.42E-03
GO:0070848	response to growth factor	759	49	1.43E-03
GO:0010646	regulation of cell communication	3543	155	1.44E-03
GO:0061138	morphogenesis of a branching epithelium	181	20	1.56E-03
GO:0001944	vasculature development	830	52	1.59E-03
GO:0060993	kidney morphogenesis	92	14	1.59E-03
GO:0003206	cardiac chamber morphogenesis	120	16	1.64E-03
GO:0006935	chemotaxis	653	44	1.75E-03
GO:0048519	negative regulation of biological process	5757	229	1.77E-03
GO:0048754	branching morphogenesis of an epithelial tube	151	18	1.84E-03
GO:0042330	taxis	656	44	1.98E-03
GO:0048870	cell motility	1828	92	2.07E-03
GO:0051674	localization of cell	1828	92	2.07E-03
GO:0072073	kidney epithelium development	137	17	2.09E-03
GO:0001568	blood vessel development	792	50	2.10E-03
GO:0048701	embryonic cranial skeleton morphogenesis	46	10	2.13E-03
GO:0031324	negative regulation of cellular metabolic process	2719	125	2.14E-03
GO:0010467	gene expression	6179	242	2.36E-03
GO:0023051	regulation of signaling	3578	155	2.63E-03
GO:0043010	camera-type eye development	331	28	2.68E-03
GO:0072009	nephron epithelium development	110	15	2.72E-03
GO:0071363	cellular response to growth factor stimulus	731	47	2.73E-03
GO:0007350	blastoderm segmentation	20	7	2.83E-03
GO:0060231	mesenchymal to epithelial transition	20	7	2.83E-03
GO:0001823	mesonephros development	97	14	3.07E-03
GO:0072078	nephron tubule morphogenesis	71	12	3.13E-03
GO:0051172	negative regulation of nitrogen compound metabolic process	2518	117	3.14E-03
GO:0014031	mesenchymal cell development	84	13	3.25E-03
GO:0050954	sensory perception of mechanical stimulus	174	19	3.52E-03
GO:0021602	cranial nerve morphogenesis	29	8	3.79E-03
GO:0048485	sympathetic nervous system development	21	7	4.14E-03
GO:0016477	cell migration	1649	84	4.15E-03
GO:0072088	nephron epithelium morphogenesis	73	12	4.25E-03
GO:0034330	cell junction organization	722	46	4.55E-03
GO:0030278	regulation of ossification	115	15	4.82E-03
GO:0009719	response to endogenous stimulus	1683	85	5.03E-03
GO:0001763	morphogenesis of a branching structure	195	20	5.04E-03
GO:0060675	ureteric bud morphogenesis	62	11	5.23E-03
GO:0051240	positive regulation of multicellular organismal process	1455	76	5.52E-03
GO:0006139	nucleobase-containing compound metabolic process	5746	226	5.58E-03
GO:0061333	renal tubule morphogenesis	75	12	5.72E-03
GO:0003151	outflow tract morphogenesis	75	12	5.72E-03
GO:0046483	heterocycle metabolic process	5904	231	5.87E-03
GO:0007498	mesoderm development	132	16	5.96E-03
GO:0072171	mesonephric tubule morphogenesis	63	11	6.16E-03
GO:0033993	response to lipid	918	54	6.51E-03
GO:0065008	regulation of biological quality	4131	172	6.73E-03
GO:0060541	respiratory system development	199	20	6.90E-03
GO:0009855	determination of bilateral symmetry	134	16	7.27E-03
GO:0022603	regulation of anatomical structure morphogenesis	1042	59	7.36E-03
GO:0048880	sensory system development	389	30	7.56E-03

GO:0021675	nerve development	77	12	7.61E-03
GO:0031016	pancreas development	77	12	7.61E-03
GO:0009799	specification of symmetry	135	16	8.02E-03
GO:2000027	regulation of animal organ morphogenesis	184	19	8.14E-03
GO:0044058	regulation of digestive system process	42	9	8.50E-03
GO:0071495	cellular response to endogenous stimulus	1422	74	8.71E-03
GO:0021537	telencephalon development	256	23	8.81E-03
GO:0019827	stem cell population maintenance	152	17	8.93E-03
GO:0001657	ureteric bud development	92	13	9.27E-03
GO:0001508	action potential	137	16	9.73E-03
GO:0072164	mesonephric tubule development	93	13	1.05E-02
GO:0072163	mesonephric epithelium development	93	13	1.05E-02
GO:0098727	maintenance of cell number	154	17	1.07E-02
GO:0071773	cellular response to BMP stimulus	171	18	1.11E-02
GO:0071772	response to BMP	171	18	1.11E-02
GO:0014032	neural crest cell development	80	12	1.15E-02
GO:0006725	cellular aromatic compound metabolic process	5956	231	1.18E-02
GO:0007626	locomotory behavior	189	19	1.21E-02
GO:0001654	eye development	379	29	1.28E-02
GO:0051241	negative regulation of multicellular organismal process	1160	63	1.30E-02
GO:0060411	cardiac septum morphogenesis	68	11	1.34E-02
GO:0001764	neuron migration	157	17	1.39E-02
GO:0001658	branching involved in ureteric bud morphogenesis	56	10	1.42E-02
GO:0007368	determination of left/right symmetry	126	15	1.52E-02
GO:0072006	nephron development	142	16	1.55E-02
GO:0150063	visual system development	383	29	1.57E-02
GO:0007351	tripartite regional subdivision	17	6	1.59E-02
GO:0048486	parasympathetic nervous system development	17	6	1.59E-02
GO:0008595	anterior/posterior axis specification, embryo	17	6	1.59E-02
GO:0048839	inner ear development	193	19	1.64E-02
GO:1901360	organic cyclic compound metabolic process	6201	238	1.68E-02
GO:0061371	determination of heart left/right asymmetry	70	11	1.79E-02
GO:0035282	segmentation	98	13	1.89E-02
GO:0048864	stem cell development	84	12	1.93E-02
GO:0048589	developmental growth	649	41	2.03E-02
GO:0001667	ameboidal-type cell migration	494	34	2.05E-02
GO:0040012	regulation of locomotion	1104	60	2.15E-02
GO:2001053	regulation of mesenchymal cell apoptotic process	11	5	2.24E-02
GO:0090066	regulation of anatomical structure size	518	35	2.26E-02
GO:0048483	autonomic nervous system development	47	9	2.27E-02
GO:0060457	negative regulation of digestive system process	18	6	2.32E-02
GO:0003008	system process	2293	105	2.39E-02
GO:0048538	thymus development	48	9	2.72E-02
GO:0098742	cell-cell adhesion via plasma-membrane adhesion molecules	277	23	3.22E-02
GO:0021761	limbic system development	103	13	3.27E-02
GO:0010002	cardioblast differentiation	19	6	3.31E-02
GO:0042474	middle ear morphogenesis	19	6	3.31E-02
GO:0072074	kidney mesenchyme development	19	6	3.31E-02
GO:0046879	hormone secretion	317	25	3.36E-02
GO:0030030	cell projection organization	1609	79	3.36E-02
GO:0032879	regulation of localization	2872	125	3.38E-02
GO:0016331	morphogenesis of embryonic epithelium	151	16	3.40E-02
GO:0048514	blood vessel morphogenesis	711	43	3.57E-02

GO:0030072	peptide hormone secretion	260	22	3.67E-02
GO:0035148	tube formation	152	16	3.69E-02
GO:0098911	regulation of ventricular cardiac muscle cell action potential	12	5	3.75E-02
GO:0097152	mesenchymal cell apoptotic process	12	5	3.75E-02
GO:0035176	social behavior	50	9	3.84E-02
GO:0021515	cell differentiation in spinal cord	50	9	3.84E-02
GO:0060688	regulation of morphogenesis of a branching structure	50	9	3.84E-02
GO:0014033	neural crest cell differentiation	90	12	3.96E-02
GO:0042475	odontogenesis of dentin-containing tooth	90	12	3.96E-02
GO:0007605	sensory perception of sound	153	16	4.01E-02
GO:0048738	cardiac muscle tissue development	224	20	4.07E-02
GO:0044057	regulation of system process	623	39	4.13E-02
GO:0003156	regulation of animal organ formation	29	7	4.49E-02
GO:0060039	pericardium development	20	6	4.60E-02
GO:0010817	regulation of hormone levels	536	35	4.65E-02

DMPs with Low Methylation in TYA

Term_ID	Term_Name	Term_Size	Intersection_Size	FDR
GO:0050911	detection of chemical stimulus involved in sensory perception of smell	397	48	2.88E-29
GO:0007608	sensory perception of smell	425	49	5.72E-29
GO:0050907	detection of chemical stimulus involved in sensory perception	446	48	6.46E-27
GO:0009593	detection of chemical stimulus	483	49	2.36E-26
GO:0007606	sensory perception of chemical stimulus	497	49	8.91E-26
GO:0050906	detection of stimulus involved in sensory perception	512	48	3.46E-24
GO:0051606	detection of stimulus	687	51	3.49E-21
GO:0007600	sensory perception	958	53	2.83E-16
GO:0007186	G protein-coupled receptor signaling pathway	1355	60	3.39E-14
GO:0050877	nervous system process	1426	59	1.54E-12
GO:0003008	system process	2293	64	3.49E-06
GO:0007154	cell communication	6829	129	1.71E-04
GO:0023052	signaling	6810	128	2.89E-04
GO:0042221	response to chemical	4781	99	3.02E-04
GO:0007165	signal transduction	6309	121	3.05E-04
GO:0050896	response to stimulus	9407	161	5.25E-04
GO:0032501	multicellular organismal process	7946	141	1.31E-03
GO:0051716	cellular response to stimulus	7781	138	2.27E-03

Table S7. GO Biological processes enriched in DMPs in TYA of Group 3 tumours DMPs with High Methylation in TYA

Term_ID	Term_Name	Term_Size	Intersection_Size	FDR
GO:0007420	brain development	754	25	4.88E-04
GO:0006366	transcription by RNA polymerase II	2723	55	6.26E-04
GO:0006357	regulation of transcription by RNA polymerase II	2555	52	1.19E-03
GO:0032502	developmental process	6628	101	1.20E-03
GO:0051254	positive regulation of RNA metabolic process	1714	39	3.27E-03
GO:0007399	nervous system development	2449	49	4.69E-03
GO:0030900	forebrain development	385	16	4.99E-03
GO:0006355	regulation of transcription, DNA-templated	3534	63	5.27E-03
GO:1903506	regulation of nucleic acid-templated transcription	3535	63	5.32E-03
GO:2001141	regulation of RNA biosynthetic process	3540	63	5.59E-03
GO:1903508	positive regulation of nucleic acid-templated transcription	1617	37	5.65E-03
GO:0045893	positive regulation of transcription, DNA-templated	1617	37	5.65E-03

GO:1902680	positive regulation of RNA biosynthetic process	1618	37	5.73E-03
GO:0048856	anatomical structure development	6106	93	6.22E-03
GO:0006351	transcription, DNA-templated	3714	65	6.37E-03
GO:0097659	nucleic acid-templated transcription	3715	65	6.43E-03
GO:0048731	system development	5052	81	6.48E-03
GO:0032774	RNA biosynthetic process	3731	65	7.48E-03
GO:0007417	central nervous system development	1032	27	1.47E-02
GO:0007275	multicellular organism development	5613	86	1.74E-02
GO:0030154	cell differentiation	4350	71	2.25E-02
GO:0045944	positive regulation of transcription by RNA polymerase II	1197	29	2.81E-02
GO:0021537	telencephalon development	256	12	2.89E-02
GO:0045935	positive regulation of nucleobase-containing compound metabolic process	1887	39	3.24E-02
GO:0051252	regulation of RNA metabolic process	3822	64	3.54E-02
GO:0010467	gene expression	6179	91	4.13E-02
GO:0048869	cellular developmental process	4428	71	4.26E-02

DMPs with Low Methylation in TYA

Term_ID	Term_Name	Term_Size	Intersection_Size	FDR
GO:0030198	extracellular matrix organization	401	19	3.74E-05
GO:0043062	extracellular structure organization	402	19	3.89E-05
GO:0045229	external encapsulating structure organization	404	19	4.22E-05

Table S8. Prognostic gene signature. The 22-genes signature that are strongly associated with the overall survival of medulloblastoma patients (n = 729). The risk score derived from the signature stratifies patients into low- and high-risk patients.

Gene Symbol	Gene Description	NCBI Gene ID	CHR	Karyotype Band
PIK3C2G	phosphatidylinositol-4-phosphate 3-kinase catalytic subunit type 2 gamma	5288	12	p12.3
SEC14L1	SEC14 like lipid binding 1	6397	17	q25.2
E2F6	E2F transcription factor 6	1876	2	p25.1
PHLPP1	PH domain and leucine rich repeat protein phosphatase 1	23239	18	q21.33
CDKN1B	cyclin dependent kinase inhibitor 1B	1027	12	p13.1
TIPARP	TCDD inducible poly (ADP-ribose) polymerase	25976	3	q25.31
MRTO4	MRT4 homolog, ribosome maturation factor	51154	1	p36.13
GUCY1A2	guanylate cyclase 1 soluble subunit alpha 2	2977	11	q22.3
CITED1	Cbp/p300 interacting transactivator with Glu/Asp rich carboxy-terminal domain 1	4435	X	q13.1
SCD5	stearoyl-CoA desaturase 5	79966	4	q21.22
CACNB2	calcium voltage-gated channel auxiliary subunit beta 2	783	10	p12.33
EPHA6	EPH receptor A6	285220	3	q11.2
GJD2	gap junction protein delta 2	57369	15	q14
MTMR8	myotubularin related protein 8	55613	X	q11.2
DNAH2	dynein axonemal heavy chain 2	146754	17	p13.1
RAB34	RAB34, member RAS oncogene family	83871	17	q11.2
LFNG	LFNG O-fucosylpeptide 3-beta-N-acetylglucosaminyltransferase	3955	7	p22.3
PLD2	phospholipase D2	5338	17	p13.2
FZD2	frizzled class receptor 2	2535	17	q21.31
AP1G2	adaptor related protein complex 1 subunit gamma 2	8906	14	q11.2
F8	coagulation factor VIII	2157	X	q28

Table S9. Univariate and multivariate Cox proportional hazard ratios for gender, molecular subgroup, and risk score for TYA in medulloblastoma.

		Univariate analysis		Multivariate analysis	
**Variable**		**HR (95% CI)**	**P-value** [a]	**HR (95% CI)**	**P-value**
Gender					
	Female	Reference		Reference	
	Male	0.56 (0.23 - 1.34)	0.192	0.79 (0.30 - 2.08)	0.636
Subgroup					
	WNT	Reference		Reference	
	SHH	2.49 (0.51 - 12.01)	0.257	1.21 (0.20 - 7.28)	0.839
	G3	0.99 (0.09 - 10.96)	0.992	0.08 (0.004 - 1.57)	0.096
	G4	2.20 (0.48 - 10.04)	0.310	0.17 (0.02 - 1.62)	0.123
Risk score					
	High	Reference		Reference	
	Low	0.34 (0.12 - 0.94)	0.037[b]	0.07 (0.01 - 0.34)	0.001

Abbreviations: CI = confidence interval; HR = hazard ratio. [a] P-value is obtained from the Wald test. likelihood ratio test. [b] The likelihood ratio test = 5.02 on 1 df, P-value = 0.025.

Table S10. C-index for univariate and multivariate Cox proportional models for TYA in medulloblastoma.

	Univariate analysis	Multivariate analysis		
**Variable**	**C-index**		**C-index**	**Validation C-index**
Gender	0.55	Model: *Gender + Subgroup + Risk score*	0.70	0.68
Subgroup	0.57			
Risk score	0.64			

Abbreviations: C-index = concordance index; Validation C-index = C-index obtained from resampling of TYA patient data (n = 1000).
